# Convenient Synthesis of 6,7,12,13-Tetrahydro-5*H*-Cyclohepta[2,1-*b*:3,4-*b’*]diindole Derivatives Mediated by Hypervalent Iodine (III) Reagent

**DOI:** 10.3390/molecules24050960

**Published:** 2019-03-08

**Authors:** Lei Peng, Xiaofei Zhang, Chunhao Yang

**Affiliations:** 1 State Key Laboratory of Drug Research, Department of Medicinal Chemistry, Shanghai Institute of Materia Medica, Chinese Academy of Sciences, 555 Zu Chong Zhi Road, Shanghai 201203, China; leipeng@simm.ac.cn; 2 School of Pharmacy, University of Chinese Academy of Sciences, No.19A Yuquan Road, Beijing 100049, China

**Keywords:** phenyliodine(III)bis(trifluoroacetate), cyclohepta[2,1-*b*:3,4-*b’*]diindole, intramolecular oxidative coupling

## Abstract

Bisindolyl alkaloids represent a large family of natural and synthetic products that display various biological activities. Among the bisindole compounds, 6,7,12,13-tetrahydro-5*H*-cyclohepta[2,1-*b*:3,4-*b’*]diindoles have received little attention. Only two methods have been developed for the construction of the 6,7,12,13-tetrahydro-5*H*-cyclohepta[2,1-*b*:3,4-*b’*]diindole scaffold thus far, including the classical Fischer indole synthesis conducted by reacting indole-fused cycloheptanone and hydrazines, and the condensation reaction to build the seven-membered ring. Here, we report for the first time a new route to synthesize 6,7,12,13-tetrahydro-5*H*-cyclohepta[2,1-*b*:3,4-*b’*]diindoles through intramolecular oxidative coupling of 1,3-di(1*H*-indol-3-yl)propanes in the presence of PIFA, DDQ and TMSCl with moderate to excellent yields.

## 1. Introduction

**Figure 1:**
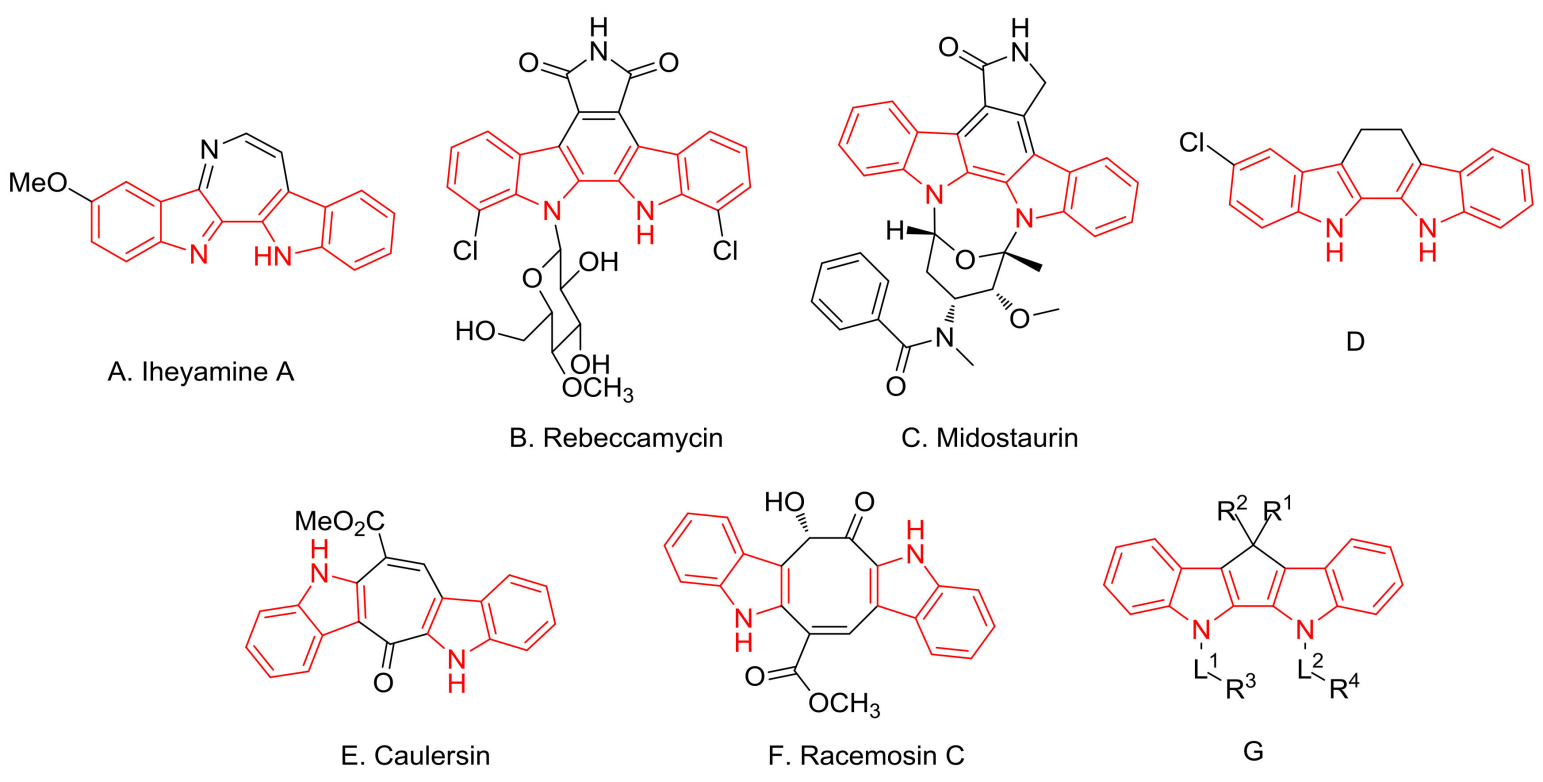
Some bioactive bisindolyl alkaloids and synthetic compounds.

The bisindole core constitutes a valuable class of biologically active molecules that are prevalent in nature [1,2,3,4,5,6]. These natural and synthetic compounds display various biological activities such as antitumor, antimicrobial and antidiabetic. For example, Iheyamine A (Figure 1A), an alkaloid isolated from a colonial ascidian *Polycitorella sp.*, shows interesting moderate cytotoxicity [7], and Rebeccamycin (Figure 1B), an antitumor antibiotic isolated from cultures of *Saccharotrix aerocolonigenes*, is well-known for its inhibitory potency toward topoisomerase I [8,9,10]. In addition, natural product derivative Midostaurin (Figure 1C) has been approved by the US FDA for the treatment of FLT3-mutated acute myelogenous leukemia (AML) [11], and synthetic indolocarbazole derivative D (Figure 1D) shows excellent antibacterial activity and good antifungal activity [12]. Furthermore, Caulersin (Figure 1E) from the alga *Caulerpa serrulate* [13,14,15] and Racemosin C (Figure 1F) isolated from the green alga *Caulerpa racemose* [16] exhibit significant PTP1B inhibitory activity. It is worth noting that both compounds E and F contain a medium ring besides the bisindole structure. The bisindole core also has applications in material science. For example, the synthetic cyclopenta[2,1-*b*:3,4-*b*’]diindole derivatives (Figure 1G) show excellent luminous efficiency as organic electroluminescent compounds [17].

**Scheme 1:**
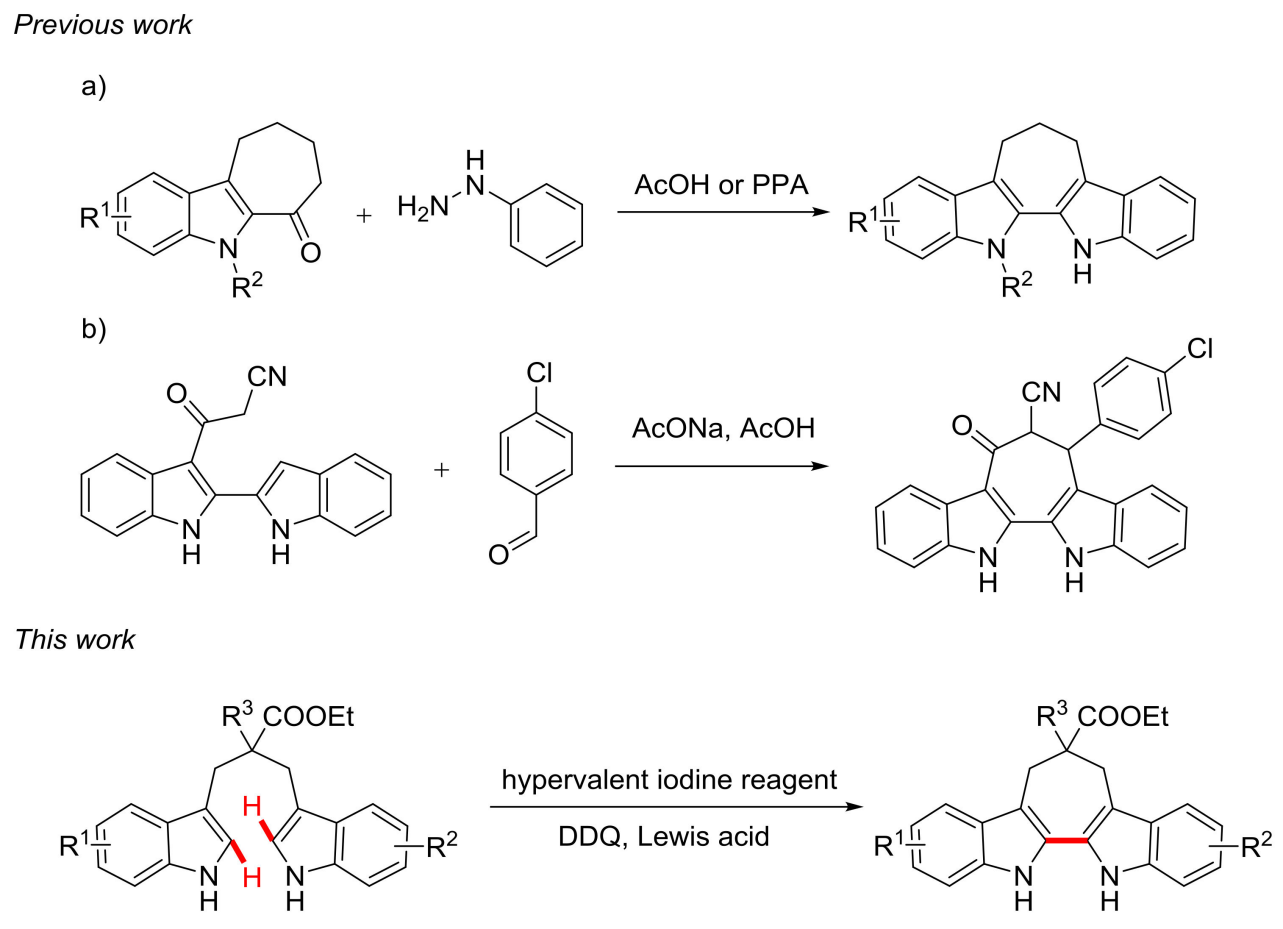
Methods for the synthesis of 6,7,12,13-tetrahydro-5*H*-cyclohepta[2,1-*b*:3,4-*b***’**]diindoles.

Among the bisindole compounds, the rarely explored 6,7,12,13-tetrahydro-5*H*-cyclohepta[2,1-*b*:3,4-*b’*]diindoles attracted our interest. To the best of our knowledge, only two methods have been reported to construct 6,7,12,13-tetrahydro-5*H*-cyclohepta[2,1-*b*:3,4-*b’*]diindoles. One was the establishment of the second indole by classic Fischer indole synthesis, which usually suffered from an excess of acids, hazardous hydrazines, and a limited number of indole-fused cycloheptanones (Scheme 1a) [18,19,20]. The other approach was the construction of seven-membered ring by condensation reaction, and this process strictly required specific functional groups on the substrates (Scheme 1b) [21].

Our group has been focused on the synthesis of natural product analogues for bioactive screening for many years [22,23,24,25,26]. Thus we hoped to develop a convenient and practical method for the synthesis of 6,7,12,13-tetrahydro-5*H*-cyclohepta[2,1-*b*:3,4-*b’*]diindoles. Inspired by previous work, the hypervalent iodine reagents, such as phenyliodine(III)bis(trifluoroacetate) (PIFA) and phenyliodine(III)diacetate (PIDA), have been widely applied to organic synthesis due to its mild oxidation capacity, low toxicity and environmentally benign properties [27,28]. Intermolecular oxidative coupling reactions of aromatic rings mediated by hypervalent iodine reagents have been studied by many researchers and many excellent examples have been reported, including heteroaromatic couplings [29,30,31,32,33,34], cross-couplings of heteroaromatic and aromatic rings [35,36,37] and aromatic couplings [38,39,40,41]. In the meantime, some progress have been achieved in intramolecular oxidative coupling reactions mediated by hypervalent iodine reagents, including the establishment of spiro structures [42,43,44] and fused rings [45,46,47,48]. Constructing a seven-membered ring through intramolecular oxidation is still highly desirable. Herein, we report for the first time a new route to synthesize 6,7,12,13-tetrahydro-5*H*-cyclohepta[2,1-*b*:3,4-*b’*]diindoles by intramolecular oxidative coupling of 1,3-di(1*H*-indol-3-yl)propanes in the presence of phenyliodine(III)bis(trifluoroacetate) (PIFA), 2,3-dichloro-5,6-dicyano-1,4-benzoquinone (DDQ), and trimethylsilyl chloride (TMSCl). This convenient and practical protocol shows good functional-group tolerance with moderate to excellent yields.

## 2. Results and Discussion

### 2.1. Preparation of 1,3-di(1H-indol-3-yl)propanes

**Scheme 2:**
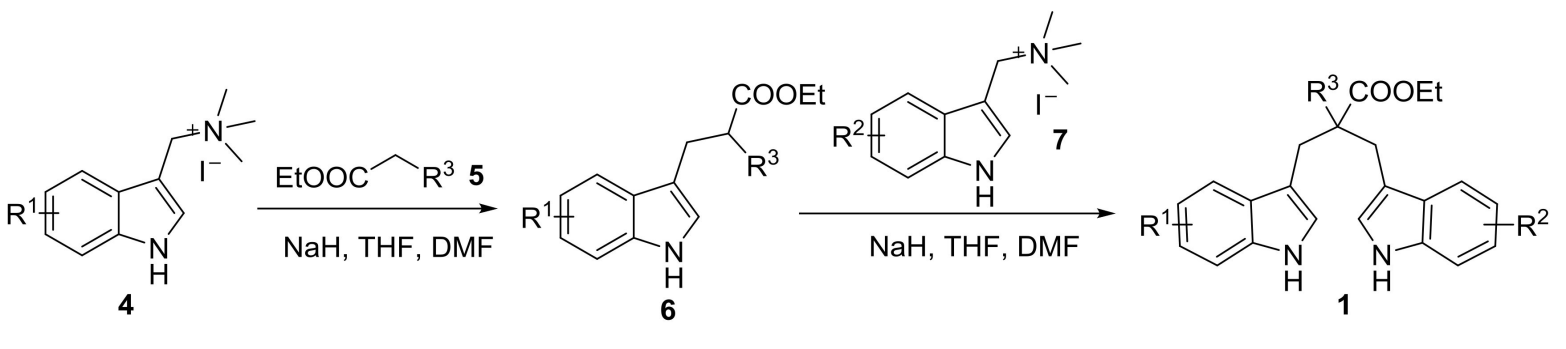
Synthetic route to 1,3-di(1*H*-indol-3-yl)propanes **1**.

First, we managed to prepare 1,3-di(1*H*-indol-3-yl)propanes **1** by two steps with high yielded substitution reaction between the indole trimethyl quaternary ammonium salts and esters **5** [49]. The synthetic route is shown in Scheme 2. The experimental characterization data are given in the Supplementary Materials.

### 2.2. Intramolecular Oxidative Coupling of 1,3-di(1H-indol-3-yl)propanes

Our studies initiated with the reaction of diethyl 2,2-bis((1*H*-indol-3-yl)methyl)malonate (**1a**) in the presence of PIFA and TMSBr in dichloromethane (DCM), the reaction mixture was stirred at 0 °C for 4 h, and then warmed to room temperature to stir for 12 h (Table 1, entry 1). To our delight, the desired product **2a** was isolated with 55% yield, and a trace of nonaromatic intermediate **3a** was detected. Interestingly, reducing the usage of PIFA did not affect the total yield of **2a** and **3a**, but the yield of **3a** was slightly increased (Table 1, entries 2–3). When 0.3 equiv. of PIFA was used and the reaction was performed under argon atmosphere, the total yield was decreased, but the yield of **3a** was improved (Table 1, entry 4 vs. 3). To suppress the formation of intermediate **3a** and decrease the loading of PIFA, the additional oxidant DDQ was employed (Table 1, entries 5–7). When 0.3 equiv. of PIFA and 0.5 equiv. of DDQ were added, the reaction yield of **2a** was improved to 60% (Table 1, entry 7). By replacing DDQ with BQ (1,4-benzoquinone) or CAN (ceric ammonium nitrate), the yield of desired product was decreased (Table 1, entries 8–9). Moreover, increasing the DDQ loading resulted in a reduced yield (Table 1, entries 5–6). Notably, when only DDQ was added as oxidant, the reaction also generated **2a** with 48% yield (Table 1, entry 10). In general, we found that 0.3 equiv. of PIFA and 0.5 equiv. of DDQ was the most favorable combination.

Next, a set of Lewis acids were examined at 0 °C. The yield was slightly increased when TMSBr was replaced by TMSCl (Table 2, entry 2 *vs* 1), while TMSOTf and BF_3_ OEt_2_ reduced the yield down to 23% and 49% (Table 2, entries 3–4). Moreover, lower temperatures turned out to be better for the reaction with a yield of 88% at −40 °C and 70% at −78 °C, respectively (Table 2, entries 5–6). Subsequently, an investigation of different hypervalent iodine reagents was performed at −40 °C. It revealed that the use of PIDA decreased the reaction yield slightly (85%, Table 2, entry 7), and the yield was reduced when IBX (2-iodoxybenzoic acid) and DMP (Dess-Martin periodinane) were used (Table 2, entries 8, 9). Finally, the optimized condition for the synthesis of 6,7,12,13-tetrahydro-5*H*-cyclohepta[2,1-*b*:3,4-*b’*]diindoles from 1,3- di(1*H*-indol-3-yl)propanes was obtained: ethyl 2-((1*H*-indol-3-yl)methyl)-3-(1*H*-indol-3-yl)propanoate **1a** (1 equiv.), PIFA (0.3 equiv.), DDQ (0.5 equiv.), and TMSCl (1 equiv.), the reaction mixture was stirred at −40 °C under air atmosphere for 4 h, and then warmed to room temperature and stirred for 12 h.

**Scheme 3:**
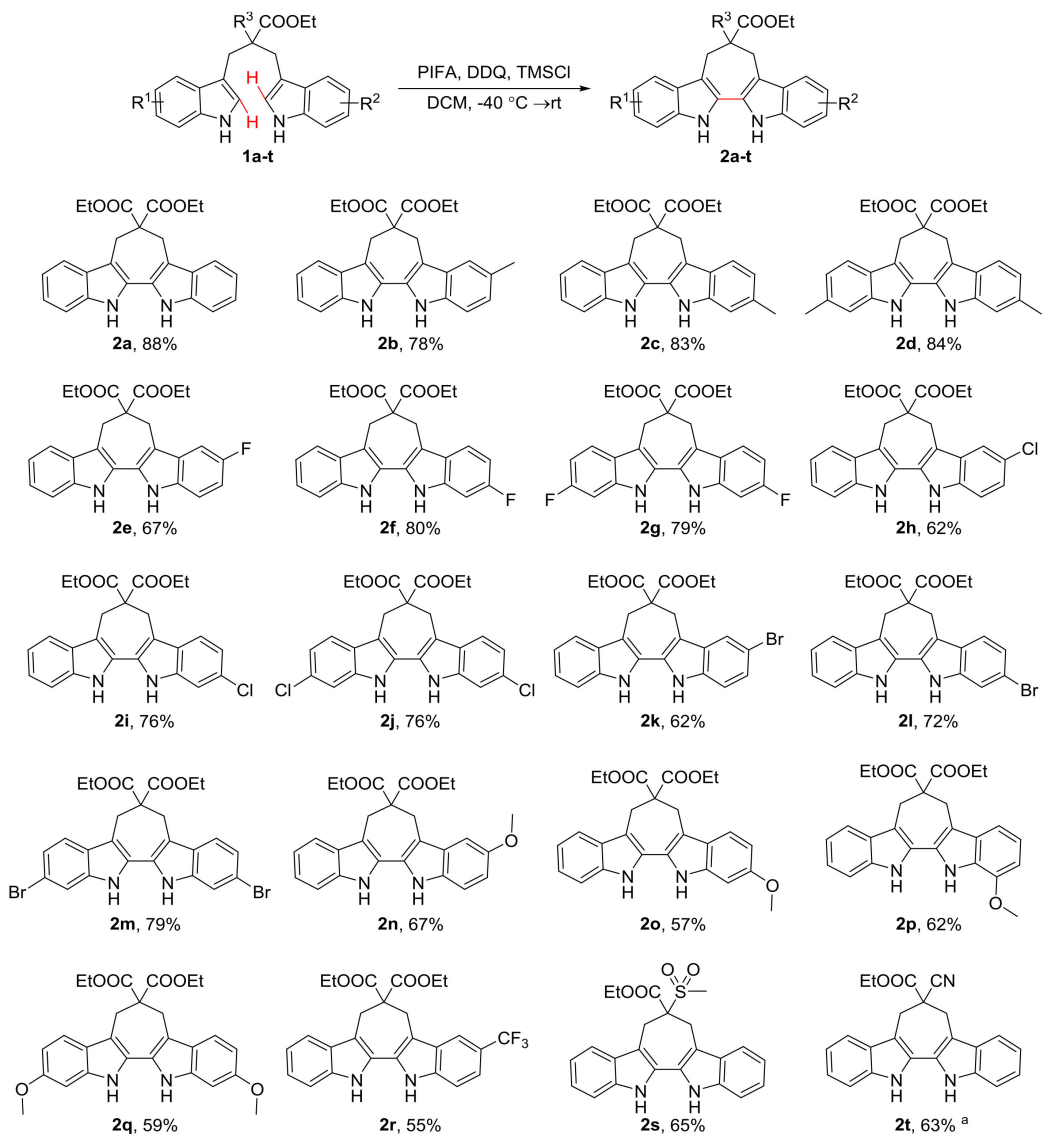
The substrate scope for the synthesis of 6,7,12,13-tetrahydro-5*H*-cyclohepta[2,1-*b*:3,4-*b’*]diindoles. ^a^ Conversion yield.

**Scheme 4:**
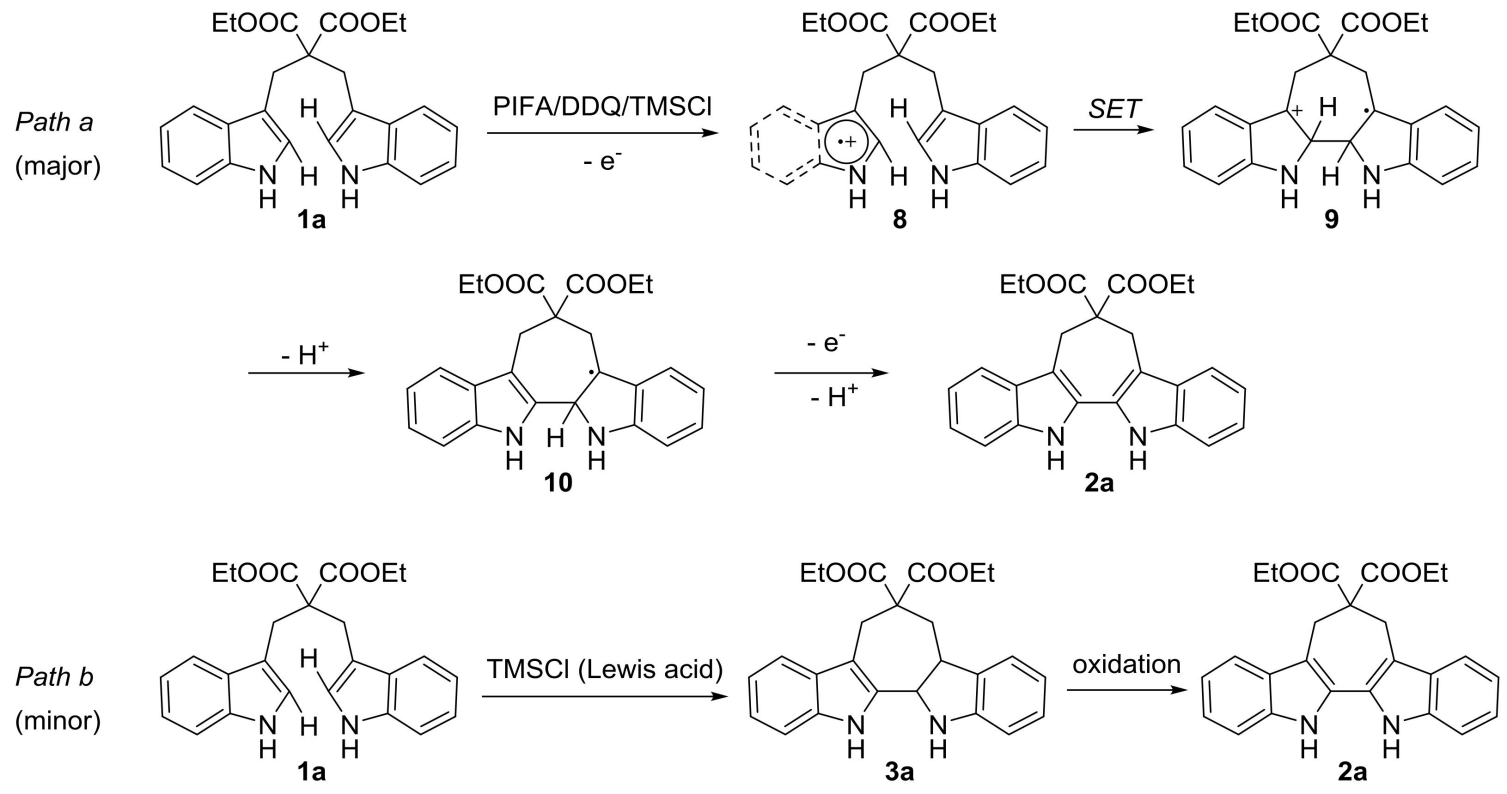
Plausible reaction mechanism.

With the optimized reaction conditions in hand, we further examined the substrate scope of the 1,3-di(1*H*-indol-3-yl)propane derivatives as shown in Scheme 3. The 1,3-di(1*H*-indol-3-yl)propanes substituted by both electron-donating groups (-Me and -OMe) and electron-withdrawing groups (-F, -Cl, -Br and -CF_3_) on the indole ring were investigated. Both the electron-donating (-Me) and electron-withdrawing groups (-F, -Cl, and -Br) at the C5/C6 positions of the indole substrates reacted smoothly to provide the target products with good to excellent yields. The substitution position had significant influence on the reaction yield: C6-substituted indoles generated the corresponding products (**2c**, **2f**, **2i**, and **2l**) in higher yields than the C5 substituted substrates (**2b**, **2e**, **2h**, and **2k**). It is worth noting that, as an interesting exception, a -OMe group on the C5, C6 and C7 positions gave similar yields under the optimized conditions (**2n**, **2o** and **2p**). Moreover, for substituents -Me, -F, -Cl, -Br and -OMe, reactions conducted on symmetrical substrates (**2d**, **2g**, **2j**, **2m** and **2q**) showed moderate to excellent yields similar to asymmetrical ones (**2c**, **2f**, **2i**, **2l** and **2o**). As for strong electron-withdrawing groups, the indole bearing a C5 CF_3_ group showed a moderate yield (**2r**, 55%). These results may be determined by the stability of cationic radical intermediate involved in the reaction (Scheme 4). To further explore the scope of the substrates, the starting materials bearing -SO_2_CH_3_ or -CN in place of -COOEt for R^3^ were also employed and were found to afford good yields, respectively (**2s**, 65% and **2t**, 63%).

Inspired by the mature mechanism research of oxidative coupling reaction mediated by hypervalent iodine reagents [29,30], a possible mechanism for the synthesis of 6,7,12,13-tetrahydro-5*H*-cyclohepta[2,1-*b*:3,4-*b’*]diindoles from 1,3-di(1*H*-indol-3-yl)propanes in the presence of PIFA, DDQ and TMSCl was proposed in Scheme 4. Initially, the radical cation was formed from **1a** with PIFA/DDQ/TMSCl. Intermediate **9** was then produced by intramolecular single electron transfer (SET), and deprotonated intermediate **10** was formed subsequently. Finally, product **2a** was generated via a further one-electron oxidation and deprotonation process (Scheme 4, Path a). Additionally, a small amount of intermediate **3a** was formed during the reaction (confirmed by LC-MS) [30,50], which may have resulted from the protonation and Friedel-Crafts type reaction of substrate **1a** in the presence of Lewis acid. This intermediate was subsequently oxidized to give product **2a** (Scheme 4, path b).

## 3. Materials and Methods 

### 3.1. General Information

All the chemical reagents were commercial products and were used without purification in all cases. TLC was performed on silica gel plates (0.15–0.2 mm thickness, Yantai Huiyou Company, Yantai, China) and detected with UV light at 254 nM, Column chromatography was carried out on silica gel (200–300 mesh). Proton and carbon magnetic resonance spectra (^1^H-NMR and ^13^C-NMR) were recorded on Varian Mercury-300, Varian Mercury-400, Varian Mercury-500 and/or Varian Mercury-600 spectrometers (Palo Alto, CA, United States). NMR experiments were conducted in CDCl_3_, CD_3_OD and DMSO-*d*_6_. Tetramethylsilane (TMS) was used as the internal standard. Chemical shifts (δ) are reported in parts per million (ppm). Data are reported as follows: chemical shift, multiplicity (br s = broad singlet, d = doublet, dd = doublet of doublet, dt = doublet of triplet, m = multiple, s = singlet and t = triplet), coupling constants (Hz). Low-resolution mass spectra (ESI) were obtained using Agilent HPLC-MS (Palo Alto, CA, United States) (1200-6110). High resolution mass spectra (HRMS) were obtained using Agilent 1290-6545 UHPLC-QTOF. Melting points (mp) were measured by Büchi 510 melting point apparatus without further correction.

### 3.2. General Procedure for the Synthesis of 6,7,12,13-Tetrahydro-5H-Cyclohepta[2,1-b:3,4-b’]diindoles *(**2a**–**t**)*

PIFA (0.1 mmol, 43 mg), DDQ (0.15 mmol, 34 mg) and TMSCl (0.3 mmol, 38 μL) were added to the stirred solution of 1,3-di(1*H*-indol-3-yl)propane **1** (0.3 mmol) in dichloromethane (2 mL) at −40 °C in sequence. The mixture was stirred under air at the same temperature for 4 h and then warmed to room temperature (25 °C) and stirred for an additional 12 h. After completion of the reaction, the mixture was diluted with aqueous NaHCO_3_ solution and extracted with dichloromethane. After drying with anhydrous Na_2_SO_4_, dichloromethane was removed under reduced pressure. The residue was purified by column chromatography on silica gel using petroleum ether/ethyl acetate (10:1 to 5:1, *v/v*) as the eluent to provide the product **2**.

*Diethyl 5,7,12,13-tetrahydro-6H-cyclohepta[2,1-b:3,4-b’]diindole-6,6-dicarboxylate* (**2a**). Yellow solid, 110 mg (yield 88%). mp 187–189 °C. ^1^H-NMR (400 MHz, DMSO-*d*_6_) δ 10.93 (s, 2H, NH), 7.54 (d, *J* = 7.9 Hz, 2H, Ar-H), 7.41 (d, *J* = 8.0 Hz, 2H, Ar-H), 7.12 (ddd, *J* = 8.1, 6.9, 1.2 Hz, 2H, Ar-H), 7.04 (ddd, *J* = 7.9, 6.9, 1.1 Hz, 2H, Ar-H), 3.94 (q, *J* = 7.1 Hz, 4H, -CO_2_C*H*_2_CH_3_), 3.54 (s, 4H, C_q_-CH_2_Ar), 0.88 (t, *J* = 7.1 Hz, 6H, -CO_2_CH_2_C*H*_3_). ^13^C-NMR (125 MHz, DMSO-*d*_6_) δ 170.40 (C=O), 135.67 (C), 128.75 (C), 127.22 (C), 121.75 (CH), 119.11 (CH), 117.76 (CH), 110.95 (CH), 108.62 (C), 60.79 (OCH_2_), 54.36 (C), 30.72 (CH_2_), 13.54 (CH_3_). HRMS (ESI): *m/z* calcd for C_25_H_25_N_2_O_4_ [M + H]^+^: 417.1809, found: 417.1820.

*Diethyl 3-methyl-5,7,12,13-tetrahydro-6H-cyclohepta[2,1-b:3,4-b’]diindole-6,6-dicarboxylate* (**2b**). Yellow solid, 101 mg (yield 78%). mp 203–205 °C. ^1^H-NMR (400 MHz, Methanol-*d*_4_) δ 7.52 (d, *J* = 7.8 Hz, 1H, Ar-H), 7.33 (d, *J* = 8.0 Hz, 1H, Ar-H), 7.31 (s, 1H, Ar-H), 7.23 (d, *J* = 8.2 Hz, 1H, Ar-H), 7.12 (t, *J* = 7.5 Hz, 1H, Ar-H), 7.05 (t, *J* = 7.4 Hz, 1H, Ar-H), 6.97 (d, *J* = 8.0 Hz, 1H, Ar-H), 4.00 (q, *J* = 7.1 Hz, 4H, -CO_2_C*H*_2_CH_3_), 3.61 (s, 2H, C_q_-CH_2_Ar), 3.59 (s, 2H, C_q_-CH_2_Ar), 2.44 (s, 3H, Ar-CH_3_), 0.98 (t, *J* = 7.1 Hz, 6H, -CO_2_CH_2_C*H*_3_). ^13^C-NMR (150 MHz, Methanol-*d*_4_) δ 172.83 (C=O), 137.67 (C), 136.06 (C), 130.76 (C), 130.54 (C), 129.57 (C), 128.85 (C), 128.78 (C), 124.69 (CH), 123.00 (CH), 120.39 (CH), 118.75 (CH), 118.49 (CH), 111.67 (CH), 111.44 (CH), 110.04 (C), 109.86 (C), 62.45 (OCH_2_), 56.56 (C), 32.31 (CH_2_), 32.25 (CH_2_), 21.70 (ArCH_3_), 14.10 (CH_3_). HRMS (ESI): *m/z* calcd for C_26_H_27_N_2_O_4_ [M + H]^+^: 431.1965, found: 431.1976.

*Diethyl 2-methyl-5,7,12,13-tetrahydro-6H-cyclohepta[2,1-b:3,4-b’]diindole-6,6-dicarboxylate* (**2c**). Yellow solid, 107 mg (yield 83%). mp 174–176 °C. ^1^H-NMR (400 MHz, Methanol-*d*_4_) δ 7.51 (d, *J* = 7.7 Hz, 1H, Ar-H), 7.39 (d, *J* = 8.1 Hz, 1H, Ar-H), 7.33 (d, *J* = 7.9 Hz, 1H, Ar-H), 7.15 – 7.09 (m, 2H, Ar-H), 7.05 (t, *J* = 7.5 Hz, 1H, Ar-H), 6.90 (dd, *J* = 8.0, 1.1 Hz, 1H, Ar-H), 3.99 (q, *J* = 7.1 Hz, 4H, -CO_2_C*H*_2_CH_3_), 3.60 (s, 2H, C_q_-CH_2_Ar), 3.58 (s, 2H, C_q_-CH_2_Ar), 2.44 (s, 3H, Ar-CH_3_), 0.97 (t, *J* = 7.1 Hz, 6H, -CO_2_CH_2_C*H*_3_). ^13^C-NMR (125 MHz, Methanol-*d*_4_) δ 172.83 (C=O), 138.14 (C), 137.65 (C), 132.95 (C), 130.57 (C), 128.92 (C), 128.53 (C), 128.06 (C), 122.91 (CH), 122.16 (CH), 120.38 (CH), 118.70 (CH), 118.56 (CH), 111.65 (2CH), 110.27 (C), 109.81 (C), 62.44 (OCH_2_), 56.54 (C), 32.38 (CH_2_), 32.26 (CH_2_), 21.88 (ArCH_3_), 14.09 (CH_3_). HRMS (ESI): *m/z* calcd for C_26_H_27_N_2_O_4_ [M + H]^+^: 431.1965, found: 431.1971.

*Diethyl 2,10-dimethyl-5,7,12,13-tetrahydro-6H-cyclohepta[2,1-b:3,4-b’]diindole-6,6-di carboxylate* (**2d**), 112 mg (yield 84%). Yellow solid. mp 212–214 °C. ^1^H-NMR (400 MHz, DMSO-*d*_6_) δ 10.72 (s, 2H, NH), 7.39 (d, *J* = 8.0 Hz, 2H, Ar-H), 7.19 (s, 2H, Ar-H), 6.87 (d, *J* = 8.1 Hz, 2H, Ar-H), 3.94 (q, *J* = 7.1 Hz, 4H, -CO_2_C*H*_2_CH_3_), 3.50 (s, 4H, C_q_-CH_2_Ar), 2.42 (s, 6H, Ar-CH_3_), 0.89 (t, *J* = 7.1 Hz, 6H, -CO_2_CH_2_C*H*_3_). ^13^C-NMR (125 MHz, DMSO-*d*_6_) δ 170.43 (C=O), 136.04 (C), 130.76 (C), 126.83 (C), 126.76 (C), 120.81 (CH), 117.40 (CH), 110.71 (CH), 108.11 (C), 60.75 (OCH_2_), 54.31 (C), 30.79 (CH_2_), 21.44 (ArCH_3_), 13.57 (CH_3_). HRMS (ESI): *m/z* calcd for C_27_H_29_N_2_O_4_ [M + H]^+^: 445.2122, found: 445.2131.

*Diethyl 3-fluoro-5,7,12,13-tetrahydro-6H-cyclohepta[2,1-b:3,4-b’]diindole-6,6-dicarboxylate* (**2e**). Yellow solid, 87 mg (yield 67%). mp 202–204 °C. ^1^H-NMR (400 MHz, Methanol-*d*_4_) δ 7.53 (d, *J* = 7.8 Hz, 1H, Ar-H), 7.35 (d, *J* = 8.0 Hz, 1H, Ar-H), 7.30 (dd, *J* = 8.8, 4.4 Hz, 1H, Ar-H), 7.18 (dd, *J* = 9.8, 2.5 Hz, 1H, Ar-H), 7.16–7.12 (m, 1H, Ar-H), 7.07 (ddd, *J* = 8.0, 7.0, 1.1 Hz, 1H, Ar-H), 6.89 (td, *J* = 9.1, 2.5 Hz, 1H, Ar-H), 4.00 (q, *J* = 7.1 Hz, 4H, -CO_2_C*H*_2_CH_3_), 3.62 (s, 2H, C_q_-CH_2_Ar), 3.56 (s, 2H, C_q_-CH_2_Ar), 0.98 (t, *J* = 7.1 Hz, 6H, -CO_2_CH_2_C*H*_3_). ^13^C-NMR (125 MHz, Methanol-*d*_4_) δ 172.68 (C=O), 159.38 (d, *J* = 233.3 Hz, CF), 137.80 (C), 134.23 (C), 130.96 (d, *J* = 9.6 Hz, C), 130.77 (C), 130.46 (C), 128.28 (C), 123.35 (CH), 120.51 (CH), 118.94 (CH), 112.51 (d, *J* = 9.8 Hz, CH), 111.80 (CH), 110.90 (d, *J* = 26.5 Hz, CH), 110.90 (C), 110.35 (d, *J* = 5.0 Hz, C), 103.49 (d, *J* = 23.9 Hz, CH), 62.50 (OCH_2_), 56.56 (C), 32.26 (CH_2_), 32.20 (CH_2_), 14.09 (CH_3_). HRMS (ESI): *m/z* calcd for C_25_H_24_FN_2_O_4_ [M + H]^+^: 435.1715, found: 435.1715.

*Diethyl 2-fluoro-5,7,12,13-tetrahydro-6H-cyclohepta[2,1-b:3,4-b’]diindole-6,6-dicarboxylate* (**2f**). Yellow solid, 104 mg (yield 80%). mp 219–221 °C. ^1^H-NMR (400 MHz, Methanol-*d*_4_) δ 7.52 (d, *J* = 7.9 Hz, 1H, Ar-H), 7.48 (dd, *J* = 8.7, 5.2 Hz, 1H, Ar-H), 7.34 (d, *J* = 8.0 Hz, 1H, Ar-H), 7.13 (ddd, *J* = 8.1, 7.0, 1.2 Hz, 1H, Ar-H), 7.09–7.03 (m, 2H, Ar-H), 6.85 (ddd, *J* = 9.7, 8.7, 2.3 Hz, 1H, Ar-H), 4.00 (q, *J* = 7.1 Hz, 4H, -CO_2_C*H*_2_CH_3_), 3.61 (s, 2H, C_q_-CH_2_Ar), 3.59 (s, 2H, C_q_-CH_2_Ar), 0.98 (t, *J* = 7.1 Hz, 6H, -CO_2_CH_2_C*H*_3_). ^13^C-NMR (125 MHz, Methanol-*d*_4_) δ 172.70 (C=O), 161.54 (d, *J* = 236.2 Hz, CF), 137.74 (C), 137.68 (d, *J* = 14.0 Hz, C), 130.49 (C), 129.27 (d, *J* = 3.5 Hz, C), 128.48 (C), 127.30 (C), 123.13 (CH), 120.48 (CH), 119.72 (d, *J* = 10.3 Hz, CH), 118.82 (CH), 111.76 (CH), 110.34 (C), 110.14 (C), 108.75 (d, *J* = 24.9 Hz, CH), 97.88 (d, *J* = 26.2 Hz, CH), 62.49 (OCH_2_), 56.51 (C), 32.26 (CH_2_), 32.17 (CH_2_), 14.09 (CH_3_). HRMS (ESI): *m/z* calcd for C_25_H_24_FN_2_O_4_ [M + H]^+^: 435.1715, found: 435.1723.

*Diethyl 2,10-difluoro-5,7,12,13-tetrahydro-6H-cyclohepta[2,1-b:3,4-b’]diindole-6,6-dicarboxylate* (**2g**). Yellow solid, 107 mg (yield 79%). mp 254–256 °C. ^1^H-NMR (400 MHz, DMSO-*d*_6_) δ 11.05 (s, 2H, NH), 7.53 (dd, *J* = 8.7, 5.4 Hz, 2H, Ar-H), 7.25 (dd, *J* = 9.9, 2.4 Hz, 2H, Ar-H), 6.89 (ddd, *J* = 9.9, 8.7, 2.4 Hz, 2H, Ar-H), 3.94 (q, *J* = 7.1 Hz, 4H, -CO_2_C*H*_2_CH_3_), 3.52 (s, 4H, C_q_-CH_2_Ar), 0.88 (t, *J* = 7.1 Hz, 6H, -CO_2_CH_2_C*H*_3_). ^13^C-NMR (125 MHz, DMSO-*d*_6_) δ 170.30 (C=O), 159.28 (d, *J* = 235.2 Hz, CF), 135.60 (d, *J* = 12.8 Hz, C), 127.58 (d, *J* = 3.5 Hz, C), 125.63 (C), 118.89 (d, *J* = 10.4 Hz, CH), 108.61 (C), 107.56 (d, *J* = 24.5 Hz, CH), 97.27 (d, *J* = 25.9 Hz, CH), 60.86 (OCH_2_), 54.24 (C), 30.64 (CH_2_), 13.56 (CH_3_). HRMS (ESI): *m/z* calcd for C_25_H_23_F_2_N_2_O_4_ [M + H]^+^: 453.1620, found: 453.1629.

*Diethyl 3-chloro-5,7,12,13-tetrahydro-6H-cyclohepta[2,1-b:3,4-b’]diindole-6,6-dicarboxylate* (**2h**). Yellow solid, 84 mg (yield 62%). mp 229–231 °C. ^1^H-NMR (400 MHz, Methanol-*d*_4_) δ 7.54 (d, *J* = 7.8 Hz, 1H, Ar-H), 7.49 (d, *J* = 2.0 Hz, 1H, Ar-H), 7.35 (d, *J* = 8.0 Hz, 1H, Ar-H), 7.31 (d, *J* = 8.5 Hz, 1H, Ar-H), 7.15 (t, *J* = 7.5 Hz, 1H, Ar-H), 7.10–7.04 (m, 2H, Ar-H), 4.00 (q, *J* = 7.1 Hz, 4H, -CO_2_C*H*_2_CH_3_), 3.62 (s, 2H, C_q_-CH_2_Ar), 3.56 (s, 2H, C_q_-CH_2_Ar), 0.98 (t, *J* = 7.1 Hz, 6H, -CO_2_CH_2_C*H*_3_). ^13^C-NMR (150 MHz, Methanol-*d*_4_) δ 172.61 (C=O), 137.82 (C), 136.05 (C), 131.64 (C), 130.47 (C), 130.42 (C), 128.07 (C), 126.19 (C), 123.43 (CH), 123.00 (CH), 120.54 (CH), 118.98 (CH), 118.18 (CH), 112.93 (CH), 111.83 (CH), 111.10 (C), 109.83 (C), 62.52 (OCH_2_), 56.48 (C), 32.21 (CH_2_), 32.05 (CH_2_), 14.10 (CH_3_). HRMS (ESI): *m/z* calcd for C_25_H_24_ClN_2_O_4_ [M + H]^+^: 451.1419, found: 451.1426.

*Diethyl 2-chloro-5,7,12,13-tetrahydro-6H-cyclohepta[2,1-b:3,4-b’]diindole-6,6-dicarboxylate* (**2i**). Yellow solid, 103 mg (yield 76%). mp 231–233 °C. ^1^H-NMR (400 MHz, DMSO-*d*_6_) δ 11.08 (s, 1H, NH), 10.99 (s, 1H, NH), 7.55 (d, *J* = 8.4 Hz, 1H, Ar-H), 7.54 (d, *J* = 7.7 Hz, 1H, Ar-H), 7.50 (d, *J* = 1.9 Hz, 1H, Ar-H), 7.41 (d, *J* = 8.0 Hz, 1H, Ar-H), 7.14 (ddd, *J* = 8.0, 6.9, 1.2 Hz, 1H, Ar-H), 7.08–7.01 (m, 2H, Ar-H), 3.94 (q, *J* = 7.0 Hz, 4H, -CO_2_C*H*_2_CH_3_), 3.54 (s, 2H, C_q_-CH_2_Ar), 3.53 (s, 2H, C_q_-CH_2_Ar), 0.88 (t, *J* = 7.1 Hz, 6H, -CO_2_CH_2_C*H*_3_). ^13^C-NMR (125 MHz, DMSO-*d*_6_) δ 170.32 (C=O), 136.06 (C), 135.77 (C), 128.69 (C), 128.32 (C), 127.60 (C), 126.70 (C), 126.27 (C), 122.02 (CH), 119.49 (CH), 119.22 (CH), 119.14 (CH), 117.91 (CH), 111.06 (CH), 110.66 (CH), 109.20 (C), 108.77 (C), 60.86 (OCH_2_), 54.28 (C), 30.73 (CH_2_), 30.56 (CH_2_), 13.57 (CH_3_). HRMS (ESI): *m/z* calcd for C_25_H_24_ClN_2_O_4_ [M + H]^+^: 451.1419, found: 451.1422.

*Diethyl 2,10-dichloro-5,7,12,13-tetrahydro-6H-cyclohepta[2,1-b:3,4-b’]diindole-6,6-di carboxylate* (**2j**). Yellow solid, 111 mg (yield 76%). mp 273–275 °C. ^1^H-NMR (300 MHz, DMSO-*d*_6_) δ 11.16 (s, 2H, NH), 7.56 (d, *J* = 8.5 Hz, 2H, Ar-H), 7.50 (d, *J* = 1.9 Hz, 2H, Ar-H), 7.05 (dd, *J* = 8.5, 1.9 Hz, 2H, Ar-H), 3.94 (q, *J* = 7.1 Hz, 4H, -CO_2_C*H*_2_CH_3_), 3.52 (s, 4H, C_q_-CH_2_Ar), 0.88 (t, *J* = 7.1 Hz, 6H, -CO_2_CH_2_C*H*_3_). ^13^C-NMR (150 MHz, DMSO-*d*_6_) δ 170.21 (C=O), 136.13 (C), 127.77 (C), 127.52 (C), 126.52 (C), 119.59 (CH), 119.28 (CH), 110.73 (CH), 109.33 (C), 60.91 (OCH_2_), 54.18 (C), 30.56 (CH_2_), 13.58 (CH_3_). HRMS (ESI): *m/z* calcd for C_25_H_21_Cl_2_N_2_O_4_ [M − H]^−^: 483.0884, found: 483.0880.

*Diethyl 3-bromo-5,7,12,13-tetrahydro-6H-cyclohepta[2,1-b:3,4-b’]diindole-6,6-dicarboxylate* (**2k**). Yellow solid, 92 mg (yield 62%). mp 253–255 °C. ^1^H-NMR (400 MHz, DMSO-*d*_6_) δ 11.14 (s, 1H, NH), 10.96 (s, 1H, NH), 7.73 (s, 1H, Ar-H), 7.55 (d, *J* = 7.9 Hz, 1H, Ar-H), 7.42 (d, *J* = 7.5 Hz, 1H, Ar-H), 7.39 (d, *J* = 8.6 Hz, 1H, Ar-H), 7.22 (d, *J* = 8.5 Hz, 1H, Ar-H), 7.14 (t, *J* = 7.7 Hz, 1H, Ar-H), 7.05 (t, *J* = 7.5 Hz, 1H, Ar-H), 3.94 (q, *J* = 7.1 Hz, 4H, -CO_2_C*H*_2_CH_3_), 3.55 (s, 2H, C_q_-CH_2_Ar), 3.51 (s, 2H, C_q_-CH_2_Ar), 0.89 (t, *J* = 7.1 Hz, 6H, -CO_2_CH_2_C*H*_3_). ^13^C-NMR (125 MHz, DMSO-*d*_6_) δ 170.30 (C=O), 135.80 (C), 134.38 (C), 130.60 (C), 128.79 (C), 128.65 (C), 126.59 (C), 124.02 (CH), 122.10 (CH), 120.11 (CH), 119.23 (CH), 117.95 (CH), 112.96 (CH), 111.74 (C), 111.08 (CH), 109.50 (C), 108.30 (C), 60.85 (OCH_2_), 54.33 (C), 30.69 (CH_2_), 30.54 (CH_2_), 13.54 (CH_3_). HRMS (ESI): *m/z* calcd for C_25_H_24_BrN_2_O_4_ [M + H]^+^: 495.0914, found: 495.0918.

*Diethyl 2-bromo-5,7,12,13-tetrahydro-6H-cyclohepta[2,1-b:3,4-b’]diindole-6,6-dicarboxylate* (**2l**). Yellow solid, 107 mg (yield 72%). mp 230–232 °C. ^1^H-NMR (400 MHz, DMSO-*d*_6_) δ 11.08 (s, 1H, NH), 11.00 (s, 1H, NH), 7.63 (d, *J* = 1.8 Hz, 1H, Ar-H), 7.54 (d, *J* = 7.9 Hz, 1H, Ar-H), 7.50 (d, *J* = 8.5 Hz, 1H, Ar-H), 7.41 (d, *J* = 8.1 Hz, 1H, Ar-H), 7.19–7.11 (m, 2H, Ar-H), 7.05 (t, *J* = 7.3 Hz, 1H, Ar-H), 3.94 (q, *J* = 7.1 Hz, 4H, -CO_2_C*H*_2_CH_3_), 3.54 (s, 2H, C_q_-CH_2_Ar), 3.52 (s, 2H, C_q_-CH_2_Ar), 0.88 (t, *J* = 7.1 Hz, 6H, -CO_2_CH_2_C*H*_3_). ^13^C-NMR (125 MHz, DMSO-*d*_6_) δ 170.31 (C=O), 136.52 (C), 135.78 (C), 128.70 (C), 128.23 (C), 127.83 (C), 126.64 (C), 122.05 (2CH), 119.53 (CH), 119.23 (CH), 117.92 (CH), 114.31 (C), 113.55 (CH), 111.07 (CH), 109.31 (C), 108.80 (C), 60.87 (OCH_2_), 54.27 (C), 30.73 (CH_2_), 30.52 (CH_2_), 13.57 (CH_3_). HRMS (ESI): *m/z* calcd for C_25_H_24_BrN_2_O_4_ [M + H]^+^: 495.0914, found: 495.0915.

*Diethyl 2,10-dibromo-5,7,12,13-tetrahydro-6H-cyclohepta[2,1-b:3,4-b’]diindole-6,6-di carboxylate* (**2m**). Yellow solid, 136 mg (yield 79%). mp 269–271 °C. ^1^H-NMR (400 MHz, DMSO-*d*_6_) δ 11.15 (s, 2H, NH), 7.64 (d, *J* = 1.8 Hz, 2H, Ar-H), 7.52 (d, *J* = 8.4 Hz, 2H, Ar-H), 7.17 (dd, *J* = 8.4, 1.8 Hz, 2H, Ar-H), 3.94 (q, *J* = 7.1 Hz, 4H, -CO_2_C*H*_2_CH_3_), 3.52 (s, 4H, C_q_-CH_2_Ar), 0.88 (t, *J* = 7.1 Hz, 6H, -CO_2_CH_2_C*H*_3_). ^13^C-NMR (150 MHz, DMSO-*d*_6_) δ 170.19 (C=O), 136.59 (C), 127.76 (C), 127.62 (C), 122.16 (CH), 119.66 (CH), 114.59 (C), 113.62 (CH), 109.47 (C), 60.90 (OCH_2_), 54.19 (C), 30.52 (CH_2_), 13.57 (CH_3_). HRMS (ESI): *m/z* calcd for C_25_H_21_Br_2_N_2_O_4_ [M − H]^−^: 570.9874, found: 570.9867.

*Diethyl 3-methoxy-5,7,12,13-tetrahydro-6H-cyclohepta[2,1-b:3,4-b’]diindole-6,6-dicarboxylate* (**2n**). Yellow solid, 90 mg (yield 67%). mp 192–194 °C. ^1^H-NMR (400 MHz, DMSO-*d*_6_) δ 10.89 (s, 1H, NH), 10.76 (s, 1H, NH), 7.52 (d, *J* = 7.8 Hz, 1H, Ar-H), 7.40 (d, *J* = 8.0 Hz, 1H, Ar-H), 7.30 (d, *J* = 8.7 Hz, 1H, Ar-H), 7.12 (t, *J* = 7.1 Hz, 1H, Ar-H), 7.06–6.99 (m, 2H, Ar-H), 6.75 (dd, *J* = 8.7, 2.4 Hz, 1H, Ar-H), 3.95 (q, *J* = 7.0 Hz, 4H, -CO_2_C*H*_2_CH_3_), 3.80 (s, 3H, -OCH_3_), 3.53 (s, 2H, C_q_-CH_2_Ar), 3.51 (s, 2H, C_q_-CH_2_Ar), 0.90 (t, *J* = 7.1 Hz, 6H, -CO_2_CH_2_C*H*_3_). ^13^C-NMR (125 MHz, DMSO-*d*_6_) δ 170.48 (C=O), 153.61 (C), 135.67 (C), 130.76 (C), 129.15 (C), 128.77 (C), 127.86 (C), 127.36 (C), 121.71 (CH), 119.09 (CH), 117.74 (CH), 112.03 (CH), 111.74 (CH), 110.93 (CH), 108.52 (C), 108.45 (C), 99.43 (CH), 60.81 (OCH_2_), 55.39 (C), 54.47 (OCH_3_), 30.84 (CH_2_), 30.75 (CH_2_), 13.58 (CH_3_). HRMS (ESI): *m/z* calcd for C_26_H_27_N_2_O_5_ [M + H]^+^: 447.1914, found: 447.1920.

*Diethyl 2-methoxy-5,7,12,13-tetrahydro-6H-cyclohepta[2,1-b:3,4-b’]diindole-6,6-dicarboxylate* (**2o**). Yellow solid, 76 mg (yield 57%). mp 118–120 °C. ^1^H-NMR (400 MHz, Methanol-*d*_4_) δ 7.50 (d, *J* = 7.8 Hz, 1H, Ar-H), 7.39 (d, *J* = 8.7 Hz, 1H, Ar-H), 7.32 (d, *J* = 7.9 Hz, 1H, Ar-H), 7.10 (t, *J* = 7.5 Hz, 1H, Ar-H), 7.04 (t, *J* = 7.4 Hz, 1H, Ar-H), 6.87 (d, *J* = 2.2 Hz, 1H, Ar-H), 6.73 (dd, *J* = 8.7, 2.2 Hz, 1H, Ar-H), 3.99 (q, *J* = 7.1 Hz, 4H, -CO_2_C*H*_2_CH_3_), 3.84 (s, 3H, -OCH_3_), 3.58 (s, 2H, C_q_-CH_2_Ar), 3.57 (s, 2H, C_q_-CH_2_Ar), 0.97 (t, *J* = 7.2 Hz, 6H, -CO_2_CH_2_C*H*_3_). ^13^C-NMR (150 MHz, Methanol-*d*_4_) δ 172.82 (C=O), 158.23 (C), 138.47 (C), 137.61 (C), 130.60 (C), 129.01 (C), 127.52 (C), 125.07 (C), 122.76 (CH), 120.35 (CH), 119.48 (CH), 118.61 (CH), 111.59 (CH), 110.44 (CH, C), 109.22 (C), 95.14 (CH), 62.44 (OCH_2_), 56.51 (C), 55.99 (OCH_3_), 32.38 (CH_2_), 32.20 (CH_2_), 14.10 (CH_3_). HRMS (ESI): *m/z* calcd for C_26_H_27_N_2_O_5_ [M + H]^+^: 447.1914, found: 447.1924.

*Diethyl 1-methoxy-5,7,12,13-tetrahydro-6H-cyclohepta[2,1-b:3,4-b’]diindole-6,6-dicarboxylate* (**2p**). Yellow solid, 83 mg (yield 62%). mp 224–226 °C. ^1^H-NMR (400 MHz, DMSO-*d*_6_) δ 11.09 (s, 1H, NH), 10.97 (s, 1H, NH), 7.53 (d, *J* = 7.8 Hz, 1H, Ar-H), 7.39 (d, *J* = 8.1 Hz, 1H, Ar-H), 7.16–7.09 (m, 2H, Ar-H), 7.03 (t, *J* = 7.4 Hz, 1H, Ar-H), 6.97 (t, *J* = 7.8 Hz, 1H, Ar-H), 6.71 (d, *J* = 7.7 Hz, 1H, Ar-H), 3.97 (s, 3H, -OCH_3_), 3.95 (q, *J* = 7.1 Hz, 4H, -CO_2_C*H*_2_CH_3_), 3.54 (s, 2H, C_q_-CH_2_Ar), 3.53 (s, 2H, C_q_-CH_2_Ar), 0.89 (t, *J* = 7.1 Hz, 6H, -CO_2_CH_2_C*H*_3_). ^13^C-NMR (125 MHz, DMSO-*d*_6_) δ 170.38 (C=O), 145.63 (C), 135.46 (C), 130.05 (C), 128.67 (C), 127.11 (C), 126.81 (C), 125.66 (C), 121.69 (CH), 119.73 (CH), 119.04 (CH), 117.73 (CH), 110.82 (CH), 110.68 (CH), 109.09 (C), 108.31 (C), 102.40 (CH), 60.79 (OCH_2_), 55.14 (C), 53.99 (OCH_3_), 31.00 (CH_2_), 30.82 (CH_2_), 13.55 (CH_3_). HRMS (ESI): *m/z* calcd for C_26_H_27_N_2_O_5_ [M + H]^+^: 447.1914, found: 447.1927.

*Diethyl 2,10-dimethoxy-5,7,12,13-tetrahydro-6H-cyclohepta[2,1-b:3,4-b’]diindole-6,6-di carboxylate* (**2q**). Yellow solid, 84 mg (yield 59%). mp 199–201 °C. ^1^H-NMR (400 MHz, Methanol-*d*_4_) δ 7.37 (d, *J* = 8.6 Hz, 2H, Ar-H), 6.86 (d, *J* = 2.2 Hz, 2H, Ar-H), 6.72 (dd, *J* = 8.6, 2.3 Hz, 2H, Ar-H), 3.99 (q, *J* = 7.1 Hz, 4H, -CO_2_C*H*_2_CH_3_), 3.83 (s, 6H, -OCH_3_), 3.54 (s, 4H, C_q_-CH_2_Ar), 0.98 (t, *J* = 7.1 Hz, 6H, -CO_2_CH_2_C*H*_3_). ^13^C-NMR (150 MHz, Methanol-*d*_4_) δ 172.85 (C=O), 158.01 (C), 138.37 (C), 127.86 (C), 125.16 (C), 119.28 (CH), 110.26 (CH), 109.37 (C), 95.19 (CH), 62.42 (OCH_2_), 56.52 (C), 56.01 (OCH_3_), 32.31 (CH_2_), 14.12 (CH_3_). HRMS (ESI): *m/z* calcd for C_27_H_29_N_2_O_6_ [M + H]^+^: 477.2020, found: 477.2025.

*Diethyl 3-(trifluoromethyl)-5,7,12,13-tetrahydro-6H-cyclohepta[2,1-b:3,4-b’]diindole-6,6-di carboxylate* (**2r**). Yellow solid, 80 mg (yield 55%). mp 263–265 °C. ^1^H-NMR (300 MHz, DMSO-*d*_6_) δ 11.41 (s, 1H, NH), 11.00 (s, 1H, NH), 7.94 (s, 1H, Ar-H), 7.61 (d, *J* = 8.4 Hz, 1H, Ar-H), 7.57 (d, *J* = 8.3 Hz, 1H, Ar-H), 7.47 –7.37 (m, 2H, Ar-H), 7.16 (t, *J* = 7.0 Hz, 1H, Ar-H), 7.06 (t, *J* = 7.5 Hz, 1H, Ar-H), 3.94 (q, *J* = 7.1 Hz, 4H, -CO_2_C*H*_2_CH_3_), 3.59 (s, 2H, C_q_-CH_2_Ar), 3.57 (s, 2H, C_q_-CH_2_Ar), 0.88 (t, *J* = 7.1 Hz, 6H, -CO_2_CH_2_C*H*_3_). ^13^C-NMR (125 MHz, DMSO-*d*_6_) δ 170.29 (C=O), 137.22 (C), 135.86 (C), 129.50 (C), 128.64 (C), 128.14 (C), 126.38 (C), 125.58 (q, *J* = 271.4 Hz, CF_3_), 122.26 (CH), 120.09 (q, *J* = 31.1 Hz, *C*CF_3_), 119.30 (CH), 118.04 (CH), 117.93 (d, *J* = 3.1 Hz, CH), 115.40 (d, *J* = 3.9 Hz, CH), 111.68 (CH), 111.16 (CH), 109.87 (C), 109.49 (C), 60.87 (OCH_2_), 54.34 (C), 30.75 (CH_2_), 30.43 (CH_2_), 13.51 (CH_3_). HRMS (ESI): *m/z* calcd for C_26_H_24_F_3_N_2_O_4_ [M + H]^+^: 485.1683, found: 485.1682.

*Ethyl 6-(methylsulfonyl)-6,7,12,13-tetrahydro-5H-cyclohepta[2,1-b:3,4-b’]diindole-6-carboxylate* (**2s**). Yellow solid, 82 mg (yield 65%). mp 227–229 °C. ^1^H-NMR (400 MHz, Methanol-*d*_4_) δ 7.61 (d, *J* = 7.8 Hz, 2H, Ar-H), 7.36 (d, *J* = 8.1 Hz, 2H, Ar-H), 7.16 (t, *J* = 7.5 Hz, 2H, Ar-H), 7.09 (t, *J* = 7.4 Hz, 2H, Ar-H), 4.17 (d, *J* = 14.7 Hz, 2H, C_q_-C*H*HAr), 3.76 (q, *J* = 7.1 Hz, 2H, -CO_2_C*H*_2_CH_3_), 3.48 (d, *J* = 14.7 Hz, 2H, C_q_-CH*H*Ar), 3.33 (s, 3H, -SO_2_CH_3_), 0.53 (t, *J* = 7.1 Hz, 3H, -CO_2_CH_2_C*H*_3_). ^13^C-NMR (125 MHz, DMSO-*d*_6_) δ 165.95 (C=O), 135.73 (C), 128.73 (C), 126.88 (C), 122.03 (CH), 119.27 (CH), 118.04 (CH), 111.03 (CH), 107.36 (C), 71.30 (C), 61.26 (OCH_2_), 37.23 (SO_2_CH_3_), 27.31 (CH_2_), 12.84 (CH_3_). HRMS (ESI): *m/z* calcd for C_23_H_23_N_2_O_4_S [M + H]^+^: 423.1373, found: 423.1373.

*Ethyl 6-cyano-6,7,12,13-tetrahydro-5H-cyclohepta[2,1-b:3,4-b’]diindole-6-carboxylate* (**2t**). Yellow solid, 45 mg (recycled raw material 39 mg, conversion yield 63%). mp 252–254 °C. ^1^H-NMR (300 MHz, DMSO-*d*_6_) δ 11.19 (s, 2H, NH), 7.60 (d, *J* = 7.9 Hz, 2H, Ar-H), 7.47 (d, *J* = 8.0 Hz, 2H, Ar-H), 7.17 (ddd, *J* = 8.1, 7.0, 1.2 Hz, 2H, Ar-H), 7.07 (ddd, *J* = 8.0, 7.0, 1.1 Hz, 2H, Ar-H), 4.32 (q, *J* = 7.1 Hz, 2H, -CO_2_C*H*_2_CH_3_), 3.87 (d, *J* = 15.9 Hz, 2H, C_q_-C*H*HAr), 3.54 (d, *J* = 16.0 Hz, 2H, C_q_-CH*H*Ar), 1.28 (t, *J* = 7.1 Hz, 3H, -CO_2_CH_2_C*H*_3_). ^13^C-NMR (150 MHz, DMSO-*d*_6_) δ 169.11 (C=O), 135.63 (C), 128.47 (C), 127.52 (C), 122.25 (CH), 119.50 (CH), 118.49 (CN), 117.98 (CH), 111.18 (CH), 106.70 (C), 62.79 (OCH_2_), 42.75 (C), 32.97 (CH_2_), 13.81 (CH_3_). HRMS (ESI): *m/z* calcd for C_23_H_20_N_3_O_2_ [M + H]^+^: 370.1550, found: 370.1553.

## 4. Conclusions

In summary, we have developed a novel oxidative cyclization/ aromatization of 1,3-di(1*H*-indol-3-yl)propanes to synthesize 6,7,12,13-tetrahydro-5*H*-cyclohepta[2,1-*b*:3,4-*b’*] diindoles. By employing PIFA/DDQ as coordinative oxidants in the presence of TMSCl as Lewis acid, the efficient intramolecular cyclization/aromatization generated the corresponding products with moderate to excellent yields. This protocol tolerated a broad range of functional-groups and the reaction could be carried out in air. Notably, no expensive transition metal catalysts/reagents were used for this transformation and this provided a new approach for these valuable compounds. Most importantly, the existing ester/CN groups in the target compounds could be transformed into other functional groups. Further biological evaluation of these compounds is in progress in our lab and the results will be reported soon.

## Figures and Tables

**Table 1:** Optimization of PIFA mediated oxidative cyclization/ aromatization.

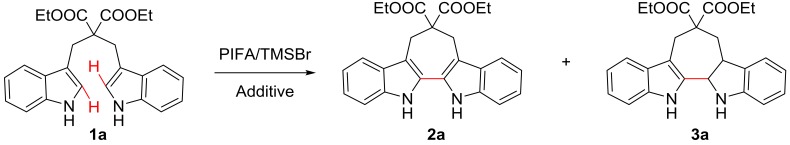			
Entry	Hypervalent iodine Reagent	Additive	Yield/% (2a/3a) ^b^
1	PIFA (1 eq)	None	55/trace
2	PIFA (0.5 eq)	None	38/19
3	PIFA (0.3 eq)	None	36/22
4 ^a^	PIFA (0.3 eq)	None	21/25
5	PIFA (0.3 eq)	DDQ (1 eq)	43/trace
6	PIFA (0.3 eq)	DDQ (0.7 eq)	56/trace
7	PIFA (0.3 eq)	DDQ (0.5 eq)	60/trace
8	PIFA (0.3 eq)	BQ (0.5 eq)	53/trace
9	PIFA (0.3 eq)	CAN (0.5 eq)	50/trace
10	None	DDQ (1 eq)	48/trace

Reagents and reaction conditions: **1a** (0.3 mmol, 1 eq), TMSBr (0.3 mmol, 1 eq), solvent (DCM, 2 mL), 0 °C for 4 h and then room temperature for 12 h. ^a^ The reaction was performed under Ar. ^b^ Isolated yields.

**Table 2:** Optimization of reaction conditions.

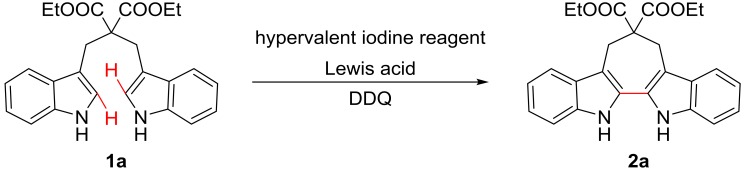				
Entry	Hypervalent Iodine Reagent	Lewis Acid	Temperature	Yield/% ^a^
1	PIFA	TMSBr	0 °C	60
2	PIFA	TMSCl	0 °C	61
3	PIFA	TMSOTf	0 °C	23
4	PIFA	BF_3_ OEt_2_	0 °C	49
5	PIFA	TMSCl	−40 °C	88
6	PIFA	TMSCl	−78 °C	70
7	PIDA	TMSCl	−40 °C	85
8	IBX	TMSCl	−40 °C	58
9	DMP	TMSCl	−40 °C	75

Reagents and reaction conditions: **1a** (0.3 mmol, 1 eq), hypervalent iodine reagent (0.1 mmol, 0.3 eq), DDQ (0.15 mmol, 0.5 eq), Lewis acid (0.3 mmol, 1 eq), solvent (DCM, 2 mL), Low temperature for 4 h and then room temperature for 12 h. ^a^ Isolated yields.

## Supplementary Materials

The NMR spectra of the obtained compounds and some additional experimental details are available online.
