# Supplementary material for: Convenient Synthesis of 6,7,12,13-Tetrahydro-5H-Cyclohepta[2,1-b:3,4-b’]diindole Derivatives Mediated by Hypervalent Iodine (III) Reagent
Source: Molecules. 2019 Mar 8;24(5):960. doi: 10.3390/molecules24050960 (PMC6429186; doi:10.3390/molecules24050960)
Supplement: Supplementary file 1 [file molecules-24-00960-s001.pdf]

Supporting Information

Convenient synthesis of 6,7,12,13-tetrahydro-5*H*-cyclohepta[2,1-*b*:3,4-*b'*]diindole derivatives mediated by hypervalent iodine (III) reagent

Lei Peng [1,2], Xiaofei Zhang [1,*] and Chunhao Yang [1,2,*]

[1] State Key Laboratory of Drug Research, Department of Medicinal Chemistry, Shanghai Institute of Materia Medica, Chinese Academy of Sciences, 555 Zu Chong Zhi Road, Shanghai 201203, China; leipeng@simm.ac.cn (L.P.)

[2] School of Pharmacy, University of Chinese Academy of Sciences, No.19A Yuquan Road, Beijing 100049, China

* Correspondence: chyang@simm.ac.cn (C.Y.); xiaofeizhang@simm.ac.cn (X.Z.); Tel.: +86-21-50806600-3512 (C.Y.); +86-21-50806600-3511 (X.Z.)

Contents

General information S2

General procedure for preparation of the substrates **1** S2

Experiment data for substrates **1** S3

Experiment data for compound **3a** S11

Copies of NMR spectra S12

Copies of HRMS chromatograms S53

General

All the chemical reagents were commercial products and used without purification in all cases. TLC was performed on silica gel plates (0.15-0.2 mm thickness, Yantai Huiyou Company, China) and detected with UV light at 254 nM, Column chromatography was carried out on silica gel (200-300 mesh). Proton and carbon magnetic resonance spectra (^{1}H NMR and ^{13}C NMR) were recorded on Varian Mercury-300, Varian Mercury-400, Varian Mercury-500 and/or Varian Mercury-600 spectrometers. NMR experiments were conducted in CD_3OD and DMSO-d_6. Tetramethylsilane (TMS) was used as internal standard. Chemical shifts (δ) are reported in parts per million (ppm). Data are reported as follows: chemical shift, multiplicity (br s = broad singlet, d = doublet, dd = doublet of doublet, dt = doublet of triplet, m = multiple, s = singlet and t = triplet), coupling constants (Hz). Low-resolution mass spectra (ESI) were obtained using Agilent HPLC-MS (1200-6110). High resolution mass spectra (HRMS) were obtained using Agilent 1290-6545 UHPLC-QTOF. Melting points (mp) were measured by Büchi 510 melting point apparatus without further corrected.

General procedure for preparation of the substrates (both symmetric and asymmetric ones) ***1***

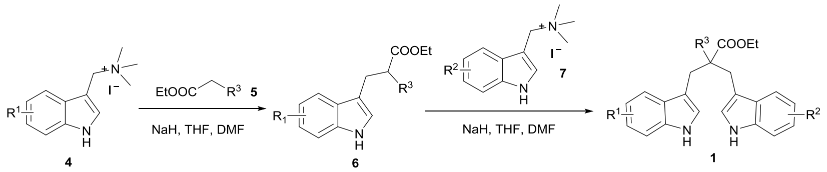

Ester **5** (3.3 mmol, 1.1 eq) was dissolved in tetrahydrofuran, sodium hydride (3.9 mmol, 1.3 eq) was added slowly at 0 ℃, and the mixture was moved to room temperature and stirred for 30 minutes, quaternary ammonium salt **4** (3 mmol, 1 eq) was dissolved in DMF and the solution was added to the reaction mixture, and stirred for 4 h at room temperature. After completion of the reaction, the resulting mixture was poured into water and extracted with ethyl acetate, the organic layer was washed with brine and dried over anhydrous sodium sulfate and concentrated in vacuum. The resulting residue was purified by silica gel chromatography (petroleum ether/ethyl acetate, v/v, 10:1 to 5:1) to afford **6**.

Ethyl R^3-substituted-3-(1*H*-indol-3-yl)propanoate **6** (1 mmol, 1 eq) was dissolved in tetrahydrofuran, sodium hydride (1.5 mmol, 1.5 eq) was added slowly at 0 ℃, and the mixture was

moved to room temperature and stirred for 30 minutes, quaternary ammonium salt **7** (1.5 mmol, 1.5 eq) was dissolved in DMF and the solution was added to the reaction mixture, and stirred for 4 h at room temperature. After completion of the reaction, the resulting mixture was poured into water and extracted with ethyl acetate, The organic layer was washed with brine and dried over anhydrous sodium sulfate and concentrated in vacuum. The resulting residue was purified by silica gel chromatography (petroleum ether/ethyl acetate, v/v, 8:1 to 4:1) to give 1,3-di(1*H*-indol-3-yl)propanes **1**.

*Experiment data for substrates **1***

Diethyl 2,2-bis((1*H*-indol-3-yl)methyl)malonate (1a)

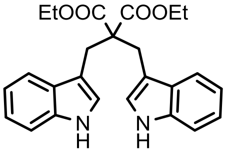

Yellow solid. (Yield of two steps, 82%). mp 120-122 ℃. ^{1}H NMR (400 MHz, DMSO-d_6) δ 10.97 (s, 2H, NH), 7.41 (d, *J* = 7.9 Hz, 2H, Ar-H), 7.35 (d, *J* = 8.1 Hz, 2H, Ar-H), 7.16 (d, *J* = 2.4 Hz, 2H, Ar-H), 7.06 (t, *J* = 7.5 Hz, 2H, Ar-H), 6.95 (t, *J* = 7.4 Hz, 2H, Ar-H), 3.90 (q, *J* = 7.1 Hz, 4H, -$CO_2CH_2CH_3$), 3.34 (s, 4H, C_q-CH_2Ar), 1.00 (t, *J* = 7.1 Hz, 6H, -$CO_2CH_2CH_3$). ^{13}C NMR (125 MHz, DMSO-d_6) δ 170.92 (C=O), 135.73 (C), 127.81 (C), 124.00 (CH), 120.86 (CH), 118.24 (CH), 118.20 (CH), 111.30 (CH), 108.19 (C), 60.62 (OCH_2), 58.67 (C), 28.12 (CH_2), 13.53 (CH_3). HRMS (ESI): *m/z* calcd for $C_{25}H_{27}N_2O_4$ $[M + H]^+$: 419.1965, found: 419.1973.

Diethyl 2-((1*H*-indol-3-yl)methyl)-2-((5-methyl-1*H*-indol-3-yl)methyl)malonate (1b)

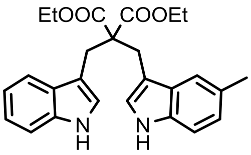

Yellow solid. (Yield of two steps, 84%). mp 132-134 ℃. ^{1}H NMR (400 MHz, DMSO-d_6) δ 10.97 (d, *J* = 2.7 Hz, 1H, NH), 10.83 (d, *J* = 2.6 Hz, 1H, NH), 7.40 (d, *J* = 7.9 Hz, 1H, Ar-H), 7.36 (d, *J* = 8.1 Hz, 1H, Ar-H), 7.22 (d, *J* = 8.2 Hz, 1H, Ar-H), 7.16 (d, *J* = 2.4 Hz, 1H, Ar-H), 7.14 – 7.09 (m, 2H, Ar-H), 7.06 (ddd, *J* = 8.1, 6.9, 1.2 Hz, 1H, Ar-H), 6.95 (ddd, *J* = 8.0, 6.9, 1.1 Hz, 1H, Ar-H), 6.87 (dd, *J* = 8.2, 1.6 Hz, 1H, Ar-H), 3.92 (q, *J* = 7.1 Hz, 4H, -$CO_2CH_2CH_3$), 3.32 (s, 4H, C_q-CH_2Ar), 2.29 (s, 3H, Ar-CH_3), 1.04 (t, *J* = 7.1 Hz, 6H, -$CO_2CH_2CH_3$). ^{13}C NMR (125 MHz, DMSO-d_6) δ 170.96 (C=O), 135.75 (C), 134.12 (C), 128.05 (C), 127.83 (C), 126.62 (C), 124.10 (CH), 123.85 (CH), 122.47 (CH), 120.90 (CH), 118.26 (CH), 118.22 (CH), 117.86 (CH), 111.30 (CH), 110.97 (CH), 108.27 (C), 107.60 (C), 60.65 (OCH_2), 58.54 (C), 27.96 (CH_2), 21.16 ($ArCH_3$), 13.57 (CH_3). HRMS (ESI): *m/z*

calcd for $C_{26}H_{29}N_2O_4$ $[M + H]^+$: 433.2122, found: 433.2130.

Diethyl 2-((1*H*-indol-3-yl)methyl)-2-((6-methyl-1*H*-indol-3-yl)methyl)malonate (1c)

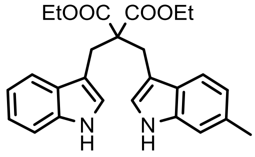

Yellow solid. (Yield of two steps, 87%). mp 119-121 ℃. ^{1}H NMR (400 MHz, DMSO-d_6) δ 10.97 (s, 1H, NH), 10.81 (s, 1H, NH), 7.41 (d, J = 8.0 Hz, 1H, Ar-H), 7.35 (d, J = 8.1 Hz, 1H, Ar-H), 7.29 (d, J = 8.1 Hz, 1H, Ar-H), 7.15 (d, J = 2.5 Hz, 1H, Ar-H), 7.13 (s, 1H, Ar-H), 7.09 – 7.03 (m, 2H, Ar-H), 6.98 – 6.91 (m, 1H, Ar-H), 6.78 (d, J = 8.0 Hz, 1H, Ar-H), 3.90 (q, J = 7.1 Hz, 4H, -$CO_2CH_2CH_3$), 3.33 (s, 2H, C_q-CH_2Ar), 3.31 (s, 2H, C_q-CH_2Ar), 2.37 (s, 3H, Ar-CH_3), 1.02 (t, J = 7.1 Hz, 6H, -$CO_2CH_2CH_3$). ^{13}C NMR (125 MHz, DMSO-d_6) δ 170.93 (C=O), 136.18 (C), 135.73 (C), 129.84 (C), 127.81 (C), 125.82 (C), 124.00 (CH), 123.25 (CH), 120.87 (CH), 120.05 (CH), 118.24 (CH), 118.21 (CH), 117.95 (CH), 111.30 (CH), 111.12 (CH), 108.22 (C), 108.01 (C), 60.61 (OCH_2), 58.64 (C), 28.16 (CH_2), 28.03 (CH_2), 21.32 ($ArCH_3$), 13.57 (CH_3). HRMS (ESI): *m/z* calcd for $C_{26}H_{29}N_2O_4$ $[M + H]^+$: 433.2122, found: 433.2134.

Diethyl 2,2-bis((6-methyl-1*H*-indol-3-yl)methyl)malonate (1d)

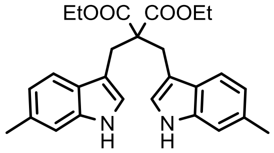

Yellow solid. (Yield of two steps, 80%). mp 147-149 ℃. ^{1}H NMR (400 MHz, DMSO-d_6) δ 10.79 (d, J = 2.5 Hz, 2H, NH), 7.28 (d, J = 8.1 Hz, 2H, Ar-H), 7.13 (s, 2H, Ar-H), 7.05 (d, J = 2.4 Hz, 2H, Ar-H), 6.78 (dd, J = 8.2, 1.5 Hz, 2H, Ar-H), 3.90 (q, J = 7.1 Hz, 4H, -$CO_2CH_2CH_3$), 3.29 (s, 4H, C_q-CH_2Ar), 2.37 (s, 6H, Ar-CH_3), 1.03 (t, J = 7.1 Hz, 6H, -$CO_2CH_2CH_3$). ^{13}C NMR (150 MHz, DMSO-d_6) δ 171.41 (C=O), 136.66 (C), 130.31 (C), 126.29 (C), 123.72 (CH), 120.52 (CH), 118.42 (CH), 111.59 (CH), 108.51 (C), 61.08 (OCH_2), 59.07 (C), 28.53 (CH_2), 21.81 ($ArCH_3$), 14.07 (CH_3). HRMS (ESI): *m/z* calcd for $C_{27}H_{31}N_2O_4$ $[M + H]^+$: 447.2278, found: 447.2290.

Diethyl 2-((1*H*-indol-3-yl)methyl)-2-((5-fluoro-1*H*-indol-3-yl)methyl)malonate (1e)

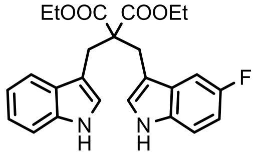

Yellow solid. (Yield of two steps, 83%). mp 119-121 ℃. ^{1}H NMR (400 MHz, DMSO-d_6) δ 11.09 (s, 1H, NH), 10.98 (s, 1H, NH), 7.41 (dd, J = 8.1, 4.1 Hz, 1H, Ar-H), 7.37 – 7.30 (m, 2H, Ar-H), 7.25 (d, J = 2.3 Hz, 1H, Ar-H), 7.18 (d, J = 2.1 Hz, 1H, Ar-H), 7.14 – 7.08 (m, 1H, Ar-H), 7.06 (t, J = 7.6 Hz, 1H, Ar-H), 6.95 (t, J = 7.5 Hz, 1H, Ar-H), 6.90 (td, J = 9.3, 2.5 Hz, 1H, Ar-H), 3.89 (q, J = 7.1 Hz, 4H, -$CO_2CH_2CH_3$), 3.33 (s, 2H, C_q-CH_2Ar), 3.29 (s, 2H, C_q-CH_2Ar), 1.00 (t, J = 7.1 Hz, 6H, -$CO_2CH_2CH_3$). ^{13}C NMR

(125 MHz, DMSO-d_6) δ 170.89 (C=O), 156.69 (d, *J* = 230.9 Hz, CF), 135.74 (C), 132.39 (C), 128.08 (d, *J* = 9.7 Hz, C), 127.80 (C), 126.30 (CH), 124.03 (CH), 120.88 (CH), 118.26 (CH), 118.22 (CH), 112.22 (d, *J* = 9.9 Hz, CH), 111.30 (CH), 108.99 (d, *J* = 26.2 Hz, CH), 108.55 (d, *J* = 5.2 Hz, C), 108.12 (C), 102.91 (d, *J* = 23.1 Hz, CH), 60.65 (OCH_2), 58.70 (C), 28.31 (CH_2), 28.23 (CH_2), 13.50 (CH_3). HRMS (ESI): *m/z* calcd for $C_{25}H_{25}FN_2NaO_4$ $[M + Na]^+$: 459.1691, found: 459.1703.

Diethyl 2-((1*H*-indol-3-yl)methyl)-2-((6-fluoro-1*H*-indol-3-yl)methyl)malonate (1f)

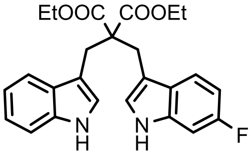

Yellow solid. (Yield of two steps, 81%). mp 124-126 ℃. ^1H NMR (400 MHz, DMSO-d_6) δ 11.04 (d, *J* = 2.5 Hz, 1H, NH), 10.97 (s, 1H, NH), 7.43 – 7.36 (m, 2H, Ar-H), 7.34 (d, *J* = 7.9 Hz, 1H, Ar-H), 7.18 (d, *J* = 2.4 Hz, 1H, Ar-H), 7.16 (d, *J* = 2.4 Hz, 1H, Ar-H), 7.12 (dd, *J* = 10.1, 2.4 Hz, 1H, Ar-H), 7.05 (ddd, *J* = 8.0, 6.8, 1.1 Hz, 1H, Ar-H), 6.94 (t, *J* = 7.5 Hz, 1H, Ar-H), 6.85 – 6.76 (m, 1H, Ar-H), 3.89 (q, *J* = 7.1 Hz, 4H, -$CO_2CH_2CH_3$), 3.33 (s, 2H, C_q-CH_2Ar), 3.31 (s, 2H, C_q-CH_2Ar), 1.00 (t, *J* = 7.1 Hz, 6H, -$CO_2CH_2CH_3$). ^{13}C NMR (125 MHz, DMSO-d_6) δ 170.87 (C=O), 158.68 (d, *J* = 234.0 Hz, CF), 135.73 (C), 135.51 (d, *J* = 12.7 Hz, C), 127.79 (C), 124.66 (CH), 124.63 (d, *J* = 3.1 Hz, C), 124.04 (CH), 120.87 (CH), 119.24 (d, *J* = 10.3 Hz, CH), 118.25 (CH), 118.20 (CH), 111.31 (CH), 108.51 (C), 108.11 (C), 106.73 (d, *J* = 24.4 Hz, CH), 97.18 (d, *J* = 25.2 Hz, CH), 60.65 (OCH_2), 58.66 (C), 28.21 (CH_2), 28.06 (CH_2), 13.53 (CH_3). HRMS (ESI): *m/z* calcd for $C_{25}H_{26}FN_2O_4$ $[M + H]^+$: 437.1871, found: 437.1878.

Diethyl 2,2-bis((6-fluoro-1*H*-indol-3-yl)methyl)malonate (1g)

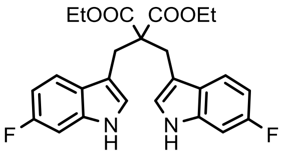

Yellow solid. (Yield of two steps, 80%). mp 119-121 ℃. ^1H NMR (600 MHz, DMSO-d_6) δ 11.01 (s, 2H, NH), 7.37 (dd, *J* = 8.7, 5.4 Hz, 2H, Ar-H), 7.16 (d, *J* = 2.4 Hz, 2H, Ar-H), 7.11 (dd, *J* = 10.1, 2.4 Hz, 2H, Ar-H), 6.80 (ddd, *J* = 9.8, 8.7, 2.4 Hz, 2H, Ar-H), 3.89 (q, *J* = 7.1 Hz, 4H, -$CO_2CH_2CH_3$), 3.31 (s, 4H, C_q-CH_2Ar), 0.99 (t, *J* = 7.1 Hz, 6H, -$CO_2CH_2CH_3$). ^{13}C NMR (125 MHz, DMSO-d_6) δ 170.85 (C=O), 158.71 (d, *J* = 233.8 Hz, CF), 135.54 (d, *J* = 12.7 Hz, C), 124.70 (d, *J* = 3.4 Hz, C), 124.67 (CH), 119.26 (d, *J* = 10.2 Hz, CH), 108.44 (C), 106.79 (d, *J* = 24.5 Hz, CH), 97.22 (d, *J* = 25.3 Hz, CH), 60.72 (OCH_2), 58.65 (C), 28.16 (CH_2), 13.55 (CH_3). HRMS (ESI): *m/z* calcd for $C_{25}H_{25}F_2N_2O_4$ $[M + H]^+$: 455.1777, found: 455.1782.

Diethyl 2-((1*H*-indol-3-yl)methyl)-2-((5-chloro-1*H*-indol-3-yl)methyl)malonate (1h)

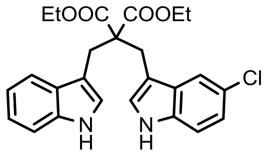

Yellow solid. (Yield of two steps, 82%). mp 122-124 ℃. ^{1}H NMR (400 MHz, DMSO-d_6) δ 11.18 (s, 1H, NH), 10.99 (s, 1H, NH), 7.43 (d, *J* = 8.0 Hz, 1H, Ar-H), 7.38 (d, *J* = 2.0 Hz, 1H, Ar-H), 7.36 (d, *J* = 8.7 Hz, 1H, Ar-H), 7.35 (d, *J* = 8.0 Hz, 1H, Ar-H), 7.27 (d, *J* = 2.5 Hz, 1H, Ar-H), 7.19 (d, *J* = 2.4 Hz, 1H, Ar-H), 7.09 – 7.02 (m, 2H, Ar-H), 6.95 (t, *J* = 7.4 Hz, 1H, Ar-H), 3.89 (q, *J* = 7.1 Hz, 4H, -$CO_2CH_2CH_3$), 3.32 (s, 2H, C_q-CH_2Ar), 3.30 (s, 2H, C_q-CH_2Ar), 1.01 (t, *J* = 7.1 Hz, 6H, -$CO_2CH_2CH_3$). ^{13}C NMR (125 MHz, DMSO-d_6) δ 170.84 (C=O), 135.75 (C), 134.18 (C), 128.90 (C), 127.78 (C), 126.20 (CH), 124.08 (CH), 123.09 (C), 120.89 (CH), 120.76 (CH), 118.28 (CH), 118.23 (CH), 117.57 (CH), 112.84 (CH), 111.31 (CH), 108.18 (C), 108.05 (C), 60.68 (OCH_2), 58.65 (C), 28.34 (CH_2), 28.06 (CH_2), 13.50 (CH_3). HRMS (ESI): *m/z* calcd for $C_{25}H_{26}ClN_2O_4$ $[M + H]^+$: 453.1576, found: 453.1582.

Diethyl 2-((1*H*-indol-3-yl)methyl)-2-((6-chloro-1*H*-indol-3-yl)methyl)malonate (1i)

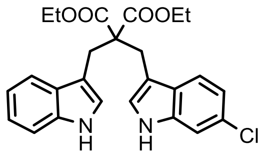

Yellow solid. (Yield of two steps, 80%). mp 129-131 ℃. ^{1}H NMR (400 MHz, DMSO-d_6) δ 11.11 (s, 1H, NH), 10.98 (s, 1H, NH), 7.44 – 7.37 (m, 3H, Ar-H), 7.35 (d, *J* = 8.1 Hz, 1H, Ar-H), 7.23 (d, *J* = 2.4 Hz, 1H, Ar-H), 7.16 (d, *J* = 2.4 Hz, 1H, Ar-H), 7.06 (ddd, *J* = 8.1, 6.9, 1.2 Hz, 1H, Ar-H), 6.99 – 6.91 (m, 2H, Ar-H), 3.89 (q, *J* = 7.1 Hz, 4H, -$CO_2CH_2CH_3$), 3.33 (s, 2H, C_q-CH_2Ar), 3.31 (s, 2H, C_q-CH_2Ar), 1.00 (t, *J* = 7.1 Hz, 6H, -$CO_2CH_2CH_3$). ^{13}C NMR (125 MHz, DMSO-d_6) δ 170.85 (C=O), 136.08 (C), 135.75 (C), 127.79 (C), 126.64 (C), 125.60 (C), 125.27 (CH), 124.08 (CH), 120.89 (CH), 119.65 (CH), 118.61 (CH), 118.28 (CH), 118.20 (CH), 111.33 (CH), 110.91 (CH), 108.63 (C), 108.07 (C), 60.68 (OCH_2), 58.68 (C), 28.27 (CH_2), 28.03 (CH_2), 13.54 (CH_3). HRMS (ESI): *m/z* calcd for $C_{25}H_{26}ClN_2O_4$ $[M + H]^+$: 453.1576, found: 453.1577.

Diethyl 2,2-bis((6-chloro-1*H*-indol-3-yl)methyl)malonate (1j)

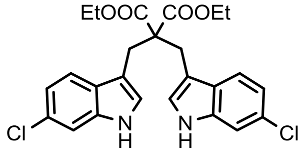

Yellow solid. (Yield of two steps, 81%). mp 172-174 ℃. ^{1}H NMR (600 MHz, DMSO-d_6) δ 11.10 (d, *J* = 2.5 Hz, 2H, NH), 7.42 – 7.37 (m, 4H, Ar-H), 7.21 (d, *J* = 2.4 Hz, 2H, Ar-H), 6.96 (dd, *J* = 8.4, 2.0 Hz, 2H, Ar-H), 3.89 (q, *J* = 7.1 Hz, 4H, -$CO_2CH_2CH_3$), 3.31 (s, 4H, C_q-CH_2Ar), 0.99 (t, *J* = 7.1 Hz, 6H, -$CO_2CH_2CH_3$). ^{13}C NMR (125 MHz, DMSO-d_6) δ 170.78 (C=O), 136.10 (C), 126.63 (C), 125.63 (C), 125.37 (CH), 119.67 (CH), 118.65 (CH), 110.95 (CH), 108.51 (C), 60.75 (OCH_2), 58.67

(C), 28.16 (CH_2), 13.55 (CH_3). HRMS (ESI): *m/z* calcd for $C_{25}H_{23}Cl_2N_2O_4$ [M - H]$^-$: 485.1040, found: 485.1028.

Diethyl 2-((1*H*-indol-3-yl)methyl)-2-((5-bromo-1*H*-indol-3-yl)methyl)malonate (1k)

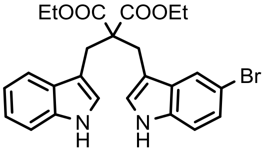

Yellow solid. (Yield of two steps, 82%). mp 124-126 ℃. ^{1}H NMR (400 MHz, DMSO-d_6) δ 11.20 (s, 1H, NH), 10.99 (s, 1H, NH), 7.53 (d, *J* = 2.0 Hz, 1H, Ar-H), 7.43 (d, *J* = 8.0 Hz, 1H, Ar-H), 7.35 (d, *J* = 8.2 Hz, 1H, Ar-H), 7.32 (d, *J* = 8.6 Hz, 1H, Ar-H), 7.26 (d, *J* = 2.5 Hz, 1H, Ar-H), 7.20 (d, *J* = 2.4 Hz, 1H, Ar-H), 7.16 (dd, *J* = 8.6, 1.9 Hz, 1H, Ar-H), 7.06 (t, *J* = 7.6 Hz, 1H, Ar-H), 6.96 (t, *J* = 7.4 Hz, 1H, Ar-H), 3.89 (q, *J* = 7.1 Hz, 4H, -$CO_2C\mathit{H}_2CH_3$), 3.32 (s, 2H, C_q-CH_2Ar), 3.30 (s, 2H, C_q-CH_2Ar), 1.02 (t, *J* = 7.1 Hz, 6H, -$CO_2CH_2C\mathit{H}_3$). ^{13}C NMR (125 MHz, DMSO-d_6) δ 170.83 (C=O), 135.76 (C), 134.41 (C), 129.59 (C), 127.78 (C), 126.08 (CH), 124.09 (CH), 123.27 (CH), 120.89 (CH), 120.61 (CH), 118.29 (CH), 118.23 (CH), 113.32 (CH), 111.32 (CH), 111.03 (C), 108.08 (C), 108.04 (C), 60.69 (OCH_2), 58.61 (C), 28.33 (CH_2), 28.04 (CH_2), 13.52 (CH_3). HRMS (ESI): *m/z* calcd for $C_{25}H_{26}BrN_2O_4$ [M + H]$^+$: 497.1070, found: 497.1072.

Diethyl 2-((1*H*-indol-3-yl)methyl)-2-((6-bromo-1*H*-indol-3-yl)methyl)malonate (1l)

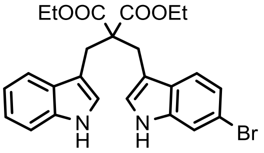

Yellow solid. (Yield of two steps, 81%). mp 132-134 ℃. ^{1}H NMR (600 MHz, DMSO-d_6) δ 11.10 (s, 1H, NH), 10.95 (s, 1H, NH), 7.53 (d, *J* = 1.8 Hz, 1H, Ar-H), 7.41 (d, *J* = 7.4 Hz, 1H, Ar-H), 7.36 (d, *J* = 2.4 Hz, 1H, Ar-H), 7.34 (d, *J* = 2.0 Hz, 1H, Ar-H), 7.21 (d, *J* = 2.4 Hz, 1H, Ar-H), 7.15 (d, *J* = 2.4 Hz, 1H, Ar-H), 7.09 – 7.03 (m, 2H, Ar-H), 6.95 (ddd, *J* = 8.0, 6.9, 1.0 Hz, 1H, Ar-H), 3.90 (q, *J* = 7.1 Hz, 4H, -$CO_2C\mathit{H}_2CH_3$), 3.33 (s, 2H, C_q-CH_2Ar), 3.31 (s, 2H, C_q-CH_2Ar), 1.00 (t, *J* = 7.1 Hz, 6H, -$CO_2CH_2C\mathit{H}_3$). ^{13}C NMR (125 MHz, DMSO-d_6) δ 170.82 (C=O), 136.56 (C), 135.72 (C), 127.76 (C), 126.85 (C), 125.21 (CH), 124.08 (CH), 121.13 (CH), 120.88 (CH), 120.05 (CH), 118.26 (CH), 118.19 (CH), 113.85 (CH), 113.60 (C), 111.31 (CH), 108.62 (C), 108.03 (C), 60.67 (OCH_2), 58.64 (C), 28.23 (CH_2), 27.96 (CH_2), 13.52 (CH_3). HRMS (ESI): *m/z* calcd for $C_{25}H_{26}BrN_2O_4$ [M + H]$^+$: 497.1070, found: 497.1077.

Diethyl 2,2-bis((6-bromo-1*H*-indol-3-yl)methyl)malonate (1m)

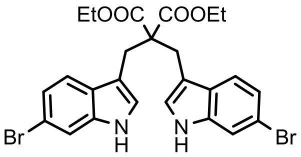

Yellow solid. (Yield of two steps, 81%). mp 183-185 ℃. ^{1}H NMR (400 MHz, DMSO-d_6) δ 11.13 (d, *J* = 2.6 Hz, 2H, NH), 7.53 (d, *J* = 1.8 Hz, 2H, Ar-H), 7.35 (d, *J* = 8.5 Hz, 2H, Ar-H), 7.22 (d, *J* = 2.5 Hz, 2H, Ar-H), 7.07 (dd, *J* = 8.5, 1.9 Hz, 2H, Ar-H), 3.89 (q, *J* = 7.1 Hz, 4H, -$CO_2CH_2CH_3$), 3.30 (s, 4H, C_q-CH_2Ar), 0.99 (t, *J* = 7.1 Hz, 6H, -$CO_2CH_2CH_3$). ^{13}C NMR (125 MHz, DMSO-d_6) δ 170.73 (C=O), 136.59 (C), 126.84 (C), 125.28 (CH), 121.16 (CH), 120.05 (CH), 113.88 (CH), 113.63 (C), 108.53 (C), 60.73 (OCH_2), 58.65 (C), 28.14 (CH_2), 13.53 (CH_3). HRMS (ESI): *m/z* calcd for $C_{25}H_{23}Br_2N_2O_4$ $[M - H]^-$: 573.0030, found: 573.0013.

Diethyl 2-((1*H*-indol-3-yl)methyl)-2-((5-methoxy-1*H*-indol-3-yl)methyl)malonate (1n)

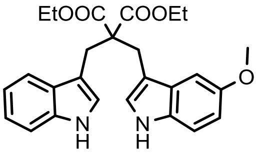

Yellow solid. (Yield of two steps, 81%). mp 95-97 ℃. ^{1}H NMR (400 MHz, Methanol-d_4) δ 7.42 (d, *J* = 8.0 Hz, 1H, Ar-H), 7.32 (d, *J* = 8.1 Hz, 1H, Ar-H), 7.20 (d, *J* = 8.8 Hz, 1H, Ar-H), 7.11 (s, 1H, Ar-H), 7.08 (ddd, *J* = 8.1, 7.0, 1.2 Hz, 1H, Ar-H), 7.03 (s, 1H, Ar-H), 6.96 (ddd, *J* = 8.0, 7.0, 1.0 Hz, 1H, Ar-H), 6.83 (d, *J* = 2.4 Hz, 1H, Ar-H), 6.71 (dd, *J* = 8.7, 2.4 Hz, 1H, Ar-H), 3.98 (q, *J* = 7.2 Hz, 4H, -$CO_2CH_2CH_3$), 3.57 (s, 3H, -OCH_3), 3.46 (s, 2H, C_q-CH_2Ar), 3.45 (s, 2H, C_q-CH_2Ar), 1.05 (t, *J* = 7.1 Hz, 6H, -$CO_2CH_2CH_3$). ^{13}C NMR (150 MHz, Methanol-d_4) δ 173.52 (C=O), 154.87 (C), 137.60 (C), 132.78 (C), 129.88 (C), 129.54 (C), 125.43 (CH), 124.37 (CH), 122.37 (CH), 119.59 (CH), 119.50 (CH), 112.77 (CH), 112.70 (CH), 112.09 (CH), 110.41 (C), 110.12 (C), 101.34 (CH), 62.36 (OCH_2), 60.84 (C), 55.98 (OCH_3), 29.79 (CH_2), 29.69 (CH_2), 14.08 (CH_3). HRMS (ESI): *m/z* calcd for $C_{26}H_{29}N_2O_5$ $[M + H]^+$: 449.2071, found: 449.2082.

Diethyl 2-((1*H*-indol-3-yl)methyl)-2-((6-methoxy-1*H*-indol-3-yl)methyl)malonate (1o)

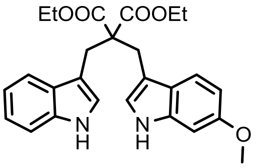

Yellow solid. (Yield of two steps, 84%). mp 114-116 ℃. ^{1}H NMR (400 MHz, DMSO-d_6) δ 10.96 (d, *J* = 2.4 Hz, 1H, NH), 10.76 (s, 1H, NH), 7.40 (d, *J* = 7.9 Hz, 1H, Ar-H), 7.34 (d, *J* = 8.1 Hz, 1H, Ar-H), 7.26 (d, *J* = 8.7 Hz, 1H, Ar-H), 7.14 (d, *J* = 2.4 Hz, 1H, Ar-H), 7.08 – 7.03 (m, 1H, Ar-H), 7.02 (d, *J* = 2.3 Hz, 1H, Ar-H), 6.97 – 6.91 (m, 1H, Ar-H), 6.84 (d, *J* = 2.3 Hz, 1H, Ar-H), 6.60 (dd, *J* = 8.7, 2.3 Hz, 1H, Ar-H), 3.90 (q, *J* = 7.1 Hz, 4H, -$CO_2CH_2CH_3$), 3.75 (s, 3H, -OCH_3), 3.32 (s, 2H, C_q-CH_2Ar), 3.28 (s, 2H, C_q-CH_2Ar), 1.02 (t, *J* = 7.1 Hz, 6H, -$CO_2CH_2CH_3$). ^{13}C NMR (125 MHz, DMSO-d_6) δ 170.94 (C=O), 155.39 (C), 136.43 (C), 135.74 (C), 127.82 (C), 124.00 (CH), 122.55 (CH), 122.22 (C),

120.88 (CH), 118.84 (CH), 118.25 (CH), 118.21 (CH), 111.31 (CH), 108.56 (CH), 108.21 (C), 108.18 (C), 94.31 (CH), 60.63 (OCH_2), 58.64 (C), 55.11 (OCH_3), 28.15 (CH_2), 28.02 (CH_2), 13.58 (CH_3). HRMS (ESI): *m/z* calcd for $C_{26}H_{29}N_2O_5$ $[M + H]^+$: 449.2071, found: 449.2084.

Diethyl 2-((1*H*-indol-3-yl)methyl)-2-((7-methoxy-1*H*-indol-3-yl)methyl)malonate (1p)

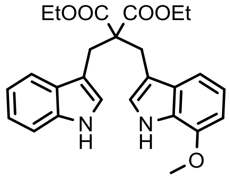

Yellow solid. (Yield of two steps, 78%). mp 159-161 ℃. ^{1}H NMR (400 MHz, DMSO-d_6) δ 11.08 (d, *J* = 2.5 Hz, 1H, NH), 10.96 (s, 1H, NH), 7.40 (d, *J* = 7.9 Hz, 1H, Ar-H), 7.34 (d, *J* = 8.1 Hz, 1H, Ar-H), 7.14 (d, *J* = 2.4 Hz, 1H, Ar-H), 7.08 – 7.03 (m, 2H, Ar-H), 7.01 (d, *J* = 8.1 Hz, 1H, Ar-H), 6.97 – 6.91 (m, 1H, Ar-H), 6.87 (t, *J* = 7.8 Hz, 1H, Ar-H), 6.63 (d, *J* = 7.6 Hz, 1H, Ar-H), 3.90 (q, *J* = 7.1 Hz, 4H, -$CO_2CH_2CH_3$), 3.90 (s, 3H, -OCH_3), 3.32 (s, 2H, C_q-CH_2Ar), 3.31 (s, 2H, C_q-CH_2Ar), 1.01 (t, *J* = 7.1 Hz, 6H, -$CO_2CH_2CH_3$). ^{13}C NMR (125 MHz, DMSO-d_6) δ 170.92 (C=O), 146.03 (C), 135.73 (C), 129.38 (C), 127.81 (C), 125.82 (C), 123.98 (CH), 123.48 (CH), 120.88 (CH), 118.84 (CH), 118.26 (CH), 118.19 (CH), 111.31 (CH), 111.13 (CH), 108.76 (C), 108.19 (C), 101.41 (CH), 60.64 (OCH_2), 58.66 (C), 55.03 (OCH_3), 28.29 (CH_2), 28.07 (CH_2), 13.56 (CH_3). HRMS (ESI): *m/z* calcd for $C_{26}H_{29}N_2O_5$ $[M + H]^+$: 449.2071, found: 449.2082.

Diethyl 2,2-bis((6-methoxy-1*H*-indol-3-yl)methyl)malonate (1q)

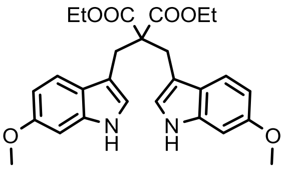

Yellow solid. (Yield of two steps, 80%). mp 129-131 ℃. ^{1}H NMR (400 MHz, DMSO-d_6) δ 10.75 (d, *J* = 2.5 Hz, 2H, NH), 7.25 (d, *J* = 8.7 Hz, 2H, Ar-H), 7.00 (d, *J* = 2.4 Hz, 2H, Ar-H), 6.84 (d, *J* = 2.3 Hz, 2H, Ar-H), 6.60 (dd, *J* = 8.7, 2.3 Hz, 2H, Ar-H), 3.91 (q, *J* = 7.0 Hz, 4H, -$CO_2CH_2CH_3$), 3.74 (s, 6H, -OCH_3), 3.27 (s, 4H, C_q-CH_2Ar), 1.03 (t, *J* = 7.1 Hz, 6H, -$CO_2CH_2CH_3$). ^{13}C NMR (150 MHz, DMSO-d_6) δ 170.92 (C=O), 155.38 (C), 136.42 (C), 122.53 (CH), 122.21 (C), 118.82 (CH), 108.54 (CH), 108.18 (C), 94.29 (CH), 60.61 (OCH_2), 58.59 (C), 55.10 (OCH_3), 28.03 (CH_2), 13.59 (CH_3). HRMS (ESI): *m/z* calcd for $C_{27}H_{31}N_2O_6$ $[M + H]^+$: 479.2177, found: 479.2189.

Diethyl 2-((1*H*-indol-3-yl)methyl)-2-((5-(trifluoromethyl)-1*H*-indol-3-yl)methyl)malonate (1r)

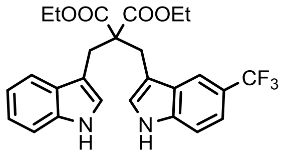

Yellow solid. (Yield of two steps, 84%). mp 127-129 ℃. ^{1}H NMR (400 MHz, Methanol-d_4) δ 7.75 (d, *J* = 1.7 Hz, 1H, Ar-H), 7.50 – 7.43 (m, 2H, Ar-H), 7.36 – 7.30 (m, 2H, Ar-H), 7.25 (s, 1H, Ar-H), 7.12 (s, 1H, Ar-H), 7.08 (t, *J* = 7.4 Hz, 1H, Ar-H), 6.97 (td, *J* = 7.4, 7.0, 1.0 Hz, 1H, Ar-H), 3.93 (q, *J* = 7.2 Hz,

4H, $-CO_2CH_2CH_3$), 3.48 (s, 2H, C_q-CH_2Ar), 3.45 (s, 2H, C_q-CH_2Ar), 1.03 (t, *J* = 7.1 Hz, 6H, -$CO_2CH_2CH_3$). ^{13}C NMR (125 MHz, DMSO-d_6) δ 170.82 (C=O), 137.25 (C), 135.77 (C), 127.76 (C), 127.04 (C), 126.86 (CH), 125.64 (q, *J* = 271.1 Hz, CF_3), 124.10 (CH), 120.90 (CH), 119.24 (q, *J* = 30.9 Hz, *C*CF_3), 118.28 (CH), 118.21 (CH), 117.23 (d, *J* = 3.7 Hz, CH), 115.85 (d, *J* = 4.6 Hz, CH), 112.13 (CH), 111.32 (CH), 109.50 (C), 107.99 (C), 60.67 (OCH_2), 58.61 (C), 28.37 (CH_2), 27.99 (CH_2), 13.43 (CH_3). HRMS (ESI): *m/z* calcd for $C_{26}H_{26}F_3N_2O_4$ $[M + H]^+$: 487.1839, found: 487.1842.

Ethyl 2-((1*H*-indol-3-yl)methyl)-3-(1*H*-indol-3-yl)-2-(methylsulfonyl)propanoate (1s)

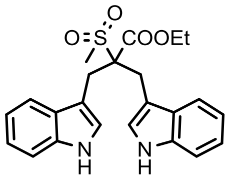

Yellow solid. (Yield of two steps, 78%). mp 194-196 ℃. ^{1}H NMR (400 MHz, DMSO-d_6) δ 11.00 (d, *J* = 2.6 Hz, 2H, NH), 7.54 (d, *J* = 7.9 Hz, 2H, Ar-H), 7.34 (d, *J* = 8.0 Hz, 2H, Ar-H), 7.14 (d, *J* = 2.5 Hz, 2H, Ar-H), 7.06 (ddd, *J* = 8.1, 6.9, 1.3 Hz, 2H, Ar-H), 6.98 (ddd, *J* = 7.4, 7.0, 1.2 Hz, 2H, Ar-H), 4.02 (q, *J* = 7.1 Hz, 2H, -$CO_2CH_2CH_3$), 3.64 (d, *J* = 15.0 Hz, 2H, C_q-C*H*HAr), 3.47 (d, *J* = 15.1 Hz, 2H, C_q-CH*H*Ar), 2.80 (s, 3H, - SO_2CH_3), 0.96 (t, *J* = 7.1 Hz, 3H, -$CO_2CH_2CH_3$). ^{13}C NMR (125 MHz, DMSO-d_6) δ 168.16 (C=O), 135.56 (C), 127.95 (C), 125.29 (CH), 120.91 (CH), 118.66 (CH), 118.46 (CH), 111.33 (CH), 107.06 (C), 75.68 (C), 61.78 (OCH_2), 40.29 (SO_2CH_3), 27.97 (CH_2), 13.29 (CH_3). HRMS (ESI): *m/z* calcd for $C_{23}H_{25}N_2O_4S$ $[M + H]^+$: 425.1530, found: 425.1541.

Ethyl 2-((1*H*-indol-3-yl)methyl)-2-cyano-3-(1*H*-indol-3-yl)propanoate (1t)

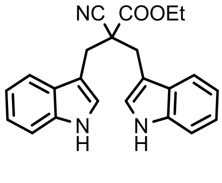

Yellow solid. (Yield of two steps, 85%). mp 100-102 ℃. ^{1}H NMR (400 MHz, DMSO-d_6) δ 11.06 (s, 2H, NH), 7.58 (d, *J* = 7.9 Hz, 2H, Ar-H), 7.36 (d, *J* = 8.1 Hz, 2H, Ar-H), 7.22 (d, *J* = 2.5 Hz, 2H, Ar-H), 7.08 (ddd, *J* = 8.2, 6.9, 1.2 Hz, 2H, Ar-H), 6.99 (ddd, *J* = 8.0, 6.9, 1.1 Hz, 2H, Ar-H), 3.89 (q, *J* = 7.1 Hz, 2H, -$CO_2CH_2CH_3$), 3.53 (d, *J* = 14.5 Hz, 2H, C_q-C*H*HAr), 3.44 (d, *J* = 14.5 Hz, 2H, C_q-CH*H*Ar), 0.86 (t, *J* = 7.1 Hz, 3H, -$CO_2CH_2CH_3$). ^{13}C NMR (125 MHz, DMSO-d_6) δ 168.84 (C=O), 135.79 (C), 127.19 (C), 124.62 (CH), 121.07 (CH), 120.07 (CN), 118.54 (CH), 118.50 (CH), 111.41 (CH), 107.71 (C), 62.06 (OCH_2), 53.90 (C), 32.87 (CH_2), 13.39 (CH_3). HRMS (ESI): *m/z* calcd for $C_{23}H_{22}N_3O_2$ $[M + H]^+$: 372.1707, found: 372.1717.

*Experiment data for compound **3a***

Diethyl 4b,5,7,12,12b,13-hexahydro-6*H*-cyclohepta[2,1-*b*:3,4-*b'*]diindole-6,6-dicarboxylate (3a)

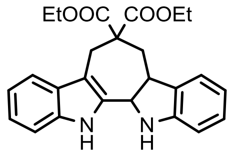

Yellow solid. mp 90-92 ℃. ^{1}H NMR (500 MHz, Methanol-d_4) δ 7.51 (d, J = 7.6 Hz, 1H, Ar-H), 7.29 (d, J = 7.9 Hz, 1H, Ar-H), 7.13 (d, J = 7.4 Hz, 1H, Ar-H), 7.06 – 7.01 (m, 2H, Ar-H), 6.99 (ddd, J = 8.0, 7.0, 1.2 Hz, 1H, Ar-H), 6.75 (td, J = 7.4, 1.0 Hz, 1H, Ar-H), 6.71 (d, J = 7.8 Hz, 1H, Ar-H), 5.14 (d, J = 8.5 Hz, 1H, -NHC*H*), 4.32 – 4.21 (m, 2H, -$CO_2C\mathit{H}_2CH_3$), 4.00 (q, J = 7.1 Hz, 2H, -$CO_2C\mathit{H}_2CH_3$), 3.84 (ddd, J = 11.1, 8.9, 1.6 Hz, 1H, -NHCHC*H*), 3.57 (d, J = 15.1 Hz, 1H, C_q-C*H*HAr), 3.31 – 3.27 (m, 1H, C_q-C*H*HAr), 2.27 (dd, J = 14.9, 11.4 Hz, 1H, C_q-CH*H*Ar), 2.13 (d, J = 14.8 Hz, 1H, C_q-CH*H*Ar), 1.29 (t, J = 7.1 Hz, 3H, -$CO_2CH_2C\mathit{H}_3$), 1.10 (t, J = 7.1 Hz, 3H, -$CO_2CH_2C\mathit{H}_3$). ^{13}C NMR (125 MHz, Methanol-d_4) δ 173.48 (C=O), 173.04 (C=O), 150.92 (C), 137.21 (C), 136.21 (C), 134.88 (C), 129.41 (C), 128.97 (CH), 125.34 (CH), 121.81 (CH), 120.76 (CH), 119.82 (CH), 119.12 (CH), 111.72 (CH), 111.30 (CH), 106.21 (C), 62.66 (OCH_2), 62.53 (OCH_2), 60.79 (NHCH), 57.84 (C), 41.70 (CH), 36.69 (CH_2), 27.53 (CH_2), 14.51 (CH_3), 14.27 (CH_3). HRMS (ESI): *m/z* calcd for $C_{25}H_{27}N_2O_4$ $[M + H]^+$: 419.1965, found: 419.1964.

Copies of NMR spectra

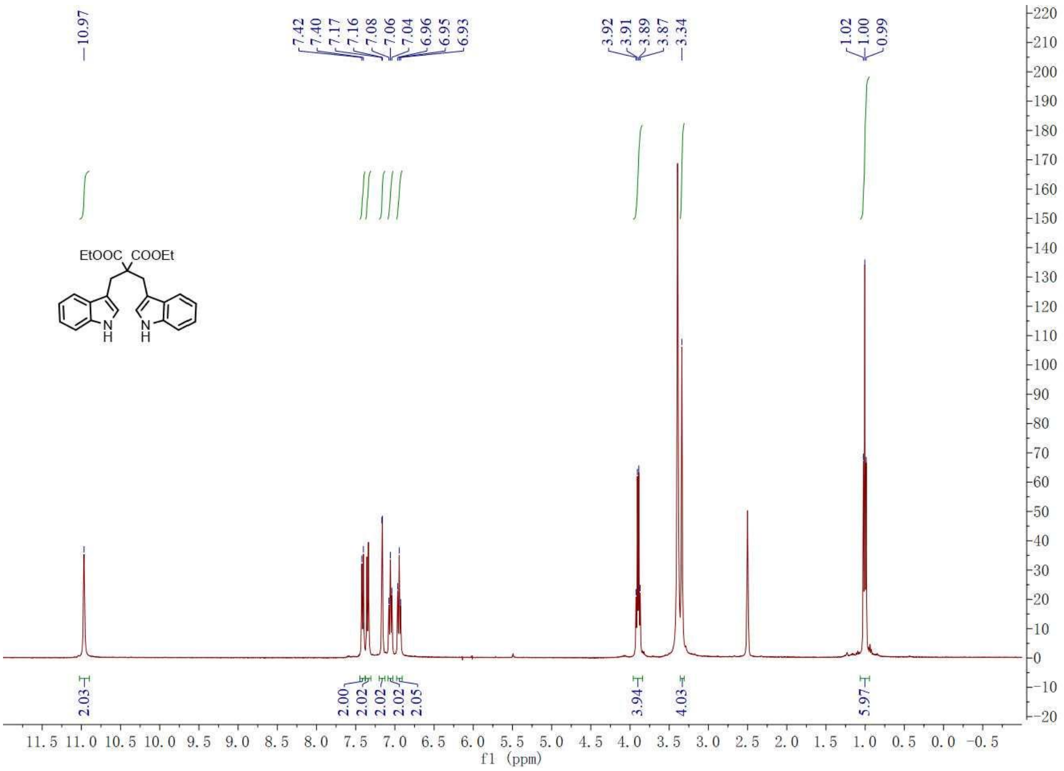

Figure S1. ^{1}H NMR spectrum of diethyl 2,2-bis((1*H*-indol-3-yl)methyl)malonate (**1a**) in DMSO-d_6

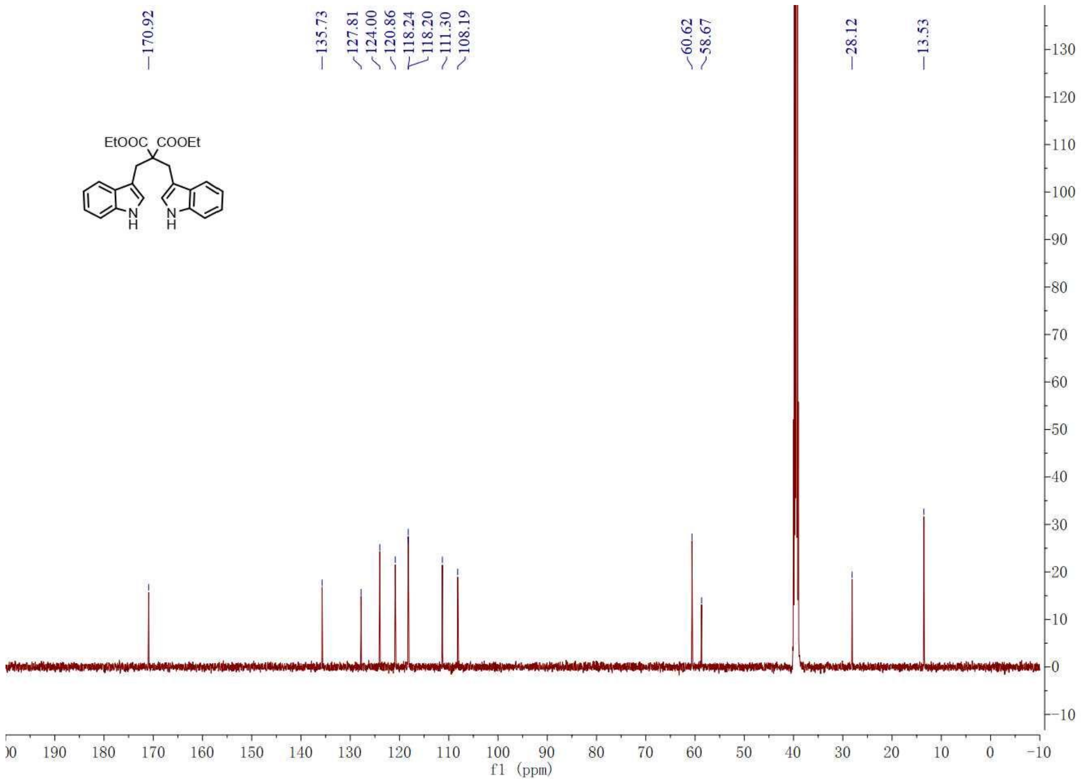

Figure S2. ^{13}C NMR spectrum of diethyl 2,2-bis((1*H*-indol-3-yl)methyl)malonate (**1a**) in DMSO-d_6

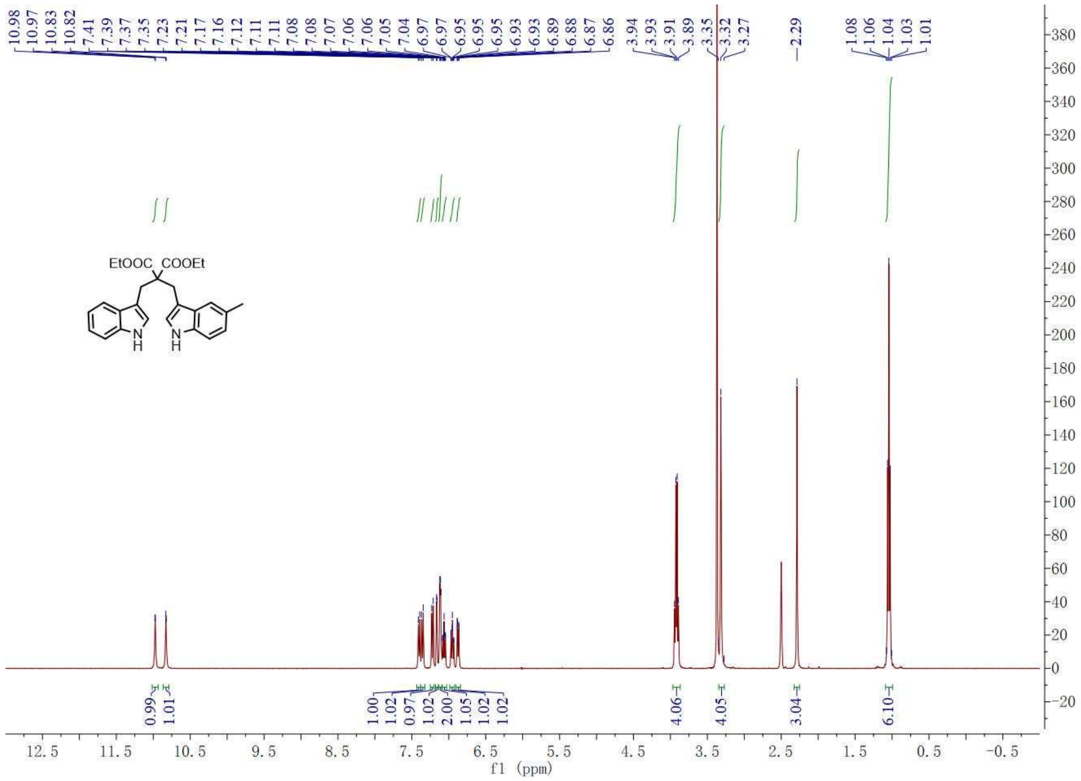

Figure S3. ^{1}H NMR spectrum of diethyl 2-((1*H*-indol-3-yl)methyl)-2-((5-methyl-1*H*-indol-3-yl)methyl)malonate (**1b**) in DMSO-d_6

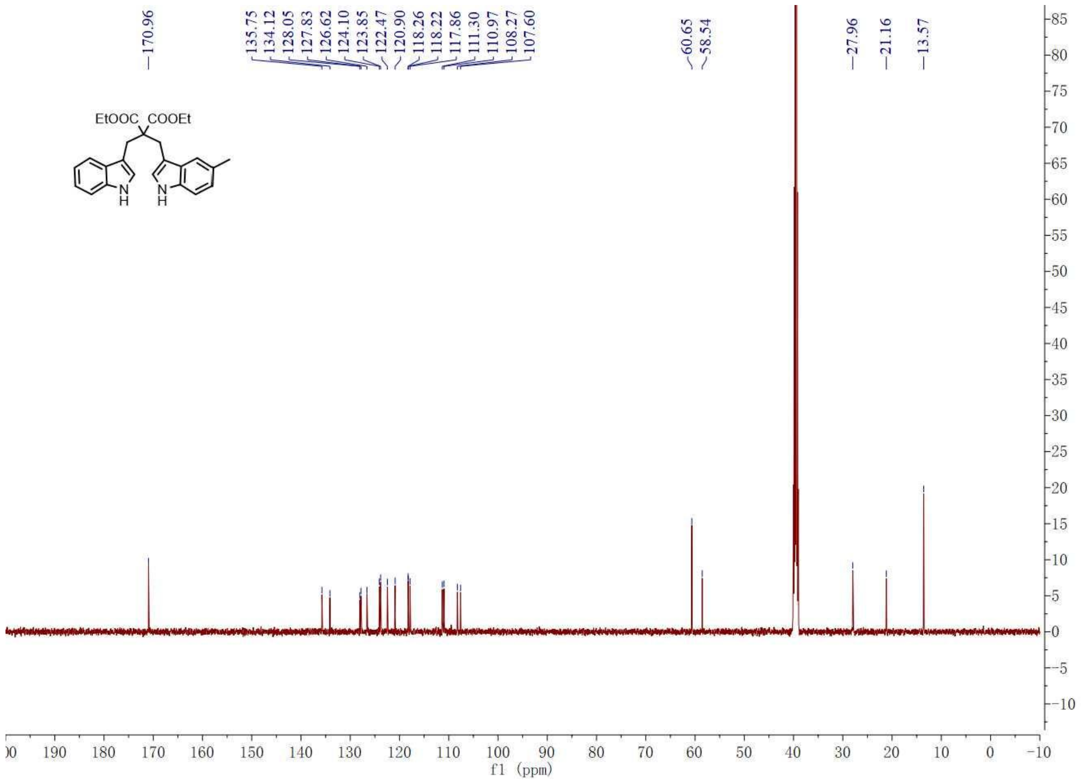

Figure S4. ^{13}C NMR spectrum of diethyl 2-((1*H*-indol-3-yl)methyl)-2-((5-methyl-1*H*-indol-3-yl)methyl)malonate (**1b**) in DMSO-d_6

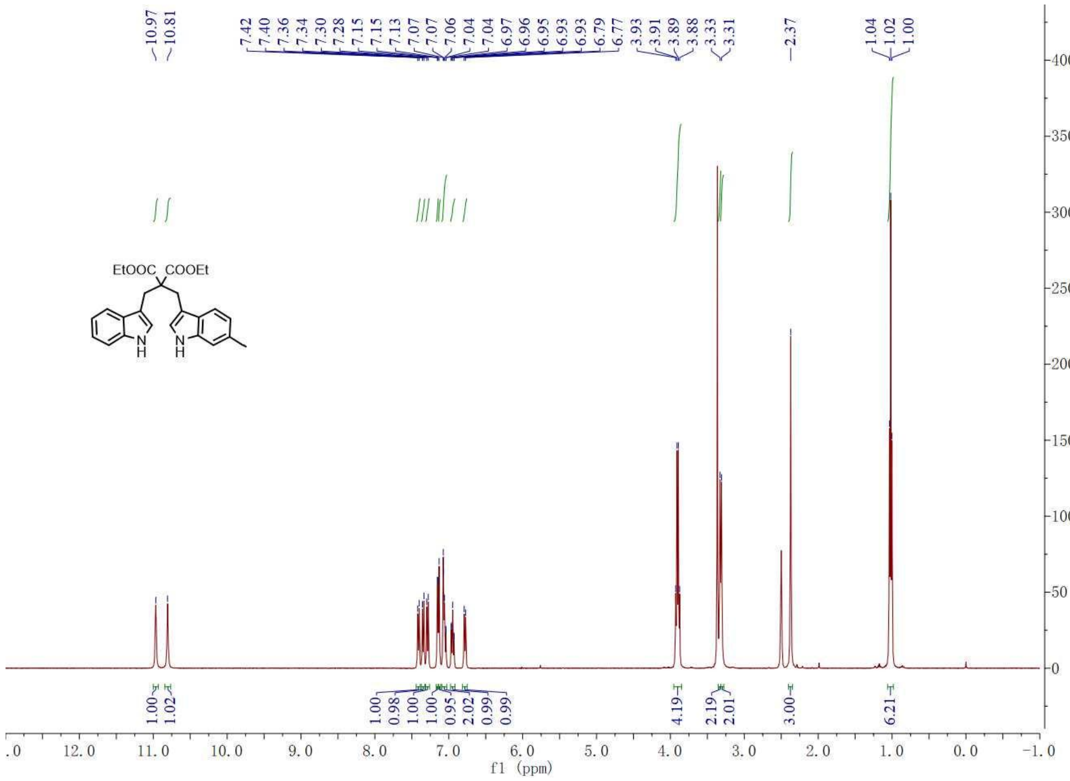

Figure S5. ^{1}H NMR spectrum of diethyl 2-((1*H*-indol-3-yl)methyl)-2-((6-methyl-1*H*-indol-3-yl)methyl)malonate (**1c**) in DMSO-d_6

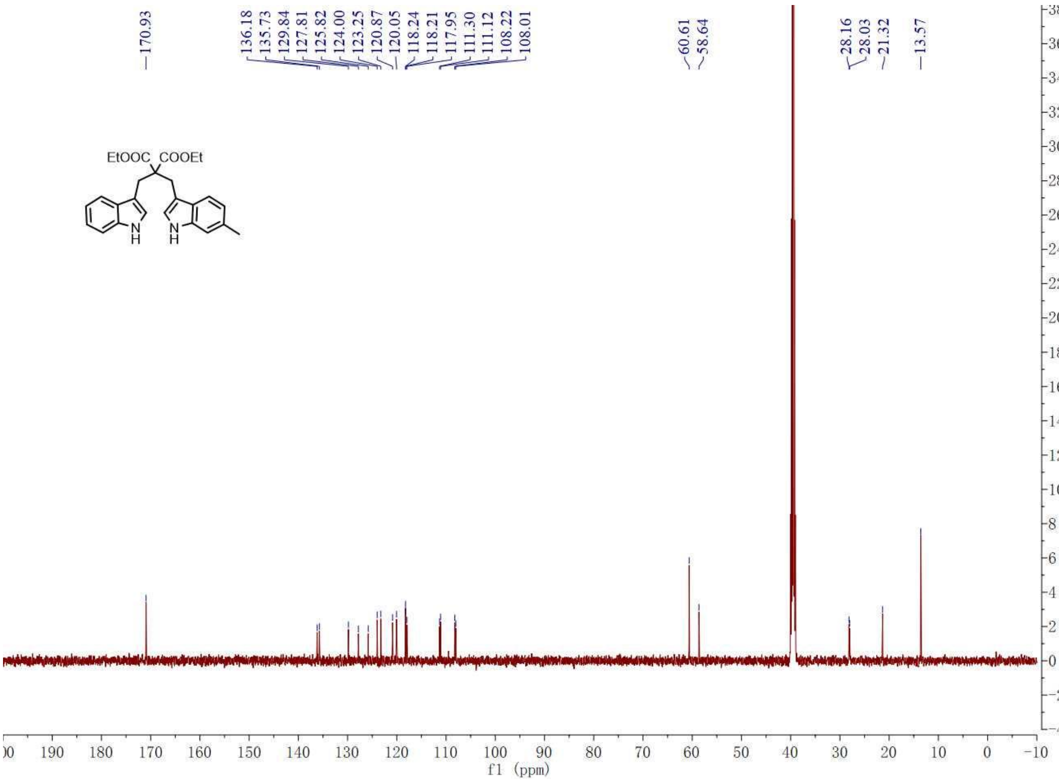

Figure S6. ^{13}C NMR spectrum of diethyl 2-((1*H*-indol-3-yl)methyl)-2-((6-methyl-1*H*-indol-3-yl)methyl)malonate (**1c**) in DMSO-d_6

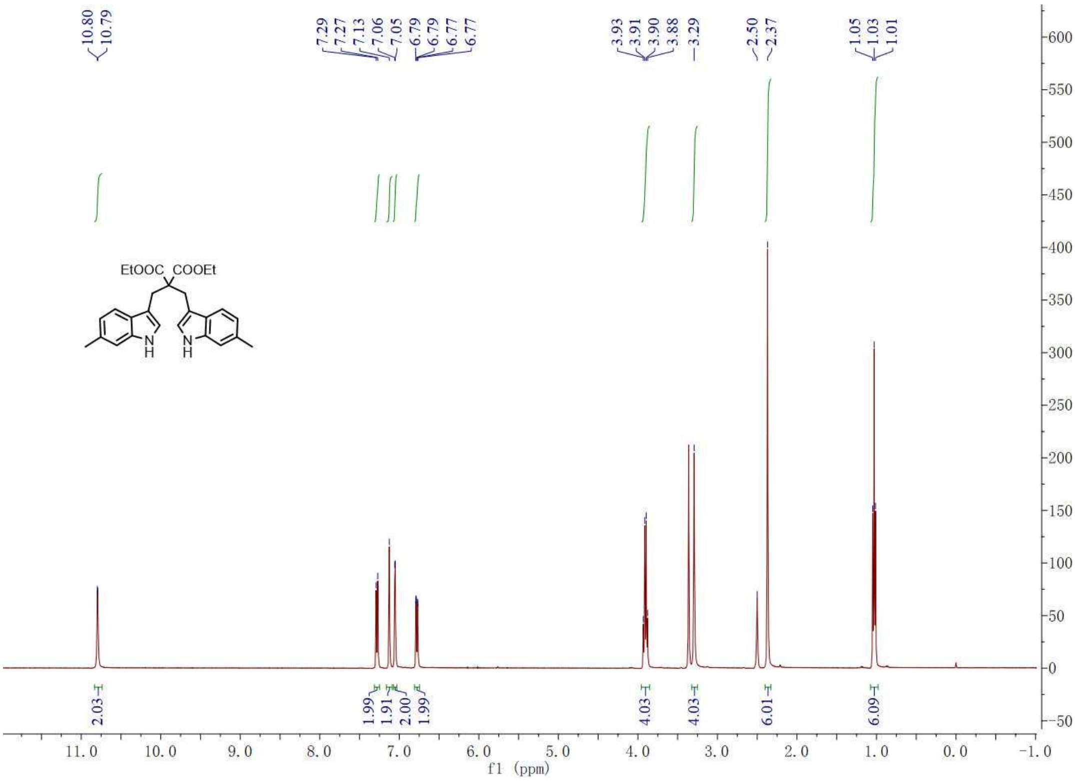

Figure S7. ^{1}H NMR spectrum of diethyl 2,2-bis((6-methyl-1*H*-indol-3-yl)methyl)malonate (**1d**) in DMSO-d_6

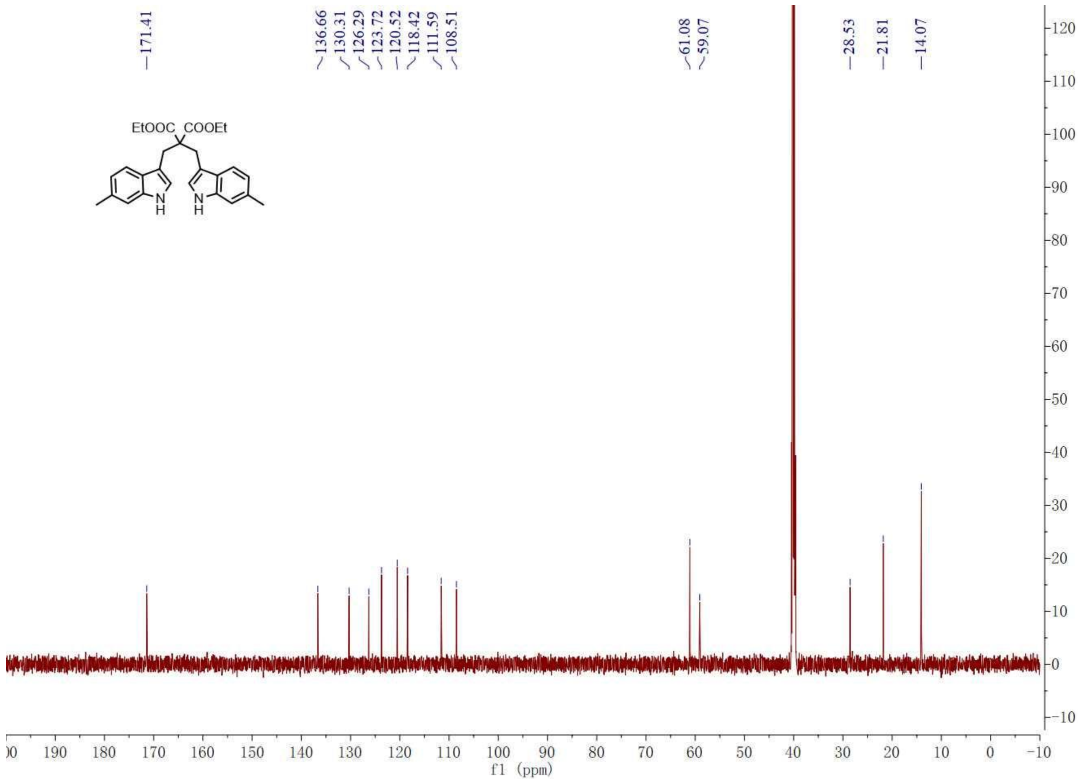

Figure S8. ^{13}C NMR spectrum of diethyl 2,2-bis((6-methyl-1*H*-indol-3-yl)methyl)malonate (**1d**) in DMSO-d_6

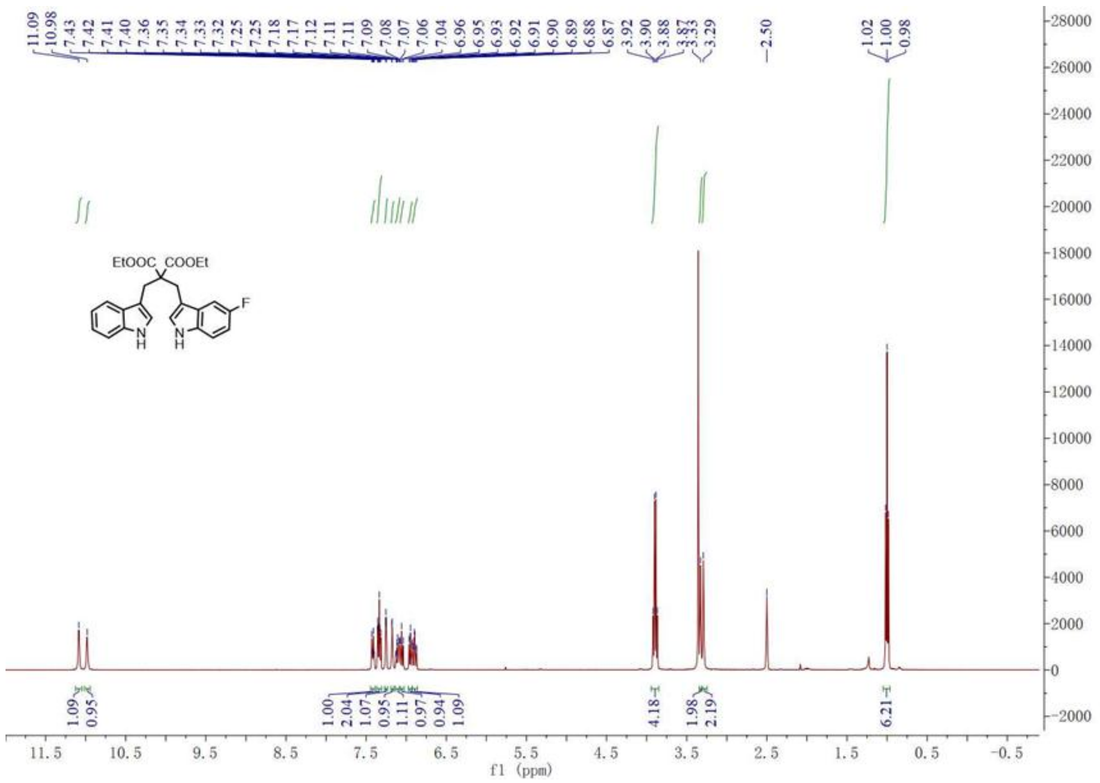

Figure S9. ^{1}H NMR spectrum of diethyl 2-((1*H*-indol-3-yl)methyl)-2-((5-fluoro-1*H*-indol-3-yl)methyl)malonate (**1e**) in DMSO-d_6

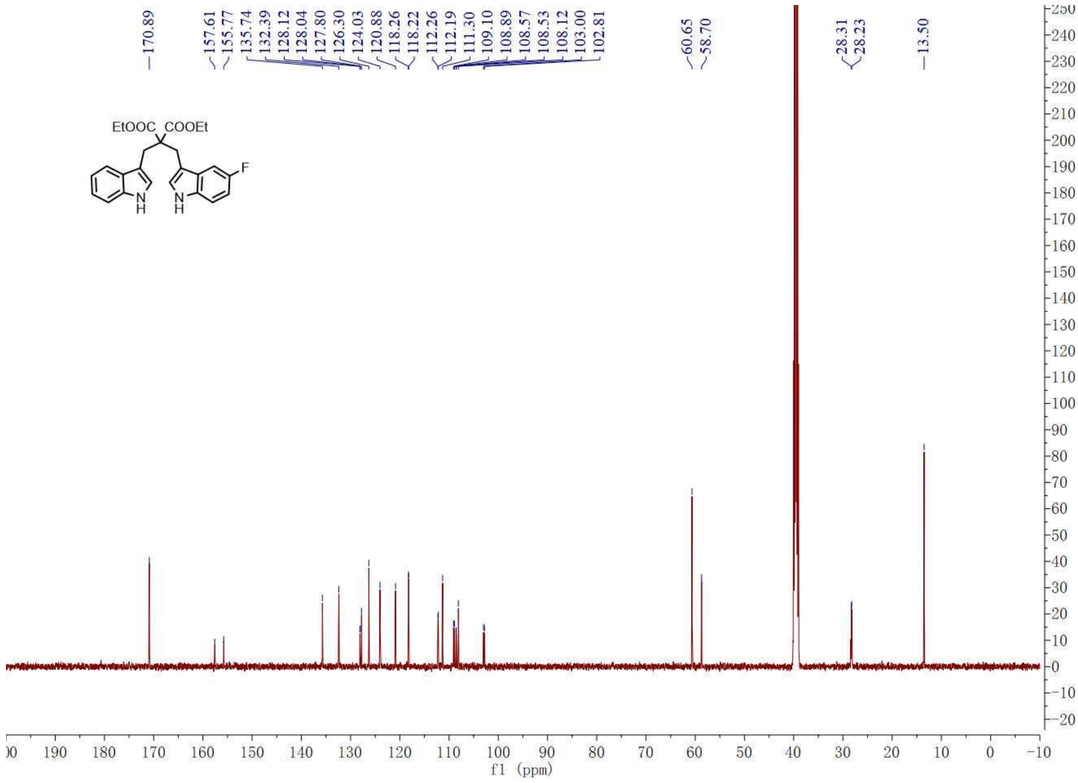

Figure S10. ^{13}C NMR spectrum of diethyl 2-((1*H*-indol-3-yl)methyl)-2-((5-fluoro-1*H*-indol-3-yl)methyl)malonate (**1e**) in DMSO-*d*6

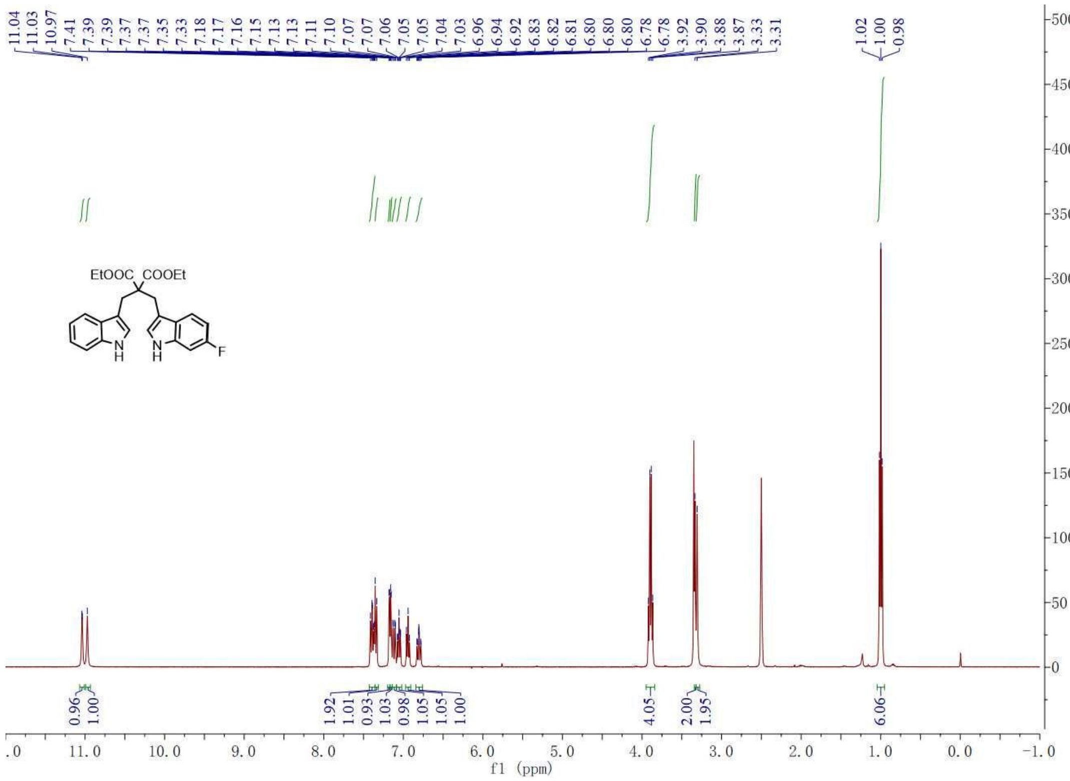

Figure S11. ^{1}H NMR spectrum of diethyl 2-((1*H*-indol-3-yl)methyl)-2-((6-fluoro-1*H*-indol-3-yl)methyl)malonate (**1f**) in DMSO-*d*6

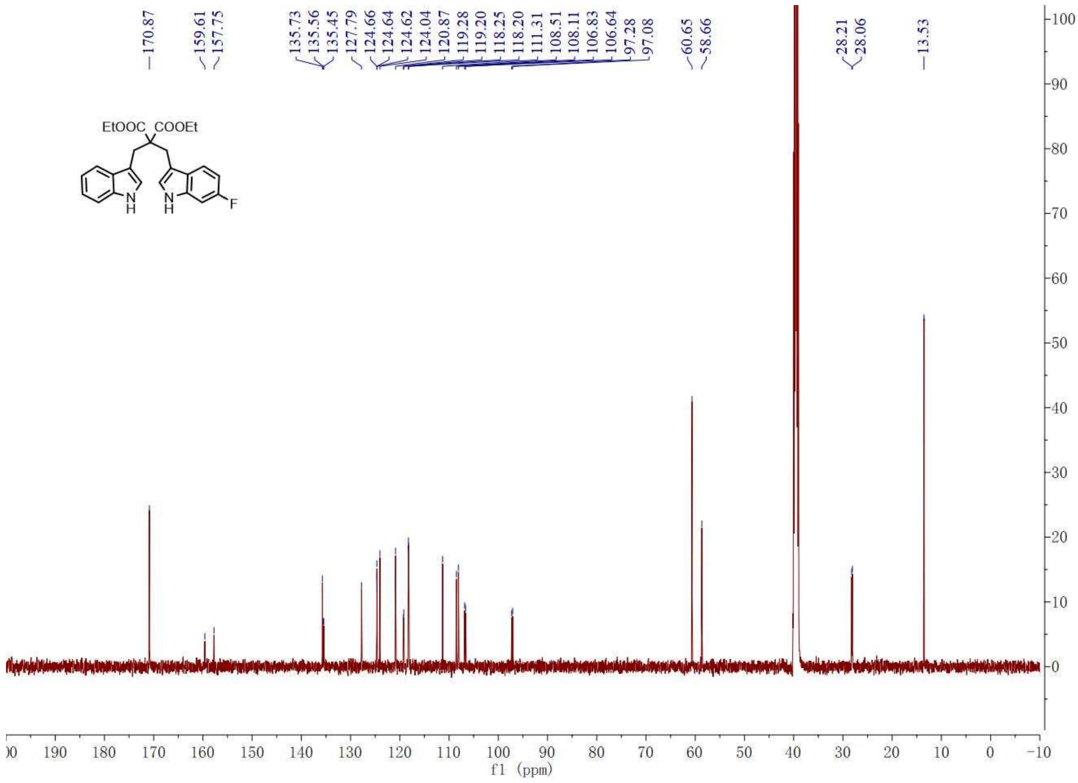

Figure S12. ^{13}C NMR spectrum of diethyl 2-((1*H*-indol-3-yl)methyl)-2-((6-fluoro-1*H*-indol-3-yl)methyl)malonate (**1f**) in DMSO-d_6

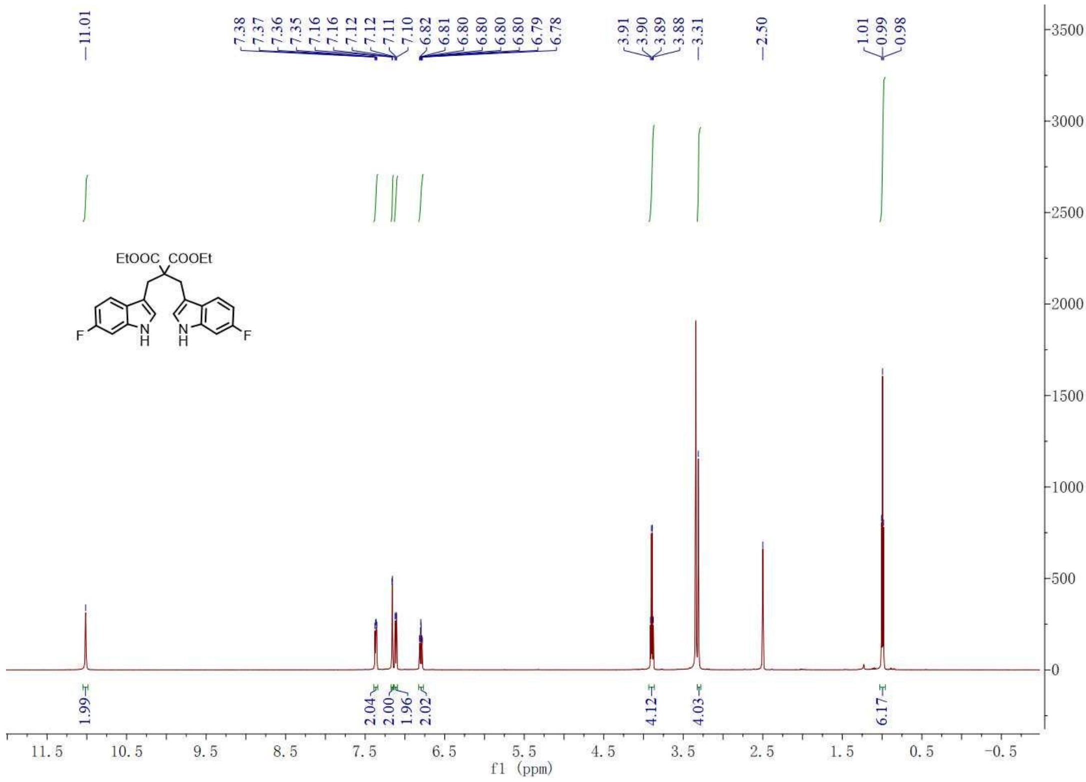

Figure S13. ^{1}H NMR spectrum of diethyl 2,2-bis((6-fluoro-1*H*-indol-3-yl)methyl)malonate (**1g**) in DMSO-d_6

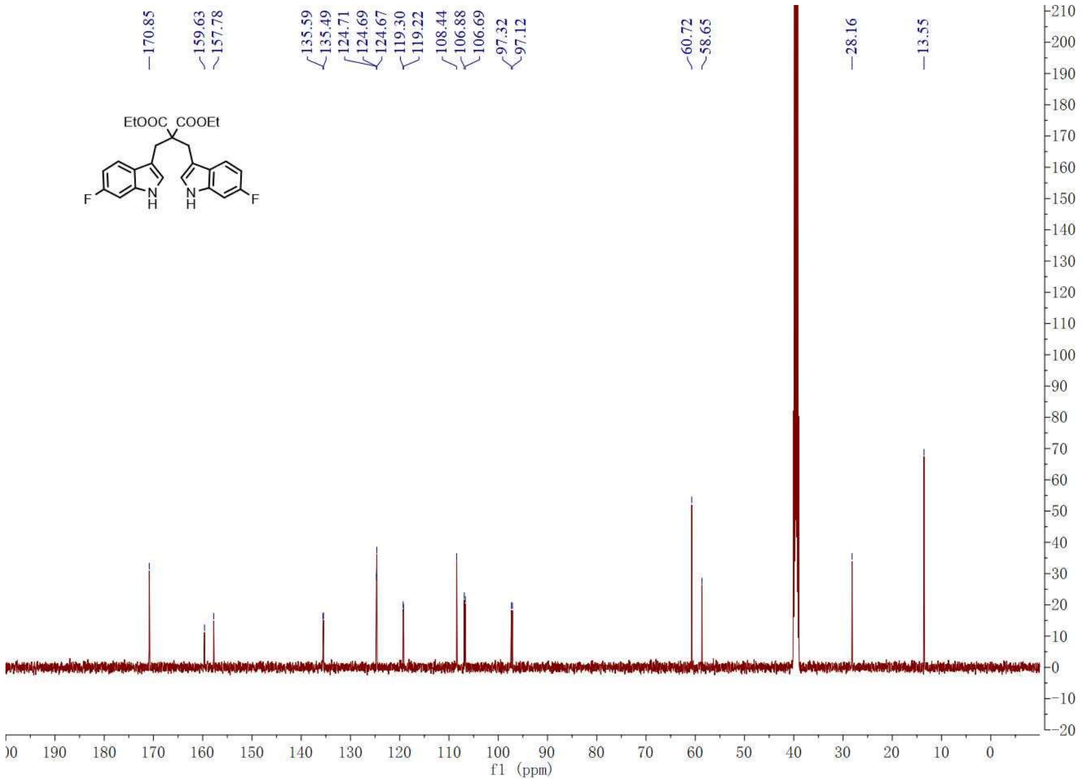

Figure S14. ^{13}C NMR spectrum of diethyl 2,2-bis((6-fluoro-1*H*-indol-3-yl)methyl)malonate (**1g**) in DMSO-d_6

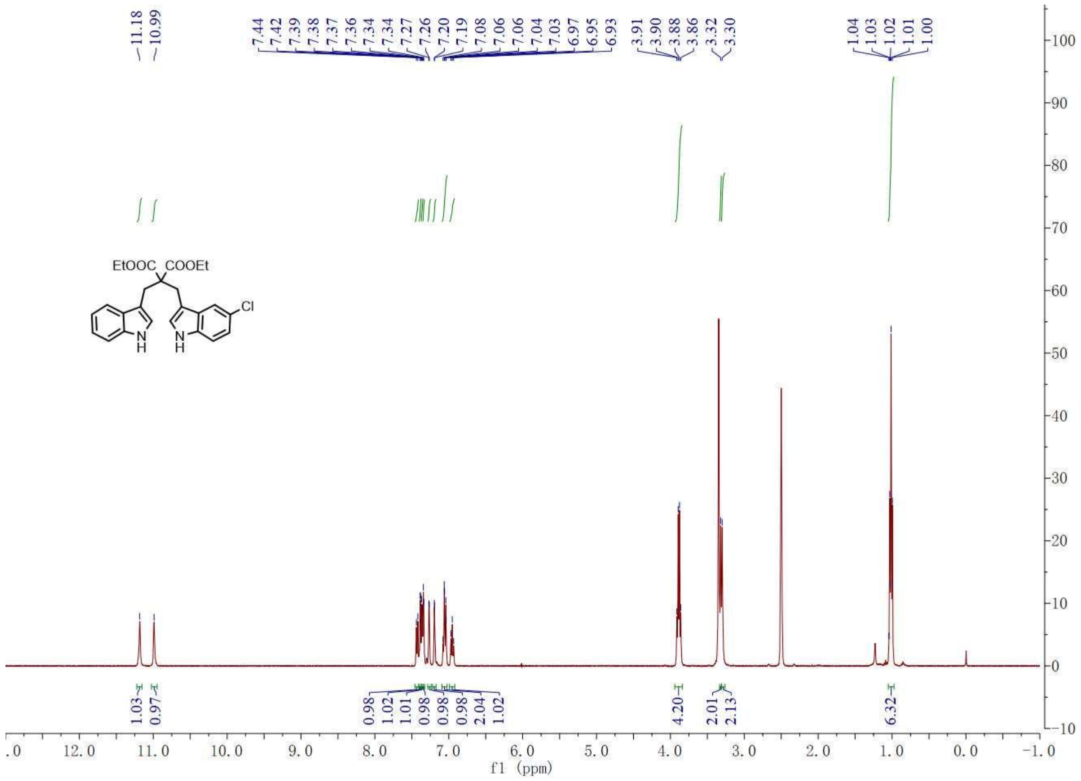

Figure S15. ^{1}H NMR spectrum of diethyl 2-((1*H*-indol-3-yl)methyl)-2-((5-chloro-1*H*-indol-3-yl)methyl)malonate (**1h**) in DMSO-d_6

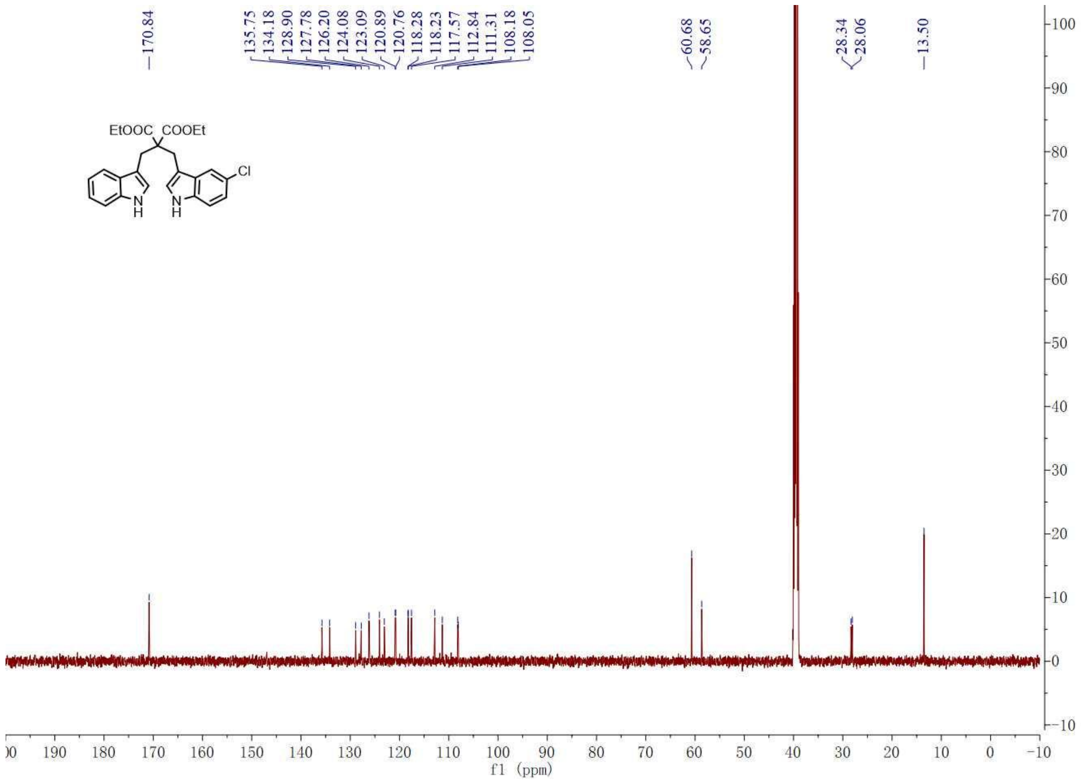

Figure S16. ^{13}C NMR spectrum of diethyl 2-((1*H*-indol-3-yl)methyl)-2-((5-chloro-1*H*-indol-3-yl)methyl)malonate (**1h**) in DMSO-d_6

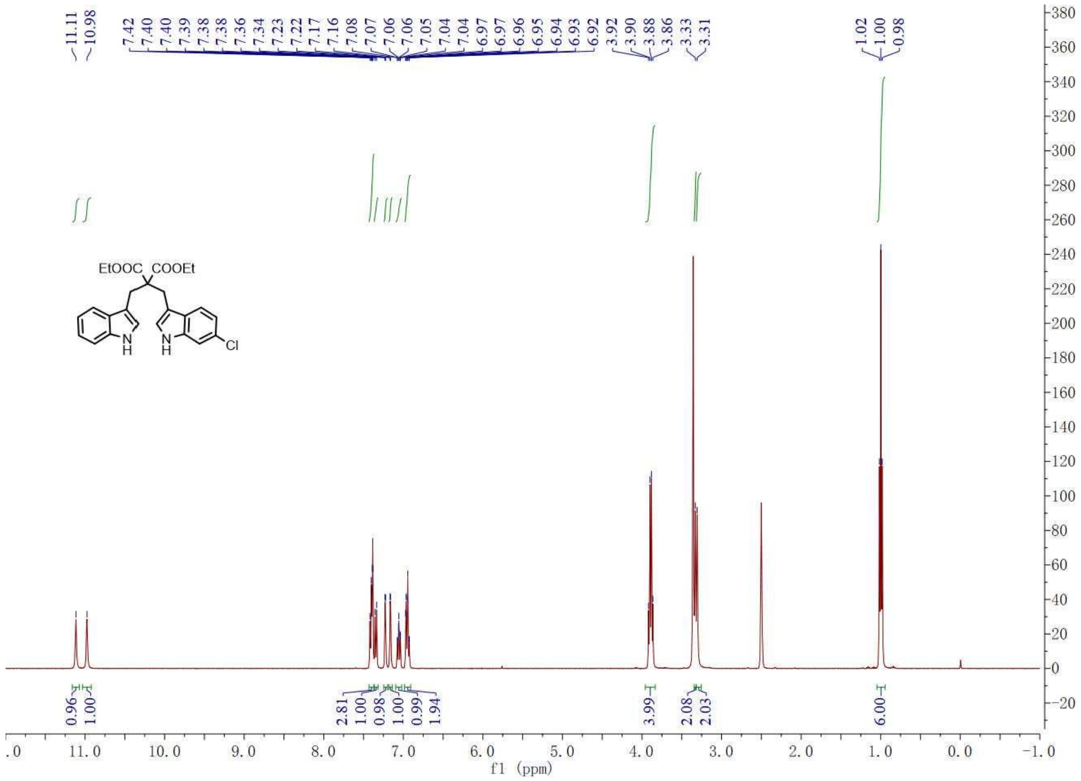

Figure S17. ^{1}H NMR spectrum of diethyl 2-((1*H*-indol-3-yl)methyl)-2-((6-chloro-1*H*-indol-3-yl)methyl)malonate (**1i**) in DMSO-d_6

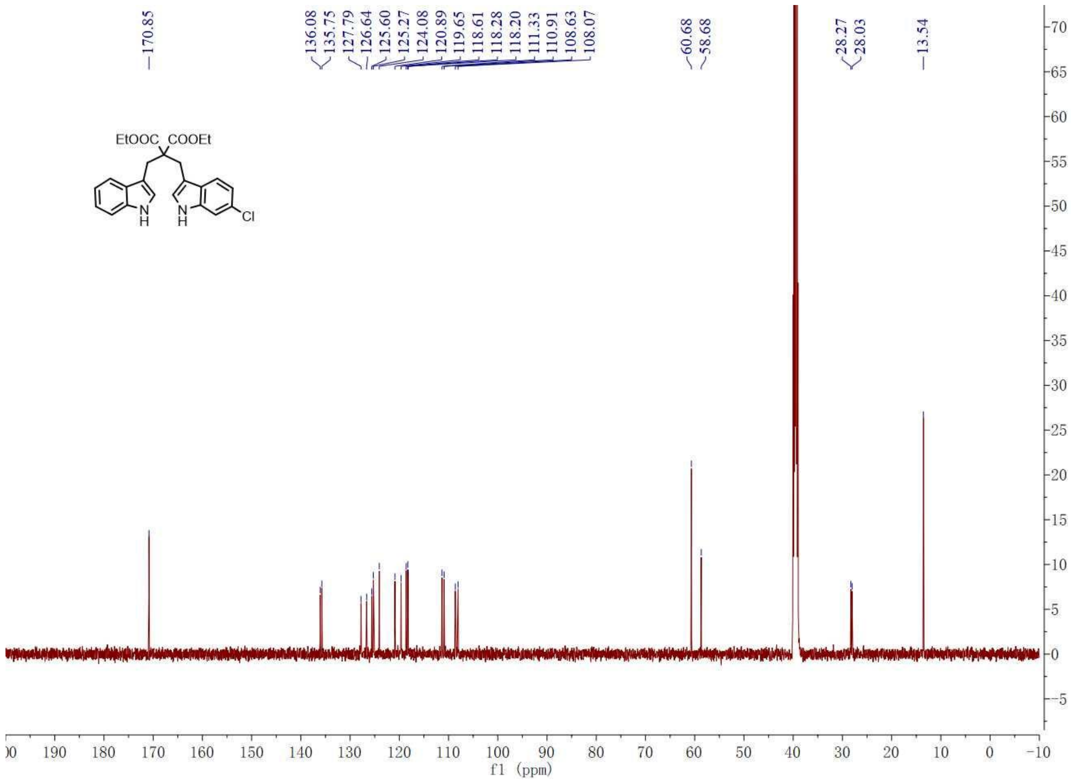

Figure S18. ^{13}C NMR spectrum of diethyl 2-((1*H*-indol-3-yl)methyl)-2-((6-chloro-1*H*-indol-3-yl)methyl)malonate (**1i**) in DMSO-d_6

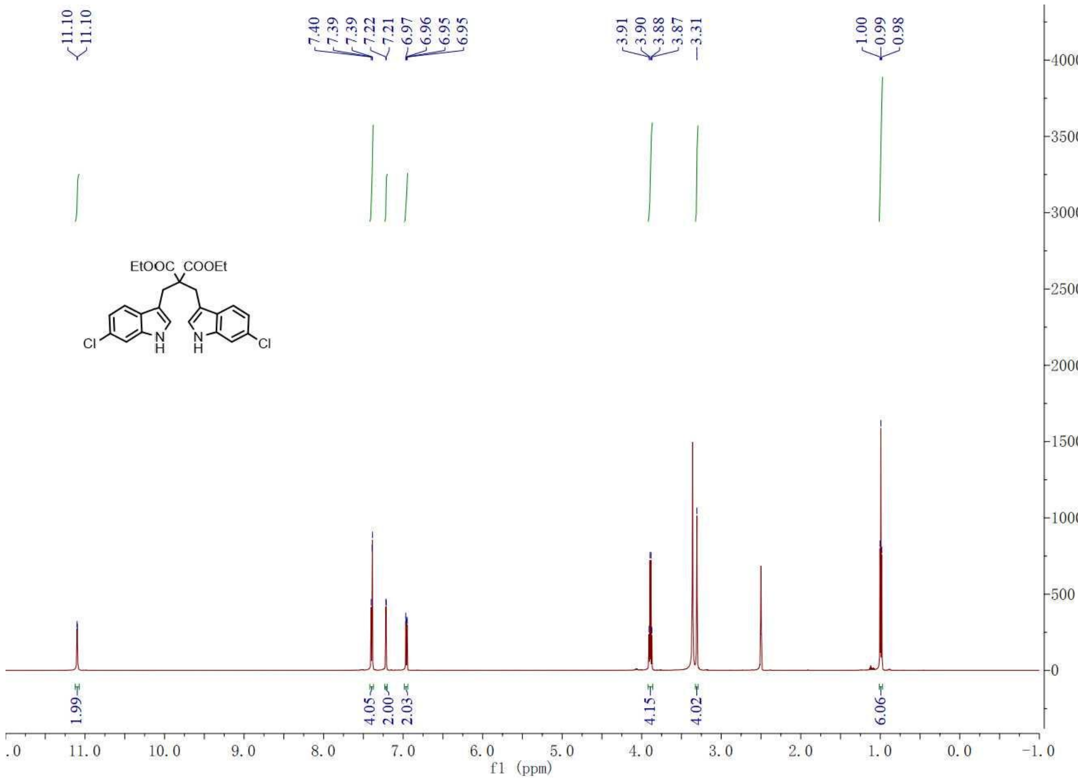

Figure S19. ^{1}H NMR spectrum of diethyl 2,2-bis((6-chloro-1*H*-indol-3-yl)methyl)malonate (**1j**) in DMSO-d_6

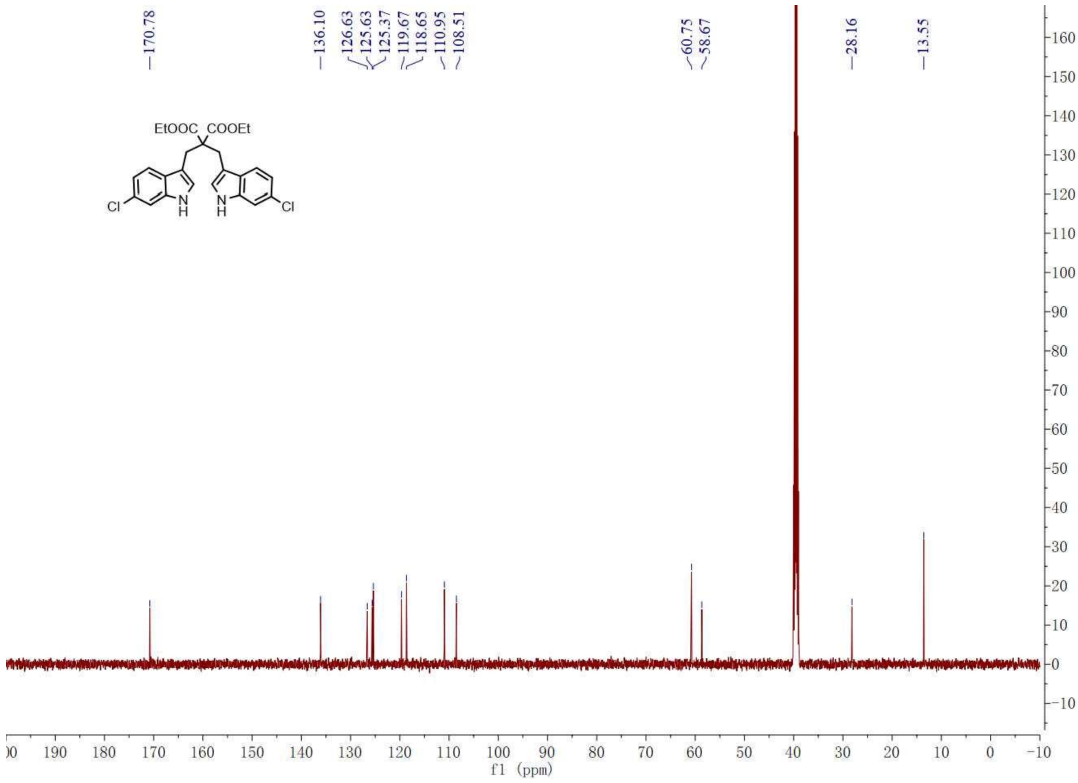

Figure S20. ^{13}C NMR spectrum of diethyl 2,2-bis((6-chloro-1*H*-indol-3-yl)methyl)malonate (**1j**) in DMSO-d_6

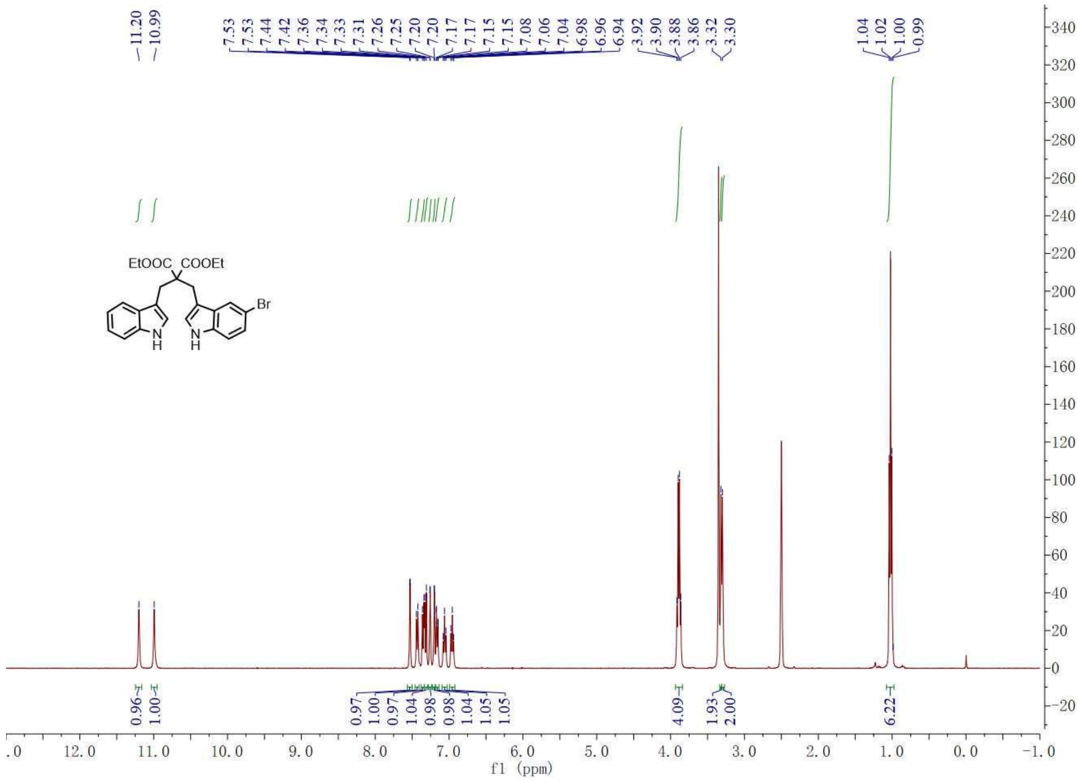

Figure S21. ^{1}H NMR spectrum of diethyl 2-((1*H*-indol-3-yl)methyl)-2-((5-bromo-1*H*-indol-3-yl)methyl)malonate (**1k**) in DMSO-d_6

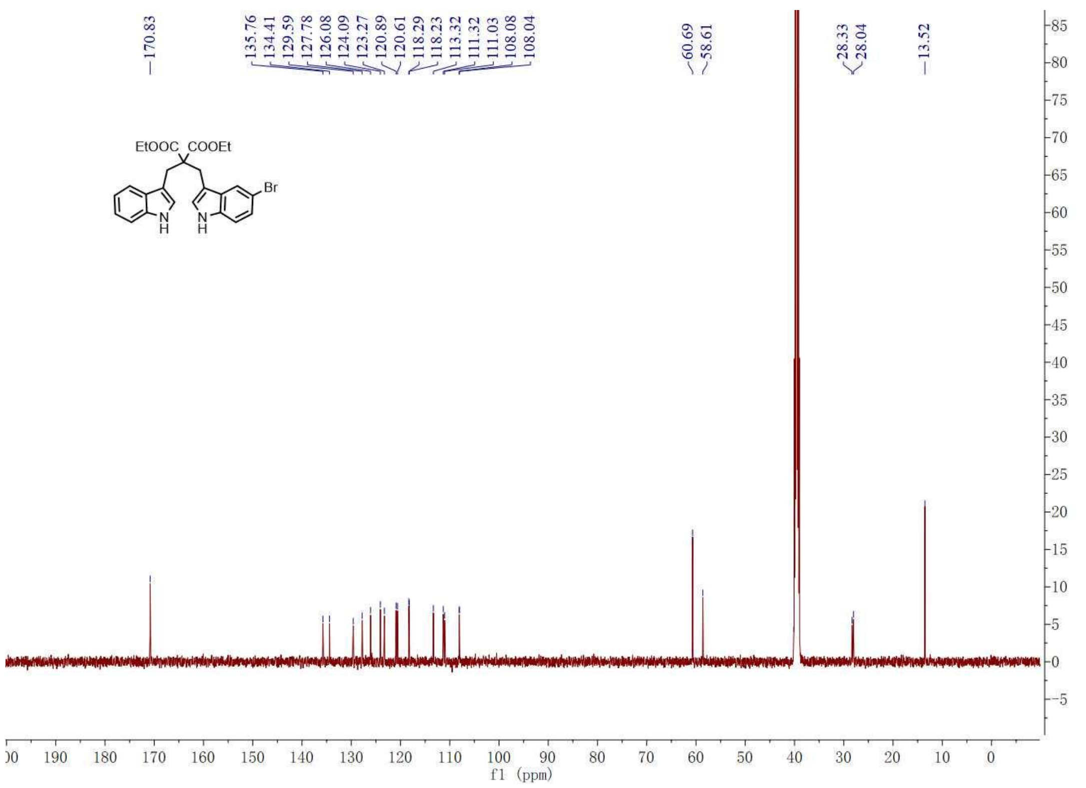

Figure S22. ^{13}C NMR spectrum of diethyl 2-((1*H*-indol-3-yl)methyl)-2-((5-bromo-1*H*-indol-3-yl)methyl)malonate (**1k**) in DMSO-d_6

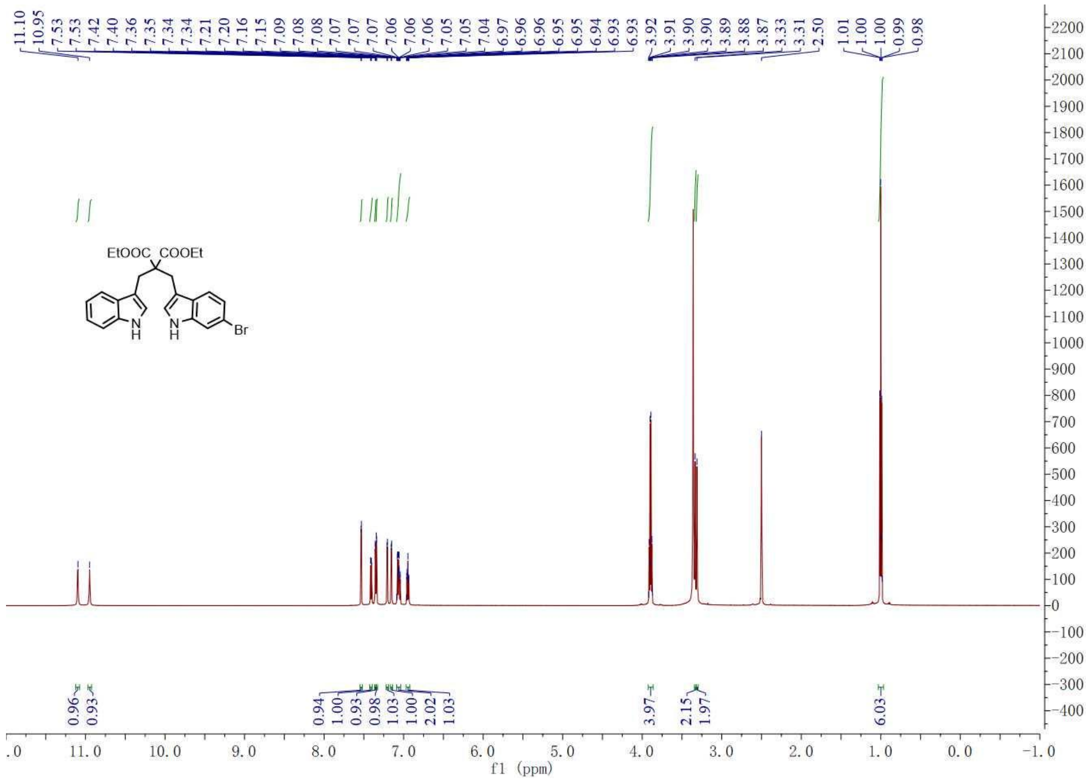

Figure S23. ^{1}H NMR spectrum of diethyl 2-((1*H*-indol-3-yl)methyl)-2-((6-bromo-1*H*-indol-3-yl)methyl)malonate (**1l**) in DMSO-d_6

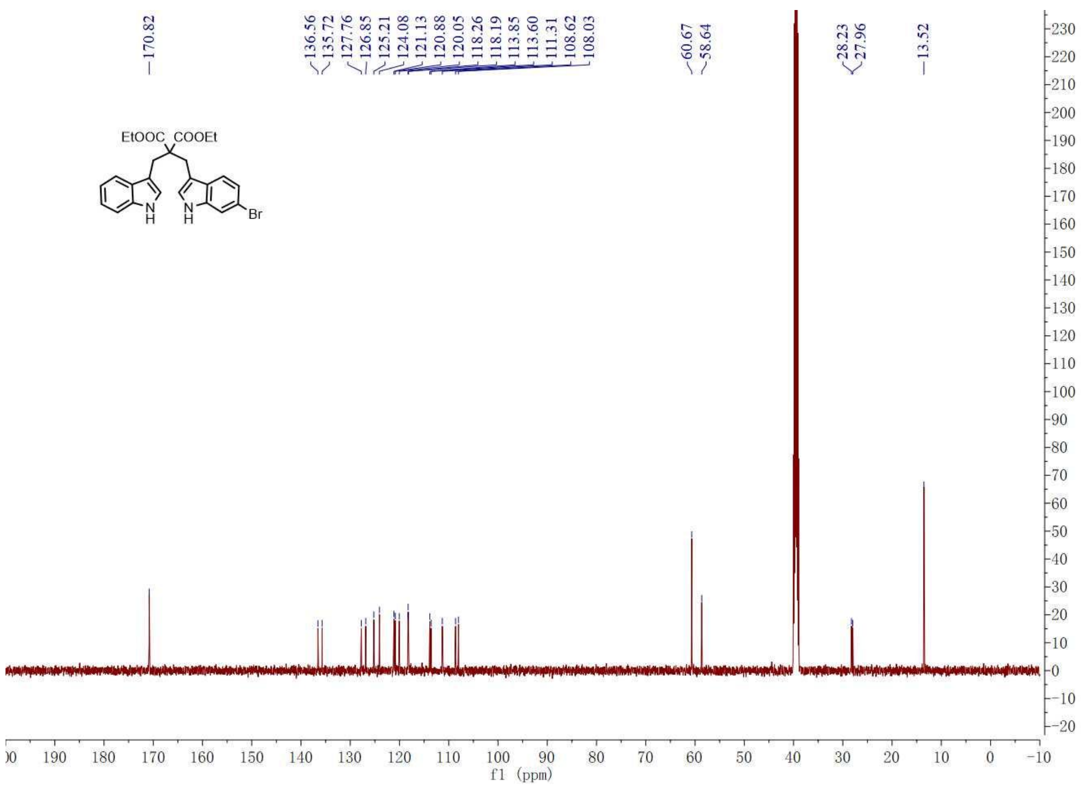

Figure S24. ^{13}C NMR spectrum of diethyl 2-((1*H*-indol-3-yl)methyl)-2-((6-bromo-1*H*-indol-3-yl)methyl)malonate (**1l**) in DMSO-d_6

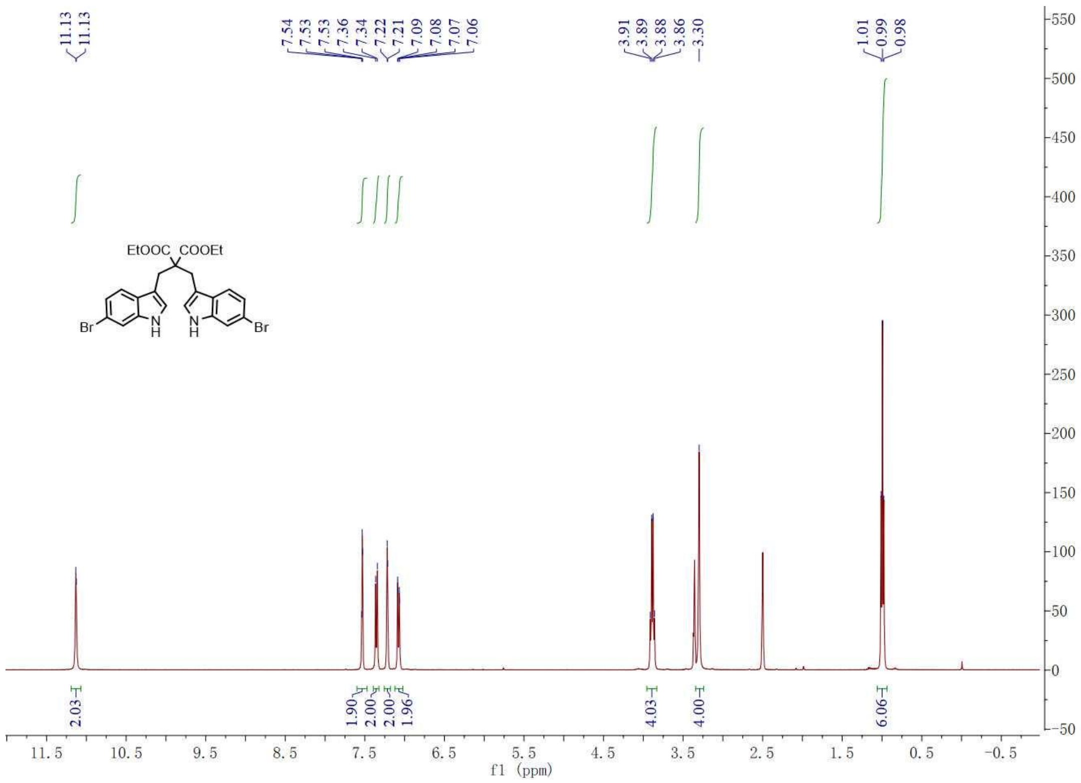

Figure S25. ^{1}H NMR spectrum of diethyl 2,2-bis((6-bromo-1*H*-indol-3-yl)methyl)malonate (**1m**) in DMSO-d_6

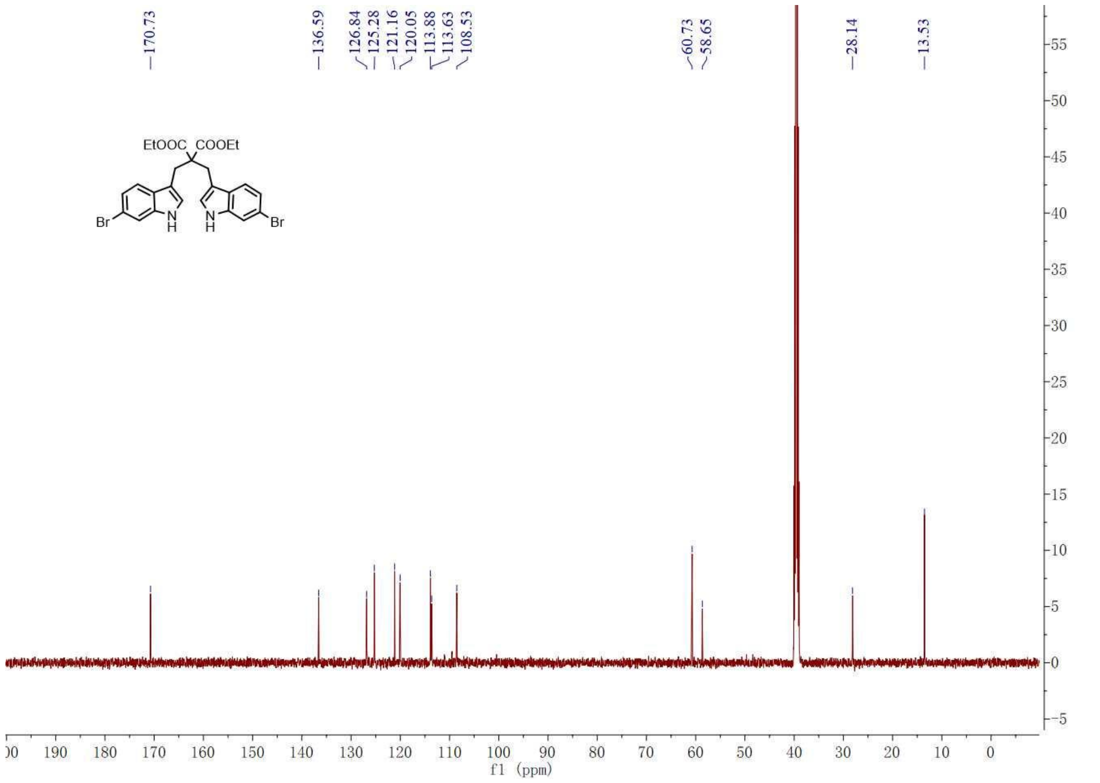

Figure S26. ^{13}C NMR spectrum of diethyl 2,2-bis((6-bromo-1*H*-indol-3-yl)methyl)malonate (**1m**) in DMSO-d_6

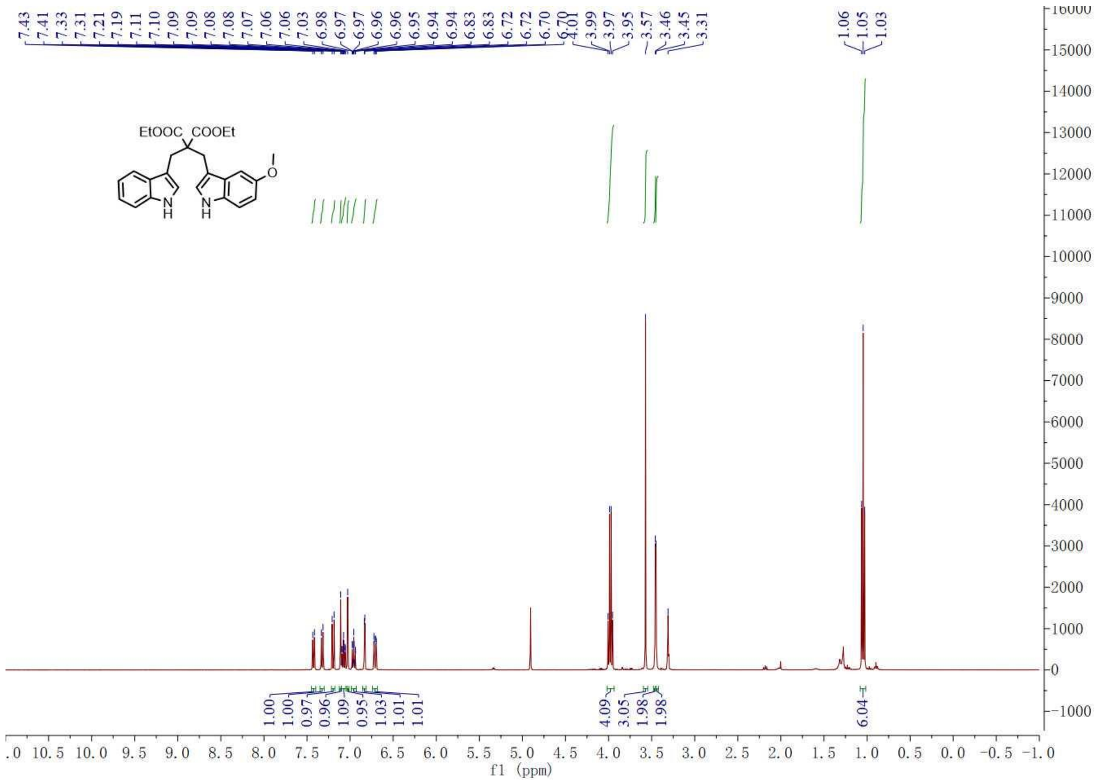

Figure S27. ^{1}H NMR spectrum of diethyl 2-((1*H*-indol-3-yl)methyl)-2-((5-methoxy-1*H*-indol-3-yl)methyl)malonate (**1n**) in Methanol-d_4

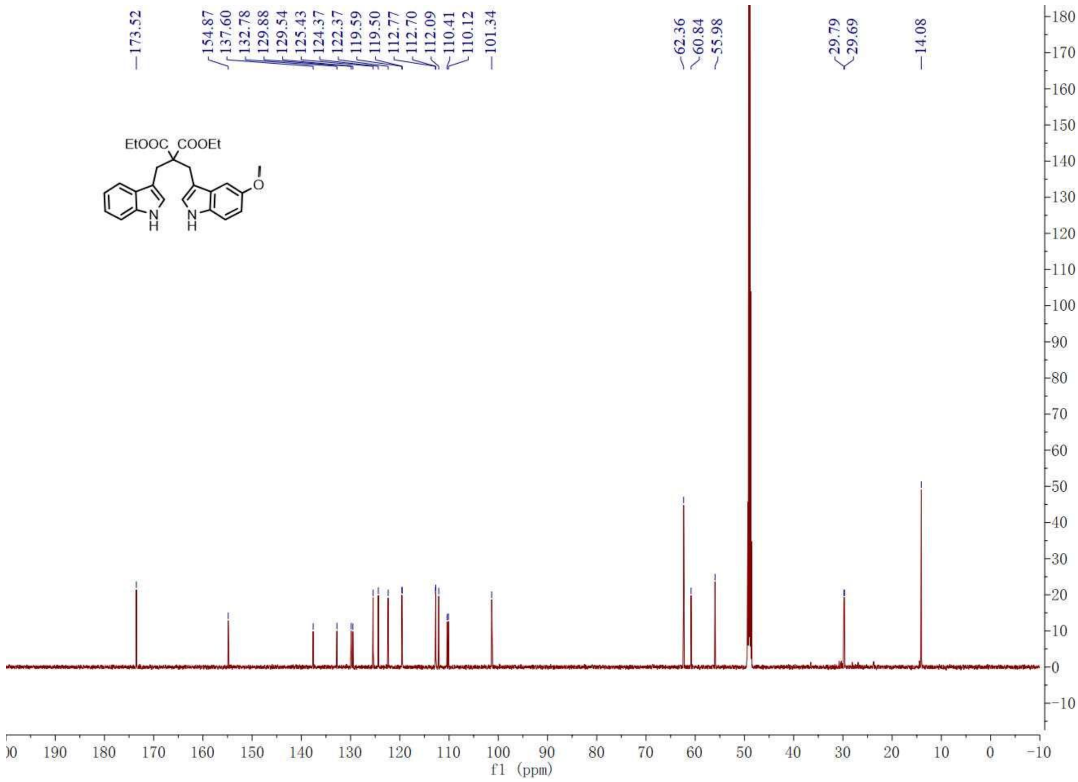

Figure S28. ^{13}C NMR spectrum of diethyl 2-((1*H*-indol-3-yl)methyl)-2-((5-methoxy-1*H*-indol-3-yl)methyl)malonate (**1n**) in Methanol-d_4

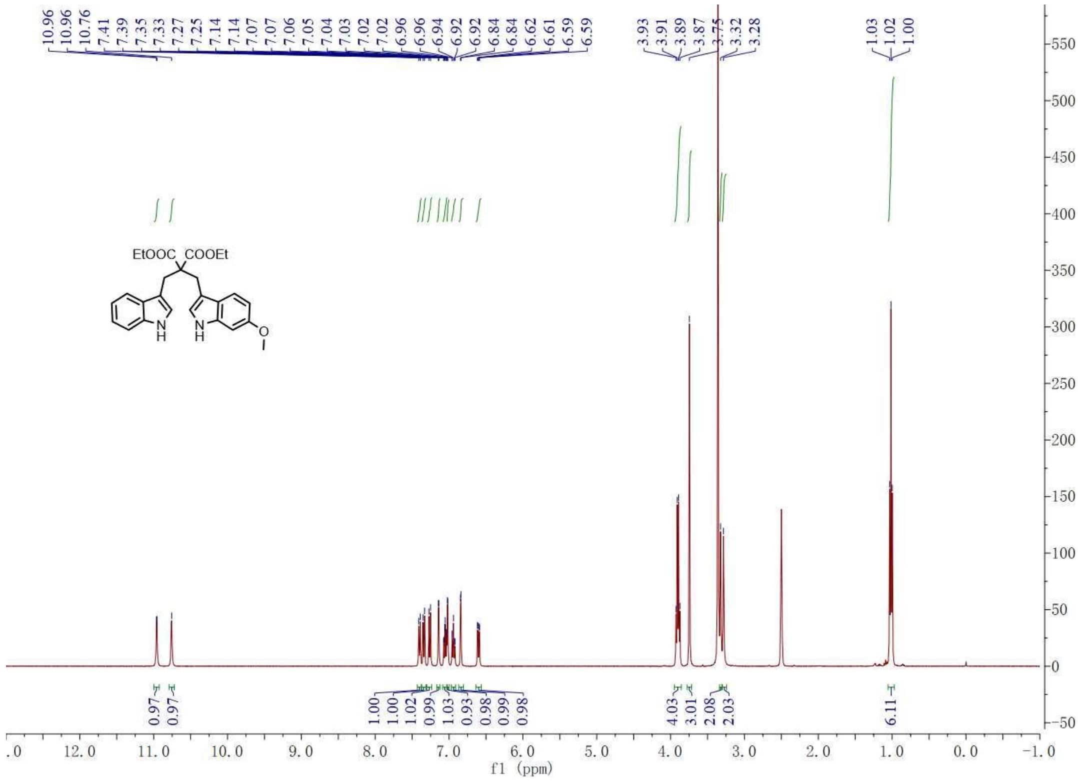

Figure S29. ^{1}H NMR spectrum of diethyl 2-((1*H*-indol-3-yl)methyl)-2-((6-methoxy-1*H*-indol-3-yl)methyl)malonate (**1o**) in DMSO-d_6

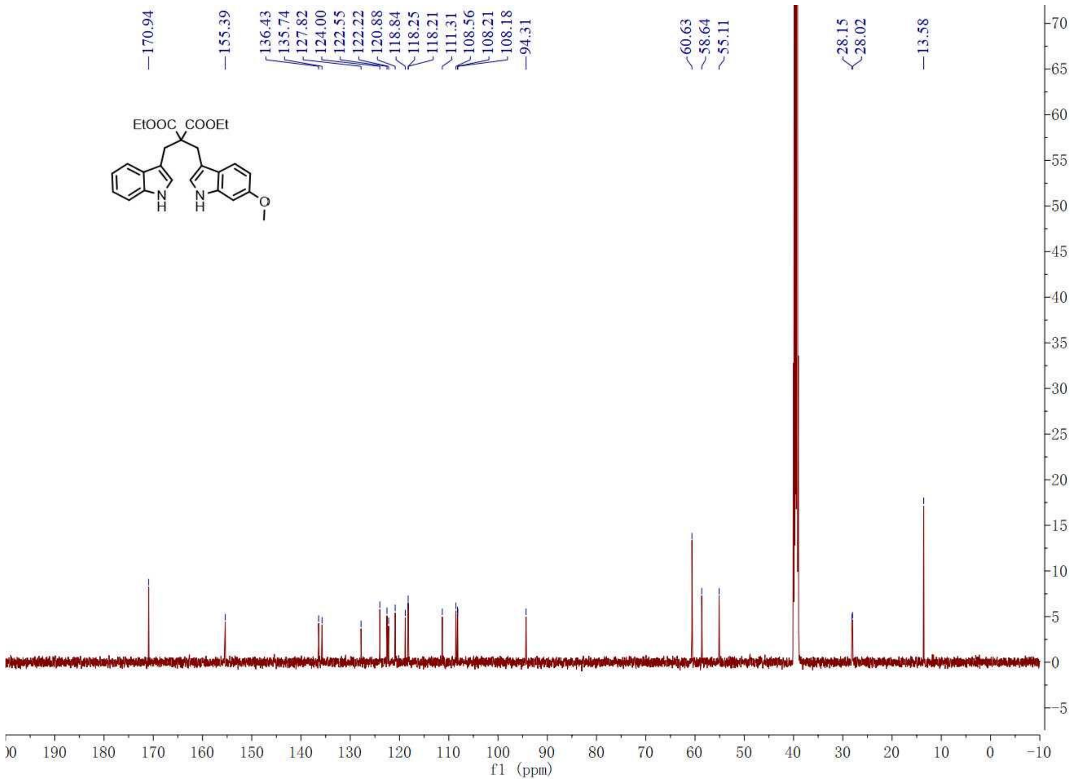

Figure S30. ^{13}C NMR spectrum of diethyl 2-((1*H*-indol-3-yl)methyl)-2-((6-methoxy-1*H*-indol-3-yl)methyl)malonate (**1o**) in DMSO-d_6

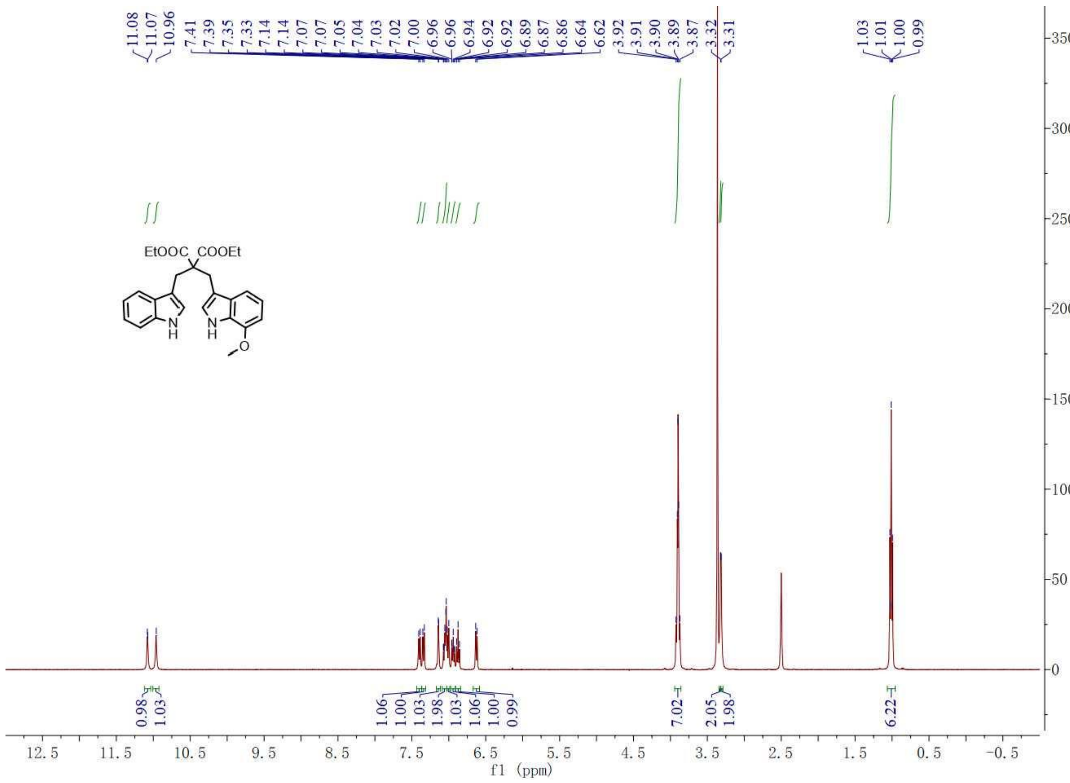

Figure S31. ^{1}H NMR spectrum of diethyl 2-((1*H*-indol-3-yl)methyl)-2-((7-methoxy-1*H*-indol-3-yl)methyl)malonate (**1p**) in DMSO-d_6

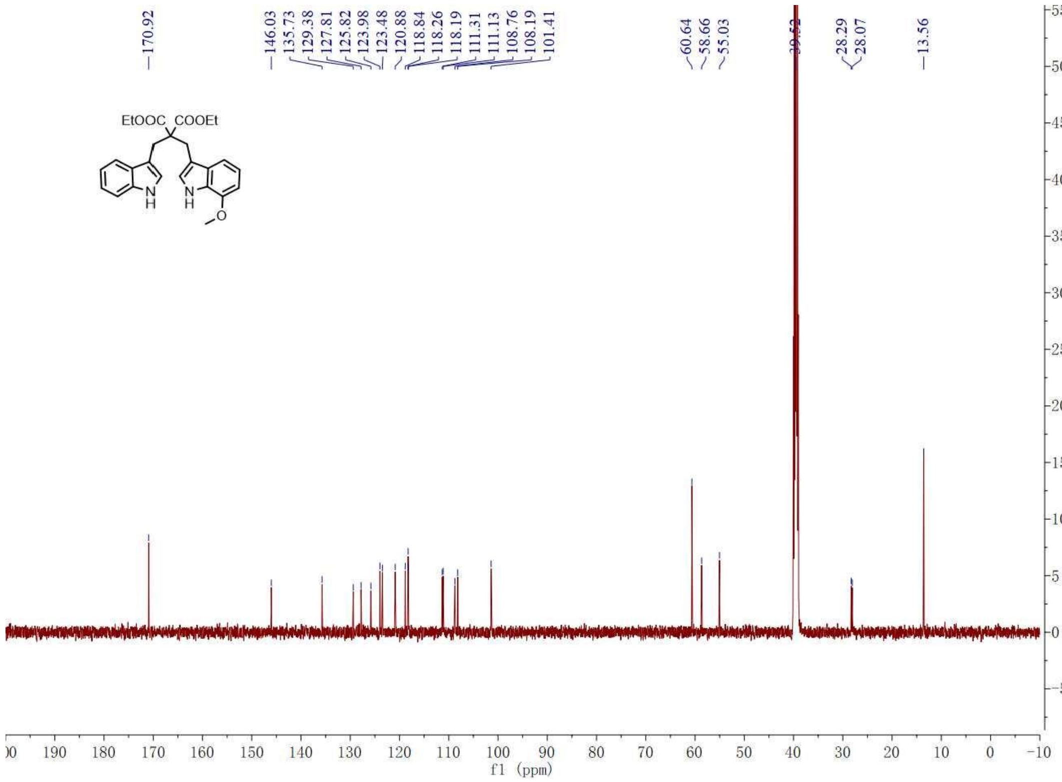

Figure S32. ^{13}C NMR spectrum of diethyl 2-((1*H*-indol-3-yl)methyl)-2-((7-methoxy-1*H*-indol-3-yl)methyl)malonate (**1p**) in DMSO-d_6

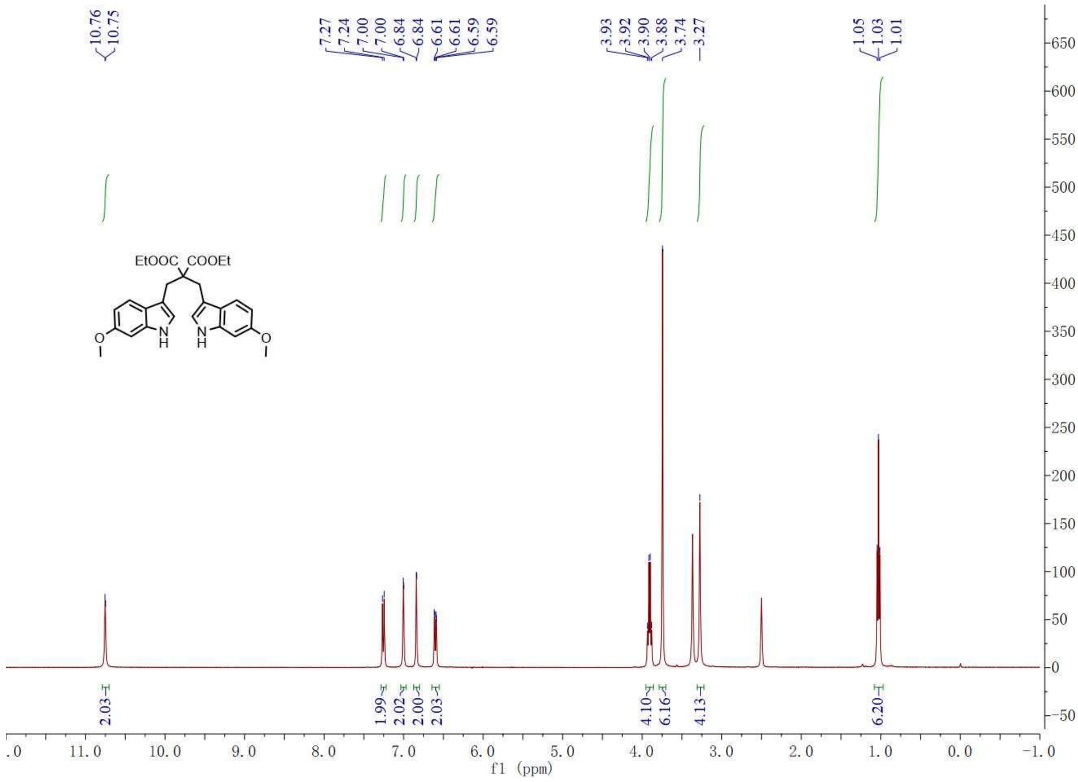

Figure S33. ^{1}H NMR spectrum of diethyl 2,2-bis((6-methoxy-1*H*-indol-3-yl)methyl)malonate (**1q**) in DMSO-d_6

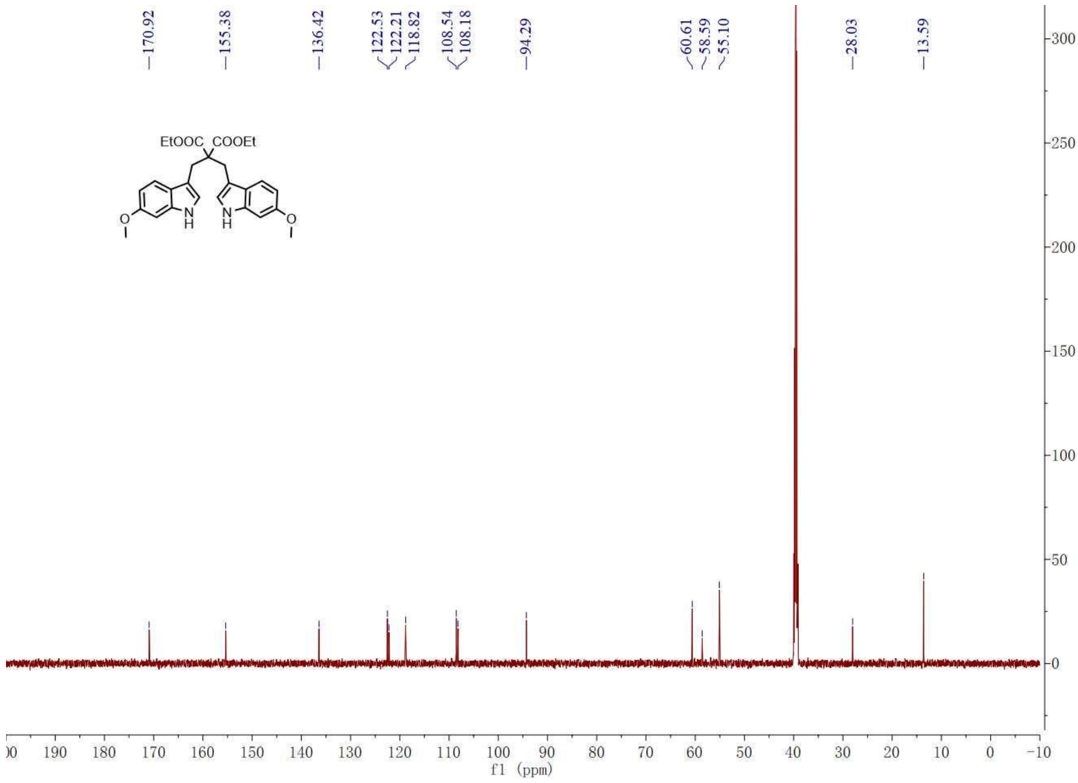

Figure S34. ^{13}C NMR spectrum of diethyl 2,2-bis((6-methoxy-1*H*-indol-3-yl)methyl)malonate (**1q**) in DMSO-d_6

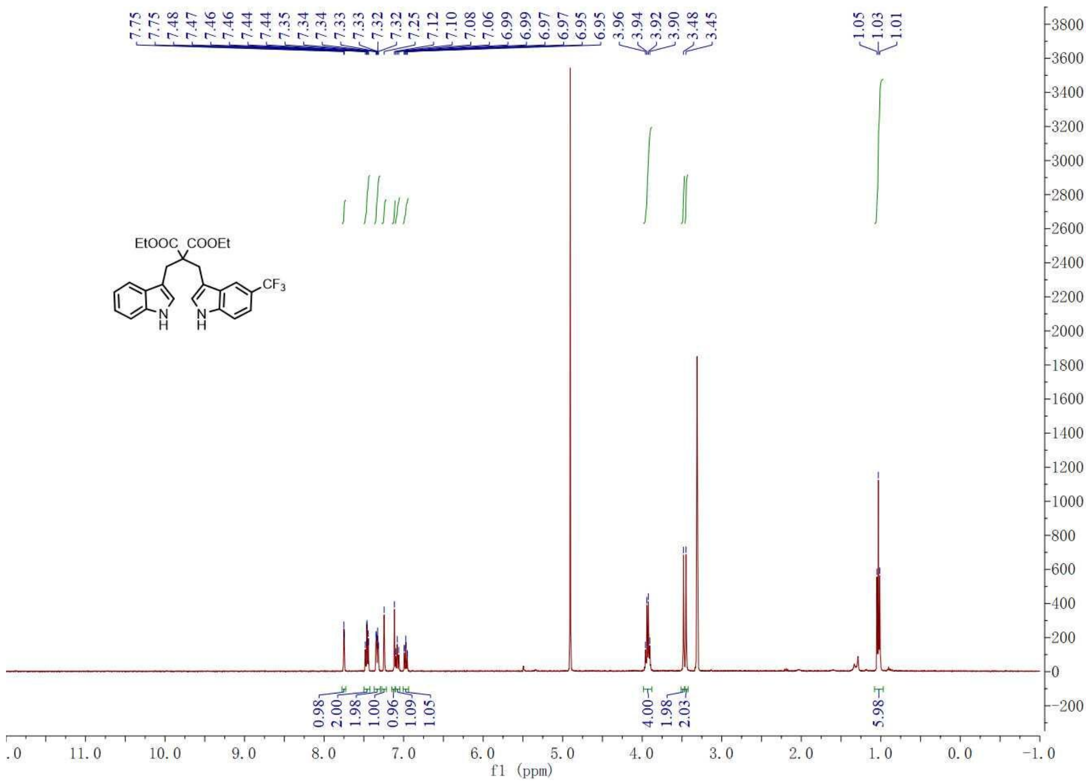

Figure S35. ^{1}H NMR spectrum of diethyl 2-((1*H*-indol-3-yl)methyl)-2-((5-(trifluoromethyl)-1*H*-indol-3-yl)methyl)malonate (**1r**) in Methanol-d_4

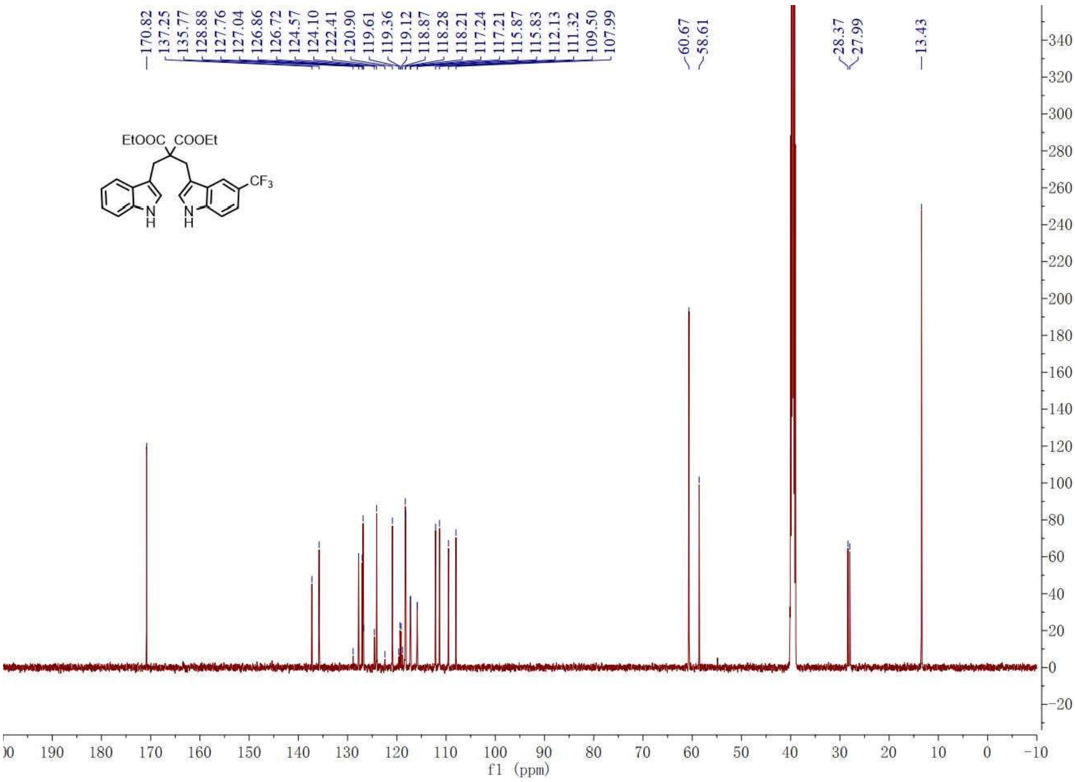

Figure S36. ^{13}C NMR spectrum of diethyl 2-((1*H*-indol-3-yl)methyl)-2-((5-(trifluoromethyl)-1*H*-indol-3-yl)methyl)malonate (**1r**) in DMSO-d_6

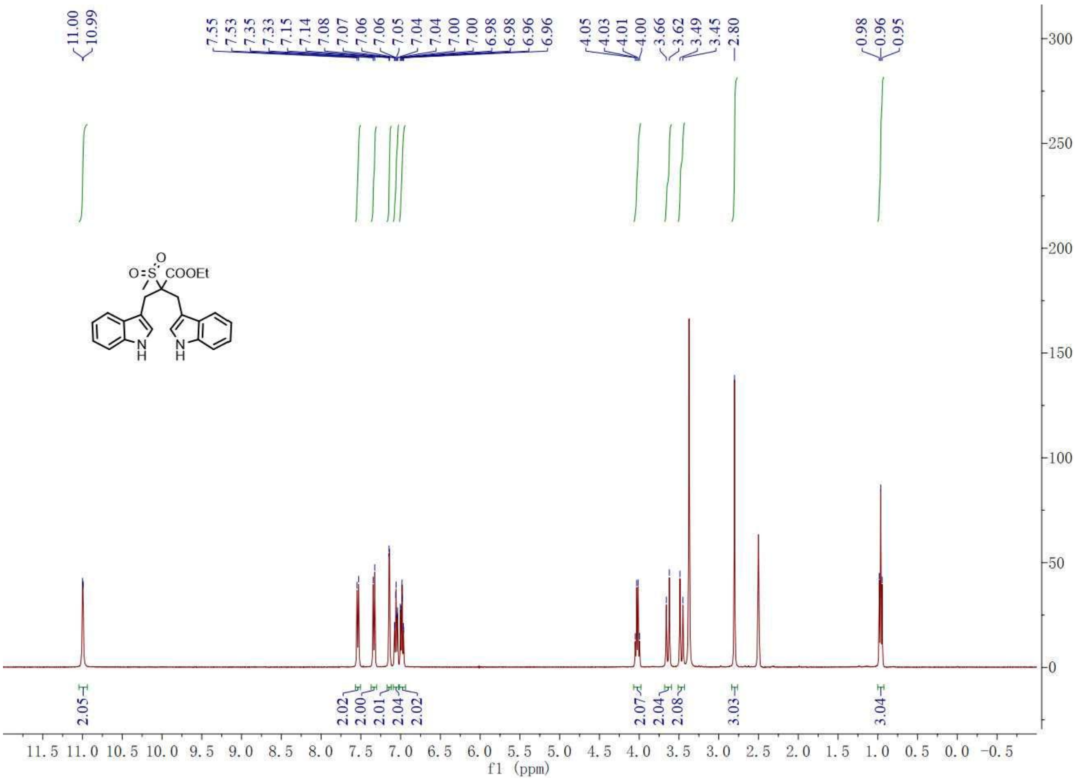

Figure S37. ^{1}H NMR spectrum of ethyl 2-((1*H*-indol-3-yl)methyl)-3-(1*H*-indol-3-yl)-2-(methylsulfonyl)propanoate (**1s**) in DMSO-d_6

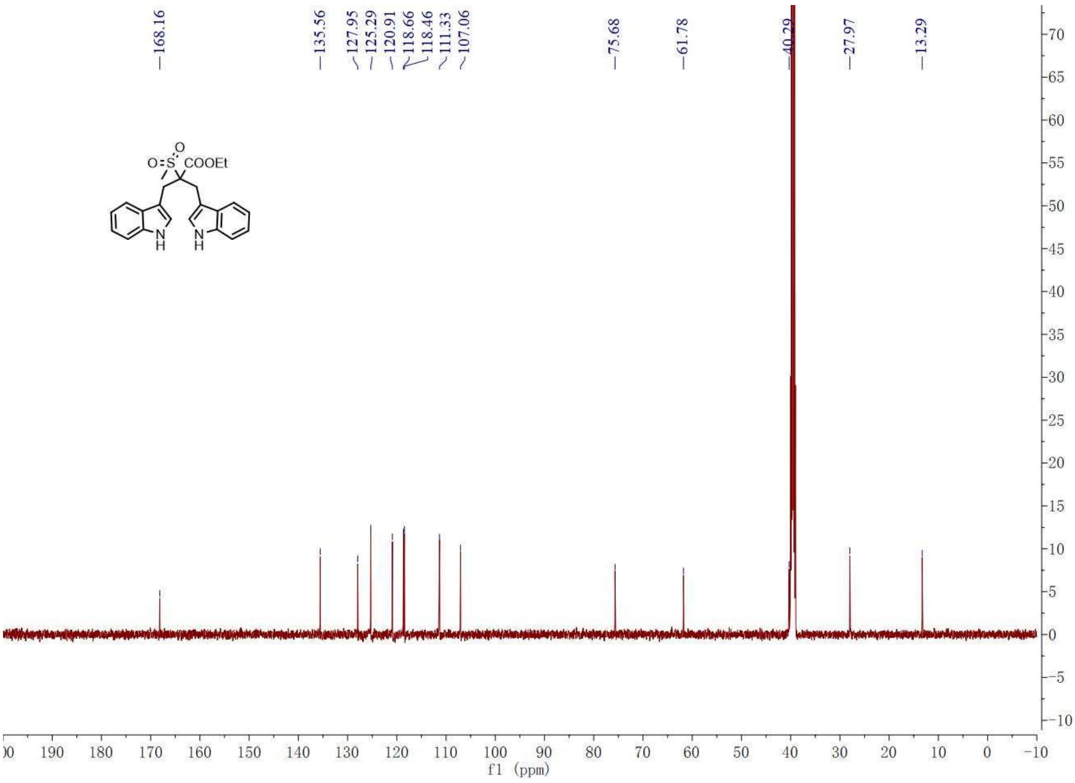

Figure S38. ^{13}C NMR spectrum of ethyl 2-((1*H*-indol-3-yl)methyl)-3-(1*H*-indol-3-yl)-2-(methylsulfonyl)propanoate (**1s**) in DMSO-*d*$_6$

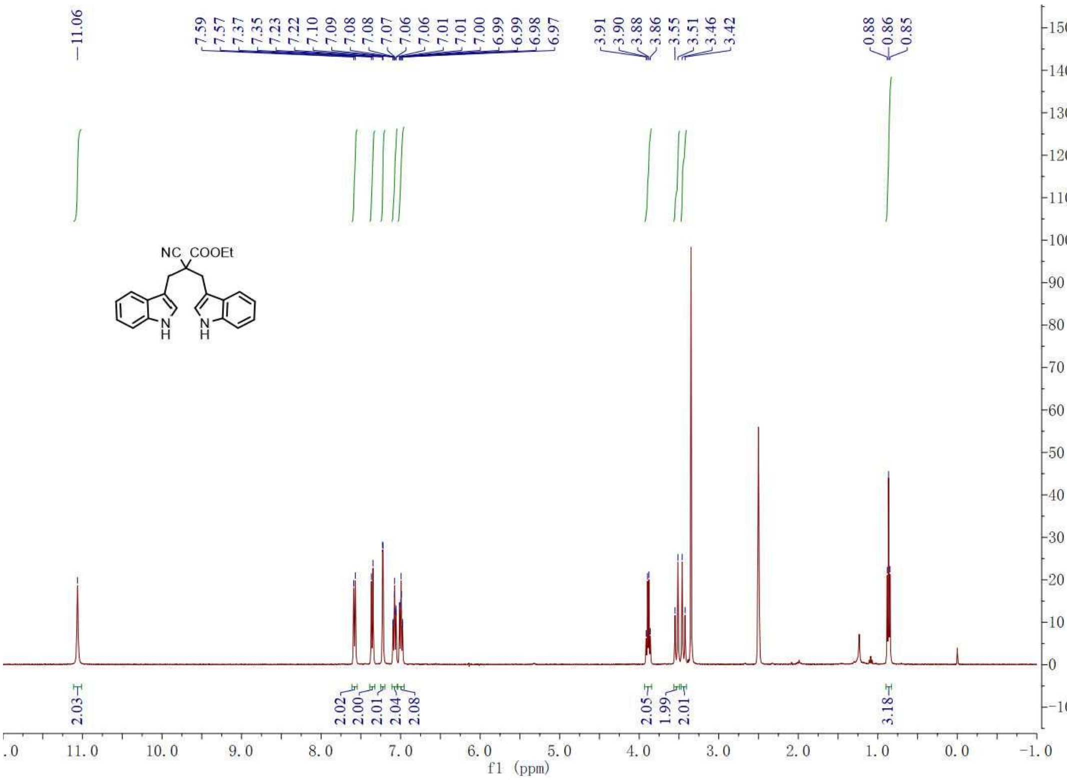

Figure S39. ^{1}H NMR spectrum of ethyl 2-((1*H*-indol-3-yl)methyl)-2-cyano-3-(1*H*-indol-3-yl)propanoate (**1t**) in DMSO-*d*$_6$

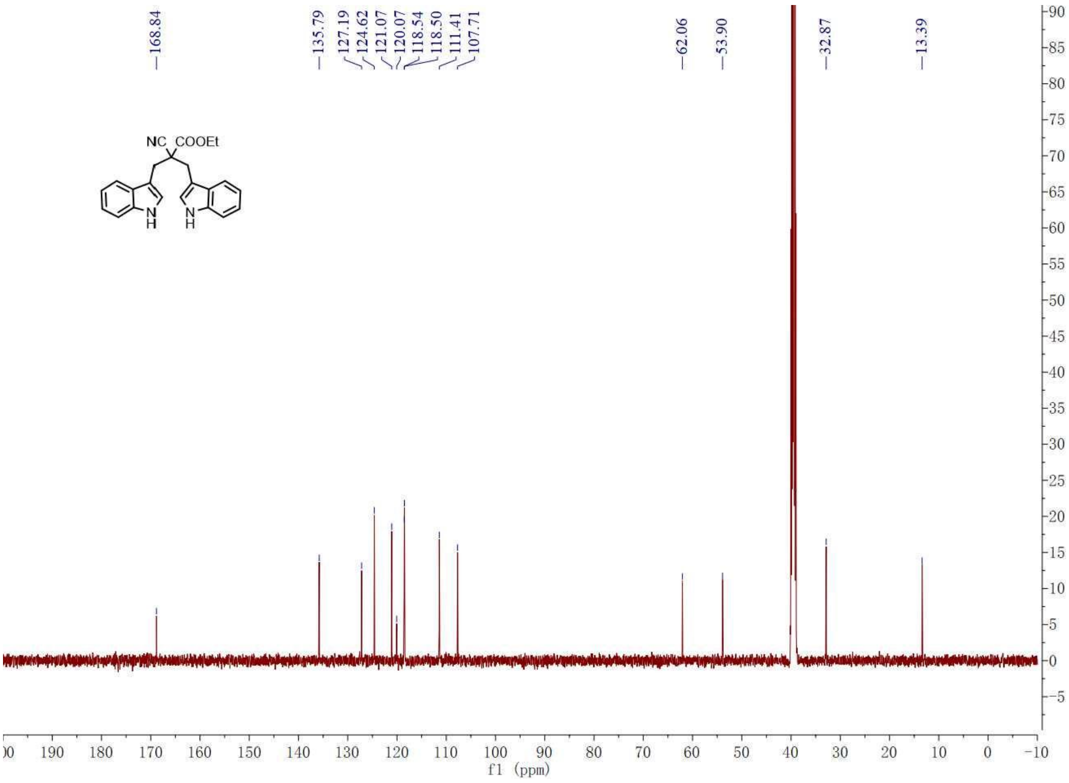

Figure S40. ^{13}C NMR spectrum of ethyl 2-((1*H*-indol-3-yl)methyl)-2-cyano-3-(1*H*-indol-3-yl)propanoate (**1t**) in DMSO-d_6

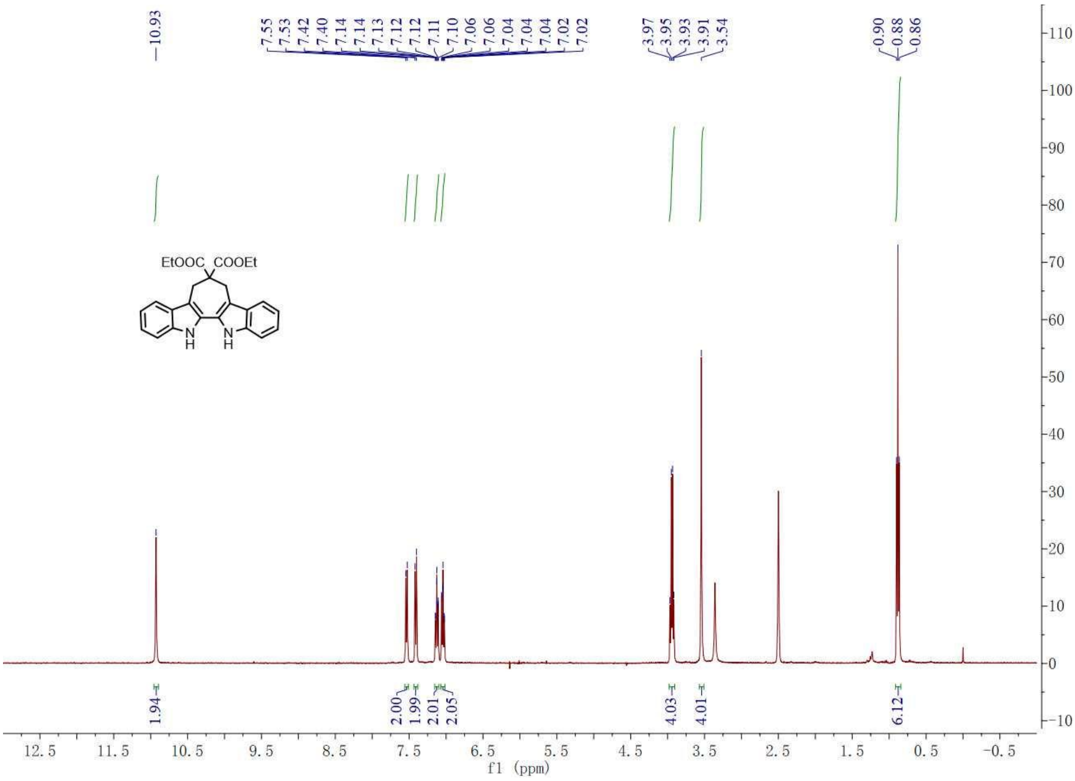

Figure S41. ^{1}H NMR spectrum of diethyl 5,7,12,13-tetrahydro-6*H*-cyclohepta[2,1-*b*:3,4-*b'*]diindole-6,6-dicarboxylate (**2a**) in DMSO-d_6

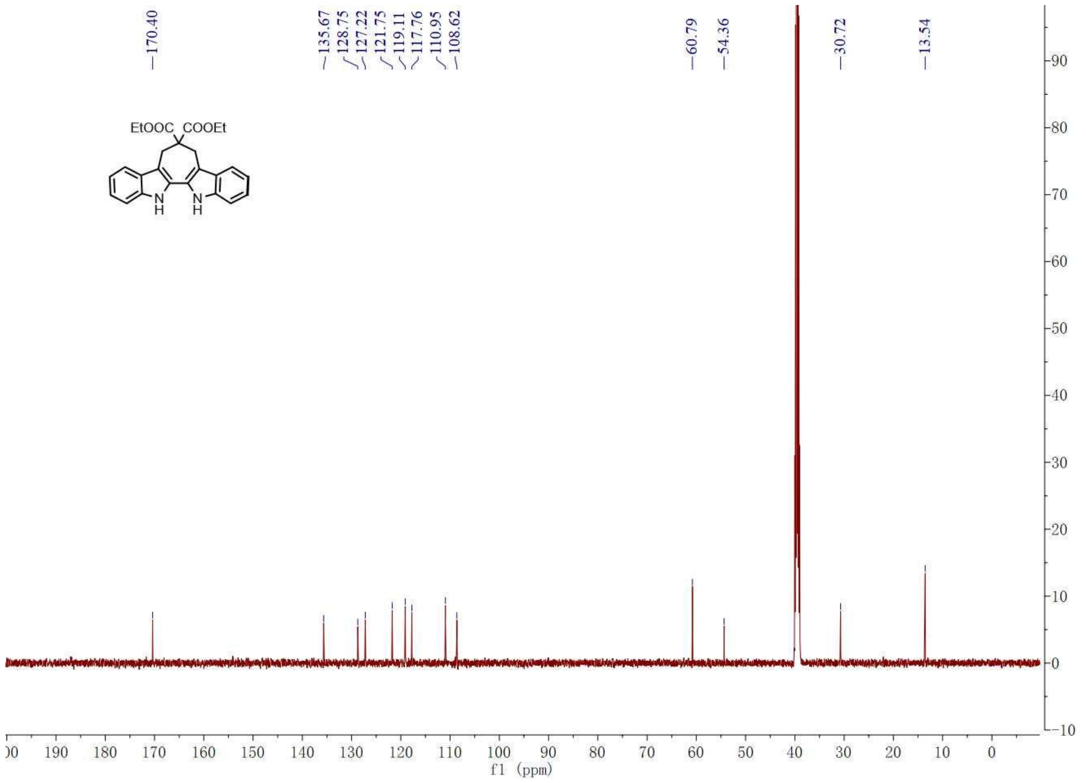

Figure S42. ^{13}C NMR spectrum of diethyl 5,7,12,13-tetrahydro-6*H*-cyclohepta[2,1-*b*:3,4-*b'*]diindole-6,6-dicarboxylate (**2a**) in DMSO-d_6

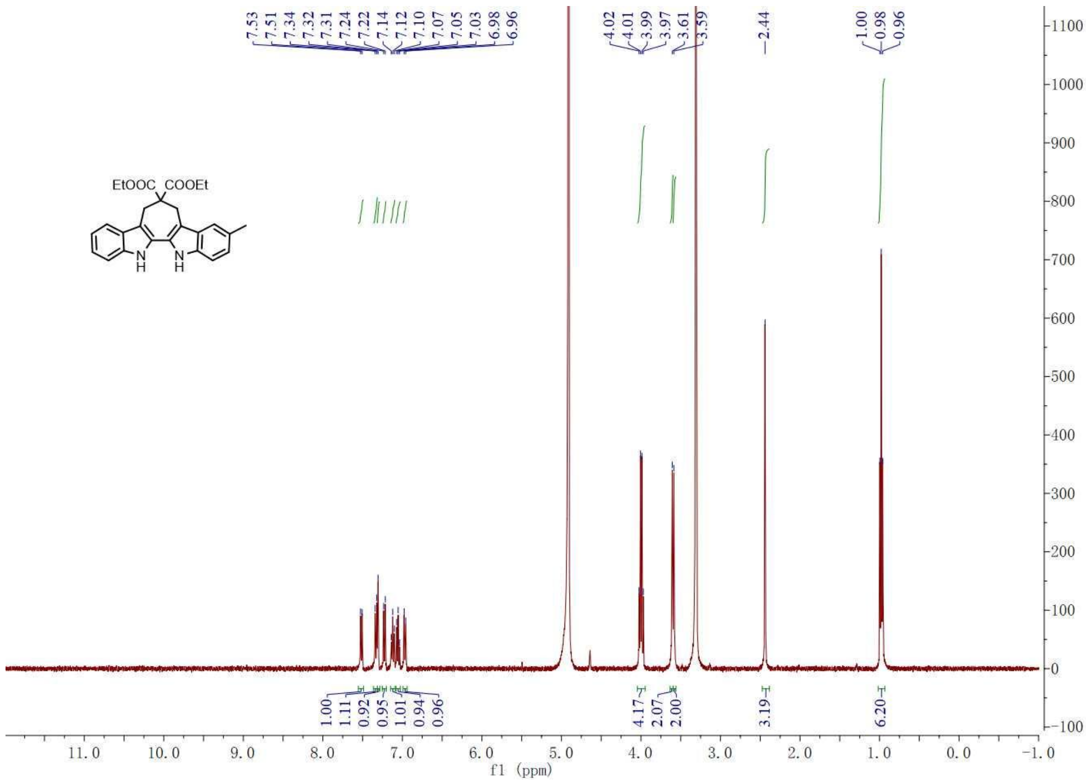

Figure S43. ^{1}H NMR spectrum of diethyl 3-methyl-5,7,12,13-tetrahydro-6*H*-cyclohepta[2,1-*b*:3,4-*b'*]diindole-6,6-dicarboxylate (**2b**) in Methanol-d_4

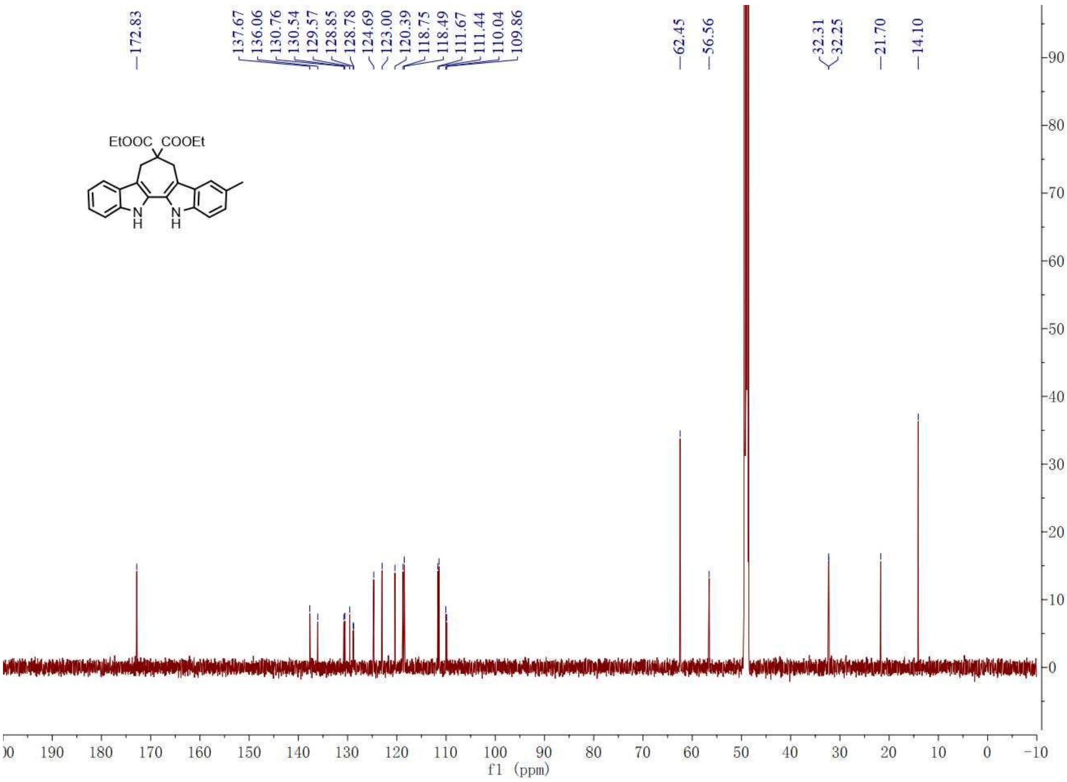

Figure S44. ^{13}C NMR spectrum of diethyl 3-methyl-5,7,12,13-tetrahydro-6*H*-cyclohepta[2,1-*b*:3,4-*b'*]diindole-6,6-dicarboxylate (**2b**) in Methanol-d_4

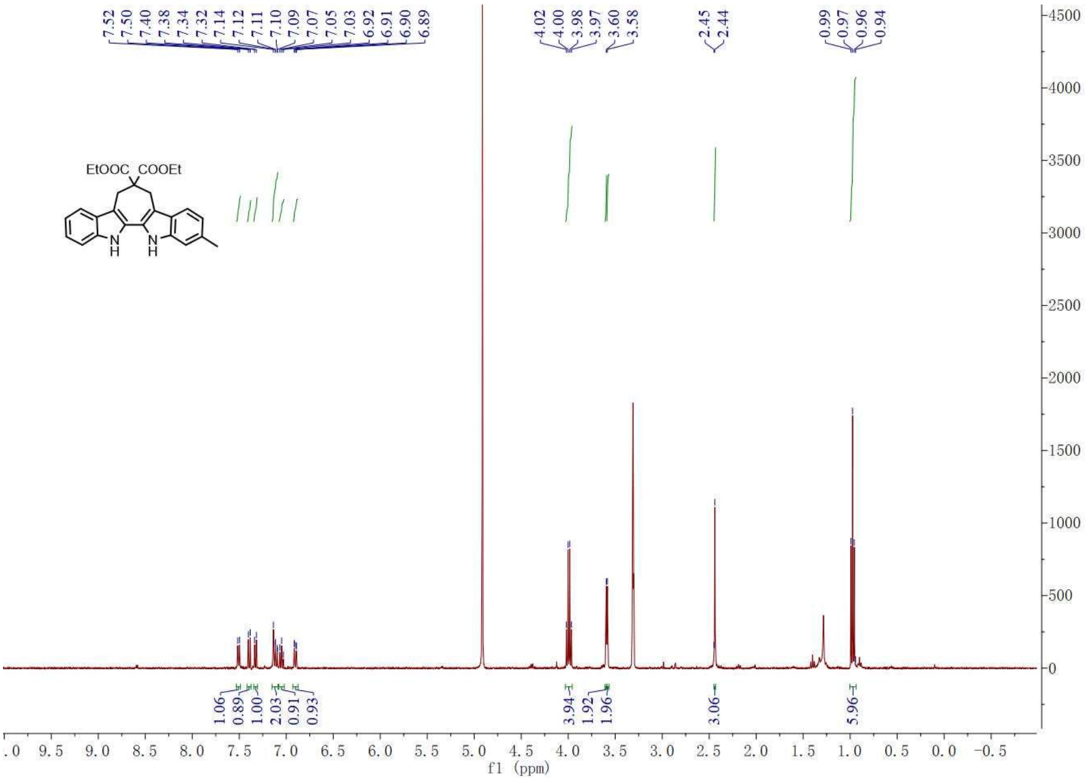

Figure S45. ^{1}H NMR spectrum of diethyl 2-methyl-5,7,12,13-tetrahydro-6*H*-cyclohepta[2,1-*b*:3,4-*b'*]diindole-6,6-dicarboxylate (**2c**) in Methanol-d_4

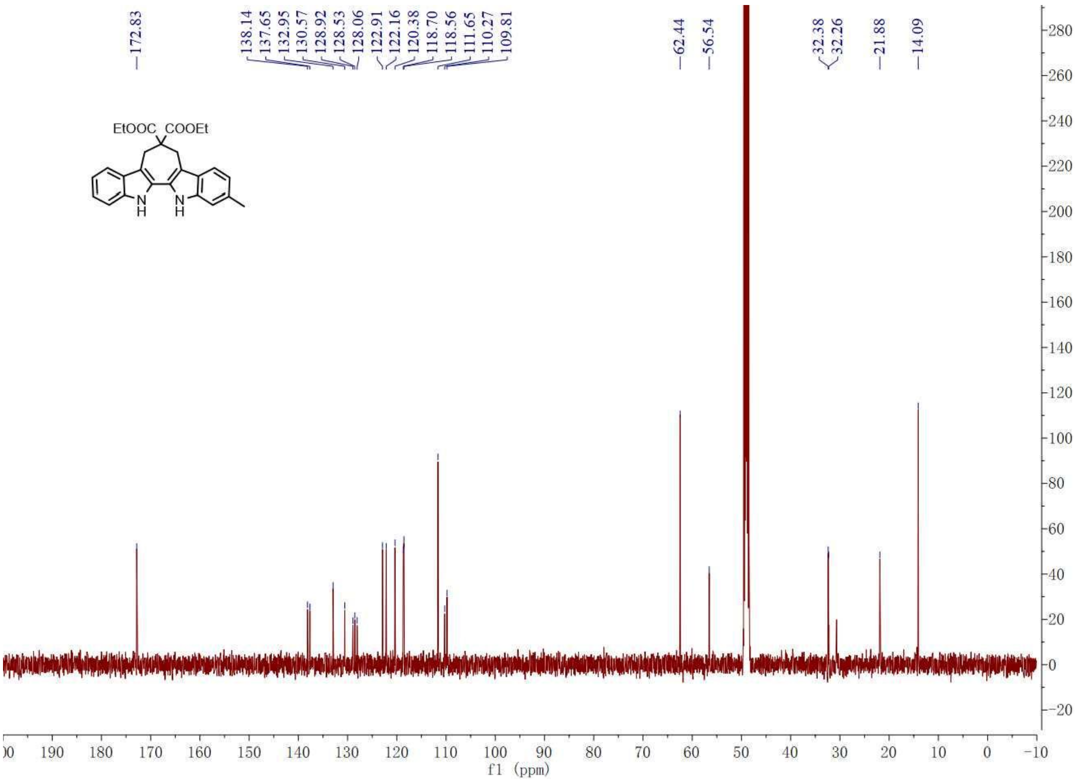

Figure S46. ^{13}C NMR spectrum of diethyl 2-methyl-5,7,12,13-tetrahydro-6*H*-cyclohepta[2,1-*b*:3,4-*b'*]diindole-6,6-dicarboxylate (**2c**) in Methanol-d_4

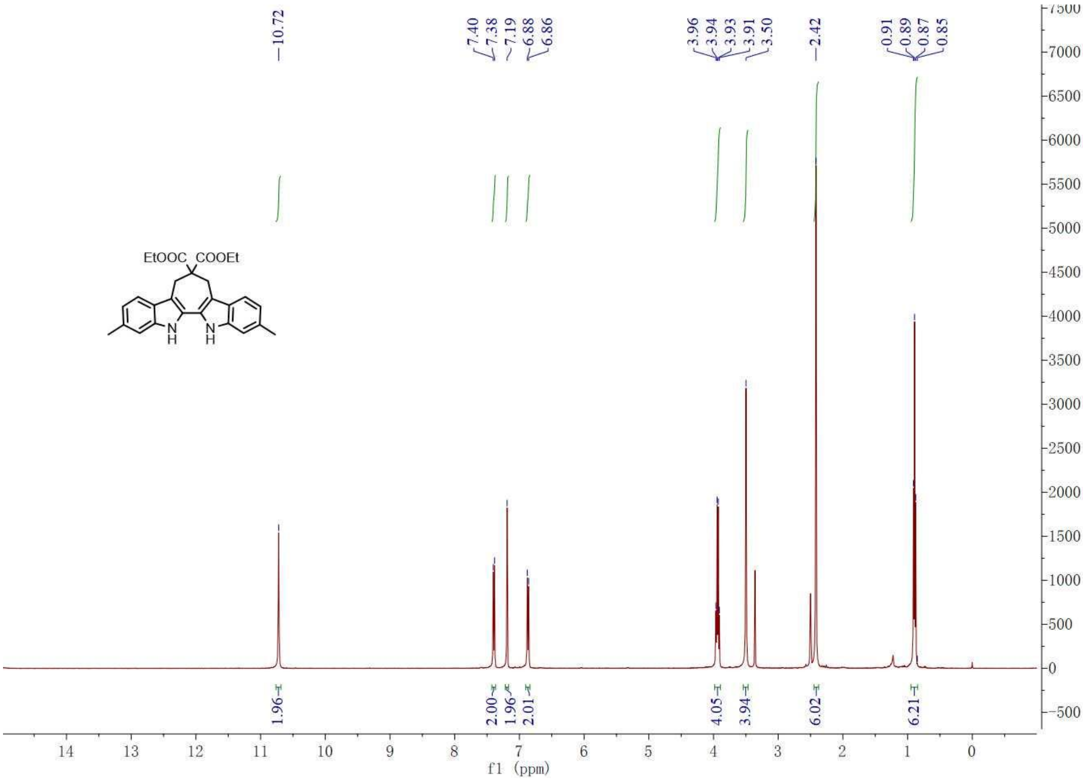

Figure S47. ^{1}H NMR spectrum of diethyl 2,10-dimethyl-5,7,12,13-tetrahydro-6*H*-cyclohepta[2,1-*b*:3,4-*b'*]diindole-6,6-di carboxylate (**2d**) in DMSO-d_6

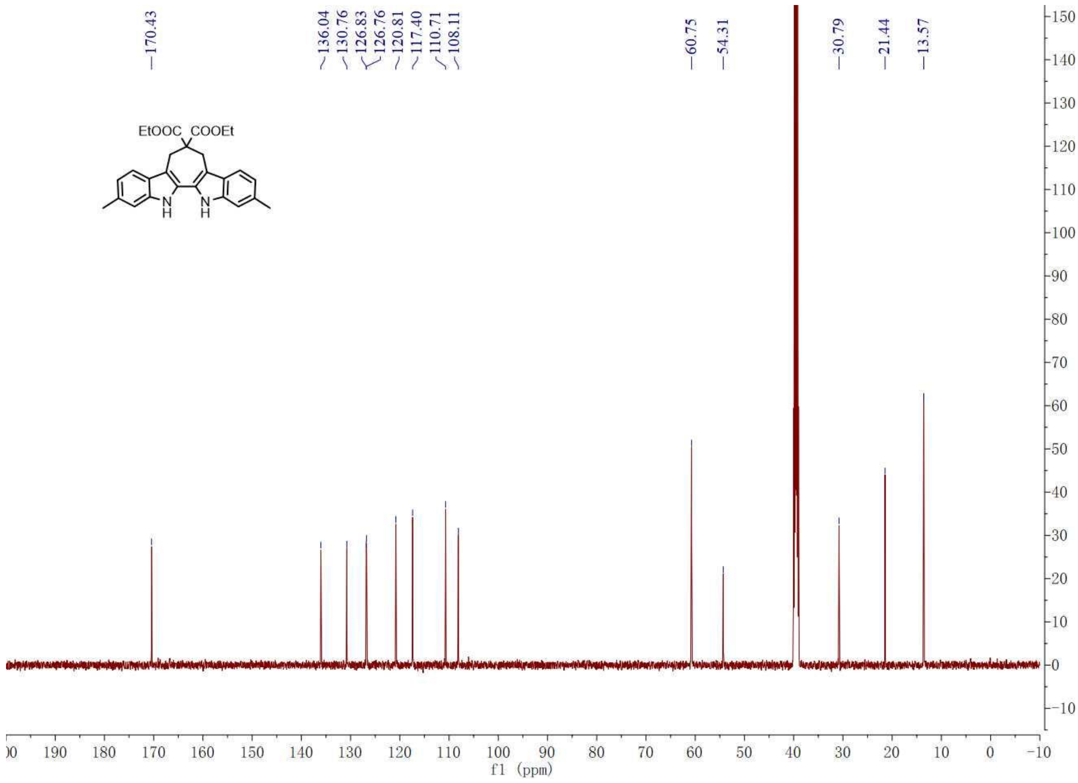

Figure S48. ^{13}C NMR spectrum of diethyl 2,10-dimethyl-5,7,12,13-tetrahydro-6*H*-cyclohepta[2,1-*b*:3,4-*b'*]diindole-6,6-di carboxylate (**2d**) in DMSO-d_6

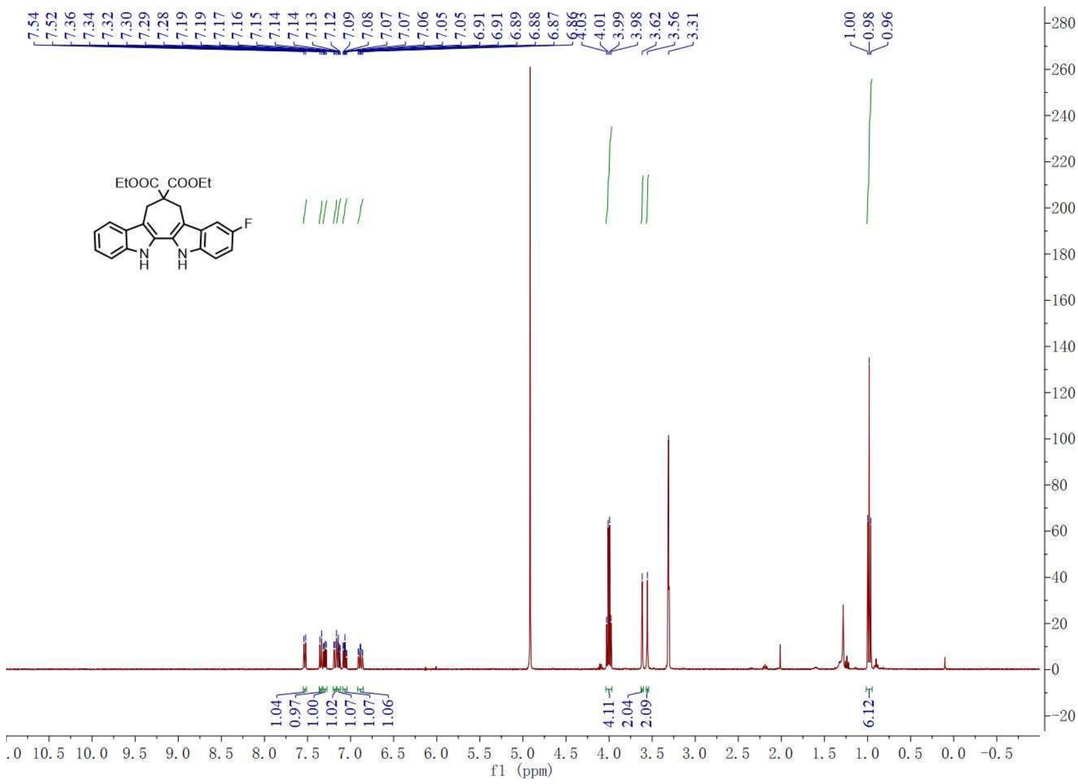

Figure S49. ^{1}H NMR spectrum of diethyl 3-fluoro-5,7,12,13-tetrahydro-6*H*-cyclohepta[2,1-*b*:3,4-*b'*]diindole-6,6-dicarboxylate (**2e**) in Methanol-d_4

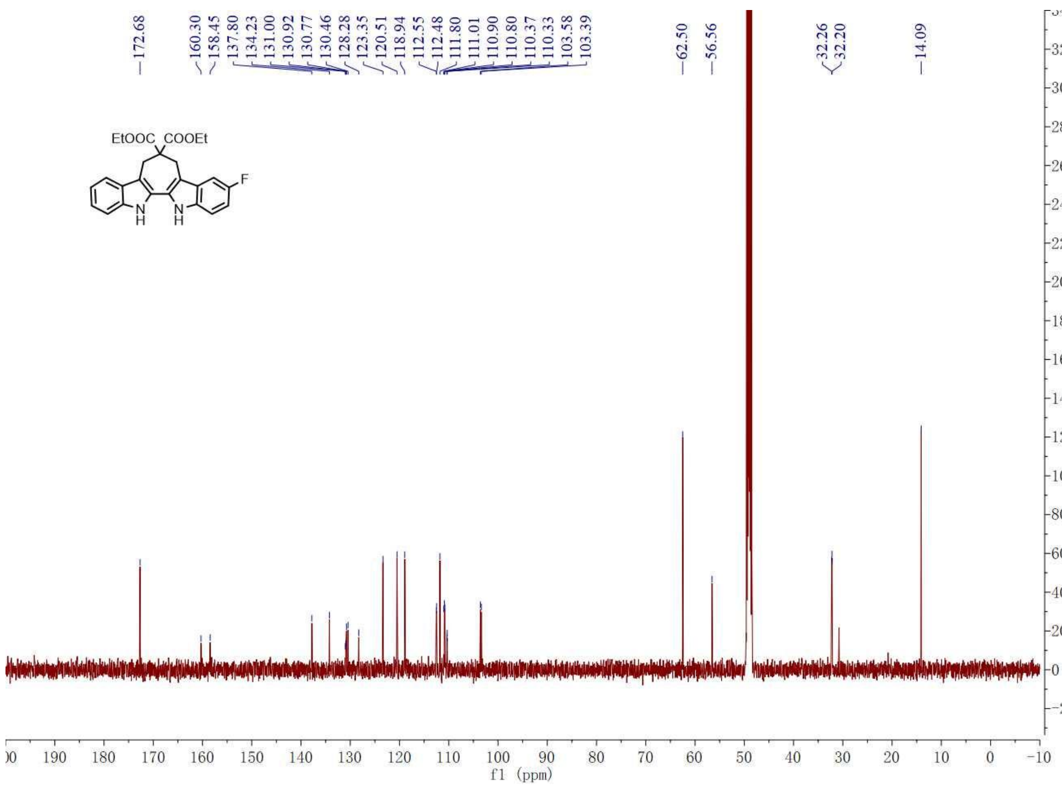

Figure S50. ^{13}C NMR spectrum of diethyl 3-fluoro-5,7,12,13-tetrahydro-6*H*-cyclohepta[2,1-*b*:3,4-*b'*]diindole-6,6-dicarboxylate (**2e**) in Methanol-d_4

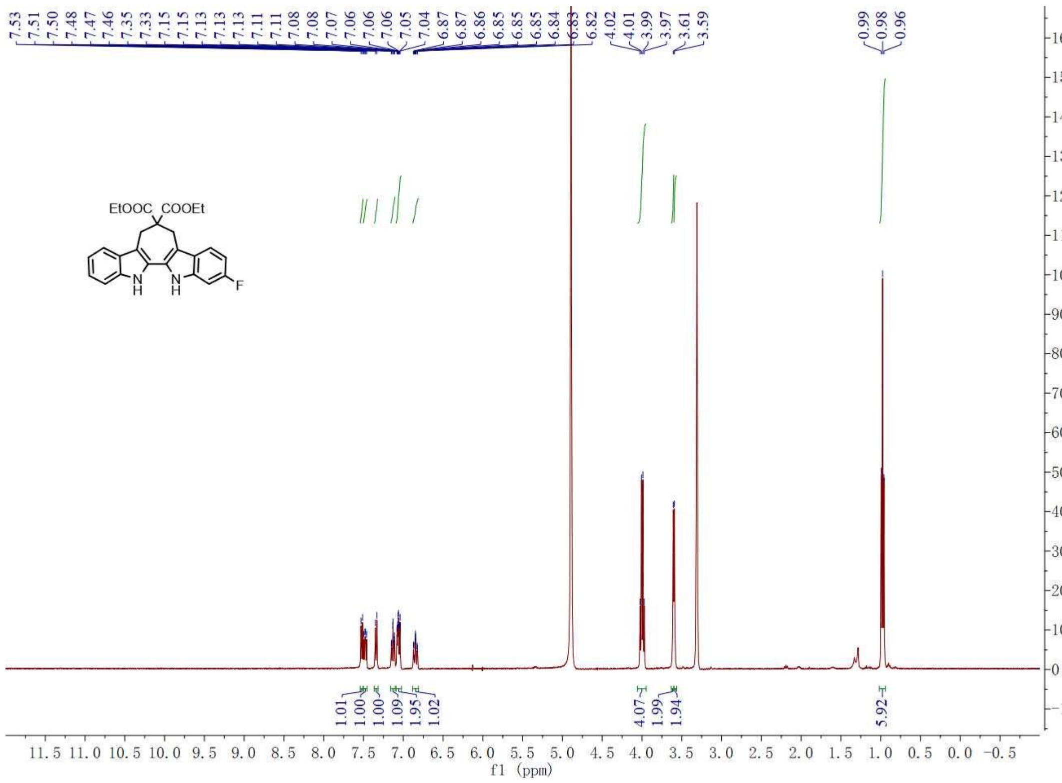

Figure S51. ^{1}H NMR spectrum of diethyl 2-fluoro-5,7,12,13-tetrahydro-6*H*-cyclohepta[2,1-*b*:3,4-*b'*]diindole-6,6-dicarboxylate (**2f**) in Methanol-d_4

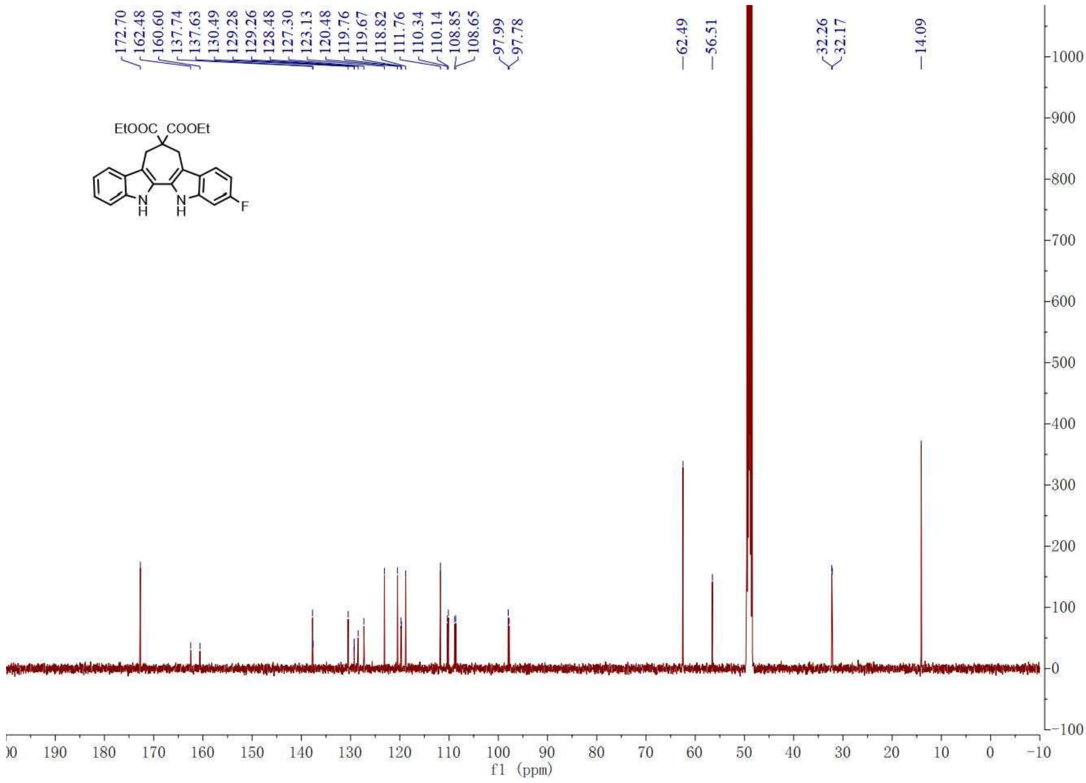

Figure S52. ^{13}C NMR spectrum of diethyl 2-fluoro-5,7,12,13-tetrahydro-6*H*-cyclohepta[2,1-*b*:3,4-*b'*]diindole-6,6-dicarboxylate (**2f**) in Methanol-d_4

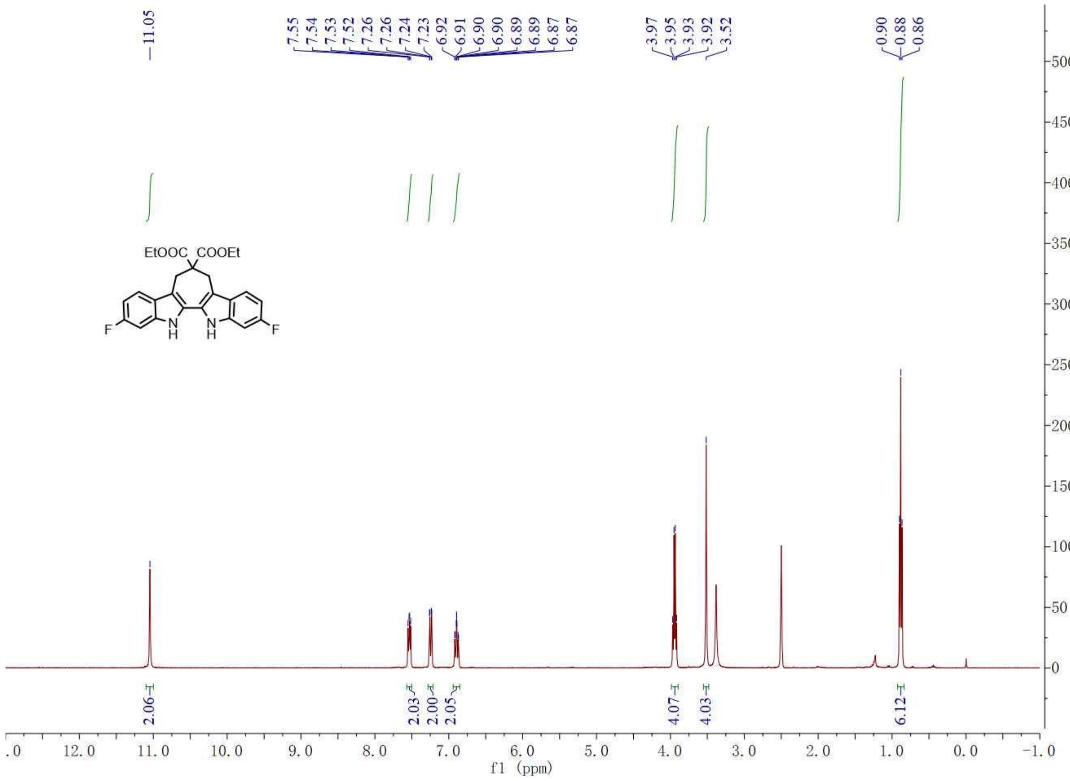

Figure S53. ^{1}H NMR spectrum of diethyl 2,10-difluoro-5,7,12,13-tetrahydro-6*H*-cyclohepta[2,1-*b*:3,4-*b'*]diindole-6,6-dicarboxylate (**2g**) in DMSO-d_6

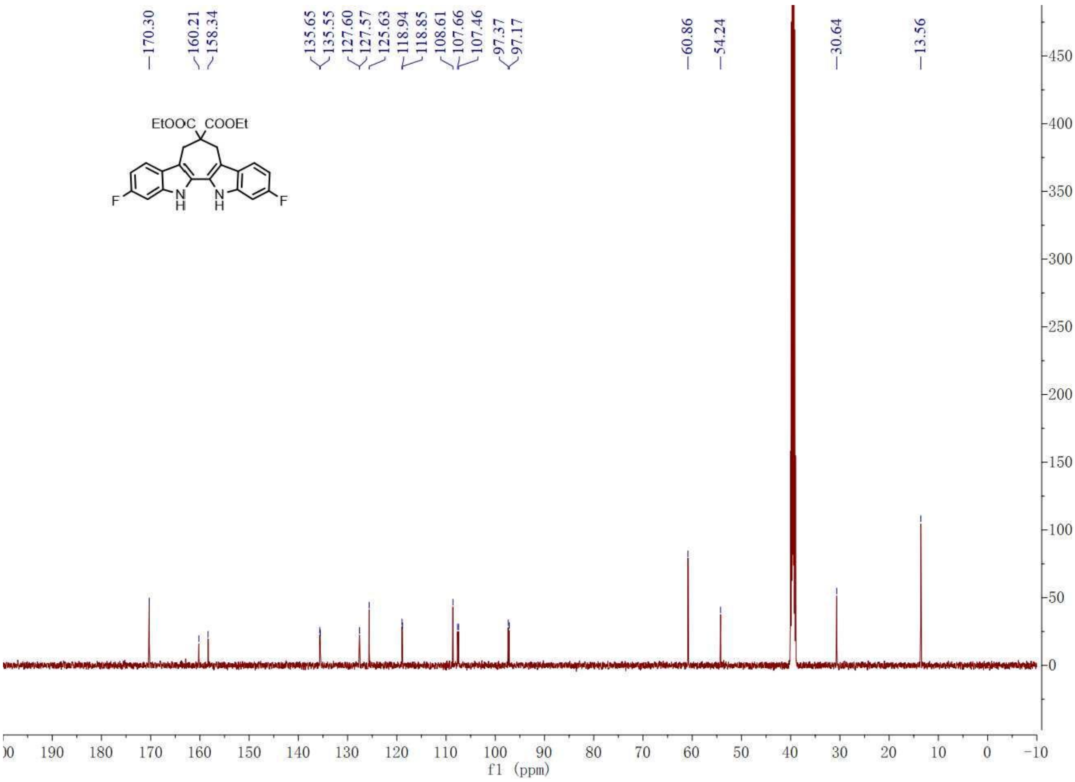

Figure S54. ^{13}C NMR spectrum of diethyl 2,10-difluoro-5,7,12,13-tetrahydro-6*H*-cyclohepta[2,1-*b*:3,4-*b'*]diindole-6,6-dicarboxylate (**2g**) in DMSO-d_6

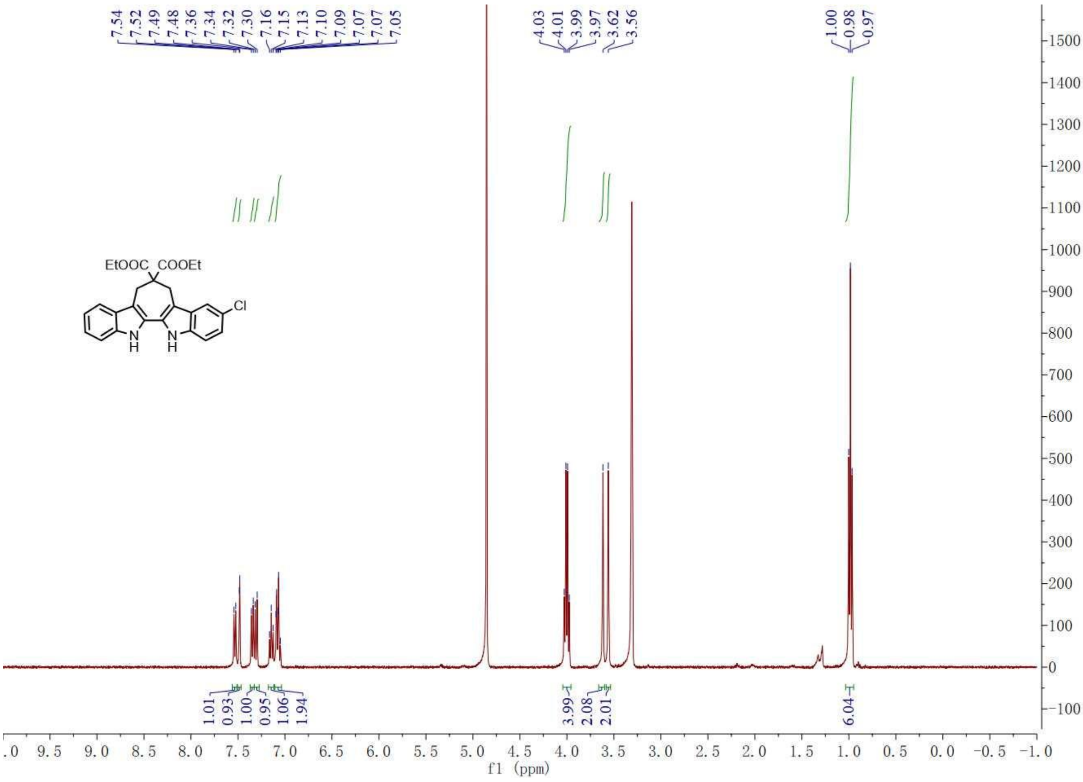

Figure S55. ^{1}H NMR spectrum of diethyl 3-chloro-5,7,12,13-tetrahydro-6*H*-cyclohepta[2,1-*b*:3,4-*b'*]diindole-6,6-dicarboxylate (**2h**) in Methanol-d_4

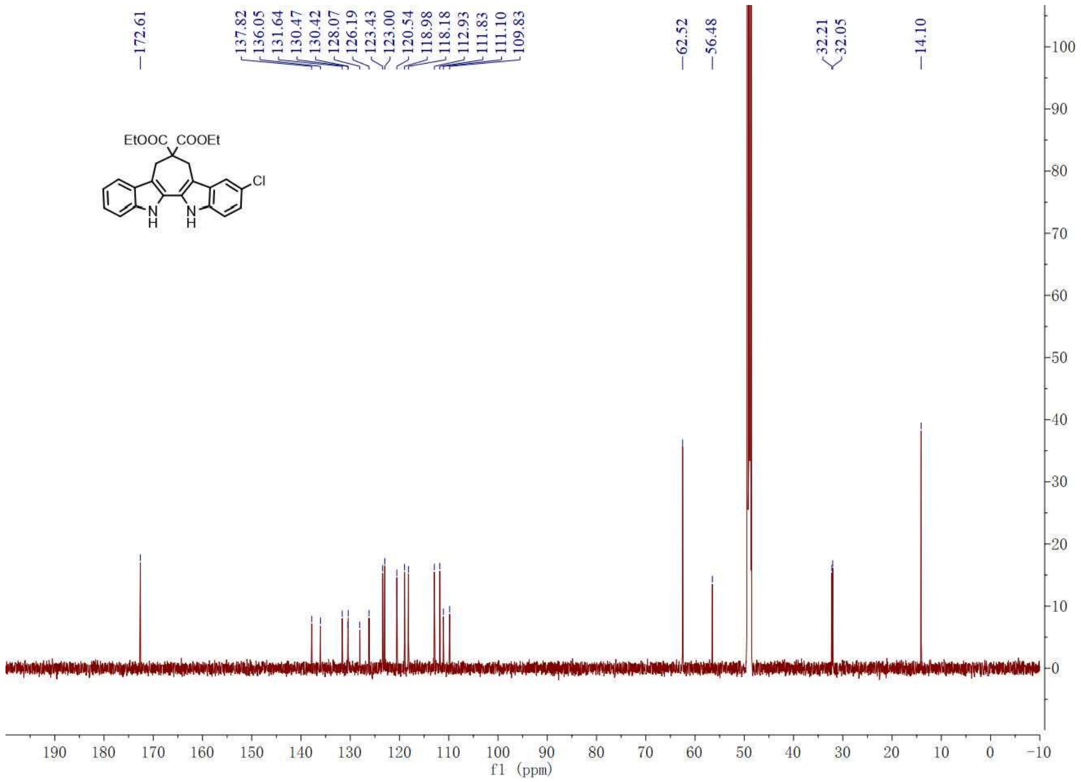

Figure S56. ^{13}C NMR spectrum of diethyl 3-chloro-5,7,12,13-tetrahydro-6*H*-cyclohepta[2,1-*b*:3,4-*b'*]diindole-6,6-dicarboxylate (**2h**) in Methanol-d_4

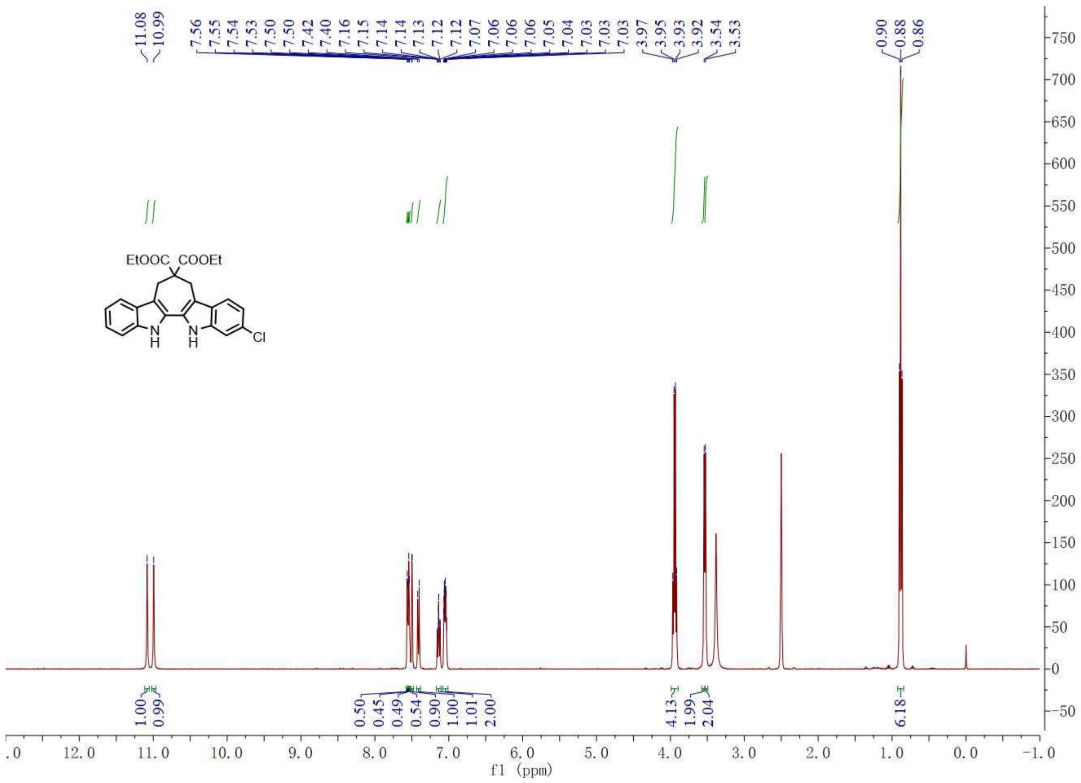

Figure S57. ^{1}H NMR spectrum of diethyl 2-chloro-5,7,12,13-tetrahydro-6*H*-cyclohepta[2,1-*b*:3,4-*b'*]diindole-6,6-dicarboxylate (**2i**) in DMSO-d_6

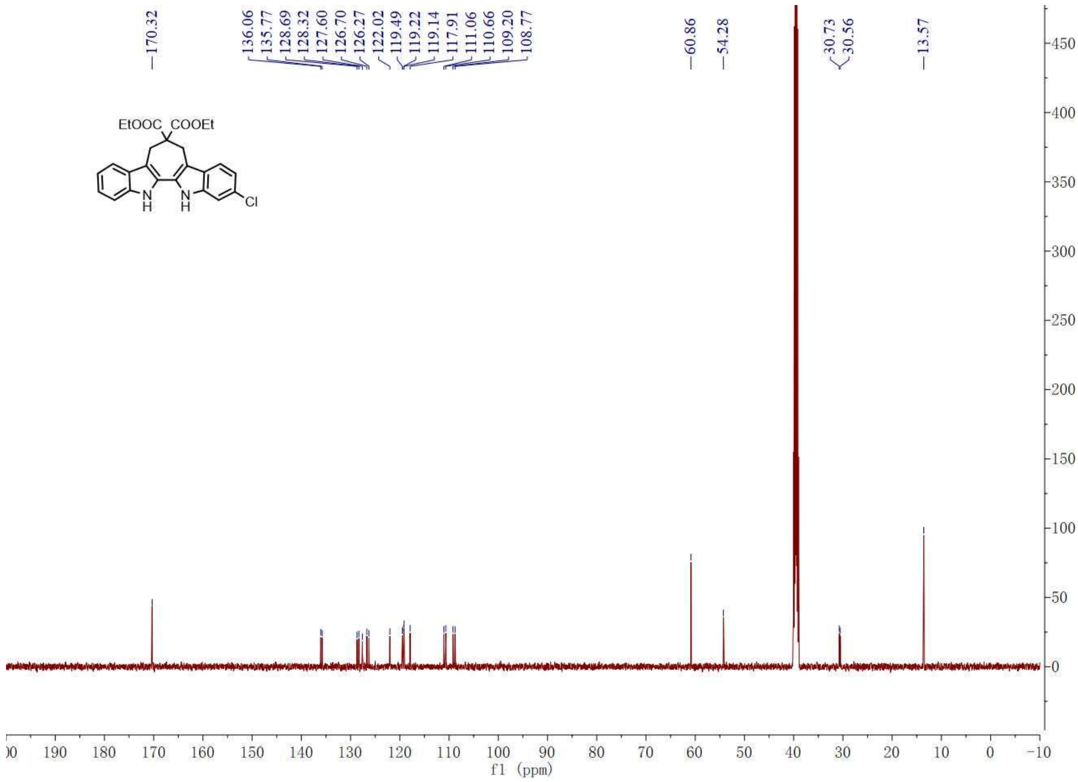

Figure S58. ^{13}C NMR spectrum of diethyl 2-chloro-5,7,12,13-tetrahydro-6*H*-cyclohepta[2,1-*b*:3,4-*b'*]diindole-6,6-dicarboxylate (**2i**) in DMSO-d_6

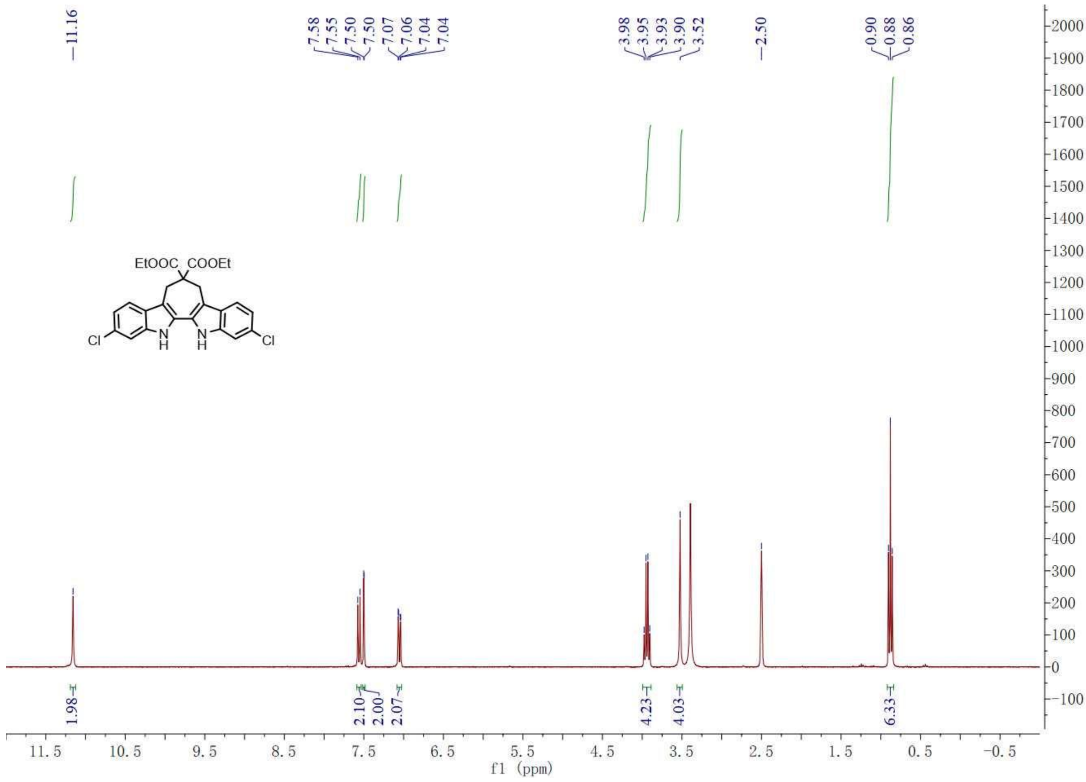

Figure S59. ^{1}H NMR spectrum of diethyl 2,10-dichloro-5,7,12,13-tetrahydro-6*H*-cyclohepta[2,1-*b*:3,4-*b'*]diindole-6,6-di carboxylate (**2j**) in DMSO-d_6

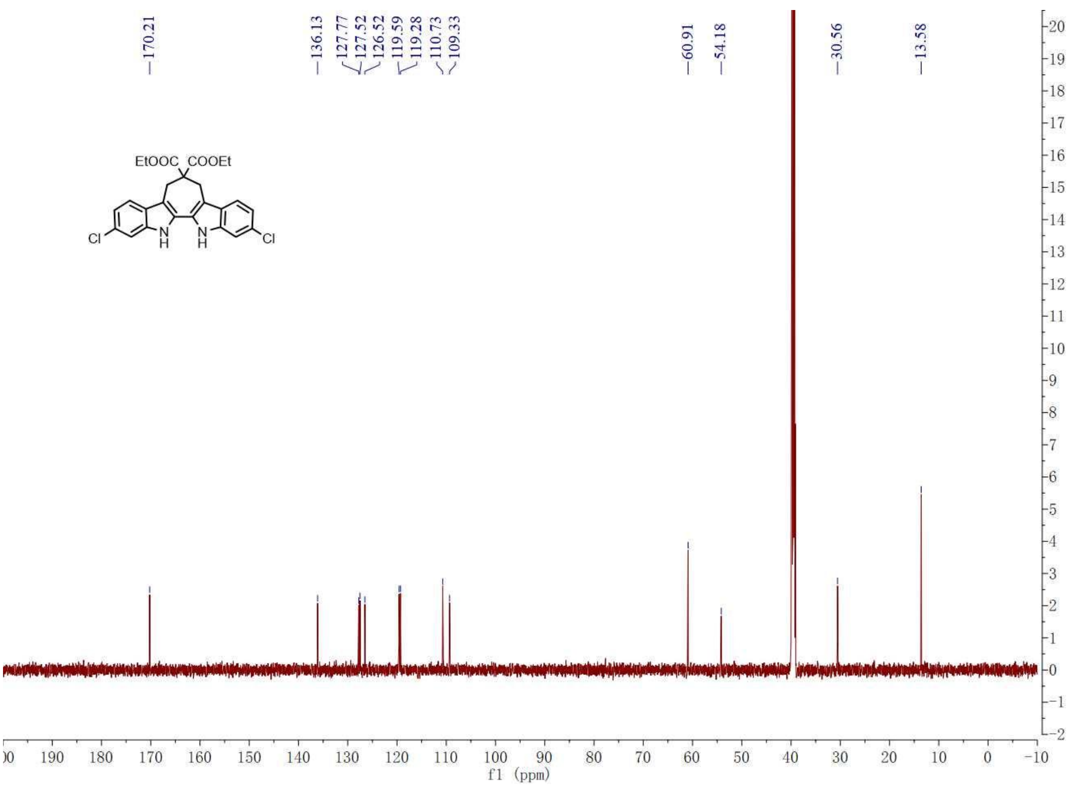

Figure S60. ^{13}C NMR spectrum of diethyl 2,10-dichloro-5,7,12,13-tetrahydro-6*H*-cyclohepta[2,1-*b*:3,4-*b'*]diindole-6,6-di carboxylate (**2j**) in DMSO-d_6

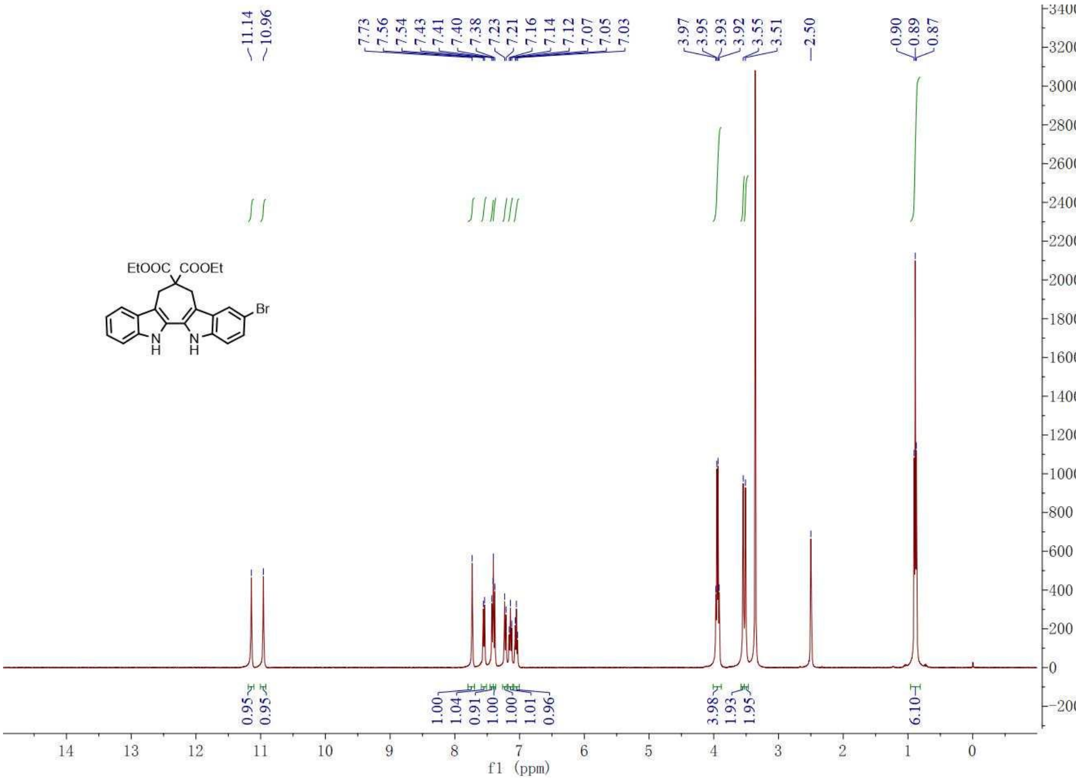

Figure S61. ^{1}H NMR spectrum of diethyl 3-bromo-5,7,12,13-tetrahydro-6*H*-cyclohepta[2,1-*b*:3,4-*b'*]diindole-6,6-dicarboxylate (**2k**) in DMSO-d_6

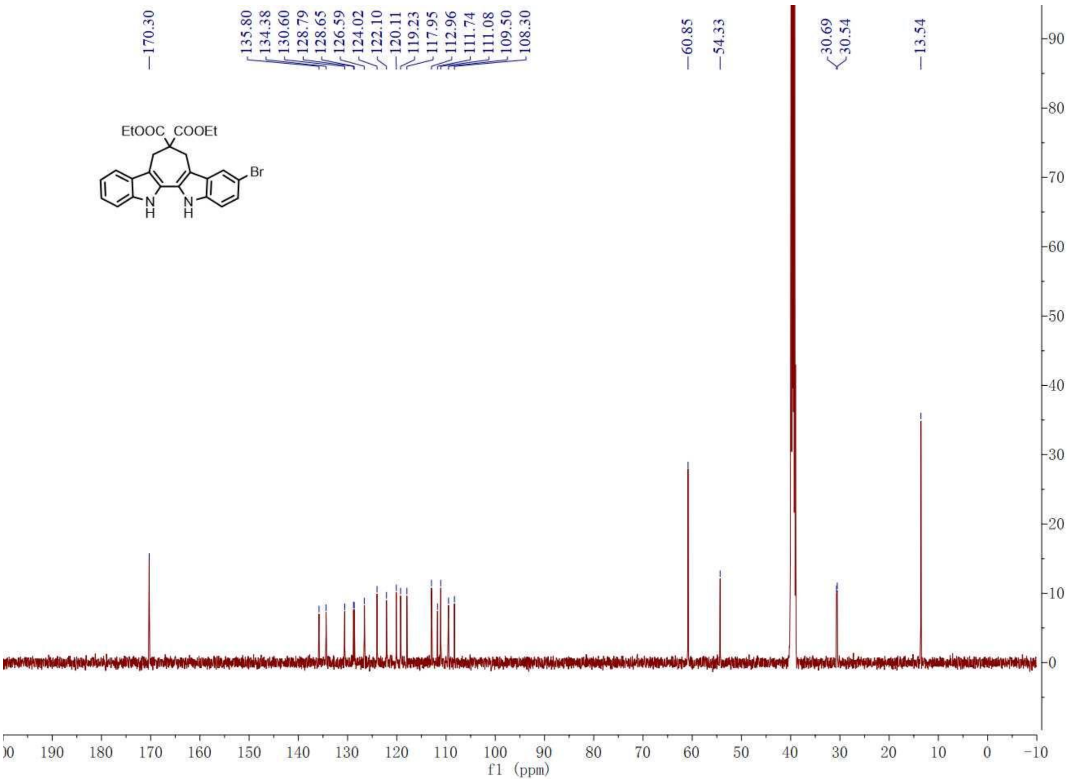

Figure S62. ^{13}C NMR spectrum of diethyl 3-bromo-5,7,12,13-tetrahydro-6*H*-cyclohepta[2,1-*b*:3,4-*b'*]diindole-6,6-dicarboxylate (**2k**) in DMSO-d_6

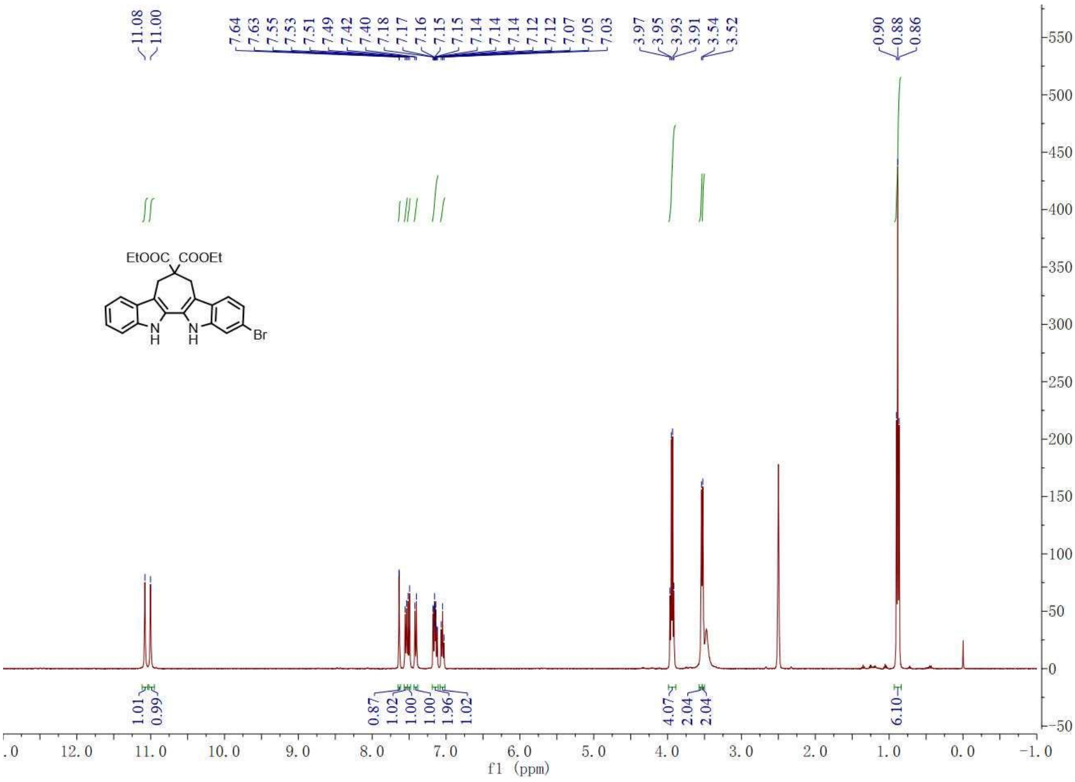

Figure S63. ^{1}H NMR spectrum of diethyl 2-bromo-5,7,12,13-tetrahydro-6*H*-cyclohepta[2,1-*b*:3,4-*b'*]diindole-6,6-dicarboxylate (**2l**) in DMSO-d_6

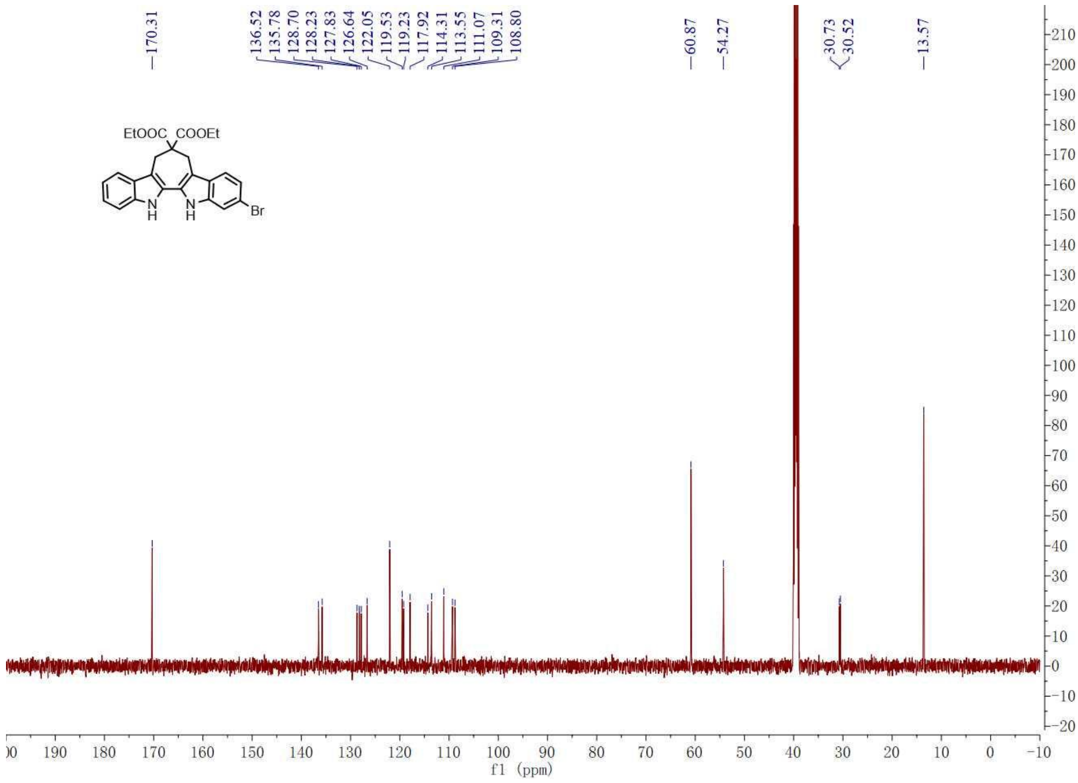

Figure S64. ^{13}C NMR spectrum of diethyl 2-bromo-5,7,12,13-tetrahydro-6*H*-cyclohepta[2,1-*b*:3,4-*b*′]diindole-6,6-dicarboxylate (**2l**) in DMSO-d_6

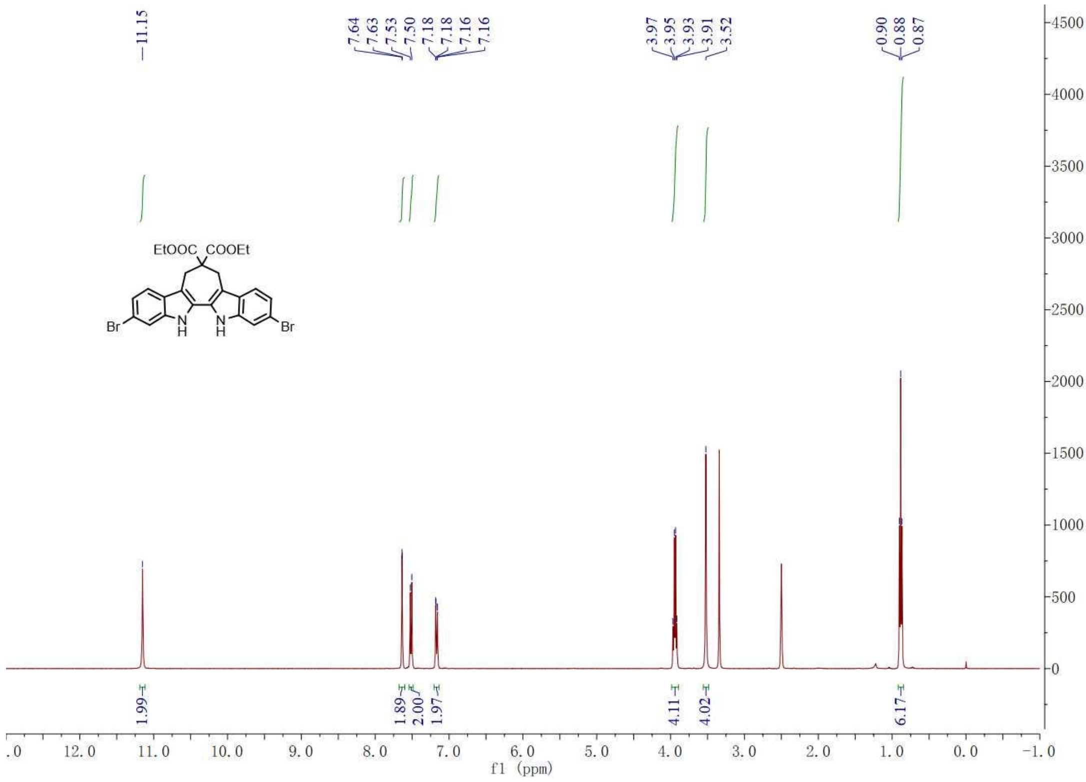

Figure S65. ^{1}H NMR spectrum of diethyl 2,10-dibromo-5,7,12,13-tetrahydro-6*H*-cyclohepta[2,1-*b*:3,4-*b*′]diindole-6,6-di carboxylate (**2m**) in DMSO-d_6

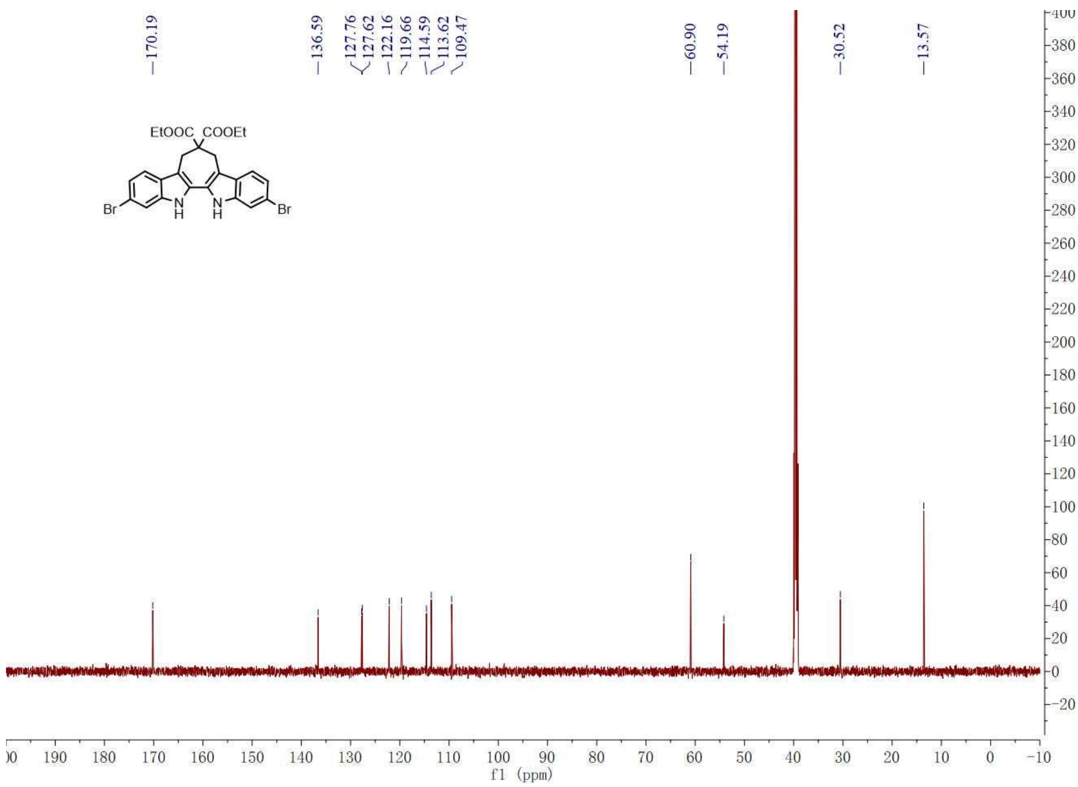

Figure S66. ^{13}C NMR spectrum of diethyl 2,10-dibromo-5,7,12,13-tetrahydro-6*H*-cyclohepta[2,1-*b*:3,4-*b'*]diindole-6,6-di carboxylate (**2m**) in DMSO-d_6

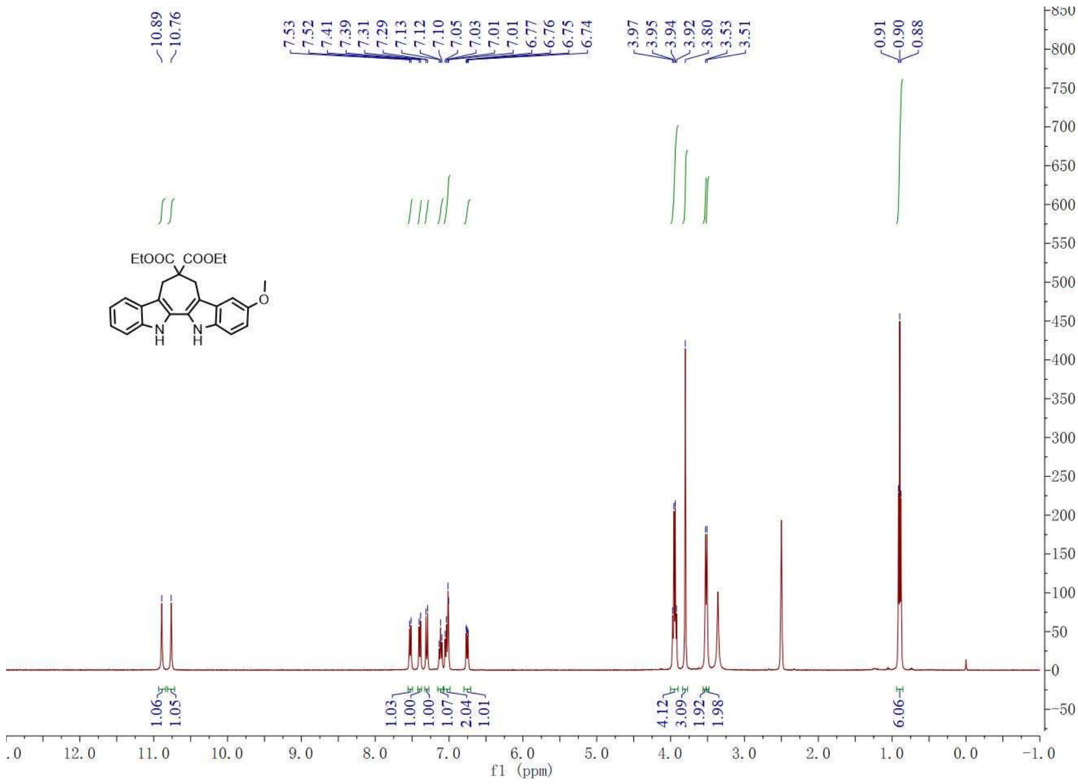

Figure S67. ^{1}H NMR spectrum of diethyl 3-methoxy-5,7,12,13-tetrahydro-6*H*-cyclohepta[2,1-*b*:3,4-*b'*]diindole-6,6-dicarboxylate (**2n**) in DMSO-d_6

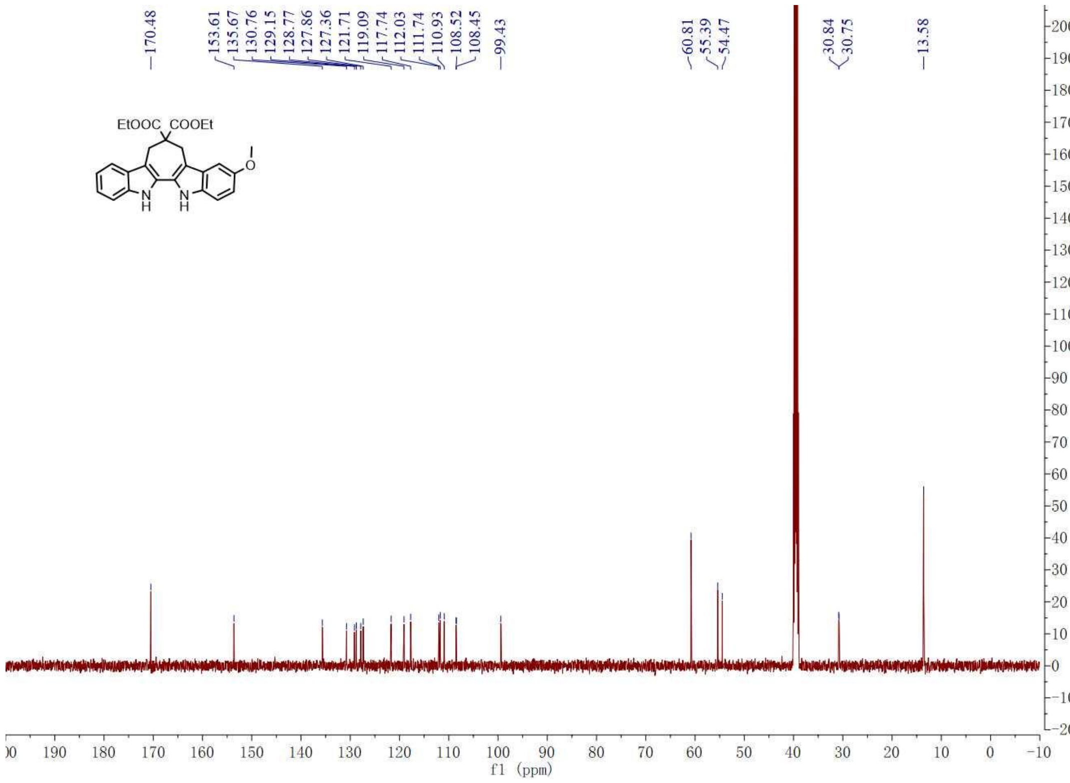

Figure S68. ^{13}C NMR spectrum of diethyl 3-methoxy-5,7,12,13-tetrahydro-6*H*-cyclohepta[2,1-*b*:3,4-*b'*]diindole-6,6-dicarboxylate (**2n**) in DMSO-d_6

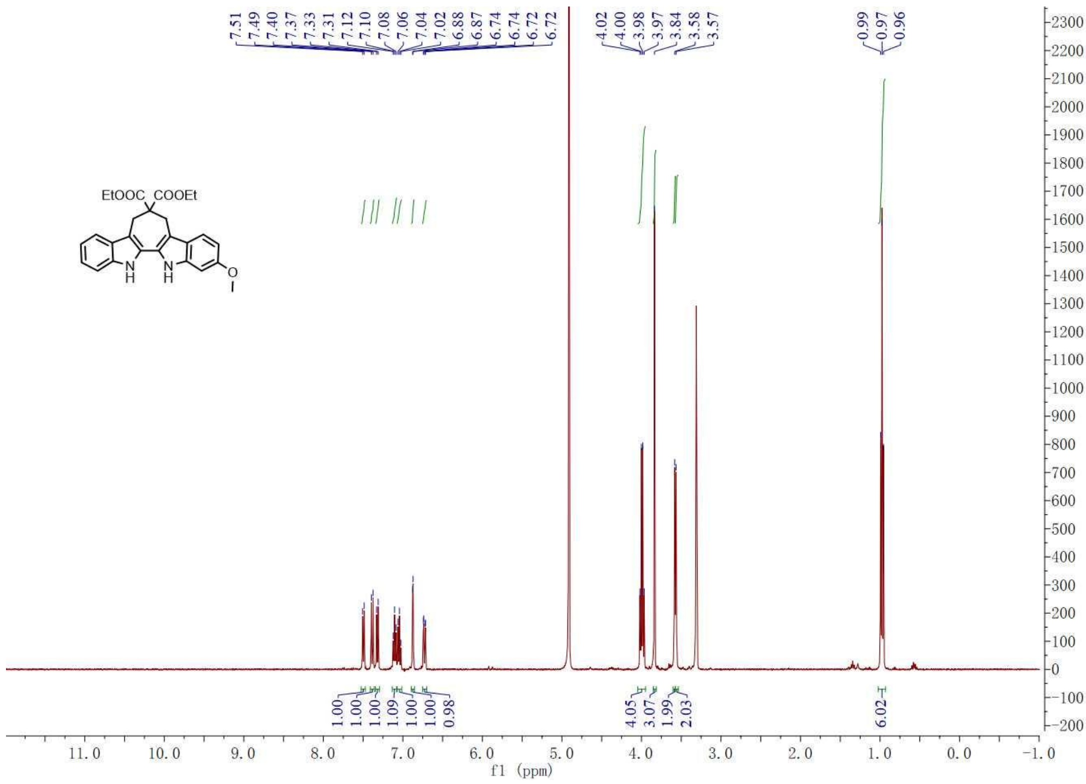

Figure S69. ^{1}H NMR spectrum of diethyl 2-methoxy-5,7,12,13-tetrahydro-6*H*-cyclohepta[2,1-*b*:3,4-*b'*]diindole-6,6-dicarboxylate (**2o**) in Methanol-d_4

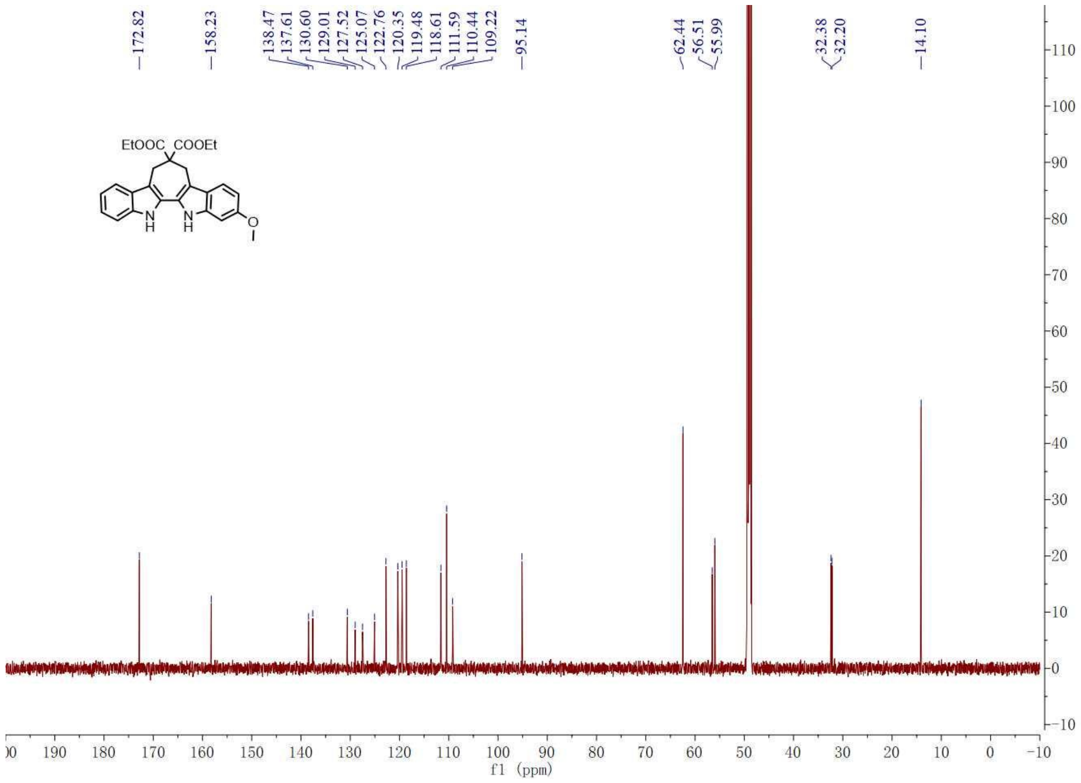

Figure S70. ^{13}C NMR spectrum of diethyl 2-methoxy-5,7,12,13-tetrahydro-6*H*-cyclohepta[2,1-*b*:3,4-*b'*]diindole-6,6-dicarboxylate (**2o**) in Methanol-d_4

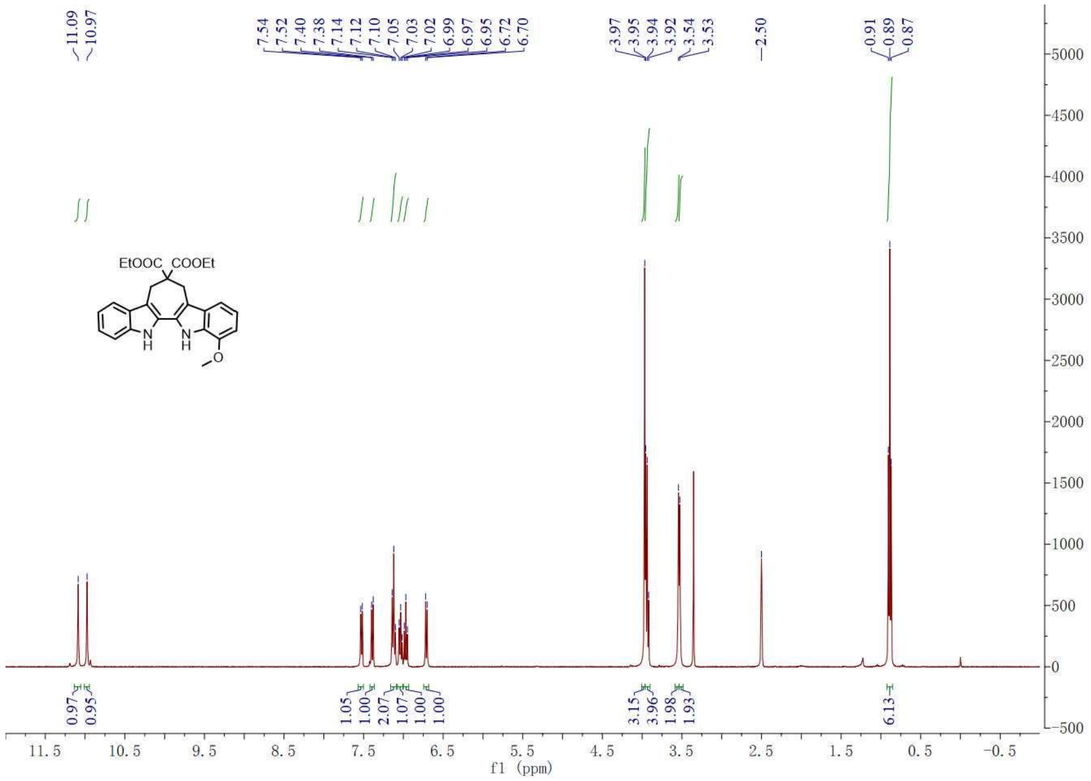

Figure S71. ^{1}H NMR spectrum of diethyl 1-methoxy-5,7,12,13-tetrahydro-6*H*-cyclohepta[2,1-*b*:3,4-*b'*]diindole-6,6-dicarboxylate (**2p**) in DMSO-d_6

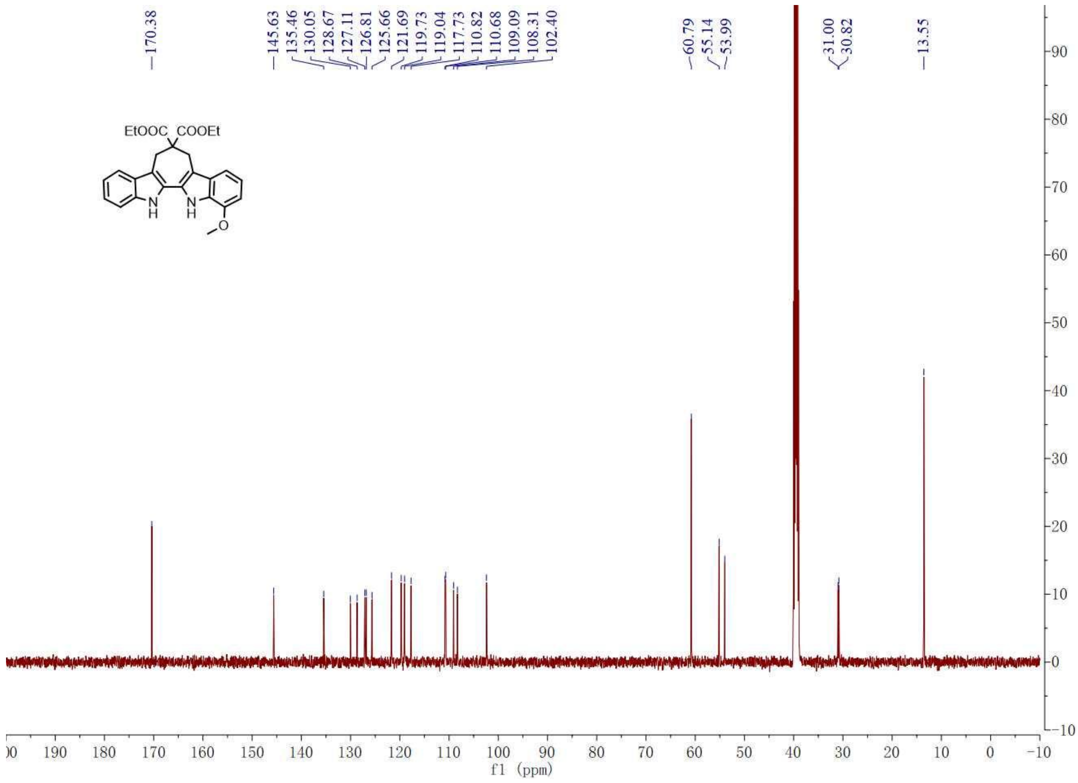

Figure S72. ^{13}C NMR spectrum of diethyl 1-methoxy-5,7,12,13-tetrahydro-6*H*-cyclohepta[2,1-*b*:3,4-*b'*]diindole-6,6-dicarboxylate (**2p**) in DMSO-d_6

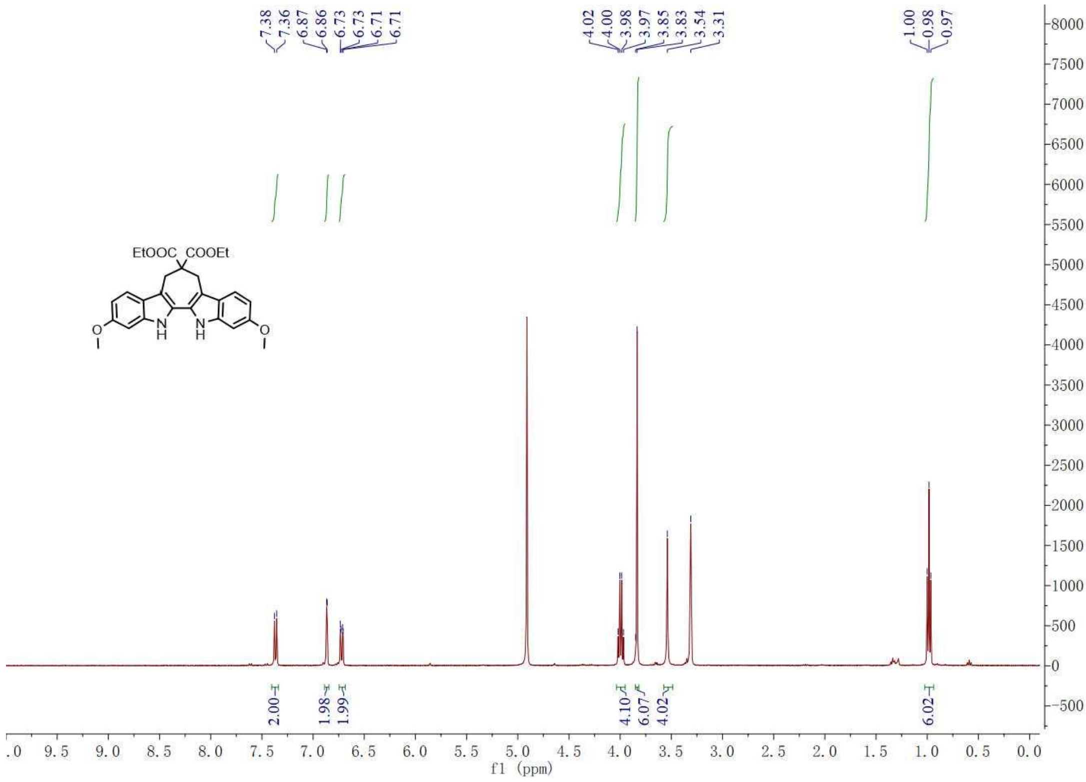

Figure S73. ^{1}H NMR spectrum of diethyl 2,10-dimethoxy-5,7,12,13-tetrahydro-6*H*-cyclohepta[2,1-*b*:3,4-*b'*]diindole-6,6-di carboxylate (**2q**) in Methanol-d_4

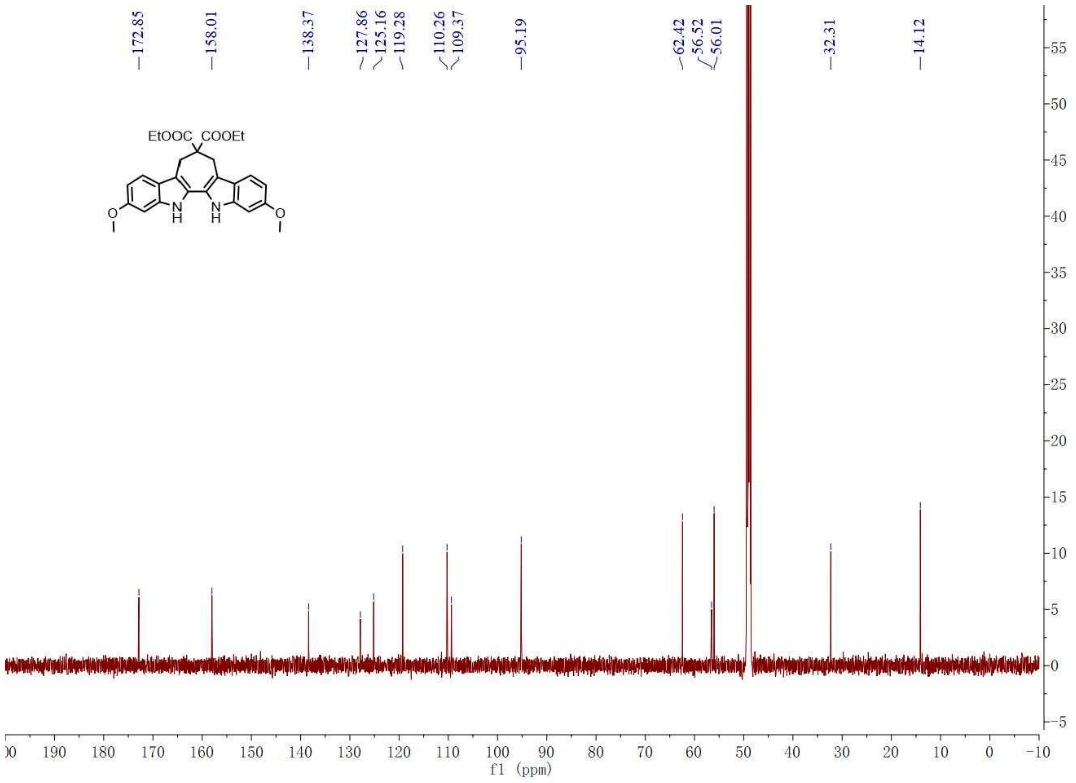

Figure S74. ^{13}C NMR spectrum of diethyl 2,10-dimethoxy-5,7,12,13-tetrahydro-6*H*-cyclohepta[2,1-*b*:3,4-*b'*]diindole-6,6-di carboxylate (**2q**) in Methanol-d_4

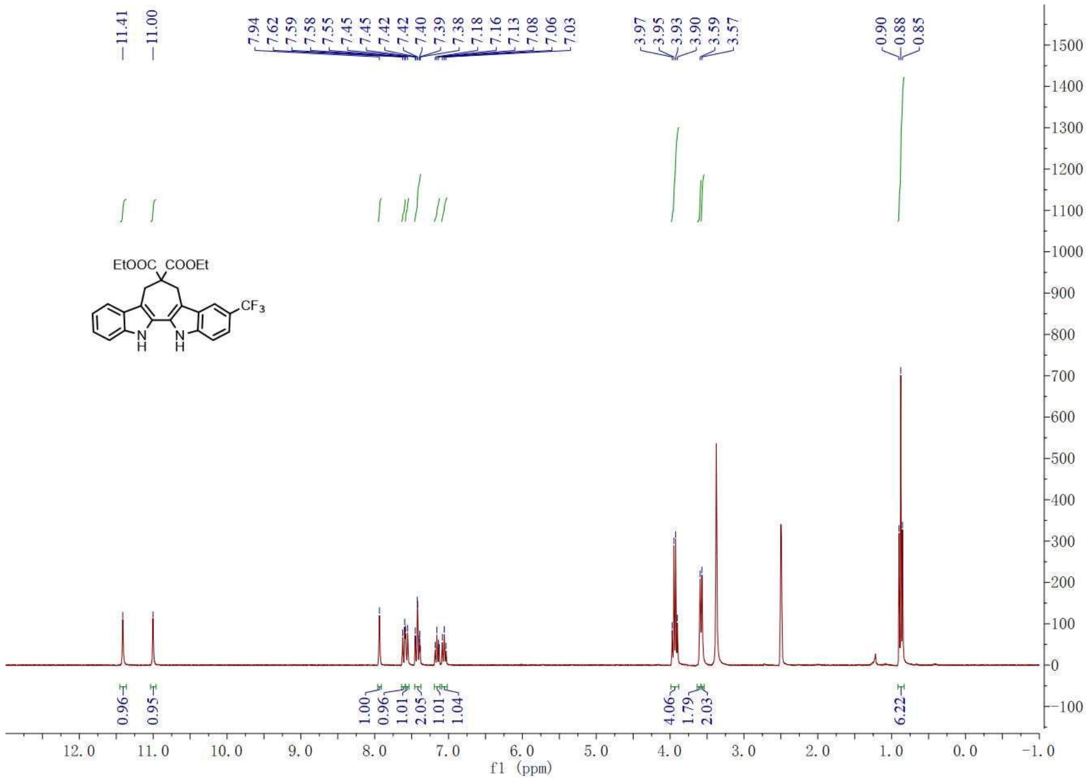

Figure S75. ^{1}H NMR spectrum of diethyl 3-(trifluoromethyl)-5,7,12,13-tetrahydro-6*H*-cyclohepta[2,1-*b*:3,4-*b'*]diindole-6,6-di carboxylate (**2r**) in DMSO-d_6

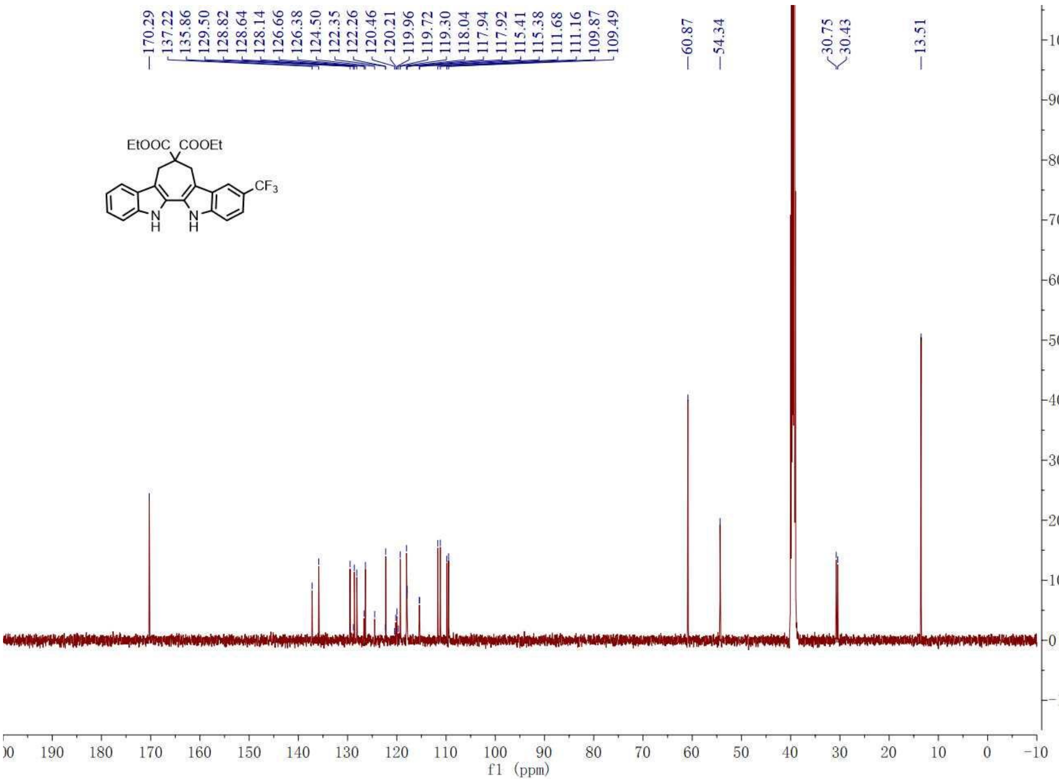

Figure S76. ^{13}C NMR spectrum of diethyl 3-(trifluoromethyl)-5,7,12,13-tetrahydro-6*H*-cyclohepta[2,1-*b*:3,4-*b'*]diindole-6,6-di carboxylate (**2r**) in DMSO-d_6

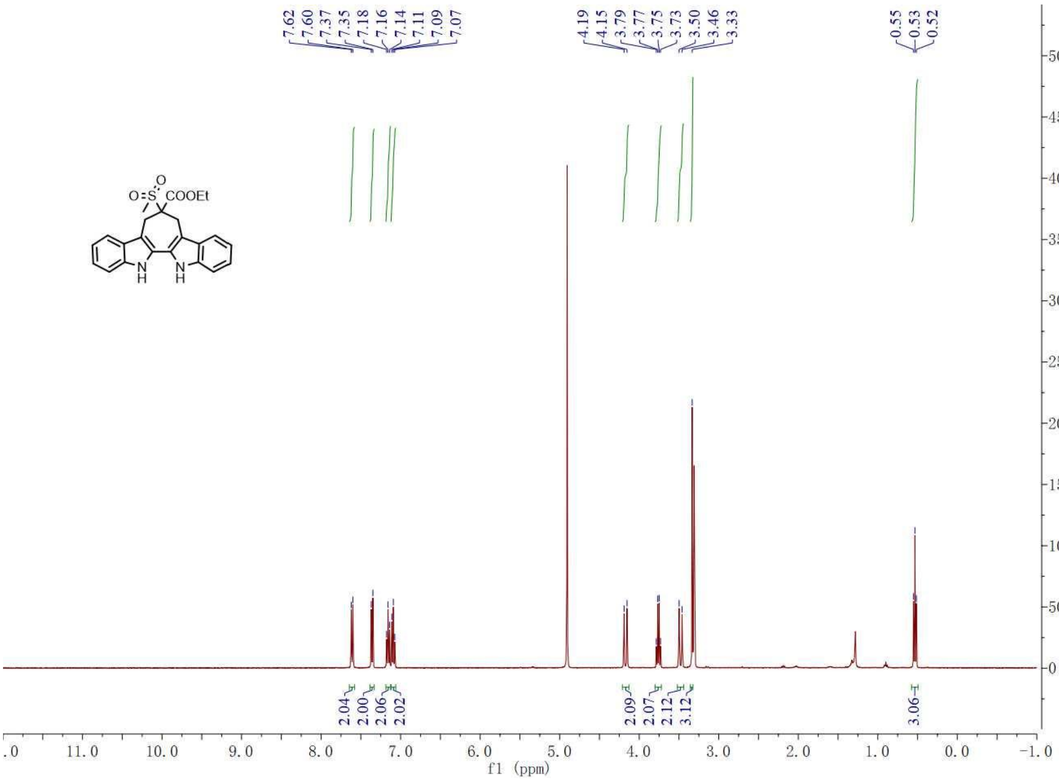

Figure S77. ^{1}H NMR spectrum of ethyl 6-(methylsulfonyl)-6,7,12,13-tetrahydro-5*H*-cyclohepta[2,1-*b*:3,4-*b'*]diindole-6-carboxylate (**2s**) in Methanol-d_4

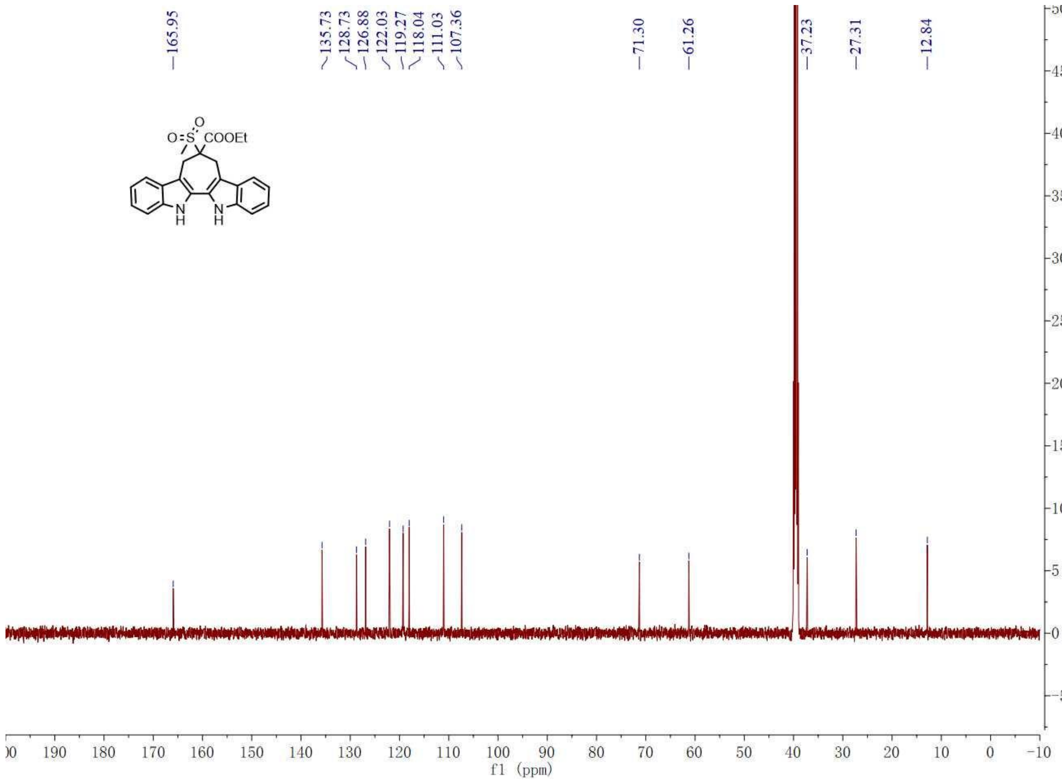

Figure S78. ^{13}C NMR spectrum of ethyl 6-(methylsulfonyl)-6,7,12,13-tetrahydro-5*H*-cyclohepta[2,1-*b*:3,4-*b'*]diindole-6-carboxylate (**2s**) in DMSO-d_6

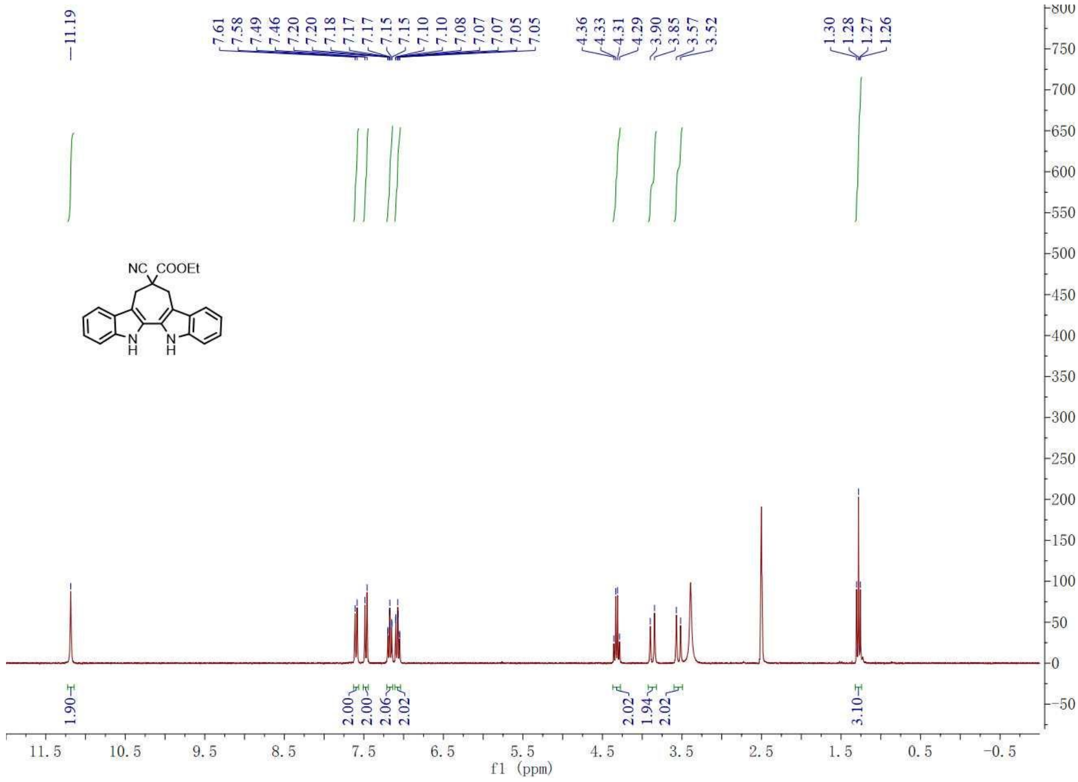

Figure S79. ^{1}H NMR spectrum of ethyl 6-cyano-6,7,12,13-tetrahydro-5*H*-cyclohepta[2,1-*b*:3,4-*b'*]diindole-6-carboxylate (**2t**) in DMSO-d_6

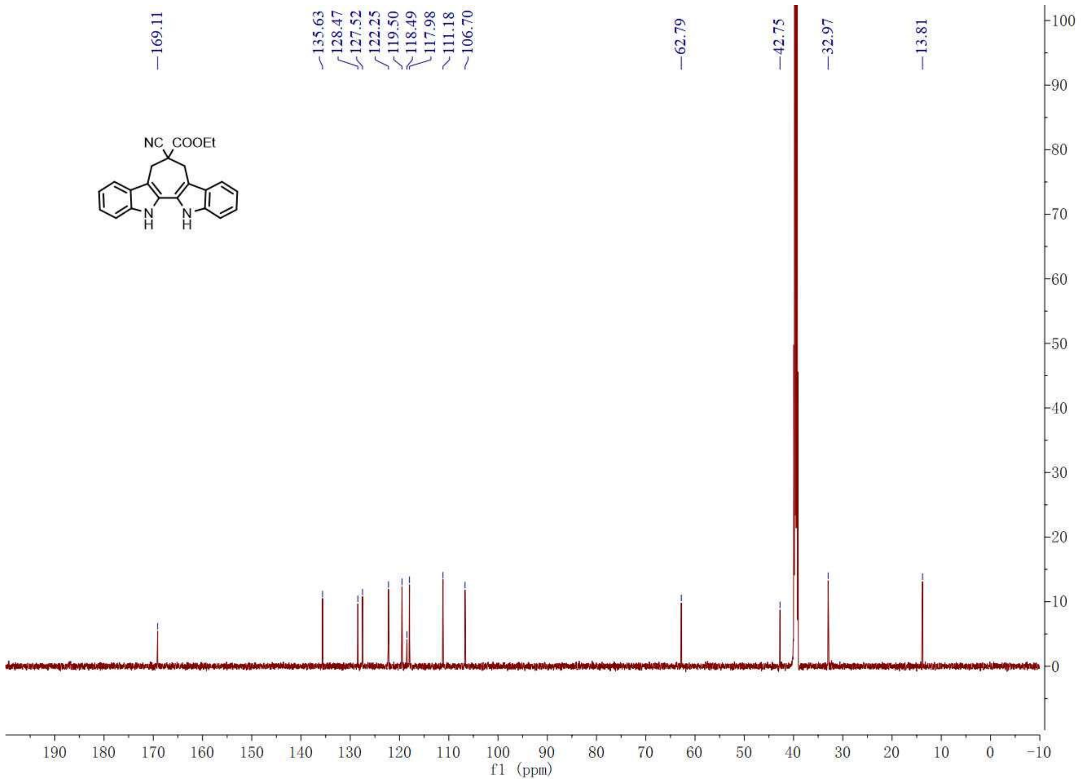

Figure S80. ^{13}C NMR spectrum of ethyl 6-cyano-6,7,12,13-tetrahydro-5*H*-cyclohepta[2,1-*b*:3,4-*b'*]diindole-6-carboxylate (**2t**) in DMSO-d_6

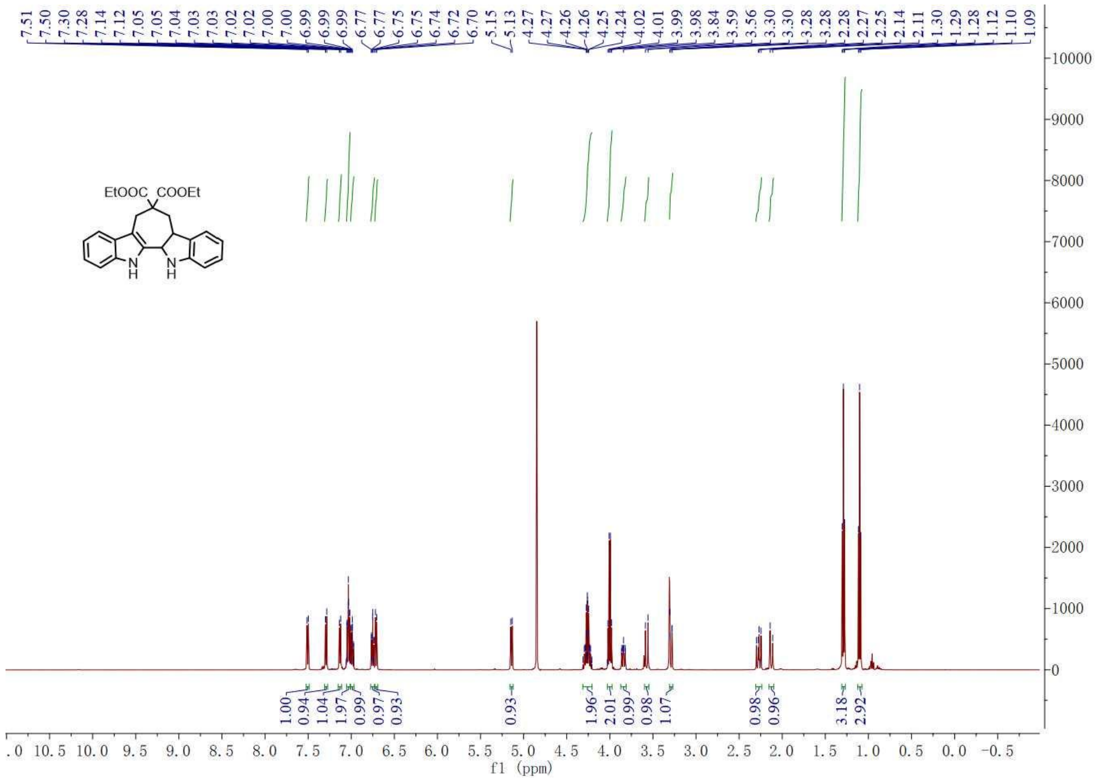

Figure S81. ^{1}H NMR spectrum of diethyl 4b,5,7,12,12b,13-hexahydro-6*H*-cyclohepta[2,1-*b*:3,4-*b'*]diindole-6,6-dicarboxylate (**3a**) in Methanol-d_4

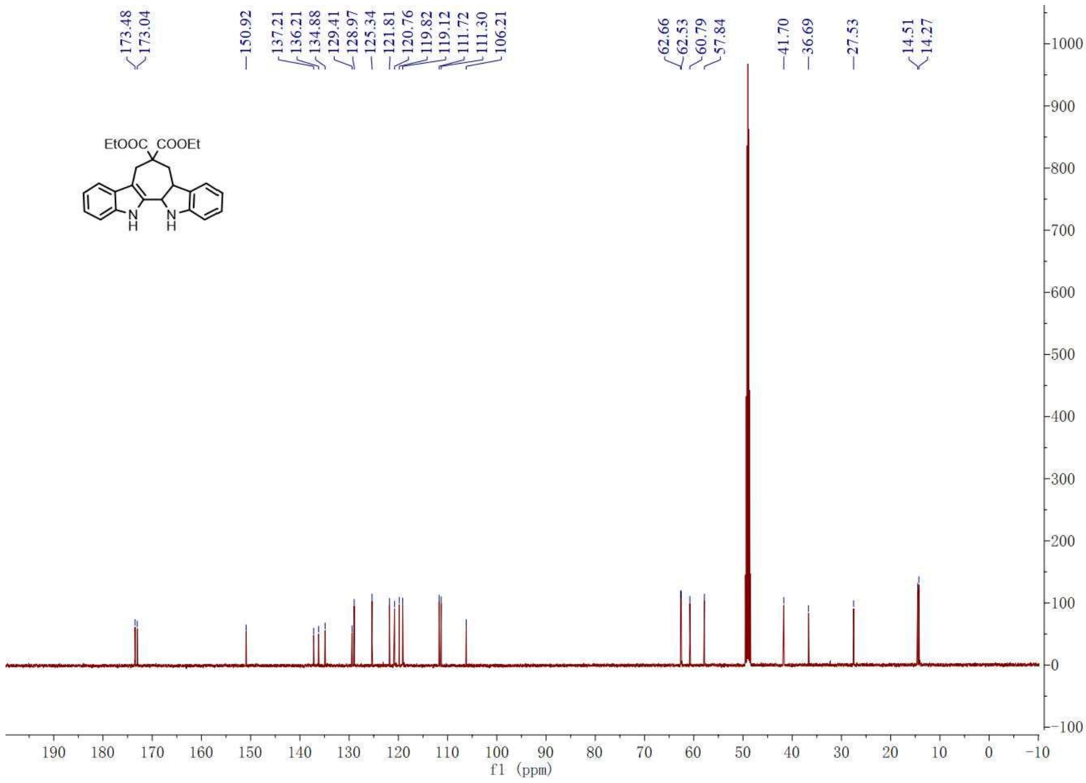

Figure S82. ^{13}C NMR spectrum of diethyl 4b,5,7,12,12b,13-hexahydro-6*H*-cyclohepta[2,1-*b*:3,4-*b'*]diindole-6,6-dicarboxylate (**3a**) in Methanol-d_4

Copies of HRMS chromatograms

Qualitative Analysis Report

**Data Filename**	ESIH_20181219_YCH_PL_21.d	**Sample Name**	PL-21
**Sample Type**	Sample	**Position**	P1-C3
**Instrument Name**	Agilent G6520 Q-TOF	**Acq Method**	20160322_MS_ESIH_POS_1min.m
**Acquired Time**	12/19/2018 11:48:24	**IRM Calibration Status**	Success
**DA Method**	small molecular data analysis method.m	**Comment**	ESIH by ZZY

User Spectra

Fragmentor Voltage	Collision Energy	Ionization Mode
125	0	ESI

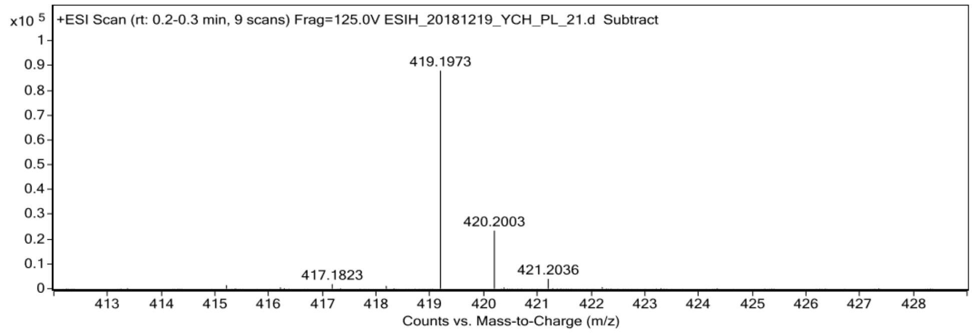

Formula Calculator Results

m/z	Calc m/z	Diff (mDa)	Diff (ppm)	Ion Formula	Ion
419.1973	419.1965	-0.75	-1.79	C25 H27 N2 O4	(M+H)+

--- End Of Report ---

Figure S83. HRMS chromatogram of diethyl 2,2-bis((1*H*-indol-3-yl)methyl)malonate (**1a**)

Qualitative Analysis Report

**Data Filename**	ESIH_20181219_YCH_PL_25.d	**Sample Name**	PL-25
**Sample Type**	Sample	**Position**	P1-C7
**Instrument Name**	Agilent G6520 Q-TOF	**Acq Method**	20160322_MS_ESIH_POS_1min.m
**Acquired Time**	12/19/2018 11:55:44	**IRM Calibration Status**	Success
**DA Method**	small molecular data analysis method.m	**Comment**	ESIH by ZZY

User Spectra

Fragmentor Voltage	Collision Energy	Ionization Mode
125	0	ESI

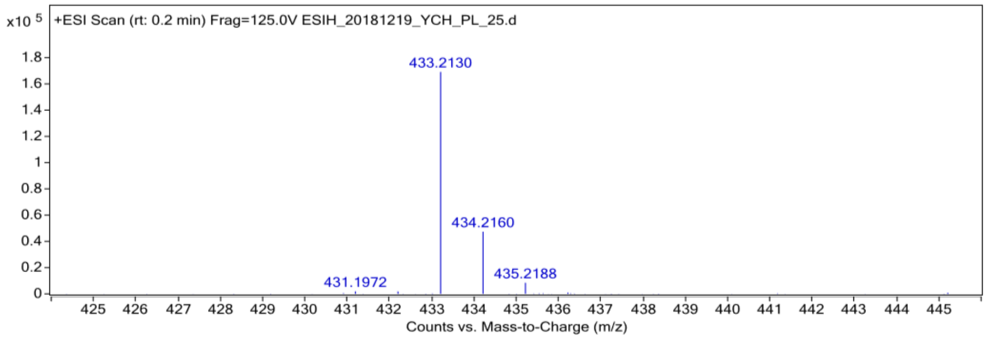

Formula Calculator Results

m/z	Calc m/z	Diff (mDa)	Diff (ppm)	Ion Formula	Ion
433.213	433.2122	-0.83	-1.92	C26 H29 N2 O4	(M+H)+

--- End Of Report ---

Figure S84. HRMS chromatogram of diethyl 2-((1*H*-indol-3-yl)methyl)-2-((5-methyl-1*H*-indol-3-yl)methyl)malonate (**1b**)

Qualitative Analysis Report

**Data Filename**	ESIH_20181219_YCH_PL_29.d	**Sample Name**	PL-29
**Sample Type**	Sample	**Position**	P1-D2
**Instrument Name**	Agilent G6520 Q-TOF	**Acq Method**	20160322_MS_ESIH_POS_1min.m
**Acquired Time**	12/19/2018 12:03:04	**IRM Calibration Status**	Success
**DA Method**	small molecular data analysis method.m	**Comment**	ESIH by ZZY

User Spectra

Fragmentor Voltage	Collision Energy	Ionization Mode
125	0	ESI

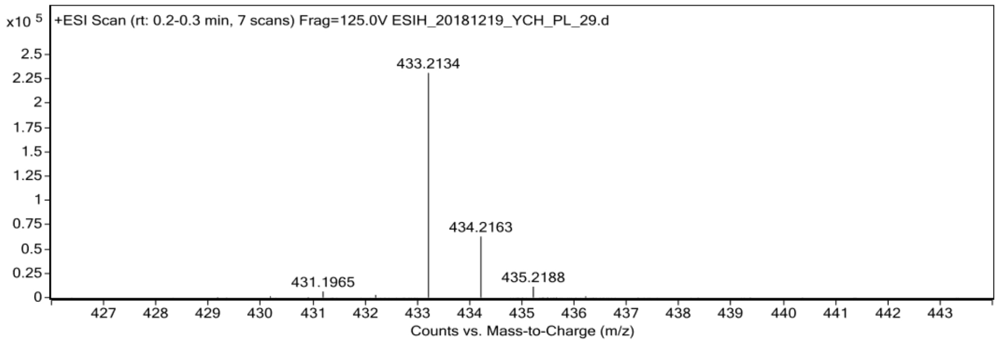

Formula Calculator Results

m/z	Calc m/z	Diff (mDa)	Diff (ppm)	Ion Formula	Ion
433.2134	433.2122	-1.17	-2.71	C26 H29 N2 O4	(M+H)+

--- End Of Report ---

Figure S85. HRMS chromatogram of diethyl 2-((1*H*-indol-3-yl)methyl)-2-((6-methyl-1*H*-indol-3-yl)methyl)malonate (**1c**)

Qualitative Analysis Report

**Data Filename**	ESIH_20181219_YCH_PL_33.d	**Sample Name**	PL-33
**Sample Type**	Sample	**Position**	P1-D6
**Instrument Name**	Agilent G6520 Q-TOF	**Acq Method**	20160322_MS_ESIH_POS_1min.m
**Acquired Time**	12/19/2018 12:10:23	**IRM Calibration Status**	Success
**DA Method**	small molecular data analysis method.m	**Comment**	ESIH by ZZY

User Spectra

Fragmentor Voltage	Collision Energy	Ionization Mode
125	0	ESI

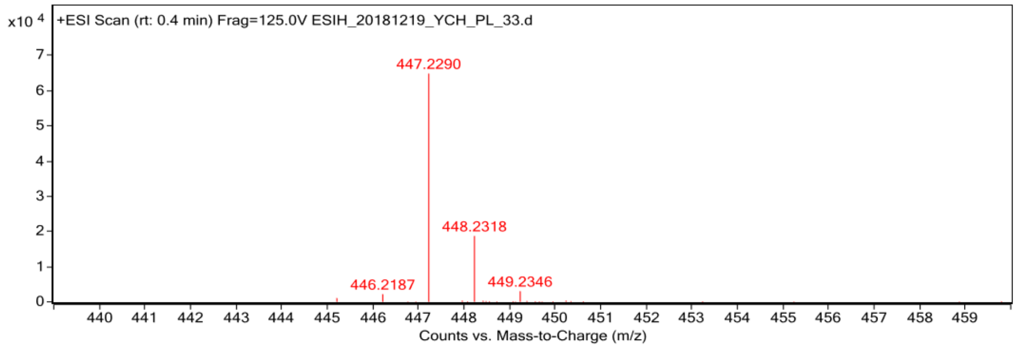

Formula Calculator Results

m/z	Calc m/z	Diff (mDa)	Diff (ppm)	Ion Formula	Ion
447.229	447.2278	-1.13	-2.52	C27 H31 N2 O4	(M+H)+

--- End Of Report ---

Figure S86. HRMS chromatogram of diethyl 2,2-bis((6-methyl-1*H*-indol-3-yl)methyl)malonate (**1d**)

Qualitative Analysis Report

**Data Filename**	ESIH_20190104_YCH_PL_03.d	**Sample Name**	7244-103
**Sample Type**	Sample	**Position**	P1-D2
**Instrument Name**	Agilent G6520 Q-TOF	**Acq Method**	20160322_MS_ESIH_POS_1min.m
**Acquired Time**	1/4/2019 14:59:32	**IRM Calibration Status**	Success
**DA Method**	small molecular data analysis method.m	**Comment**	ESIH by ZZY

User Spectra

Fragmentor Voltage	Collision Energy	Ionization Mode
225	0	ESI

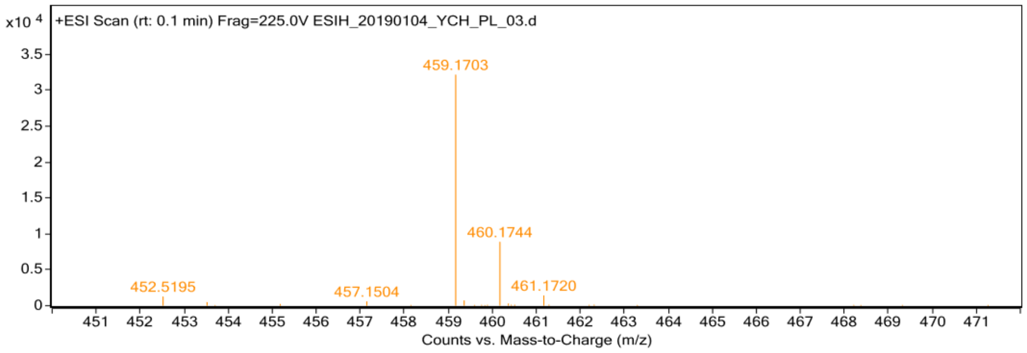

Formula Calculator Results

m/z	Calc m/z	Diff (mDa)	Diff (ppm)	Ion Formula	Ion
459.1703	459.1691	-1.22	-2.66	C25 H25 F N2 Na O4	(M+Na)+

--- End Of Report ---

Figure S87. HRMS chromatogram of diethyl 2-((1*H*-indol-3-yl)methyl)-2-((5-fluoro-1*H*-indol-3-yl)methyl)malonate (**1e**)

Qualitative Analysis Report

**Data Filename**	ESIH_20181219_YCH_PL_30.d	**Sample Name**	PL-30
**Sample Type**	Sample	**Position**	P1-D3
**Instrument Name**	Agilent G6520 Q-TOF	**Acq Method**	20160322_MS_ESIH_POS_1min.m
**Acquired Time**	12/19/2018 12:04:53	**IRM Calibration Status**	Success
**DA Method**	small molecular data analysis method.m	**Comment**	ESIH by ZZY

User Spectra

Fragmentor Voltage	Collision Energy	Ionization Mode
125	0	ESI

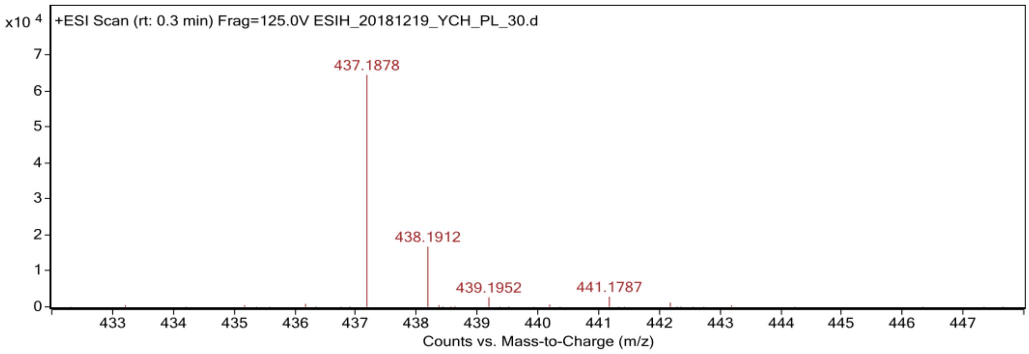

Formula Calculator Results

m/z	Calc m/z	Diff (mDa)	Diff (ppm)	Ion Formula	Ion
437.1878	437.1871	-0.65	-1.49	C25 H26 F N2 O4	(M+H)+

--- End Of Report ---

Figure S88. HRMS chromatogram of diethyl 2-((1*H*-indol-3-yl)methyl)-2-((6-fluoro-1*H*-indol-3-yl)methyl)malonate (**1f**)

Qualitative Analysis Report

**Data Filename**	ESIH_20181219_YCH_PL_35.d	**Sample Name**	PL-35
**Sample Type**	Sample	**Position**	P1-D8
**Instrument Name**	Agilent G6520 Q-TOF	**Acq Method**	20160322_MS_ESIH_POS_1min.m
**Acquired Time**	12/19/2018 12:14:04	**IRM Calibration Status**	Success
**DA Method**	small molecular data analysis method.m	**Comment**	ESIH by ZZY

User Spectra

Fragmentor Voltage	Collision Energy	Ionization Mode
125	0	ESI

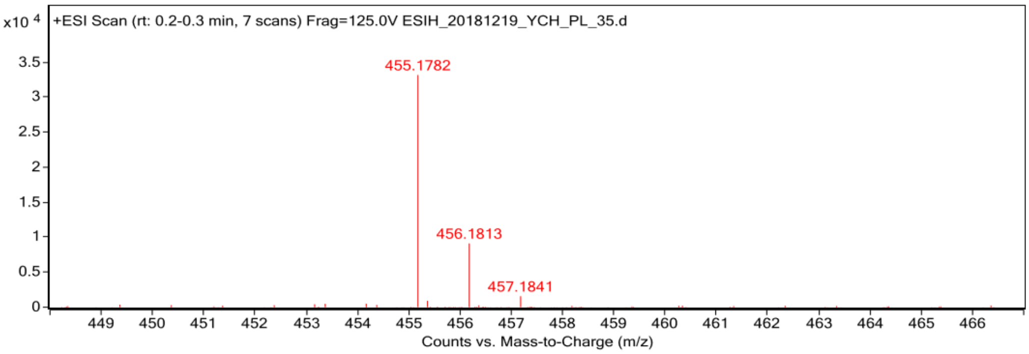

Formula Calculator Results

m/z	Calc m/z	Diff (mDa)	Diff (ppm)	Ion Formula	Ion
455.1782	455.1777	-0.55	-1.21	C25 H25 F2 N2 O4	(M+H)+

--- End Of Report ---

Figure S89. HRMS chromatogram of diethyl 2,2-bis((6-fluoro-1*H*-indol-3-yl)methyl)malonate (**1g**)

Qualitative Analysis Report

**Data Filename**	ESIH_20181219_YCH_PL_27.d	**Sample Name**	PL-27
**Sample Type**	Sample	**Position**	P1-C9
**Instrument Name**	Agilent G6520 Q-TOF	**Acq Method**	20160322_MS_ESIH_POS_1min.m
**Acquired Time**	12/19/2018 11:59:25	**IRM Calibration Status**	Success
**DA Method**	small molecular data analysis method.m	**Comment**	ESIH by ZZY

User Spectra

Fragmentor Voltage	Collision Energy	Ionization Mode
125	0	ESI

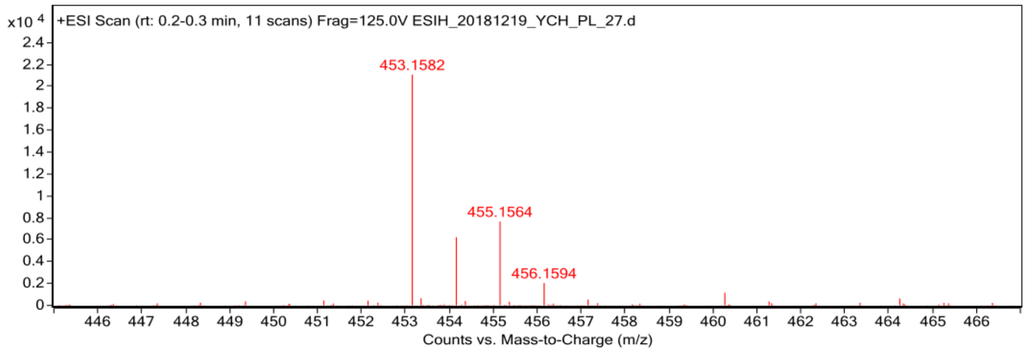

Formula Calculator Results

m/z	Calc m/z	Diff (mDa)	Diff (ppm)	Ion Formula	Ion
453.1582	453.1576	-0.62	-1.36	C25 H26 Cl N2 O4	(M+H)+

--- End Of Report ---

Figure S90. HRMS chromatogram of diethyl 2-((1*H*-indol-3-yl)methyl)-2-((5-chloro-1*H*-indol-3-yl)methyl)malonate (**1h**)

Qualitative Analysis Report

**Data Filename**	ESIH_20181219_YCH_PL_31.d	**Sample Name**	PL-31
**Sample Type**	Sample	**Position**	P1-D4
**Instrument Name**	Agilent G6520 Q-TOF	**Acq Method**	20160322_MS_ESIH_POS_1min.m
**Acquired Time**	12/19/2018 12:06:44	**IRM Calibration Status**	Success
**DA Method**	small molecular data analysis method.m	**Comment**	ESIH by ZZY

User Spectra

Fragmentor Voltage	Collision Energy	Ionization Mode
125	0	ESI

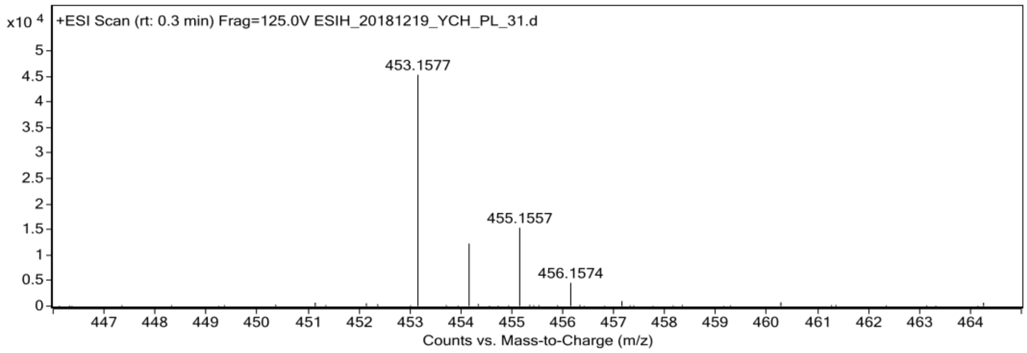

Formula Calculator Results

m/z	Calc m/z	Diff (mDa)	Diff (ppm)	Ion Formula	Ion
453.1577	453.1576	-0.12	-0.27	C25 H26 Cl N2 O4	(M+H)+

--- End Of Report ---

Figure S91. HRMS chromatogram of diethyl 2-((1*H*-indol-3-yl)methyl)-2-((6-chloro-1*H*-indol-3-

yl)methyl)malonate (**1i**)

Qualitative Analysis Report

**Data Filename**	ESIH_20181219_YCH_PL_36-1.d	**Sample Name**	PL-36
**Sample Type**	Sample	**Position**	P2-D9
**Instrument Name**	Agilent G6520 Q-TOF	**Acq Method**	20160324_MS_ESIH_NEG_1min.m
**Acquired Time**	12/19/2018 14:43:10	**IRM Calibration Status**	Success
**DA Method**	small molecular data analysis method.m	**Comment**	ESIH by ZZY

User Spectra

Fragmentor Voltage	Collision Energy	Ionization Mode
90	0	ESI

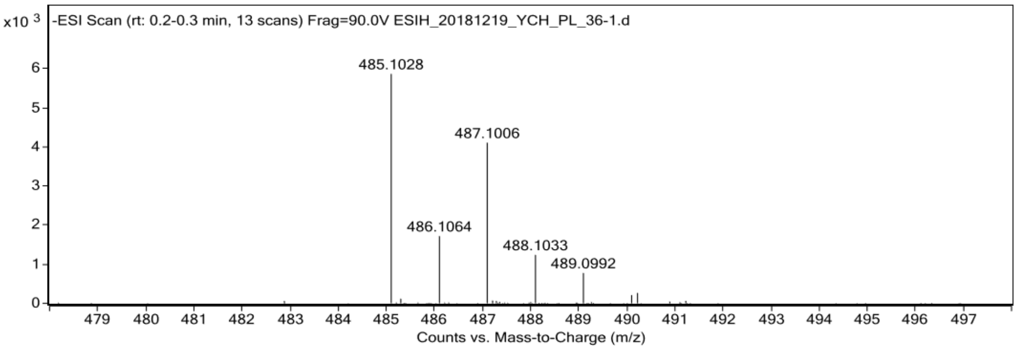

Formula Calculator Results

m/z	Calc m/z	Diff (mDa)	Diff (ppm)	Ion Formula	Ion
485.1028	485.104	1.21	2.5	C25 H23 Cl2 N2 O4	(M-H)-

--- End Of Report ---

Figure S92. HRMS chromatogram of diethyl 2,2-bis((6-chloro-1*H*-indol-3-yl)methyl)malonate (**1j**)

Qualitative Analysis Report

**Data Filename**	ESIH_20181219_YCH_PL_28.d	**Sample Name**	PL-28
**Sample Type**	Sample	**Position**	P1-D1
**Instrument Name**	Agilent G6520 Q-TOF	**Acq Method**	20160322_MS_ESIH_POS_1min.m
**Acquired Time**	12/19/2018 12:01:13	**IRM Calibration Status**	Success
**DA Method**	small molecular data analysis method.m	**Comment**	ESIH by ZZY

User Spectra

Fragmentor Voltage	Collision Energy	Ionization Mode
125	0	ESI

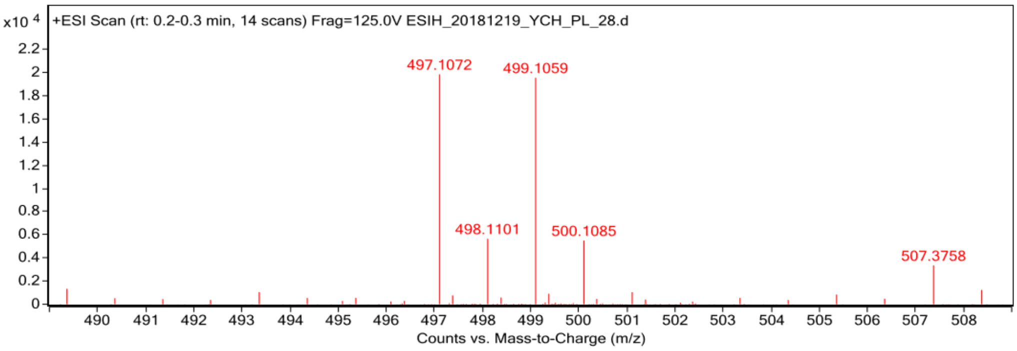

Formula Calculator Results

m/z	Calc m/z	Diff (mDa)	Diff (ppm)	Ion Formula	Ion
497.1072	497.107	-0.17	-0.33	C25 H26 Br N2 O4	(M+H)+

--- End Of Report ---

Figure S93. HRMS chromatogram of diethyl 2-((1*H*-indol-3-yl)methyl)-2-((5-bromo-1*H*-indol-3-yl)methyl)malonate (**1k**)

Qualitative Analysis Report

**Data Filename**	ESIH_20181219_YCH_PL_32.d	**Sample Name**	PL-32
**Sample Type**	Sample	**Position**	P1-D5
**Instrument Name**	Agilent G6520 Q-TOF	**Acq Method**	20160322_MS_ESIH_POS_1min.m
**Acquired Time**	12/19/2018 12:08:33	**IRM Calibration Status**	Success
**DA Method**	small molecular data analysis method.m	**Comment**	ESIH by ZZY

User Spectra

Fragmentor Voltage	Collision Energy	Ionization Mode
125	0	ESI

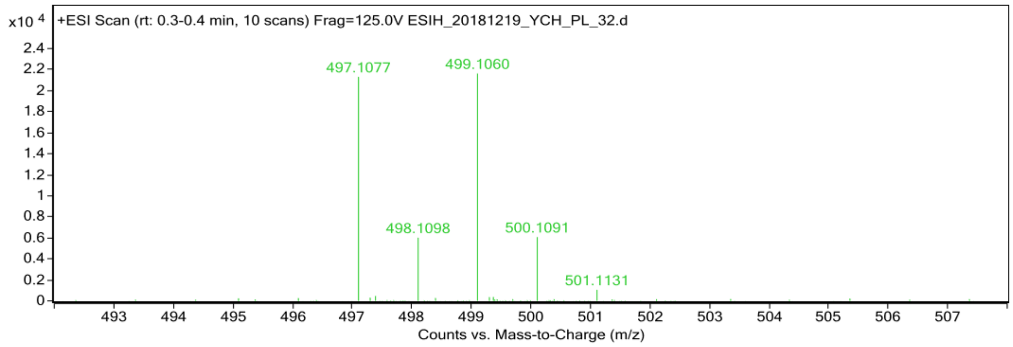

Formula Calculator Results

m/z	Calc m/z	Diff (mDa)	Diff (ppm)	Ion Formula	Ion
497.1077	497.107	-0.61	-1.23	C25 H26 Br N2 O4	(M+H)+

--- End Of Report ---

Figure S94. HRMS chromatogram of diethyl 2-((1*H*-indol-3-yl)methyl)-2-((6-bromo-1*H*-indol-3-yl)methyl)malonate (**1l**)

Qualitative Analysis Report

**Data Filename**	ESIH_20181219_YCH_PL_37-1.d	**Sample Name**	PL-37
**Sample Type**	Sample	**Position**	P2-E1
**Instrument Name**	Agilent G6520 Q-TOF	**Acq Method**	20160324_MS_ESIH_NEG_1min.m
**Acquired Time**	12/19/2018 14:45:00	**IRM Calibration Status**	Success
**DA Method**	small molecular data analysis method.m	**Comment**	ESIH by ZZY

User Spectra

Fragmentor Voltage	Collision Energy	Ionization Mode
90	0	ESI

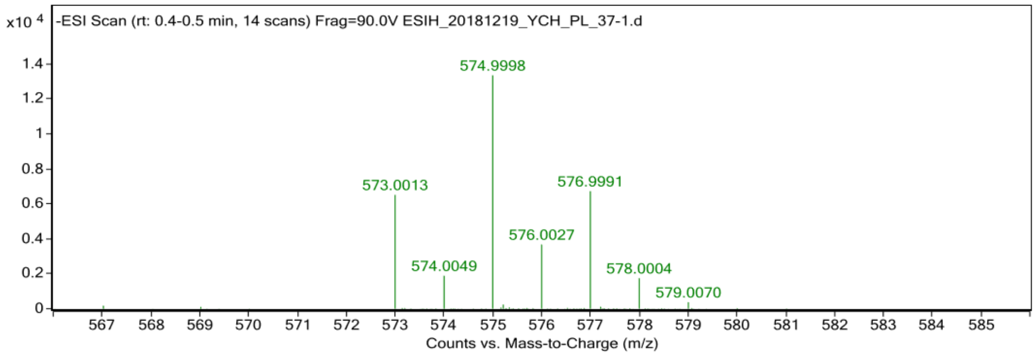

Formula Calculator Results

m/z	Calc m/z	Diff (mDa)	Diff (ppm)	Ion Formula	Ion
573.0013	573.003	1.67	2.92	C25 H23 Br2 N2 O4	(M-H)-

--- End Of Report ---

Figure S95. HRMS chromatogram of diethyl 2,2-bis((6-bromo-1*H*-indol-3-yl)methyl)malonate (**1m**)

Qualitative Analysis Report

**Data Filename**	ESIH_20181219_YCH_PL_22.d	**Sample Name**	PL-22
**Sample Type**	Sample	**Position**	P1-C4
**Instrument Name**	Agilent G6520 Q-TOF	**Acq Method**	20160322_MS_ESIH_POS_1min.m
**Acquired Time**	12/19/2018 11:50:14	**IRM Calibration Status**	Success
**DA Method**	small molecular data analysis method.m	**Comment**	ESIH by ZZY

User Spectra

Fragmentor Voltage	Collision Energy	Ionization Mode
125	0	ESI

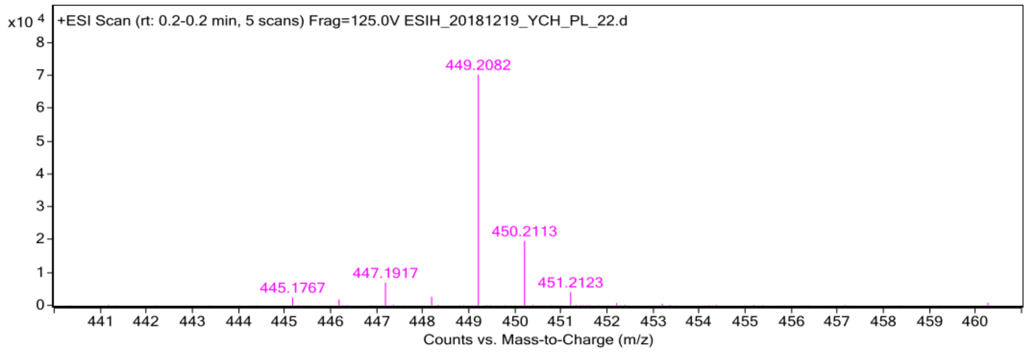

Formula Calculator Results

m/z	Calc m/z	Diff (mDa)	Diff (ppm)	Ion Formula	Ion
449.2082	449.2071	-1.09	-2.44	C26 H29 N2 O5	(M+H)+

--- End Of Report ---

Figure S96. HRMS chromatogram of diethyl 2-((1*H*-indol-3-yl)methyl)-2-((5-methoxy-1*H*-indol-3-yl)methyl)malonate (**1n**)

Qualitative Analysis Report

**Data Filename**	ESIH_20181219_YCH_PL_23.d	**Sample Name**	PL-23
**Sample Type**	Sample	**Position**	P1-C5
**Instrument Name**	Agilent G6520 Q-TOF	**Acq Method**	20160322_MS_ESIH_POS_1min.m
**Acquired Time**	12/19/2018 11:52:04	**IRM Calibration Status**	Success
**DA Method**	small molecular data analysis method.m	**Comment**	ESIH by ZZY

User Spectra

Fragmentor Voltage	Collision Energy	Ionization Mode
125	0	ESI

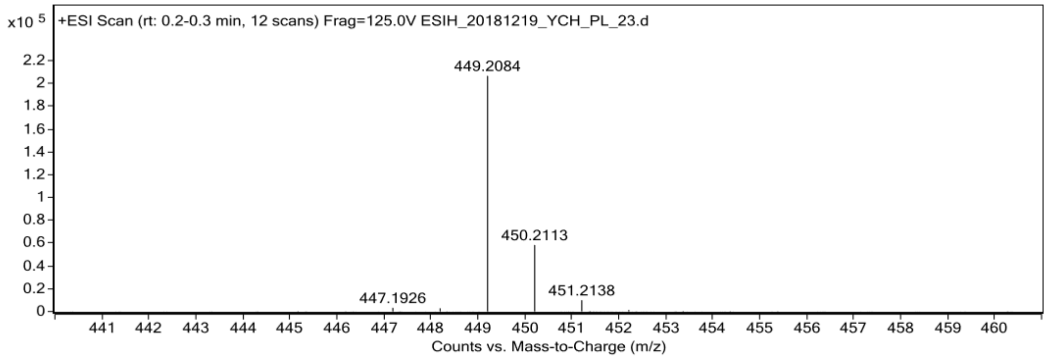

Formula Calculator Results

m/z	Calc m/z	Diff (mDa)	Diff (ppm)	Ion Formula	Ion
449.2084	449.2071	-1.34	-2.99	C26 H29 N2 O5	(M+H)+

--- End Of Report ---

Figure S97. HRMS chromatogram of diethyl 2-((1*H*-indol-3-yl)methyl)-2-((6-methoxy-1*H*-indol-3-yl)methyl)malonate (**1o**)

Qualitative Analysis Report

**Data Filename**	ESIH_20181219_YCH_PL_24.d	**Sample Name**	PL-24
**Sample Type**	Sample	**Position**	P1-C6
**Instrument Name**	Agilent G6520 Q-TOF	**Acq Method**	20160322_MS_ESIH_POS_1min.m
**Acquired Time**	12/19/2018 11:53:54	**IRM Calibration Status**	Success
**DA Method**	small molecular data analysis method.m	**Comment**	ESIH by ZZY

User Spectra

Fragmentor Voltage	Collision Energy	Ionization Mode
125	0	ESI

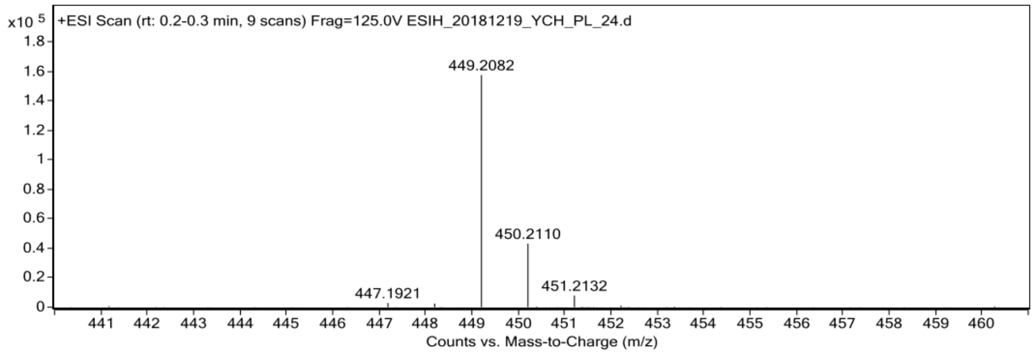

Formula Calculator Results

m/z	Calc m/z	Diff (mDa)	Diff (ppm)	Ion Formula	Ion
449.2082	449.2071	-1.15	-2.56	C26 H29 N2 O5	(M+H)+

--- End Of Report ---

Figure S98. HRMS chromatogram of diethyl 2-((1*H*-indol-3-yl)methyl)-2-((7-methoxy-1*H*-indol-3-yl)methyl)malonate (**1p**)

Qualitative Analysis Report

**Data Filename**	ESIH_20181219_YCH_PL_34.d	**Sample Name**	PL-34
**Sample Type**	Sample	**Position**	P1-D7
**Instrument Name**	Agilent G6520 Q-TOF	**Acq Method**	20160322_MS_ESIH_POS_1min.m
**Acquired Time**	12/19/2018 12:12:14	**IRM Calibration Status**	Success
**DA Method**	small molecular data analysis method.m	**Comment**	ESIH by ZZY

User Spectra

Fragmentor Voltage	Collision Energy	Ionization Mode
125	0	ESI

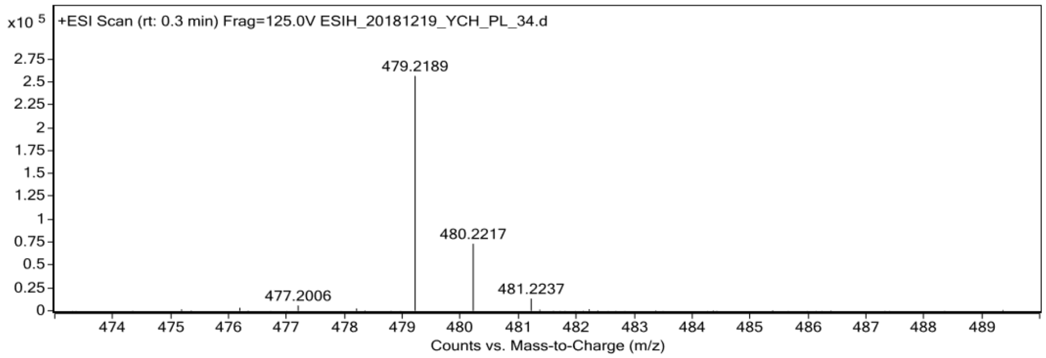

Formula Calculator Results

m/z	Calc m/z	Diff (mDa)	Diff (ppm)	Ion Formula	Ion
479.2189	479.2177	-1.21	-2.52	C27 H31 N2 O6	(M+H)+

--- End Of Report ---

Figure S99. HRMS chromatogram of diethyl 2,2-bis((6-methoxy-1*H*-indol-3-yl)methyl)malonate (**1q**)

Qualitative Analysis Report

**Data Filename**	ESIH_20181219_YCH_PL_38.d	**Sample Name**	PL-38
**Sample Type**	Sample	**Position**	P1-E2
**Instrument Name**	Agilent G6520 Q-TOF	**Acq Method**	20160322_MS_ESIH_POS_1min.m
**Acquired Time**	12/19/2018 12:19:32	**IRM Calibration Status**	Success
**DA Method**	small molecular data analysis method.m	**Comment**	ESIH by ZZY

User Spectra

Fragmentor Voltage	Collision Energy	Ionization Mode
125	0	ESI

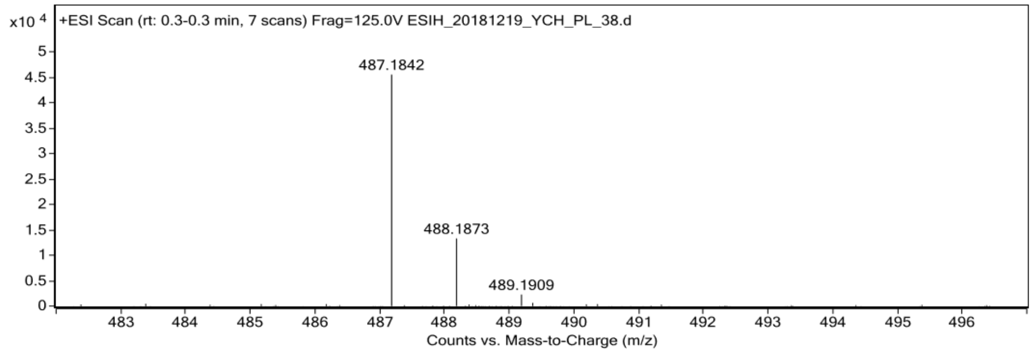

Formula Calculator Results

m/z	Calc m/z	Diff (mDa)	Diff (ppm)	Ion Formula	Ion
487.1842	487.1839	-0.32	-0.67	C26 H26 F3 N2 O4	(M+H)+

--- End Of Report ---

Figure S100. HRMS chromatogram of diethyl 2-((1*H*-indol-3-yl)methyl)-2-((5-(trifluoromethyl)-1*H*-indol-3-yl)methyl)malonate (**1r**)

Qualitative Analysis Report

**Data Filename**	ESIH_20181219_YCH_PL_39.d	**Sample Name**	PL-39
**Sample Type**	Sample	**Position**	P1-E3
**Instrument Name**	Agilent G6520 Q-TOF	**Acq Method**	20160322_MS_ESIH_POS_1min.m
**Acquired Time**	12/19/2018 12:21:22	**IRM Calibration Status**	Success
**DA Method**	small molecular data analysis method.m	**Comment**	ESIH by ZZY

User Spectra

Fragmentor Voltage	Collision Energy	Ionization Mode
125	0	ESI

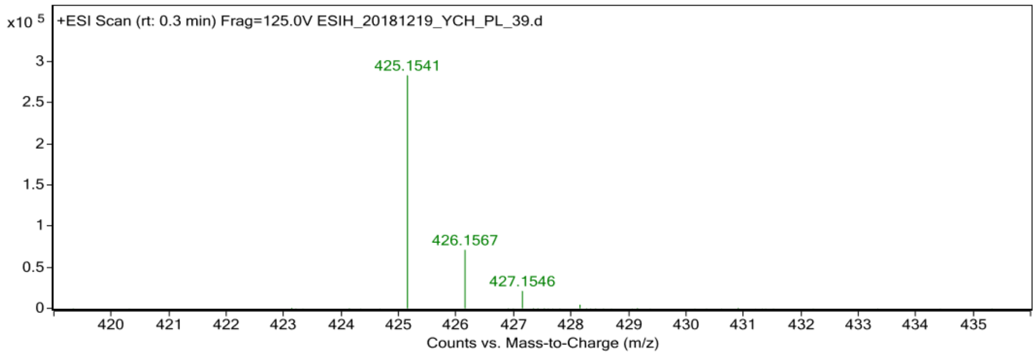

Formula Calculator Results

m/z	Calc m/z	Diff (mDa)	Diff (ppm)	Ion Formula	Ion
425.1541	425.153	-1.16	-2.73	C23 H25 N2 O4 S	(M+H)+

--- End Of Report ---

Figure S101. HRMS chromatogram of ethyl 2-((1*H*-indol-3-yl)methyl)-3-(1*H*-indol-3-yl)-2-(methylsulfonyl)propanoate (**1s**)

Qualitative Analysis Report

**Data Filename**	ESIH_20181219_YCH_PL_40.d	**Sample Name**	PL-40
**Sample Type**	Sample	**Position**	P1-E4
**Instrument Name**	Agilent G6520 Q-TOF	**Acq Method**	20160322_MS_ESIH_POS_1min.m
**Acquired Time**	12/19/2018 12:23:12	**IRM Calibration Status**	Success
**DA Method**	small molecular data analysis method.m	**Comment**	ESIH by ZZY

User Spectra

Fragmentor Voltage	Collision Energy	Ionization Mode
125	0	ESI

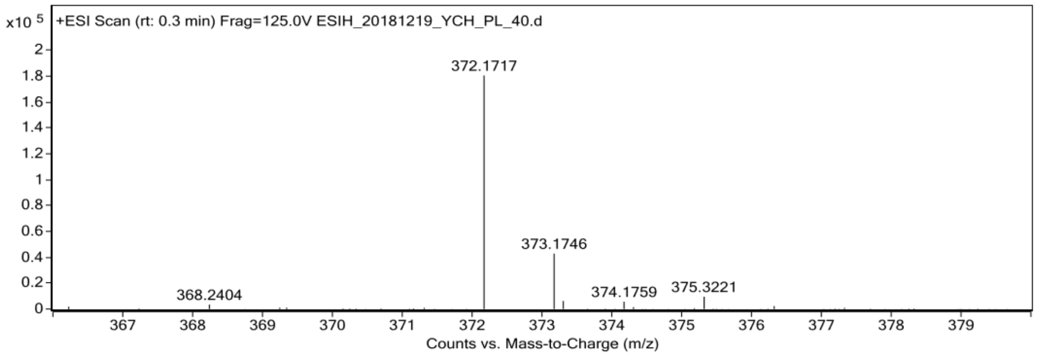

Formula Calculator Results

m/z	Calc m/z	Diff (mDa)	Diff (ppm)	Ion Formula	Ion
372.1717	372.1707	-1.08	-2.9	C23 H22 N3 O2	(M+H)+

--- End Of Report ---

Figure S102. HRMS chromatogram of ethyl 2-((1*H*-indol-3-yl)methyl)-2-cyano-3-(1*H*-indol-3-yl)propanoate (**1t**)

Qualitative Analysis Report

**Data Filename**	ESIH_20181219_YCH_PL_01.d	**Sample Name**	PL-1
**Sample Type**	Sample	**Position**	P1-A1
**Instrument Name**	Agilent G6520 Q-TOF	**Acq Method**	20160322_MS_ESIH_POS_1min.m
**Acquired Time**	12/19/2018 11:10:32	**IRM Calibration Status**	Success
**DA Method**	small molecular data analysis method.m	**Comment**	ESIH by ZZY

User Spectra

Fragmentor Voltage	Collision Energy	Ionization Mode
125	0	ESI

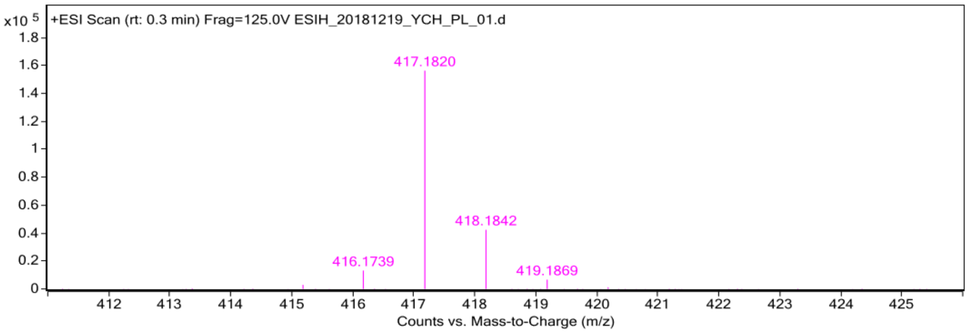

Formula Calculator Results

m/z	Calc m/z	Diff (mDa)	Diff (ppm)	Ion Formula	Ion
417.182	417.1809	-1.12	-2.69	C25 H25 N2 O4	(M+H)+

--- End Of Report ---

Figure S103. HRMS chromatogram of diethyl 5,7,12,13-tetrahydro-6*H*-cyclohepta[2,1-*b*:3,4-*b'*]diindole-6,6-dicarboxylate (**2a**)

Qualitative Analysis Report

**Data Filename**	ESIH_20181219_YCH_PL_05.d	**Sample Name**	PL-5
**Sample Type**	Sample	**Position**	P1-A5
**Instrument Name**	Agilent G6520 Q-TOF	**Acq Method**	20160322_MS_ESIH_POS_1min.m
**Acquired Time**	12/19/2018 11:19:09	**IRM Calibration Status**	Success
**DA Method**	small molecular data analysis method.m	**Comment**	ESIH by ZZY

User Spectra

Fragmentor Voltage	Collision Energy	Ionization Mode
125	0	ESI

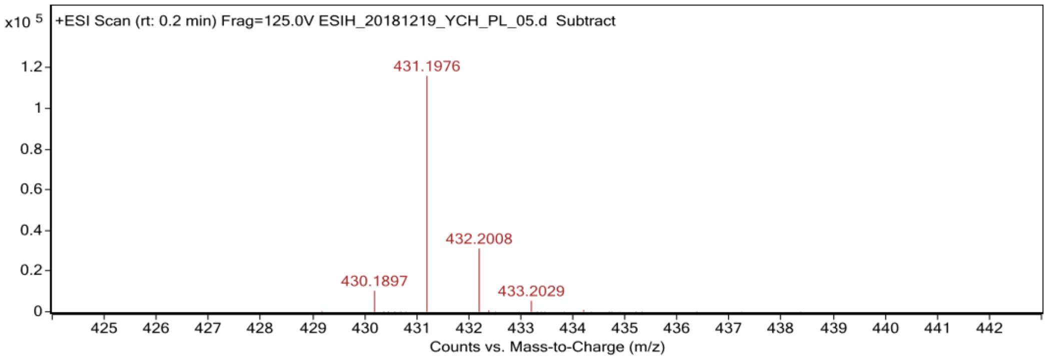

Formula Calculator Results

m/z	Calc m/z	Diff (mDa)	Diff (ppm)	Ion Formula	Ion
431.1976	431.1965	-1.09	-2.54	C26 H27 N2 O4	(M+H)+

--- End Of Report ---

Figure S104. HRMS chromatogram of diethyl 3-methyl-5,7,12,13-tetrahydro-6*H*-cyclohepta[2,1-*b*:3,4-*b'*]diindole-6,6-dicarboxylate (**2b**)

Qualitative Analysis Report

**Data Filename**	ESIH_20181219_YCH_PL_09.d	**Sample Name**	PL-9
**Sample Type**	Sample	**Position**	P1-A9
**Instrument Name**	Agilent G6520 Q-TOF	**Acq Method**	20160322_MS_ESIH_POS_1min.m
**Acquired Time**	12/19/2018 11:26:27	**IRM Calibration Status**	Success
**DA Method**	small molecular data analysis method.m	**Comment**	ESIH by ZZY

User Spectra

Fragmentor Voltage	Collision Energy	Ionization Mode
125	0	ESI

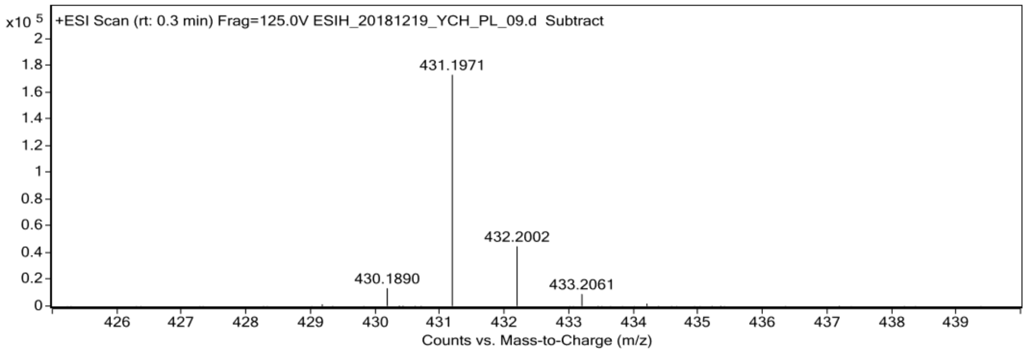

Formula Calculator Results

m/z	Calc m/z	Diff (mDa)	Diff (ppm)	Ion Formula	Ion
431.1971	431.1965	-0.58	-1.36	C26 H27 N2 O4	(M+H)+

--- End Of Report ---

Figure S105. HRMS chromatogram of diethyl 2-methyl-5,7,12,13-tetrahydro-6*H*-cyclohepta[2,1-*b*:3,4-*b'*]diindole-6,6-dicarboxylate (**2c**)

Qualitative Analysis Report

**Data Filename**	ESIH_20181219_YCH_PL_13.d	**Sample Name**	PL-13
**Sample Type**	Sample	**Position**	P1-B4
**Instrument Name**	Agilent G6520 Q-TOF	**Acq Method**	20160322_MS_ESIH_POS_1min.m
**Acquired Time**	12/19/2018 11:33:46	**IRM Calibration Status**	Success
**DA Method**	small molecular data analysis method.m	**Comment**	ESIH by ZZY

User Spectra

Fragmentor Voltage	Collision Energy	Ionization Mode
125	0	ESI

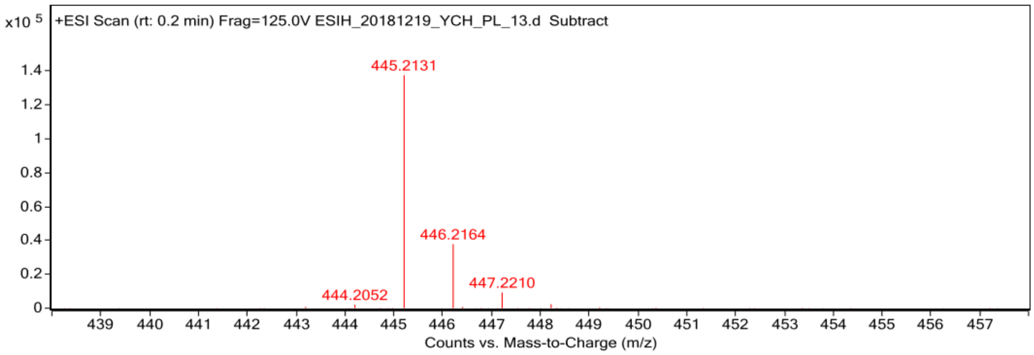

Formula Calculator Results

m/z	Calc m/z	Diff (mDa)	Diff (ppm)	Ion Formula	Ion
445.2131	445.2122	-0.92	-2.07	C27 H29 N2 O4	(M+H)+

--- End Of Report ---

Figure S106. HRMS chromatogram of diethyl 2,10-dimethyl-5,7,12,13-tetrahydro-6*H*-cyclohepta[2,1-*b*:3,4-*b'*]diindole-6,6-di carboxylate (**2d**)

Qualitative Analysis Report

**Data Filename**	ESIH_20181219_YCH_PL_06.d	**Sample Name**	PL-6
**Sample Type**	Sample	**Position**	P1-A6
**Instrument Name**	Agilent G6520 Q-TOF	**Acq Method**	20160322_MS_ESIH_POS_1min.m
**Acquired Time**	12/19/2018 11:20:59	**IRM Calibration Status**	Success
**DA Method**	small molecular data analysis method.m	**Comment**	ESIH by ZZY

User Spectra

Fragmentor Voltage	Collision Energy	Ionization Mode
125	0	ESI

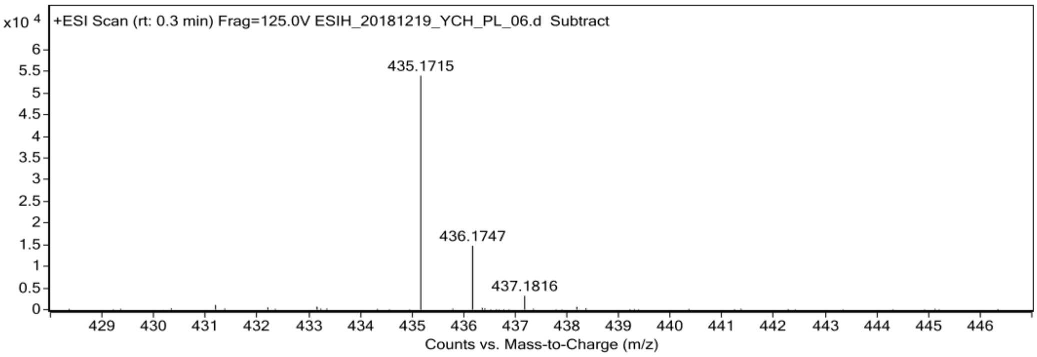

Formula Calculator Results

m/z	Calc m/z	Diff (mDa)	Diff (ppm)	Ion Formula	Ion
435.1715	435.1715	-0.03	-0.06	C25 H24 F N2 O4	(M+H)+

--- End Of Report ---

Figure S107. HRMS chromatogram of diethyl 3-fluoro-5,7,12,13-tetrahydro-6*H*-cyclohepta[2,1-*b*:3,4-*b'*]diindole-6,6-dicarboxylate (**2e**)

Qualitative Analysis Report

**Data Filename**	ESIH_20181219_YCH_PL_10.d	**Sample Name**	PL-10
**Sample Type**	Sample	**Position**	P1-B1
**Instrument Name**	Agilent G6520 Q-TOF	**Acq Method**	20160322_MS_ESIH_POS_1min.m
**Acquired Time**	12/19/2018 11:28:16	**IRM Calibration Status**	Success
**DA Method**	small molecular data analysis method.m	**Comment**	ESIH by ZZY

User Spectra

Fragmentor Voltage	Collision Energy	Ionization Mode
125	0	ESI

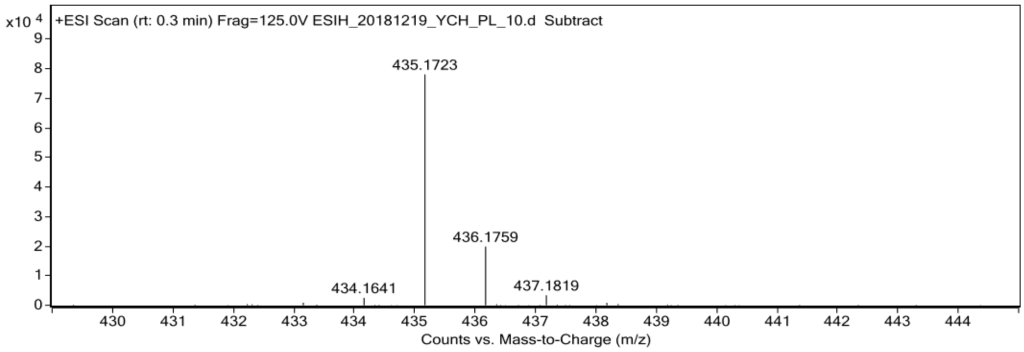

Formula Calculator Results

m/z	Calc m/z	Diff (mDa)	Diff (ppm)	Ion Formula	Ion
435.1723	435.1715	-0.81	-1.87	C25 H24 F N2 O4	(M+H)+

--- End Of Report ---

Figure S108. HRMS chromatogram of diethyl 2-fluoro-5,7,12,13-tetrahydro-6*H*-cyclohepta[2,1-*b*:3,4-*b'*]diindole-6,6-dicarboxylate (**2f**)

Qualitative Analysis Report

**Data Filename**	ESIH_20181219_YCH_PL_15.d	**Sample Name**	PL-15
**Sample Type**	Sample	**Position**	P1-B6
**Instrument Name**	Agilent G6520 Q-TOF	**Acq Method**	20160322_MS_ESIH_POS_1min.m
**Acquired Time**	12/19/2018 11:37:26	**IRM Calibration Status**	Success
**DA Method**	small molecular data analysis method.m	**Comment**	ESIH by ZZY

User Spectra

Fragmentor Voltage	Collision Energy	Ionization Mode
125	0	ESI

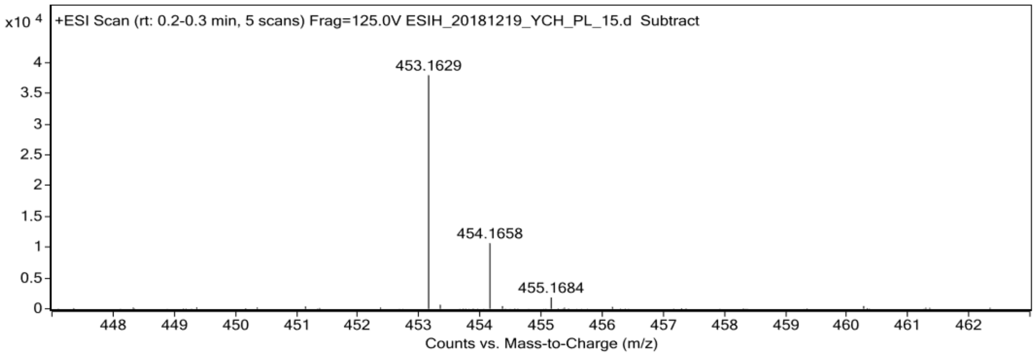

Formula Calculator Results

m/z	Calc m/z	Diff (mDa)	Diff (ppm)	Ion Formula	Ion
453.1629	453.162	-0.83	-1.83	C25 H23 F2 N2 O4	(M+H)+

--- End Of Report ---

Figure S109. HRMS chromatogram of diethyl 2,10-difluoro-5,7,12,13-tetrahydro-6*H*-cyclohepta[2,1-*b*:3,4-*b'*]diindole-6,6-dicarboxylate (**2g**)

Qualitative Analysis Report

**Data Filename**	ESIH_20181219_YCH_PL_07.d	**Sample Name**	PL-7
**Sample Type**	Sample	**Position**	P1-A7
**Instrument Name**	Agilent G6520 Q-TOF	**Acq Method**	20160322_MS_ESIH_POS_1min.m
**Acquired Time**	12/19/2018 11:22:49	**IRM Calibration Status**	Success
**DA Method**	small molecular data analysis method.m	**Comment**	ESIH by ZZY

User Spectra

Fragmentor Voltage	Collision Energy	Ionization Mode
125	0	ESI

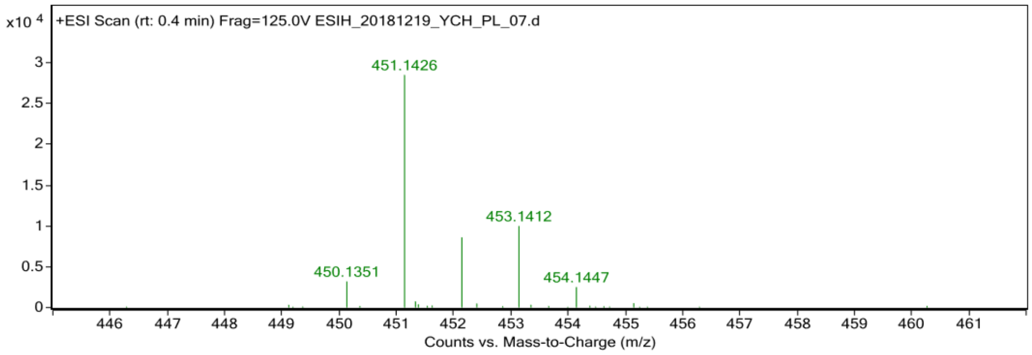

Formula Calculator Results

m/z	Calc m/z	Diff (mDa)	Diff (ppm)	Ion Formula	Ion
451.1426	451.1419	-0.72	-1.6	C25 H24 Cl N2 O4	(M+H)+

--- End Of Report ---

Figure S110. HRMS chromatogram of diethyl 3-chloro-5,7,12,13-tetrahydro-6*H*-cyclohepta[2,1-*b*:3,4-*b'*]diindole-6,6-dicarboxylate (**2h**)

Qualitative Analysis Report

**Data Filename**	ESIH_20181219_YCH_PL_11.d	**Sample Name**	PL-11
**Sample Type**	Sample	**Position**	P1-B2
**Instrument Name**	Agilent G6520 Q-TOF	**Acq Method**	20160322_MS_ESIH_POS_1min.m
**Acquired Time**	12/19/2018 11:30:07	**IRM Calibration Status**	Success
**DA Method**	small molecular data analysis method.m	**Comment**	ESIH by ZZY

User Spectra

Fragmentor Voltage	Collision Energy	Ionization Mode
125	0	ESI

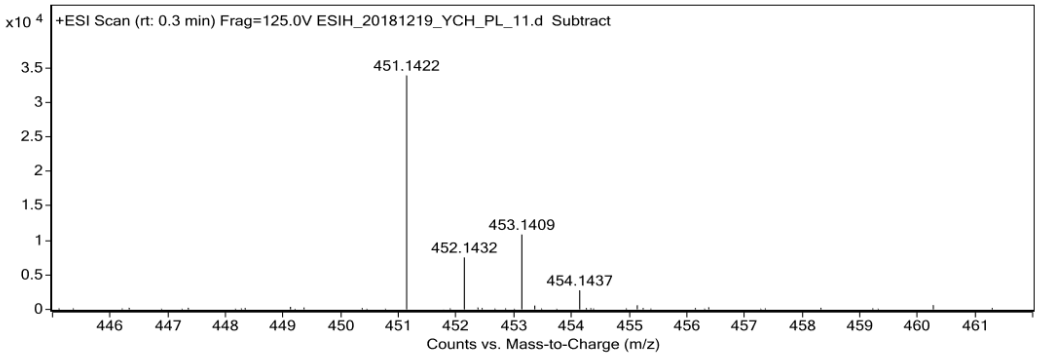

Formula Calculator Results

m/z	Calc m/z	Diff (mDa)	Diff (ppm)	Ion Formula	Ion
451.1422	451.1419	-0.29	-0.64	C25 H24 Cl N2 O4	(M+H)+

--- End Of Report ---

Figure S111. HRMS chromatogram of diethyl 2-chloro-5,7,12,13-tetrahydro-6*H*-cyclohepta[2,1-*b*:3,4-*b'*]diindole-6,6-dicarboxylate (**2i**)

Qualitative Analysis Report

**Data Filename**	ESIH_20181219_YCH_PL_16-1.d	**Sample Name**	PL-16
**Sample Type**	Sample	**Position**	P2-B7
**Instrument Name**	Agilent G6520 Q-TOF	**Acq Method**	20160324_MS_ESIH_NEG_1min.m
**Acquired Time**	12/19/2018 14:37:41	**IRM Calibration Status**	Success
**DA Method**	small molecular data analysis method.m	**Comment**	ESIH by ZZY

User Spectra

Fragmentor Voltage	Collision Energy	Ionization Mode
90	0	ESI

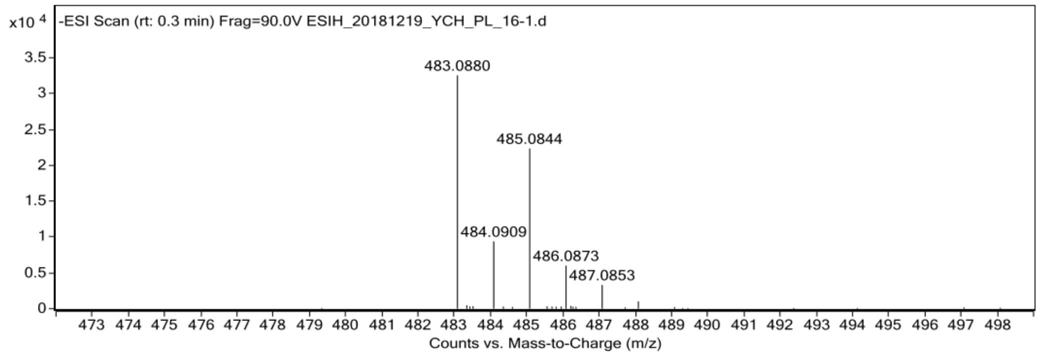

Formula Calculator Results

m/z	Calc m/z	Diff (mDa)	Diff (ppm)	Ion Formula	Ion
483.088	483.0884	0.36	0.74	C25 H21 Cl2 N2 O4	(M-H)-

--- End Of Report ---

Figure S112. HRMS chromatogram of diethyl 2,10-dichloro-5,7,12,13-tetrahydro-6*H*-cyclohepta[2,1-*b*:3,4-*b'*]diindole-6,6-di carboxylate (**2j**)

Qualitative Analysis Report

**Data Filename**	ESIH_20181219_YCH_PL_08.d	**Sample Name**	PL-8
**Sample Type**	Sample	**Position**	P1-A8
**Instrument Name**	Agilent G6520 Q-TOF	**Acq Method**	20160322_MS_ESIH_POS_1min.m
**Acquired Time**	12/19/2018 11:24:39	**IRM Calibration Status**	Success
**DA Method**	small molecular data analysis method.m	**Comment**	ESIH by ZZY

User Spectra

Fragmentor Voltage	Collision Energy	Ionization Mode
125	0	ESI

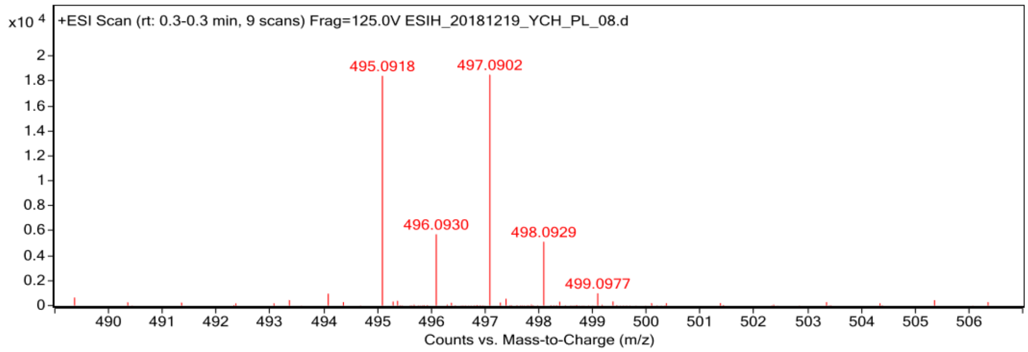

Formula Calculator Results

m/z	Calc m/z	Diff (mDa)	Diff (ppm)	Ion Formula	Ion
495.0918	495.0914	-0.41	-0.82	C25 H24 Br N2 O4	(M+H)+

--- End Of Report ---

Figure S113. HRMS chromatogram of diethyl 3-bromo-5,7,12,13-tetrahydro-6*H*-cyclohepta[2,1-*b*:3,4-*b'*]diindole-6,6-dicarboxylate (**2k**)

Qualitative Analysis Report

**Data Filename**	ESIH_20181219_YCH_PL_12.d	**Sample Name**	PL-12
**Sample Type**	Sample	**Position**	P1-B3
**Instrument Name**	Agilent G6520 Q-TOF	**Acq Method**	20160322_MS_ESIH_POS_1min.m
**Acquired Time**	12/19/2018 11:31:56	**IRM Calibration Status**	Success
**DA Method**	small molecular data analysis method.m	**Comment**	ESIH by ZZY

User Spectra

Fragmentor Voltage	Collision Energy	Ionization Mode
125	0	ESI

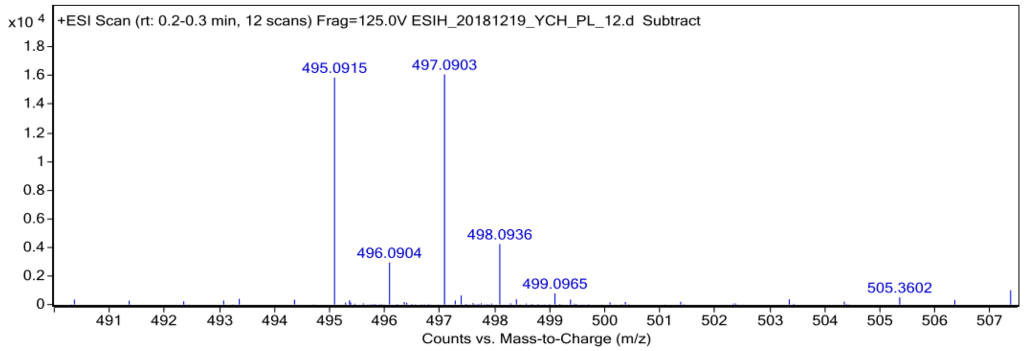

Formula Calculator Results

m/z	Calc m/z	Diff (mDa)	Diff (ppm)	Ion Formula	Ion
495.0915	495.0914	-0.1	-0.2	C25 H24 Br N2 O4	(M+H)+

--- End Of Report ---

Figure S114. HRMS chromatogram of diethyl 2-bromo-5,7,12,13-tetrahydro-6*H*-cyclohepta[2,1-*b*:3,4-*b'*]diindole-6,6-dicarboxylate (**2l**)

Qualitative Analysis Report

**Data Filename**	ESIH_20181219_YCH_PL_17-1.d	**Sample Name**	PL-17
**Sample Type**	Sample	**Position**	P2-B8
**Instrument Name**	Agilent G6520 Q-TOF	**Acq Method**	20160324_MS_ESIH_NEG_1min.m
**Acquired Time**	12/19/2018 14:39:30	**IRM Calibration Status**	Success
**DA Method**	small molecular data analysis method.m	**Comment**	ESIH by ZZY

User Spectra

Fragmentor Voltage	Collision Energy	Ionization Mode
90	0	ESI

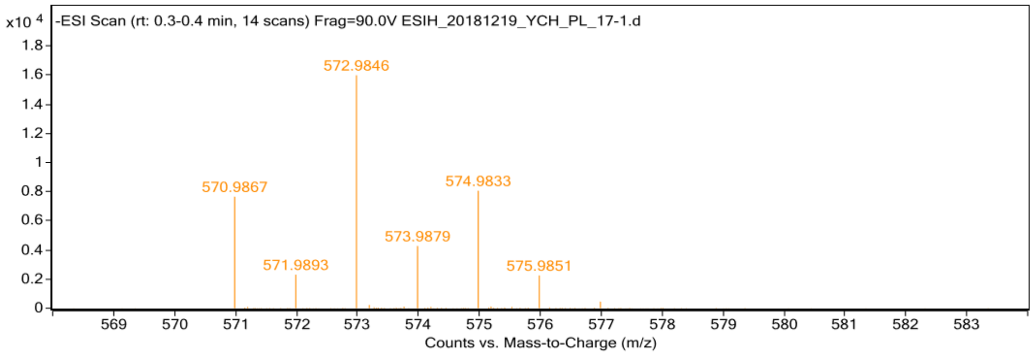

Formula Calculator Results

m/z	Calc m/z	Diff (mDa)	Diff (ppm)	Ion Formula	Ion
570.9867	570.9874	0.65	1.14	C25 H21 Br2 N2 O4	(M-H)-

--- End Of Report ---

Figure S115. HRMS chromatogram of diethyl 2,10-dibromo-5,7,12,13-tetrahydro-6*H*-cyclohepta[2,1-*b*:3,4-*b'*]diindole-6,6-di carboxylate (**2m**)

Qualitative Analysis Report

**Data Filename**	ESIH_20181219_YCH_PL_02.d	**Sample Name**	PL-2
**Sample Type**	Sample	**Position**	P1-A2
**Instrument Name**	Agilent G6520 Q-TOF	**Acq Method**	20160322_MS_ESIH_POS_1min.m
**Acquired Time**	12/19/2018 11:13:39	**IRM Calibration Status**	Success
**DA Method**	small molecular data analysis method.m	**Comment**	ESIH by ZZY

User Spectra

Fragmentor Voltage	Collision Energy	Ionization Mode
125	0	ESI

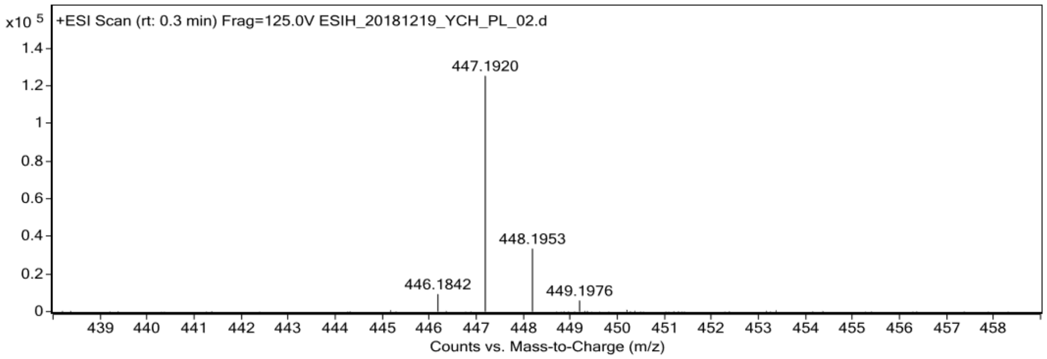

Formula Calculator Results

m/z	Calc m/z	Diff (mDa)	Diff (ppm)	Ion Formula	Ion
447.192	447.1914	-0.59	-1.31	C26 H27 N2 O5	(M+H)+

--- End Of Report ---

Figure S116. HRMS chromatogram of diethyl 3-methoxy-5,7,12,13-tetrahydro-6*H*-cyclohepta[2,1-*b*:3,4-*b'*]diindole-6,6-dicarboxylate (**2n**)

Qualitative Analysis Report

**Data Filename**	ESIH_20181219_YCH_PL_03.d	**Sample Name**	PL-3
**Sample Type**	Sample	**Position**	P1-A3
**Instrument Name**	Agilent G6520 Q-TOF	**Acq Method**	20160322_MS_ESIH_POS_1min.m
**Acquired Time**	12/19/2018 11:15:29	**IRM Calibration Status**	Success
**DA Method**	small molecular data analysis method.m	**Comment**	ESIH by ZZY

User Spectra

Fragmentor Voltage	Collision Energy	Ionization Mode
125	0	ESI

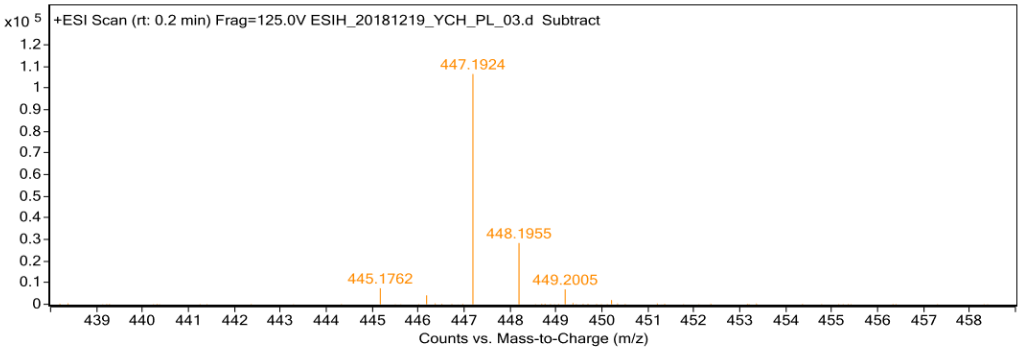

Formula Calculator Results

m/z	Calc m/z	Diff (mDa)	Diff (ppm)	Ion Formula	Ion
447.1924	447.1914	-0.92	-2.06	C26 H27 N2 O5	(M+H)+

--- End Of Report ---

Figure S117. HRMS chromatogram of diethyl 2-methoxy-5,7,12,13-tetrahydro-6*H*-cyclohepta[2,1-*b*:3,4-*b'*]diindole-6,6-dicarboxylate (**2o**)

Qualitative Analysis Report

**Data Filename**	ESIH_20181219_YCH_PL_04.d	**Sample Name**	PL-4
**Sample Type**	Sample	**Position**	P1-A4
**Instrument Name**	Agilent G6520 Q-TOF	**Acq Method**	20160322_MS_ESIH_POS_1min.m
**Acquired Time**	12/19/2018 11:17:19	**IRM Calibration Status**	Success
**DA Method**	small molecular data analysis method.m	**Comment**	ESIH by ZZY

User Spectra

Fragmentor Voltage	Collision Energy	Ionization Mode
125	0	ESI

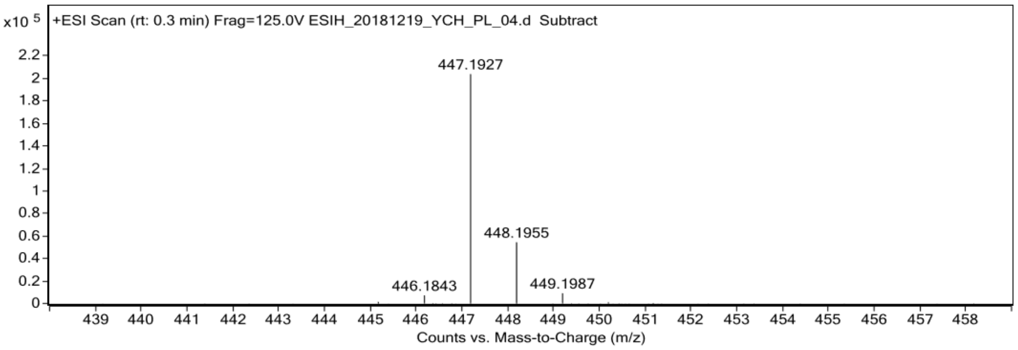

Formula Calculator Results

m/z	Calc m/z	Diff (mDa)	Diff (ppm)	Ion Formula	Ion
447.1927	447.1914	-1.28	-2.86	C26 H27 N2 O5	(M+H)+

--- End Of Report ---

Figure S118. HRMS chromatogram of diethyl 1-methoxy-5,7,12,13-tetrahydro-6*H*-cyclohepta[2,1-*b*:3,4-*b'*]diindole-6,6-dicarboxylate (**2p**)

Qualitative Analysis Report

**Data Filename**	ESIH_20181219_YCH_PL_14.d	**Sample Name**	PL-14
**Sample Type**	Sample	**Position**	P1-B5
**Instrument Name**	Agilent G6520 Q-TOF	**Acq Method**	20160322_MS_ESIH_POS_1min.m
**Acquired Time**	12/19/2018 11:35:36	**IRM Calibration Status**	Success
**DA Method**	small molecular data analysis method.m	**Comment**	ESIH by ZZY

User Spectra

Fragmentor Voltage	Collision Energy	Ionization Mode
125	0	ESI

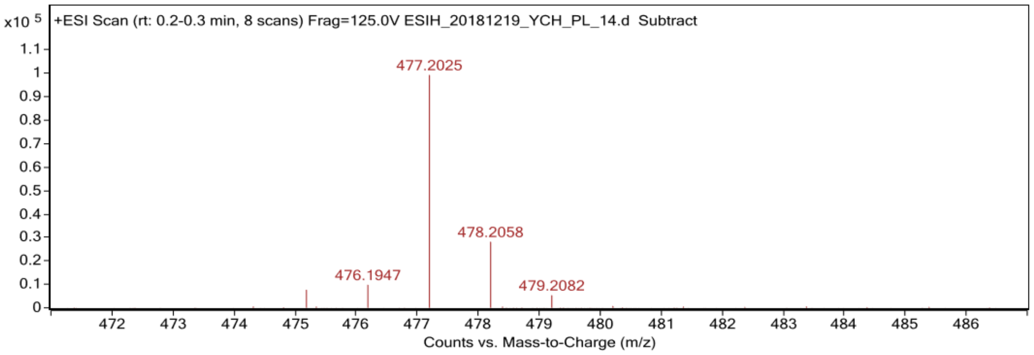

Formula Calculator Results

m/z	Calc m/z	Diff (mDa)	Diff (ppm)	Ion Formula	Ion
477.2025	477.202	-0.52	-1.1	C27 H29 N2 O6	(M+H)+

--- End Of Report ---

Figure S119. HRMS chromatogram of diethyl 2,10-dimethoxy-5,7,12,13-tetrahydro-6*H*-cyclohepta[2,1-*b*:3,4-*b'*]diindole-6,6-di carboxylate (**2q**)

Qualitative Analysis Report

**Data Filename**	ESIH_20181219_YCH_PL_18.d	**Sample Name**	PL-18
**Sample Type**	Sample	**Position**	P1-B9
**Instrument Name**	Agilent G6520 Q-TOF	**Acq Method**	20160322_MS_ESIH_POS_1min.m
**Acquired Time**	12/19/2018 11:42:55	**IRM Calibration Status**	Success
**DA Method**	small molecular data analysis method.m	**Comment**	ESIH by ZZY

User Spectra

Fragmentor Voltage	Collision Energy	Ionization Mode
125	0	ESI

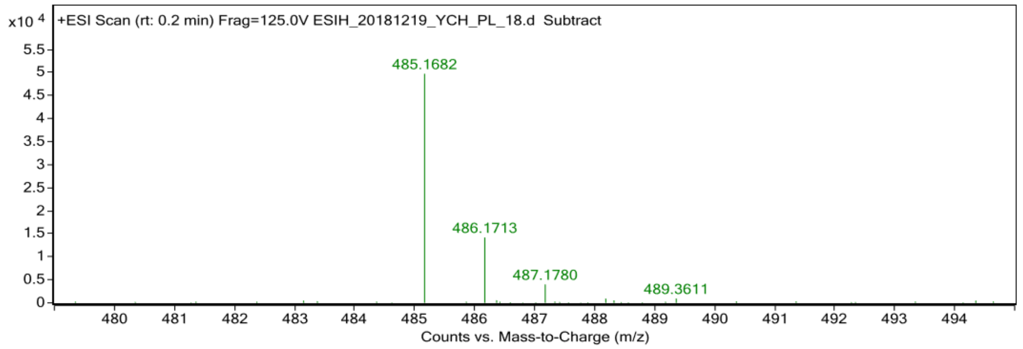

Formula Calculator Results

m/z	Calc m/z	Diff (mDa)	Diff (ppm)	Ion Formula	Ion
485.1682	485.1683	0.07	0.15	C26 H24 F3 N2 O4	(M+H)+

--- End Of Report ---

Figure S120. HRMS chromatogram of diethyl 3-(trifluoromethyl)-5,7,12,13-tetrahydro-6*H*-cyclohepta[2,1-*b*:3,4-*b'*]diindole-6,6-di carboxylate (**2r**)

Qualitative Analysis Report

**Data Filename**	ESIH_20181219_YCH_PL_19.d	**Sample Name**	PL-19
**Sample Type**	Sample	**Position**	P1-C1
**Instrument Name**	Agilent G6520 Q-TOF	**Acq Method**	20160322_MS_ESIH_POS_1min.m
**Acquired Time**	12/19/2018 11:44:44	**IRM Calibration Status**	Success
**DA Method**	small molecular data analysis method.m	**Comment**	ESIH by ZZY

User Spectra

Fragmentor Voltage	Collision Energy	Ionization Mode
125	0	ESI

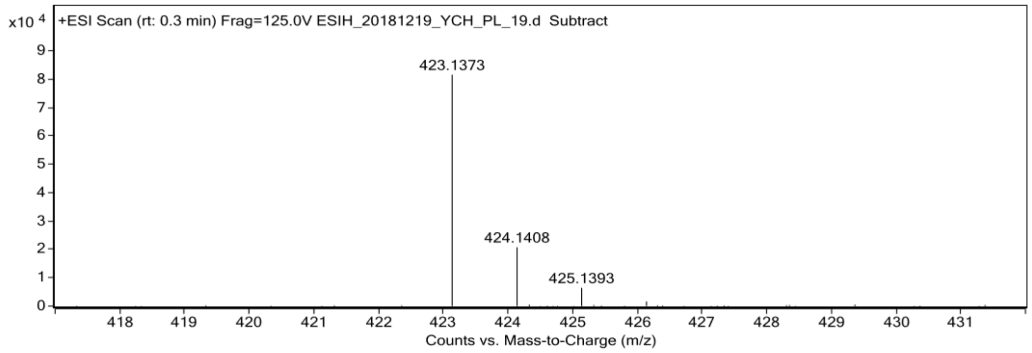

Formula Calculator Results

m/z	Calc m/z	Diff (mDa)	Diff (ppm)	Ion Formula	Ion
423.1373	423.1373	0.03	0.07	C23 H23 N2 O4 S	(M+H)+

--- End Of Report ---

Figure S121. HRMS chromatogram of ethyl 6-(methylsulfonyl)-6,7,12,13-tetrahydro-5*H*-cyclohepta[2,1-*b*:3,4-*b'*]diindole-6-carboxylate (**2s**)

Qualitative Analysis Report

**Data Filename**	ESIH_20181219_YCH_PL_20.d	**Sample Name**	PL-20
**Sample Type**	Sample	**Position**	P1-C2
**Instrument Name**	Agilent G6520 Q-TOF	**Acq Method**	20160322_MS_ESIH_POS_1min.m
**Acquired Time**	12/19/2018 11:46:34	**IRM Calibration Status**	Success
**DA Method**	small molecular data analysis method.m	**Comment**	ESIH by ZZY

User Spectra

Fragmentor Voltage	Collision Energy	Ionization Mode
125	0	ESI

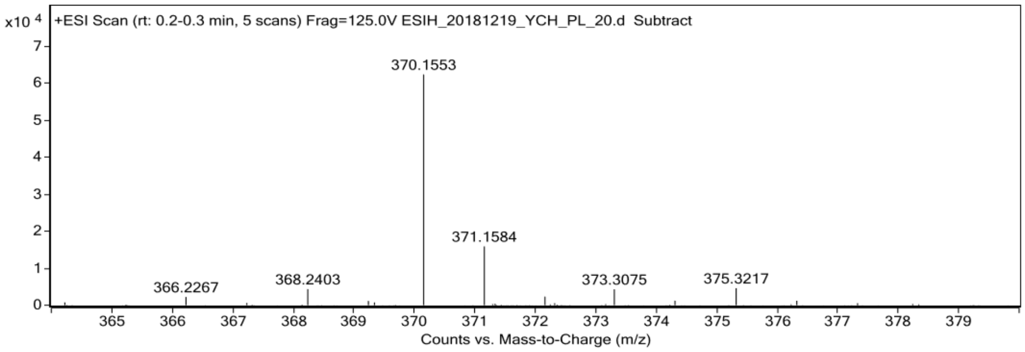

Formula Calculator Results

m/z	Calc m/z	Diff (mDa)	Diff (ppm)	Ion Formula	Ion
370.1553	370.155	-0.25	-0.67	C23 H20 N3 O2	(M+H)+

--- End Of Report ---

Figure S122. HRMS chromatogram of ethyl 6-cyano-6,7,12,13-tetrahydro-5*H*-cyclohepta[2,1-*b*:3,4-*b'*]diindole-6-carboxylate (**2t**)

Qualitative Analysis Report

**Data Filename**	ESIH_20190104_YCH_PL_02.d	**Sample Name**	PL-3a
**Sample Type**	Sample	**Position**	P1-D1
**Instrument Name**	Agilent G6520 Q-TOF	**Acq Method**	20160322_MS_ESIH_POS_1min.m
**Acquired Time**	1/4/2019 14:57:09	**IRM Calibration Status**	Success
**DA Method**	small molecular data analysis method.m	**Comment**	ESIH by ZZY

User Spectra

Fragmentor Voltage	Collision Energy	Ionization Mode
225	0	ESI

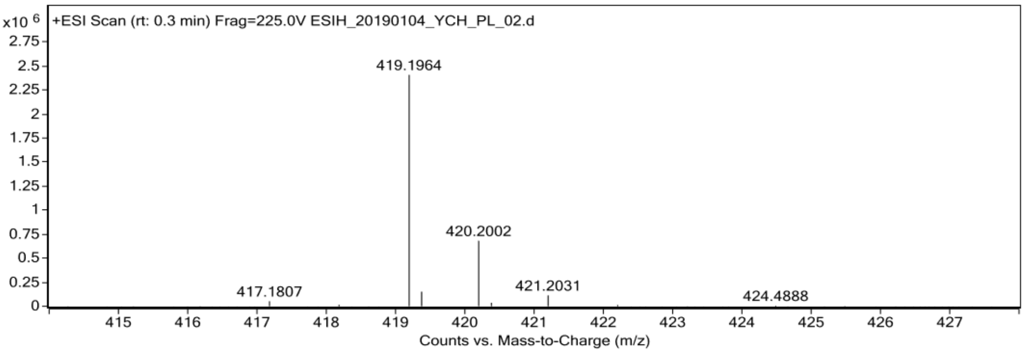

Formula Calculator Results

m/z	Calc m/z	Diff (mDa)	Diff (ppm)	Ion Formula	Ion
419.1964	419.1965	0.17	0.4	C25 H27 N2 O4	(M+H)+

--- End Of Report ---

Figure S123. HRMS chromatogram of diethyl 4b,5,7,12,12b,13-hexahydro-6*H*-cyclohepta[2,1-*b*:3,4-*b'*]diindole-6,6-dicarboxylate (**3a**)
